# Camphor-Based NHC Ligands with a Sulfur Ligator Atom in Rhodium Catalysis: Catalytic Advances in the Asymmetric Ring Opening of *N*‑Protected Azabenzonorbornenes

**DOI:** 10.1021/acs.orglett.5c02110

**Published:** 2025-07-25

**Authors:** Daniel Kamzol, Mohsen Bahramiveleshkolaei, René Wilhelm

**Affiliations:** Institute of Organic Chemistry, 26534 Clausthal University of Technology, Leibnizstr. 6, 38678, Clausthal-Zellerfeld, Germany

## Abstract

In this study, we report the synthesis, characterization, and reactivity assessment of a chiral camphor-based Rh­(I) catalyst functionalized with a sulfur moiety. The catalytic system was evaluated in the asymmetric ring-opening (ARO) of *N*-protected bicyclic substrates, with ensured high product yields and enantioselectivity. In addition to the application of various indoles as nucleophiles in the ARO reaction, a broad scope of bicyclic substrates was also explored. The catalyst demonstrated tolerance toward various functional groups present in indole derivatives, highlighting its versatility and potential applicability in asymmetric synthesis.

## Graphical abstract

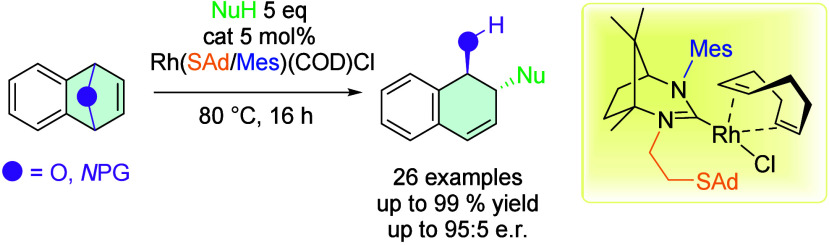

The ring-opening reaction of oxa- and azabenzononbornenes^1–3^ can provide a diverse range of products, facilitating the total synthesis of various important pharmaceuticals and natural products. Notably, among these products are derivatives of conduritol and inositol.^4–7^ Conduritol derivatives have been identified as effective inhibitors of glycosidases and exhibit antibiotic, antileukemic, and growth-regulating activities. Furthermore, they have demonstrated potent inhibitory effects against human immunodeficiency virus (HIV). Closely related derivatives, such as dihydronaphthalene and tetrahydronaphthalene, also exhibit significant biological activity, as illustrated in Scheme 1. μ-Opioid ligand as well as κ-opioid analgesics are potent centrally acting analgesics.^8,9^ Homohelidonine and chelidonine have a range of proposed pharmacological activities including tubulin interaction within target cells causing mitotic arrest.^10,11^ Sertaline was described as an antidepressant medication of the selective serotonin reuptake inhibitor (SSRI) class.^12^ Extracted from the plant *Narcissus*, narciclasine has shown an activity for treatment against cancer, in particular uterine tumors.^13–15^ *C*-Mannosyltryptophan and its glycoside analogues were found to be inhibitors of the sodium-dependent glucose cotransporter 2 (SGLT2) channel, and their prospective in the therapy of type 2 diabetes has been reported.^16^

**Scheme 1:**
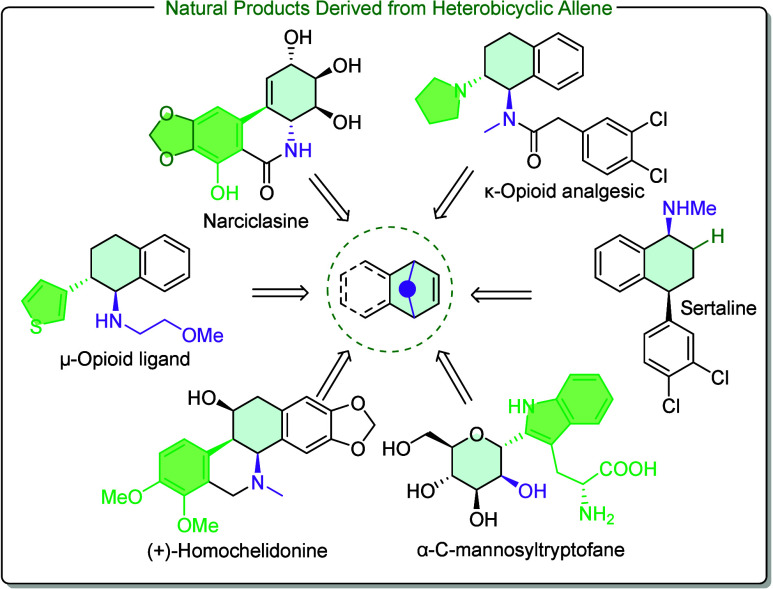
Bioactive Derivatives of Dihydronaphthalene

To ensure the appropriate stereochemistry of the ring-opening product, methods based on asymmetric ring-opening catalysis are most often employed. Such asymmetric ring-opening (ARO) reactions allow for high yields as well as suitably high enantiomeric purity of the product. Among all ARO reactions involving bicyclic systems, the ARO reactions involving azabicyclic systems are less well documented in the literature. One of the earliest examples of an *N*-protected bicyclic system undergoing ARO was reported by Lautens,^17^ where indole served as the nucleophile. In this study, *N*-protected bicyclic alkenes were subjected to ARO conditions in the presence of a C_2_-ferrophosphine ligand and a halogen salt as an additive over a period of 2 days (Scheme 2).

**Scheme 2:**
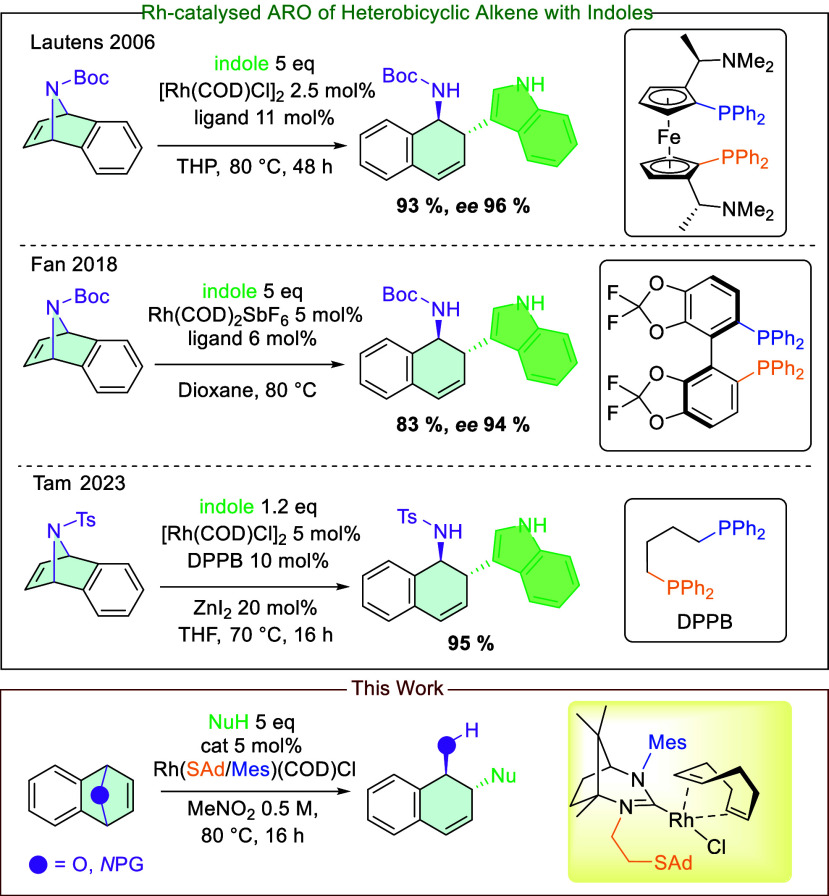
Examples of Asymmetric Ring-Opening Reactions of *N*-Protected Cyclic Alkenes

The results indicated that the catalytic system operates slowly. It is noteworthy that the published report included only a single example involving indole as the nucleophile. An additional example was described by Fan,^18^ who applied the methodology with a Rh­(COD)_2_SbF_6_ catalyst in conjunction with a chiral Segphos derivative. In their study, only a single instance of ARO with indole was reported. Different examples utilize various metal variants with in situ combination with enantiomerically pure ligands based on phosphines that provide high enantiomeric ratios (e.r.). The most frequently utilized metal catalysts in the synthesis processes include Rh,^18–21^ Ir,^22–26^ Pd,^11,27–31^ Ni,^32^ and Cu.^33–36^

In addition to enantiopure phosphine ligands, carbene-based complexes have been recently applied in the ARO. In 2020, Dora^37^ conducted an investigation into iridium-based catalytic systems incorporating chiral *N*-heterocyclic carbene (NHC) ligands. Their study focused on the application of these catalytic systems in the asymmetric ring-opening of oxabenzonorbornene derivatives, achieving high enantioselectivities in product formation. However, the substrate scope was relatively limited, which constrains the generality of the methodology. Furthermore, the catalytic system relied on the use of expensive iridium diastereomeric complexes, which could pose challenges for practical and scalable applications. Subsequently, Yoshida and Seki^38^ proposed an alternative NHC-ligand system for Rh­(I) complexes for the same transformation. In this case, the substrate scope remains limited to a few select products and enantioselectivities did not exceed 91:9 e.r. The reaction requires the addition of sodium iodide as an additive to enhance the catalytic activity. Although this system affords products in good yields, it does not exhibit fully catalytic behavior since sodium iodide was added in excess of the Rh­(I) catalyst.

In this study, we aim to develop an asymmetric ring-opening reaction utilizing a bench-stable rhodium *N*-heterocyclic carbene catalyst that operates without the need for additional additives. This approach offers an economical, efficient, enantioselective, and broadly applicable methodology. Therefore, new enantiopure sulfur-functionalized rhodium-NHC complexes were prepared and investigated in a diverse array of substrates.

In our group, we have developed a straightforward methodology for the synthesis of chiral *N*-heterocyclic carbenes applied as organocatalysts^39^ or ligands^40–42^ derived from camphoric acid from the chiral pool. Reported analogue complexes^43,44^ have not been applied successfully in asymmetric catalysis so far.^45^ Building upon this foundation, our current work aspired to synthesize a novel class of ligands featuring an additional coordination site via a functionalized side of the NHC scaffold. This approach was inspired by recent literature.^46–53^ Our objective was to prepare enantiomerically pure ligands and corresponding Rh­(I) catalysts incorporating sulfur functionalities to explore their reactivity and enantioselectivity in the asymmetric ring-opening (ARO) of *N*-protected bicyclic alkenes. This investigation endeavors to expand the scope of products accessible through this transformation and elucidate the influence of the sulfur-functionalized NHC ligands on catalytic performance.

Our investigation commenced with the synthesis of camphor-based ligand precursors, which is described in the Supporting Information (SI, Scheme S2, p S14).

The camphor-based ligand precursors (**4**) were transformed into Rh­(I) catalysts (**Rh5**) via a straightforward method (Scheme 3) employing KHMDS as a base to generate the corresponding carbene intermediates. These carbene species subsequently reacted with [Rh­(COD)­Cl]_2_ to afford the desired catalysts. Among the Rh­(I) catalysts synthesized, one group incorporated a thio-adamantane (**Rh5a**–**c**) or a thio-phenyl (**Rh5d**, **Rh5e**) substituent, while another was coordinated with a chiral SIMes ligand (**Rh5f**) for comparison.

**Scheme 3:**
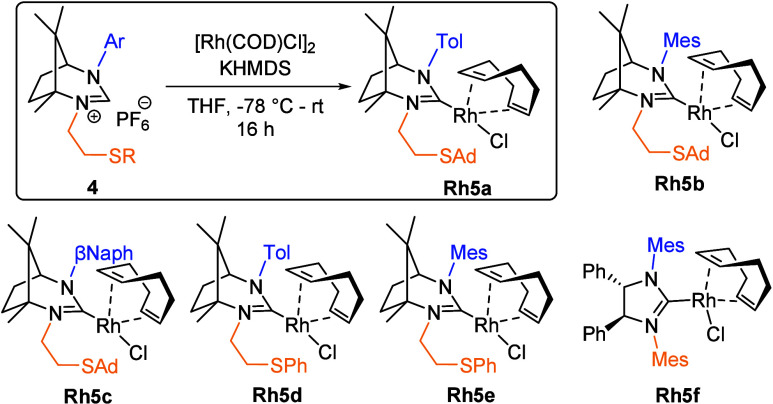
Preparation of Sulfur-Functionalized Enantiopure Rh­(I) Complexes

In the subsequent step, we evaluated the reactivity and enantioselectivity of the synthesized catalysts for the optimization (SI, Tabel S1, p S4) using *N*-(4-bromobenzenesulfonyl)-protected azabenzonorbornene (6*N*-BsBr) as the substrate in conjunction with *N*-methylindole. *N*-Methylindole substrate proved to be challenging in previous studies involving Ir-based catalysts, as reported in the initial publication.^54^ Therefore, we undertook a comprehensive investigation to confirm that our catalysts could effectively facilitate this transformation. Additionally, we screened various *N*-protected heterobicyclic alkenes for this reaction, and *N*-SO_2_C_6_H_4_Br emerged as the most promising protecting group based on our results (SI, Table S2, p S6). In Table S1 is shown that catalysts bearing SPh substituents yielded the lowest product formation, with enantioselectivities of 59:41 e.r. (**Rh5a**) and 50:50 e.r. (**Rh5d**).

More sterically hindered catalysts, such as **Rh5c**, **Rh5e**, and **Rh5f**, afforded comparable yields; however, only **Rh5c** and **Rh5e** achieved moderate enantioselectivities of **64:36** e.r. (entry 3) and **67:33** e.r. (entry 5), respectively. Additionally, a chiral **Rh­(I)** catalyst derived from the literature-reported chiral ligand (**Rh5f**) provided moderate yields but negligible enantioselectivity. Based on the optimized reaction conditions, we conclude that the presence of a 2,4,6-mesyl group in conjunction with an adamantane thioether on the ligand significantly enhances both reactivity and enantioselectivity (entry 2). Further optimization of reaction parameters gave **Rh5b** in nitromethane with a yield of 98% and an e.r. of 92:8 (SI, Table S1, p S4).

With the optimized reaction conditions, the substrate scope of the catalytic reaction (Scheme 4) was explored. Initially, simple methyl derivatives of indoles (**7a**–**7d**) were employed as substrates. Notably, all desired products were successfully synthesized, including the 3-methylindole derivative, which is less common due to the typical higher reactivity of the C-3 position in indoles. The synthesis of product **7d** represents a novel contribution to the literature, as it involves substitution at the C-2 position, yielding a moderate overall yield and high enantiomeric ratio. Since the C-2 position is less reactive than a C-3 position in an indole, **7d** resulted in a lower yield. Furthermore, we evaluated a series of 5-substituted indoles (**7e**–**7h**) in the asymmetric ring-opening reaction. These substrates exhibited broad functional group tolerance, affording products in good to very good yields with high enantioselectivity; however, **7g**, with a boronic acid group, gave just a moderate yield. Substituted indoles bearing additional substituents (**7i**–**7l**, **7p**) were also examined, resulting in the synthesis of a range of products with very high e.r. values (up to 95:5 for **7p**) and yields (up to 99% for **7j**). However, **7i**, with a relatively sterically demanding OAc group at the C-4 position next to the C-3 position, gave a lower yield. In addition, reactions involving pyrroles (**7q**, **7r**) were performed, producing good yields with satisfactory enantioselectivities. Reactions with *N*-methyl aniline as a nucleophile were also conducted, leading to the formation of *N*-SO_2_C_6_H_4_Br, *N*-Boc-protected heterobicyclic products **7s** and **7t**, respectively, with good yields and high e.r. Moreover, substrates bearing different *N*-protecting groups such as Ts, *N*-SO_2_C_6_H_4_Cl, and *N*-SO_2_C_6_H_4_F (**7m**–**7o**) were explored, resulting in good yields but moderate enantioselectivities. To assess the catalytic system’s versatility, we investigated the reactivity toward *O*-heterobicyclic alkenes, which are relevant for comparison with known ring-opening reactions of oxabenzonorbornenes. Using standard substrates such as *N*-methylindole (**7u**) and *N*-methyl anilines (**7v**–**7x**), the influence of electron-withdrawing and electron-donating groups on the reactivity and enantioselectivity was evaluated. Notably, extraordinary product **7y** was synthesized using our catalyst, despite the lower inherent reactivity of this substrate class relative to heterocyclic benzononorbornenes. All products obtained from *O*-heterobicyclic alkenes were isolated in yields ranging from 55% to 96%, with moderate enantioselectivities. This comprehensive substrate scope demonstrates the robustness and versatility of the presented catalytic system across various heteroaromatic and heterobicyclic substrates, highlighting its potential applicability in asymmetric synthesis.

**Scheme 4:**
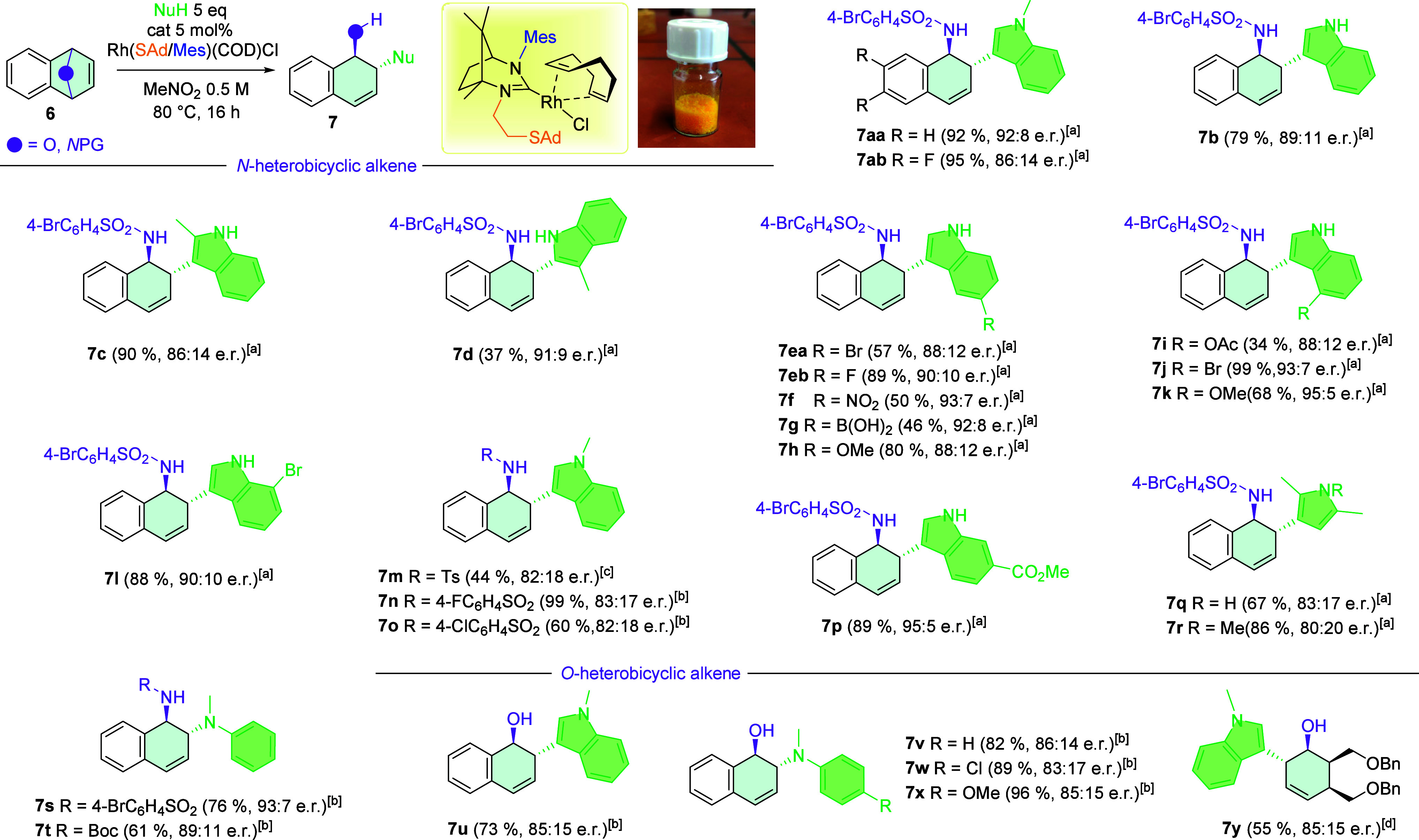
Extended Scope of the Rh-Catalyzed ARO Reaction of Heterocyclic Alkene^a^

a Isolated yields and e.r. determined by chiral HPLC. ^[a]^Standard conditions: **6** (0.112 mmol, 1 equiv), NuH (0.56 mmol, 5 equiv), **Rh5b** (5 mol %) in MeNO_2_ at 80 °C for 16 h. ^[b]^THF as solvent. ^[c]^THF as solvent and ZnI_2_ (20 mol %). ^[d]^Indole (10 equiv), no solvent 110 °C for 3 d.

In this study, we evaluated the synthetic utility of compound **7j** (Scheme 5) by demonstrating its applicability in various catalytic transformations. First, the reactivity of the carbon–carbon double bond in oxidative and reductive conditions was assessed. Under oxidative conditions using OsO_4_, the product **8c** was obtained with a 41% yield, whereas reductive conditions using H_2_ (15 bar) and Pd/C yielded the corresponding product **8a** with a 20% yield. In this reaction, a reduction process occurs concomitantly with halogen exchange, resulting in the substitution of the halogen atom with a hydrogen atom. Following these initial transformations, we explored reactions involving the aromatic bromide functionalities present in compound **7j**. Specifically, we employed magnesium to dehalogenate the aromatic bromide, resulting in compound **8b** in good yield and enantioselectivity. Lastly, a Suzuki–Miyaura cross-coupling reaction was performed using a palladium catalyst, leading to the formation of compound **8d** with a moderate yield and enantiomeric ratio.

**Scheme 5:**
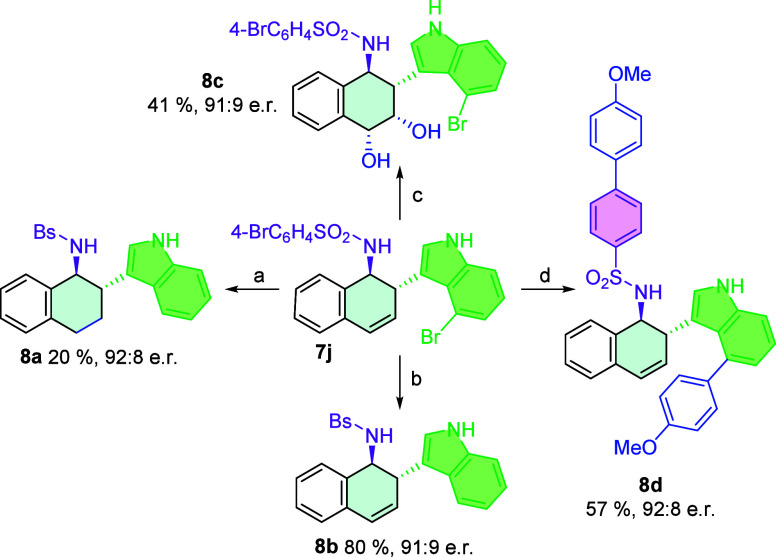
Representative Transformations of Products **7j** ^a^

a Conditions: a) Pd/C (10 mol %), H_2_ (15 bar), MeOH, rt, 16 h, b) Mg (5 equiv) (BrCH_2_)_2_ (cat.), MeOH, rt, 16 h, c) NMO (5 equiv), OsO_4_ (10 mol %), *t*BuOH/THF/H_2_O, rt,16 h, d) Pd­(Ph_3_)_4_ (10 mol %), 4-methoxyphenylboronic acid (2.5 equiv), Na_2_CO_3_ (6 equiv), Tol/H_2_O, 110 °C, 2 d.

Building upon previously reported Ir/Rh-catalyzed^55,56^ ring-opening reactions of oxabicyclic alkenes with indole nucleophiles, as well as recent studies on Rh/Ir-NHC complexes^37,46^ featuring hemilabile ligands, we propose a similar mechanism, which is described in the Supporting Information (SI, Scheme S1, p S8). To support the proposed mechanistic hypothesis, we conducted DFT calculations of the diastereomeric intermediates of the reaction (Figure 1). Details are presented in the Supporting Information (SI, p S9).

**Figure 1:**
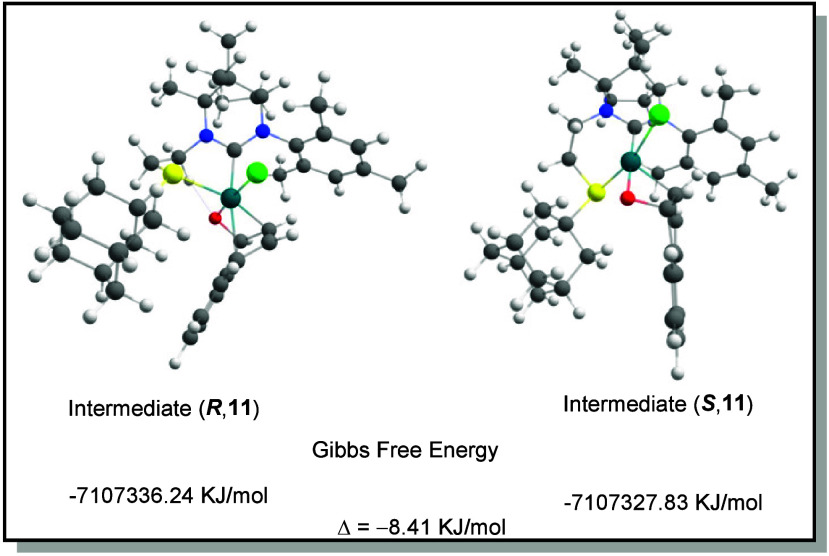
Gibbs free energies of the diastereomeric intermediates (*R* and *S* configurations) associated with the transition states **11** were computed.

As can be seen, the free energy of the intermediate with the observed stereocenter in the product is around 8.4 kJ/mol lower. This energy difference would correlate to an e.r. of ca. 97:3 at 20 °C as calculated in the literature.^57^ However, in the present case, the reaction had to be performed at 80 °C, which can be calculated to an e.r. of 94.5:5.5 via a simplified Eyring equation (see SI, p S11). This correlates very close to the best observed e.r.

In this study, we report the synthesis of novel Rh-NHC complexes based on chiral natural product-derived camphor ligands functionalized with hemilabile thioether groups. The synthesized complexes were thoroughly characterized by using multinuclear NMR spectroscopy and high-resolution mass spectrometry. Notably, the most active complex, **Rh5b**, was obtained as a single atropisomer. The developed methodology enables the efficient catalytic ring-opening of *N*-protected azabicyclic and oxabicyclic alkenes, achieving high isolated yields (up to 99%) and excellent enantioselectivities (up to 95:5 e.r.), alongside broad functional group tolerance. The catalysts exhibit high regio- and diastereoselectivity, with no formation of 1,4-ring-opening or *syn*-products observed under any reaction conditions. Furthermore, DFT calculations provided insights into the hemilabile behavior of the thioether functional group within the chiral ligand framework. Comparative analysis with literature reports on sulfur-functionalized NHC ligands suggests a proposed mechanism that explains the activation of Rh complexes in the absence of additional halogen salts for the asymmetric ring-opening reactions. Finally, a crude cost estimation of ligand **4b** with one of the best performing commercial ligands, Difluorphos, in an AOR^18^ showed that our ligand is around 88 times cheaper (for details see SI, p S2), which shows a benefit of a chiral pool derived camphor-based ligand.

## Supplementary Material



## Data Availability

The data underlying this study are available in the published article and its Supporting Information.

## Notes

Experimental, mechanistic, and DFT calculation details as well as NMR spectra and data (PDF)
