# Supplementary material for: Camphor-Based NHC Ligands with a Sulfur Ligator Atom in Rhodium Catalysis: Catalytic Advances in the Asymmetric Ring Opening of N‑Protected Azabenzonorbornenes
Source: Org Lett. 2025 Jul 25;27(31):8417–22. doi: 10.1021/acs.orglett.5c02110 (PMC12340959; doi:10.1021/acs.orglett.5c02110)

Supporting Information

Camphor based NHC Ligands with a sulfur ligator-atom in Rhodium Catalysis: Catalytic Advances in the Asymmetric Ring Opening of *N*-Protected Azabenzonorbornenes

Daniel Kamzol, Mohsen Bahramiveleshkolaei, René Wilhelm*

Institute of Organic Chemistry, Leibnizstr. 6, 38678, Clausthal-Zellerfeld, Clausthal University of Technology, Germany.

Clausthal-Zellerfeld, Germany. E-mail: rene.wilhelm@tu-clausthal.de

Table of content

1. General remarks 2

2. Optimization 4

2.1 Screening of **Rh5** catalysts to identify the optimal catalyst for the targeted reaction. 4

2.2 Optimalization for Ring Opening of Azabenzonorbornenes 5

4. DFT Calculation 9

5. Experimental procedures and characterization data 12

5.1. General Procedures 12

5.1.1 General procedure for preparation of thioether bromides (**G.P. A**) 12

5.1.2 General procedure for preparation of camphor ligands (**G.P. B**) 14

5.1.3 General procedures for preparation of camphor catalysts (**G.P.C**) 20

5.1.4 Synthesis of starting materials for Asymmetric Ring Opening reaction (**G. P. D**) 24

5.2 Scope of products for ARO reaction (**G. P. E)** 29

5.3 Prepared modifications for product **7j**. 48

5.3.1 Scale up for synthesis with **7j**. 48

5.3.2 Reduction of **7j**. 48

5.3.3 Debromination of **7j**. 49

5.3.4 Oxydation of **7j**. 50

5.3.5 Suzuki coupling of **7j**. 51

6. References 52

7. Copy of NMR data Analysis 54

1. General remarks

Unless otherwise noted, materials were purchased from commercial suppliers and used without further purification. ^{1}H and ^{13}C NMR were recorded in $CDCl_3$ solvent on a Bruker AV-400 MHz spectrometer and chemical shifts are reported in ppm. Data are reported in the following way: chemical shift, multiplicity (s = single, d = doublet, t =triplet, q = quartet, br = broad, m = multiplet), coupling constants (Hz) and integration. Assignments are based on HSQC and HMBC spectra. Spectra were calibrated using the corresponding non-deuterated solvent signal. ^{19}F NMR (376 MHz) chemical shifts are given in ppm. Primary NMR data files were processed by MestReNova. Mass spectra were recorded on Bruker Impact2 apparatus with TOF analyzer. The optical rotations were recorded on Digital polarimeter P3000 Series, in a thermostated (20 °C) 1 dm long cell with high-pressure sodium lamp and are reported as follow: $[\alpha]_D{}^T$ [solvent, c (g/100 mL)]. HPLC analysis was performed on a Knauer Azura apparatus using Chiralpak AS-H, Dr. Maisch OD-H and AD-H equivalent columns with isopropyl alcohol and hexane as eluents. Flash-column chromatography was performed over silica gel (200–300 mesh). Thin-layer chromatography (TLC) was performed using silicagel F254 TLC plates and visualization of the developed chromatogram was performed under ultraviolet light (254 nm) and on staining by immersion in aqueous, acidic ceric ammonium molybdate followed by charring at 150 °C. All products were purified by column chromatography using silica gel (Merck 60–120 mesh). The spectra and other data were consistent with the reported values. Unless otherwise stated, all reagents were purchased from commercial suppliers and used as received. All reactions to be performed under an inert atmosphere of argon were achieved using Glovebox. Toluene (puriss. p.a., ACS reagent, ≥ 99.7% (GC)) and THF (99.9% GC) with 2,6-di-tert-butyl-4-methylphenol (250 mg/L) as stabilizer were purified by passage through a column containing activated alumina under nitrogen pressure (Dry Solvent Station GT S100, GlassTechnology, Geneva, CH). (+)-(1*R*,3*S*)-camphoric acid (95-98%), 1-Adamantanethiol (95-98%) were purchased from BLD, 2-Bromotoluene (95-98%), 2-Bromonaphthalene (95-98%), Mesityl Bromide 99% were purchased from TCI Europe, Thiophenol 99% From Sigma-Aldrich, 2-Chloroethanol 99% from abcr. All the racemic compounds of the ARO reactions were synthesized in the same manner as the enantioenriched products by replacing the catalysts **Rh5b** with $[Rh(COD)Cl]_2$, DPPB and NaI as an additive as well as with Rh(SIMes) complex with NaI as additive.

In order to compare the cost of our ligand **4b** with one of the best performing commercially available ligands, Difluorphos, in the AOR, we show here a short price estimation. All unitary prices for each chemical used in the synthesis of ligand **4b** were calculated by dividing the listed prices of the largest available unit on Fluorochem (https://dougdiscovery.com) by the amount of each compound required. The unitary price of standard Difluorphos is 567 €/g, (387030.72 €/mol) while our designed **4b** proved to be ≈ 88 times cheaper based on €/mol. However, the costs of the solvent, silica gel for column chromatography, H_2SO_4, AcOH and utilities are not included in this esteem since their cost can be considered negligible compared to the cost of applied substrates. Also working hours are not included and hence, this cost comparison is for an academic environment.

Compound	Unitary price	Amount Used	Cost
1-Adamantanethiol	3.26 €/g	3 g	9.78 €
NaH	0.17 €/g	0.855 g	0.14 €
2-chloroethanol	0.72 €/ml	1.32 ml	0.95 €
	Total for 3.8 g of **1a**		10.87 €
	Unitary cost of **1a**		2.86 €
**1a**	2.86 €/g	3.80 g	10.87 €
CBr_4	0.40 €/g	8.06 g	3.22 €
PPh_3	0.14 €/g	6.38 g	0.89 €
	Total for 5.2 g of **1c**		14.98 €
	Unitary cost of **1c**		2.88 €
(+)-camphoric acid	0.53 €/g	15 g	7.95 €
NaN_3	0.40 €/g	14.61 g	5.84 €
	Total for 9 g of **2**		13.79 €
	Unitary cost of **2**		1.53 €
**2**	1.53 €/g	1.5 g	2.30 €
$Pd_2(dba)_3$	13.20 €/g	0.48 g	6.34 €
(+/-)-BINAP	1.73 €/g	0.66 g	1.14 €
NaO*t*Bu	0.2 €/g	3.04 g	0.61 €
MesBr	0.10 €/g	2.48 g	0.25 €
	Total for 2.6 g of **3b**		10.64 €
	Unitary cost of **3b**		4.09 €
**3b**	4.09 €/g	1.2 g	4.91 €
$CHC(OMe)_3$	0.12 €/mL	2.31 mL	0.28 €
**1c**	2.88 €/g	4.4 g	12.67 €
$NaPF_6$	0.49 €/g	1.77 g	0.87 €
	Total for 2.6 g of **4b**		18.73 €
	Unitary cost of **4b** /g		7.20 €
	Unitary cost of **4b** / mol		4397.26 €
	Unitary cost of Difluorphos / mol		387030.72 €

2. Optimization

2.1 Screening of **Rh5** catalysts to identify the optimal catalyst for the targeted reaction.

In Table S1, all initial screening reactions were conducted using ZnI_2 as an additive to identify the most effective catalyst. This choice was based on previous studies in the literature[1] from Tam demonstrating that the addition of halogen salt facilitates the reaction by promoting product formation. Subsequently, we investigated the effect of excluding the halogen salts (Entries 6 – 8). Our findings indicate that in the absence of any additional salts, **Rh5b** can effectively catalyze the reaction, achieving high yields without compromising enantioselectivity (Entry 8, 90% yields, 85:15 e.r.).

Table S1. *Evaluation of Camphor-Based NHC-**Rh5a-f** Catalysts in the ARO of N-Protected Heterobicyclic Alkenes.*

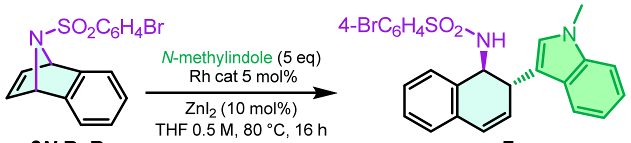

Entry	Rh cat	Conv. (Yield) [%][a]	e.r.[b]
1	**Rh5a**	100 (18)	59:41
2	**Rh5b**	100 (73)	86:14
3	**Rh5c**	100 (36)	64:36
4	**Rh5d**	100 (40)	50:50
5	**Rh5e**	100 (66)	67:33
6	**Rh5f**	100 (70)	51:49
7	**Rh5g**	20(18)	51:49
8[c]	**Rh5b**	100 (99)	86:14
9[d]	**Rh5b**	100 (90)	85:15
10[d]	**Rh5b** Dioxane	100 (74)	79:21
11[d]	**Rh5b** ACN	100 (88)	85:15
12[d]	**Rh5b** MeTHF	100 (68)	62:38
13[d]	**Rh5b** DME	100 (80)	85:15
14[d]	**Rh5b** Acetone	100 (94)	91:9
15[d]	**Rh5b** $MeNO_2$	100 (98)	92:8
16[d]	**Rh5b** (2.5 mol%)	58 (50)	84:16
17[d]	**Rh5b** no cat	0 (0)	-

All reactions were conducted under standard conditions: **6*N*-PG** (0.056 mmol, 1eq.), ***N*-methylindole** (0,28 mmol, 5eq.), **Rh5b** (2 mg, 5 mol%), **ZnI_2** (0.0112 mmol, 20 mol%). [a] Conversions and yields were determined by ^1H NMR spectroscopy using CH_2Br_2 as internal standard. [b] Determined by chiral HPLC OD-H column. [c] addition of 5 mol% ZnI_2. [d] no addition of ZnI_2.

Based on the data presented in Table S1, S4, the following conclusions can be drawn from the study. Rhodium complexes such as **Rh5a** and **Rh5d** predominantly produce almost racemic mixtures of the asymmetric reaction products. This observation is attributed to the presence of a tolyl group on one side of the camforic-NHC ligand in these catalysts. The tolyl group is freely rotatable at its position and does not impose significant steric hindrance on the catalyst's active site, resulting in reduced enantioselectivity.

In contrast, the **Rh5c** complex, which bears a β-naphthyl substituent, exhibits increased steric hindrance relative to the tolyl group, leading to higher enantioselectivity (64:36). The most effective catalyst identified in this study is **Rh5b**, featuring a mesityl substituent that provides substantial steric hindrance on the catalyst's active side, thereby enhancing enantioselectivity (86:14). An analog of **Rh5b** substituted with a phenyl group (SPh) **Rh5e** demonstrated a decrease in enantioselectivity (67:33), indicating that steric hindrance introduced via the sulfur atom substituent plays also a significant role in influencing the spatial environment around the Rh center during catalysis.

To conclude our investigation into the preparation and application of sulfur-modified camphor-based NHC Rh(I) complexes, we synthesized the complex **Rh5f**. This complex exhibits similar bulky (Mes) substituents on one side and a comparable bulky substituent at the sulfur-modified position. As detailed in Table S1, this particular catalyst demonstrates the lowest activity among all the evaluated catalysts and predominantly yields a racemic mixture of products. These findings support our hypothesis regarding the hemilabile behavior of the sulfur-modified ligands. This is further supported by the DFT calculation of two diastereomeric intermediates on a high level (see Section 4 of the SI). The calculated values are very good in compliance with observed e.r. level and show that a coordination of the Rh with the sulfur-atom are the most stable intermediates. For the starting complex a Rh-S coordination is not important, however for the formation of the intermediates it shows a stabilizing influence.

Furthermore, we examined the impact of solvents and temperature on reaction yield and enantiomeric ratio. Replacing THF with other polar solvents (Entries 9 – 14), we determined that nitromethane ($MeNO_2$) provides the optimal solvent environment for the reaction (Entry 14, 98% yield, 92:8 e.r.). Regarding temperature effects, variations from 80 °C generally resulted in lower yields and e.r. compared to the optimal temperature of 80 °C (see Figure 1, S6). Finally, we evaluated the effect of catalyst loading by reducing the catalyst amount to 2.5 mol% (Entry 15, 50% yield, 84:16 e.r.). This resulted in decreased reactivity and enantioselectivity. The reaction conducted in the absence of a catalyst did not yield product formation. Consistent with our current findings, we determine that the optimal reaction conditions involve the use of **Rh5b** catalyst at a loading of 5 mol%, performing the reaction in $MeNO_2$ at 80 °C for 16 h. Under these conditions, employing 5 equivalents of the indole derivative yields the highest conversion and enantioselectivity in the asymmetric ring-opening process.

2.2 Optimalization for Ring Opening of Azabenzonorbornenes

Knowing that **Rh5b** is the most active catalyst, we decided to search for proper azabenzononbornene as a starting material. Keeping in mind the work of Tam et al., we decided to test our catalytic reaction in the presence of, and without ZnI_2. The first step to optimize the reaction was to choose a suitable protecting group for azabenzononbornene.

Table S2. *Screening of azabenzonorbornenes as starting materials for asymmetric ring-opening reactions.*

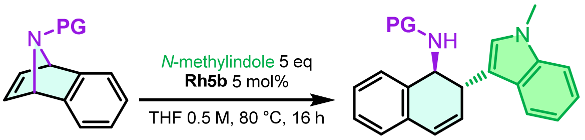

PG = Boc, 4-$BrC_6H_4SO_2$, Ts, 4-$FC_6H_4SO_2$, 4-$ClC_6H_4SO_2$, *p*Ns, Ns

PG	ZnI_2 (20 mol%)	Conv. (Yield) [%][a]	e.r.[b]
Boc	yes	-	-
Boc	no	-	-
4-$BrC_6H_4SO_2$	yes	100 (73)	86:14
4-$BrC_6H_4SO_2$	no	100 (90)	85:15
Ts	yes	70 (60)	82:18
Ts	no	50 (0)	-
4-$FC_6H_4SO_2$	yes	100 (28)	66:34
4-$FC_6H_4SO_2$	no	100 (87)	83:17
4-$ClC_6H_4SO_2$	yes	100 (99)	83:17
4-$ClC_6H_4SO_2$	no	100 (70)	82:18
*p*Ns	yes	-	-
*p*Ns	no	-	-
Ns	yes	-	-
Ns	no	-	-

All reactions were conducted under standard conditions: **6*N*-PG** (0.056 mmol, 1eq.), ***N*-methylindole** (0,28 mmol, 5eq.), **Rh5b** (2 mg, 5 mol%), **ZnI_2** (0.0112 mmol, 20 mol%). [a] Conversions and yields were determined by ^1H NMR spectroscopy using CH_2Br_2 as internal standard. [b] Determined by chiral HPLC OD-H column.

This study clearly demonstrates that the presence of ZnI_2 is crucial for the tosyl (Ts) protecting group. In our study, we investigated the interaction of ZnI_2 with various protecting groups on bicyclic amines to elucidate the underlying mechanism governing their reactivity. Previous work by Tam et al. suggested that ZnI_2 interacts with the tosyl (Ts) group, likely reducing the electron density on the nitrogen atom and thereby increasing the reactivity of the protected amine during oxidative addition. Consistent with this, we observed similar reactivity for the Ts protecting group in our experiments. To further understand the influence of the protecting group's electronic properties, we examined alternative protecting groups. Starting with a simple Boc group, we observed that the reaction was unsuccessful, which we attribute to the Boc group's steric hindrance and its lack of electron-withdrawing character. In contrast, when the Ts group was employed in the presence of ZnI_2, the reaction proceeded efficiently, providing the product with an e.r. of 82:18, indicating that ZnI_2 likely interacts with the sulfonyl group, decreasing the electron density on the nitrogen and facilitating oxidative addition.

To systematically assess the role of electron-withdrawing capacity, we substituted the Ts group with various *para*-halogenated benzenesulfonyl derivatives. Among these, benzenesulfonyl bromide (4-$BrC_6H_4SO_2$) demonstrated the highest reactivity, achieving an e.r. of 85:15 and a 90% yield in the absence of ZnI_2. Bromide substitution proved optimal, with chlorine (4-$ClC_6H_4SO_2$) providing similar results, whereas the fluorinated derivative (4-$FC_6H_4SO_2$), which possesses the strongest electron-withdrawing effect among halogens, showed good reactivity without additives but exhibited a significant reduction in yield and enantioselectivity (down to 66:34) upon addition of ZnI_2.

These findings suggest that an overly strong electron-withdrawing effect may be detrimental to the reaction's efficiency and selectivity. To further validate this hypothesis, we synthesized additional sulfonamide derivatives bearing strongly electron-withdrawing groups, such as *p*Ns and Ns groups, which contain the nitro (NO_2) substituent. Reactions with these derivatives failed entirely, supporting the notion that excessive electron withdrawal impairs the reaction pathway.

In summary, we have developed a new class of protecting groups based on slight electron-withdrawing substituents that facilitate asymmetric ring-opening reactions of bicyclic amines without the need for additional reagents. These protecting groups inherently modulate the electron density at the nitrogen atom, promoting oxidative addition and enabling efficient enantioselective transformations. The next step was to investigate suitable temperatures Figure 1, p. S6 for the reaction.

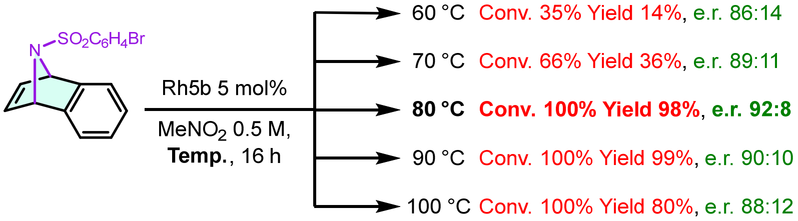

Figure S1. Screening of temperatures for *N*-Bosbenzonorbornene.

Based on the results presented in Figure S1, it is evident that the optimal temperature for the reaction is 80 °C. A decrease in reaction temperature significantly impacts the reaction yield, likely due to the inability of the catalyst to effectively remove the COD ligand and initiate interactions with substrate **6a** at lower temperatures. Additionally, enantioselectivity exhibits a slight decrease from a high level to 86:14 e.r. at 60 °C. This decrease can be expected in cases where the reaction yields are decreasing significantly. Increasing the reaction temperature maintains high reactivity; however, the yield decreases, most probably due to a reduced rate of product decomposition. Despite this decline in yield, enantioselectivity remains high, reaching 88:12 e.r. at 100 °C. These observations suggest that the **Rh5b** catalyst is relative thermally stable. Generally, increasing temperature has an effect on enantioselectivity, however as will be discussed in Section 4 of the SI, this effect is relatively small in the explored temperature ranges. The final verification step involved determining the stereochemistry of the desired product obtained from the ring-opening reaction. In this reaction, two stereoisomeric pathways are possible: *syn* and *anti* ring opening. To ascertain the stereochemical configuration, we compared the experimental spectroscopic data of our product with literature reported spectra for known *N*-Tosylated products, specifically compounds **7m**, **7u**, and **7v**.[1–4] Our analysis indicates that the experimental spectra closely match the reported spectra for the *anti*-stereoisomer, suggesting that the product possesses *anti* stereochemistry. Additionally, spectra for other stereoisomers have been documented in the literature, exhibiting distinct spectral features, which further supports our stereochemical assignment. Furthermore, the optical rotations of **7m**, **7u** and **7v** were compared with the literature in order to assign the absolute stereochemistry.

3. Proposed Mechanism

Scheme S1 shows the proposed mechanism, which is similar to a previously reported Ir/Rh-catalyzed[1,3] ring-opening reactions of oxabicyclic alkenes with indole nucleophiles, as well as recent studies on Ir/Rh-NHC complexes[2,5] featuring hemilabile ligands. Initially, during the reaction, the catalyst interacts with the substrate by binding to the *exo*-face of the azabicyclic alkene. Besides, the hemilabile SAd moiety coordinates to the metal center, enhancing the stability of the resulting complex intermediate **10**. Subsequently, the rhodium species undergoes oxidative insertion between the C-N bond, leading to the formation of intermediate **11**. The next step involves nucleophilic attack by the indole at the *C3* position via the *endo*-face, resulting in intermediate **12**, which is further transformed into intermediate **13**. Finally, reductive elimination coupled with proton transfer, restoring aromaticity in the indole, yields the final product **7a** and regenerates the active rhodium catalyst, thus completing the catalytic cycle.

Scheme S1. *Proposed mechanism for the* ***Rh5b*** *ring-opening reaction between* ***6N-BsBr*** *and N-methylindole.*

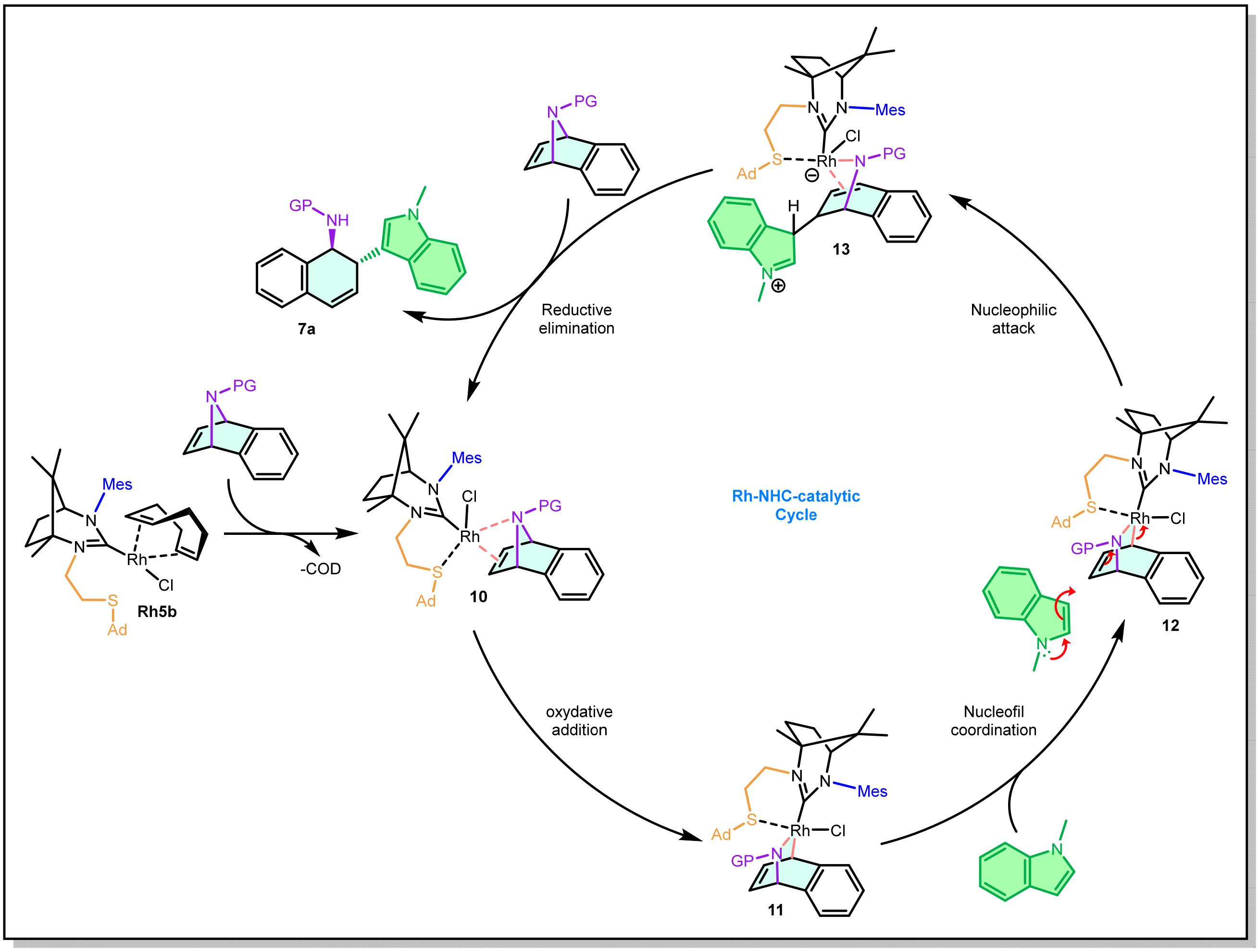

4. DFT Calculation

The DFT computations allowed us to investigate the enantioselectivity of the product and to elucidate the hemilabile behavior of the SAd arm within our catalyst system.

DFT calculations were performed for the first postulated intermediate **11**. The calculation of the diastereomeric intermediates give similar insights as the diastereomeric transition-states but are easier to optimize and calculate, hence reducing the possible error range. Each diastereomeric intermediate was initially optimized with XTB.[6,7] After that, we used the Conformer-Rotamer Ensemble Sampling Tool (CREST), which was developed by Grimme et al.[7,8] Afterwards, the conformer set was further processed using CENSO, developed by the same group,[9,10] to predict the most important conformer according to the Boltzmann distribution. In all calculations, THF was incorporated as a solvent. The conformer with the highest Boltzmann weight was further calculated with high-level DFT with ORCA.[11,12] The structure was optimized using the functional PBE0 with a dispersion correction[13,14] and the def2-TZVP basis set[15] with a PCM solvent model for THF. Frequency calculations were free from negative values.

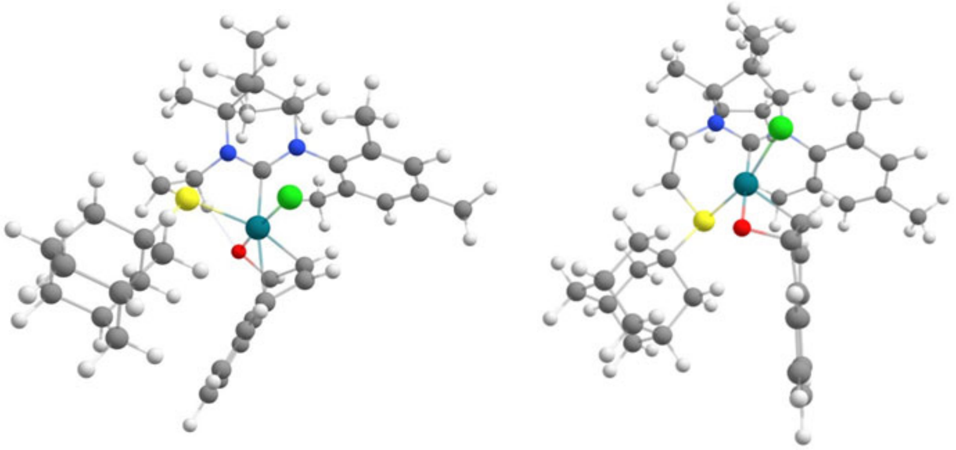

Intermediate (***R***,**11**) Intermediate (***S***,**11**)

Gibbs Free Energy

-7107336.24 KJ/mol -7107327.83 KJ/mol

Δ = –8.41 KJ/mol

X,Y,Z Coordinates for **S,11**

6	-3.798707000	1.536880000	0.195960000	6	-4.147826000	-2.972425000	0.471041000
6	-4.418108000	1.622493000	1.588062000	6	-4.569083000	-3.257458000	-0.819648000
6	-3.411772000	2.471801000	2.392589000	6	-4.386341000	-2.281593000	-1.789605000
6	-2.339985000	2.883179000	1.369483000	6	-3.788344000	-1.059435000	-1.507139000
6	-3.099115000	2.881925000	0.022894000	6	-3.698956000	-0.035601000	-2.594326000
7	-1.334742000	1.780786000	1.281408000	6	-3.143091000	-1.555036000	2.237564000
7	-2.791630000	0.459898000	0.129597000	6	-5.179080000	-4.582419000	-1.159069000
6	-2.209047000	2.995164000	-1.205185000	16	1.218545000	1.497626000	-0.163087000
6	-4.141299000	3.997103000	-0.048811000	6	5.078783000	2.812590000	-0.726826000
6	-1.558085000	0.633522000	0.623464000	6	5.802420000	1.957779000	0.312359000
6	-0.148481000	1.864744000	2.123910000	6	5.243794000	0.536696000	0.281787000
6	1.087037000	2.370749000	1.411164000	6	3.749352000	0.570502000	0.608761000
45	0.064149000	-0.595088000	0.141057000	6	3.029817000	1.424716000	-0.424732000
17	-0.370641000	-0.319969000	-2.213278000	6	3.582835000	2.850803000	-0.403644000
6	-3.330428000	-0.819504000	-0.205854000	6	5.449151000	-0.060633000	-1.108809000
6	-3.538111000	-1.767355000	0.805103000	6	5.283216000	2.215285000	-2.116571000

6	3.230601000	0.831395000	-1.819176000	1	3.051017000	3.465086000	-1.137397000
6	4.723713000	0.794906000	-2.144115000	1	3.434211000	3.302967000	0.580719000
1	-5.391672000	2.111529000	1.530944000	1	5.066854000	-1.085264000	-1.137774000
1	-4.583042000	0.634952000	2.011524000	1	6.518301000	-0.104278000	-1.342329000
1	-2.956293000	1.915866000	3.213852000	1	6.349494000	2.203296000	-2.366106000
1	-3.887081000	3.353678000	2.825401000	1	4.778228000	2.832118000	-2.867796000
1	-1.500442000	2.175164000	-1.309243000	1	2.697361000	1.439675000	-2.556378000
1	-1.644678000	3.930088000	-1.172287000	1	2.803191000	-0.172916000	-1.853429000
1	-2.828130000	3.020440000	-2.104893000	1	4.855911000	0.363157000	-3.140933000
1	-3.660136000	4.953036000	-0.262076000	6	4.348165000	-3.587242000	1.914537000
1	-4.836321000	3.788067000	-0.865715000	6	3.721036000	-3.065207000	0.792487000
1	-4.722351000	4.110472000	0.866253000	6	3.627635000	-3.802910000	3.083046000
1	0.050352000	0.869752000	2.510546000	1	4.280561000	-2.926526000	-0.126468000
1	-0.371537000	2.508989000	2.972551000	1	4.116951000	-4.218825000	3.956565000
1	1.962592000	2.162262000	2.026420000	6	2.365199000	-2.744551000	0.825467000
1	1.048002000	3.439367000	1.203397000	6	2.277596000	-3.474066000	3.128156000
1	-4.291959000	-3.712699000	1.252619000	1	1.709950000	-3.617974000	4.042641000
1	-4.721353000	-2.470744000	-2.805178000	6	1.649678000	-2.937922000	2.016147000
1	-4.612676000	0.567251000	-2.634753000	1	5.402116000	-3.839255000	1.872446000
1	-2.850300000	0.625981000	-2.449532000	6	0.266746000	-2.367018000	2.041416000
1	-3.590769000	-0.524936000	-3.563321000	6	1.628475000	-2.311600000	-0.356911000
1	-3.980125000	-1.160212000	2.819346000	1	2.185800000	-2.198112000	-1.279272000
1	-2.866310000	-2.508767000	2.689671000	6	0.275080000	-2.673675000	-0.435796000
1	-2.297602000	-0.878470000	2.348470000	1	-0.202356000	-2.856646000	-1.389704000
1	-4.409533000	-5.290285000	-1.482994000	6	-0.484244000	-2.606764000	0.735487000
1	-5.682440000	-5.019664000	-0.294930000	1	-1.508104000	-2.945679000	0.738117000
1	-5.900327000	-4.490516000	-1.973226000	8	0.424065000	-0.967898000	2.085668000
1	5.467465000	3.835413000	-0.701393000	1	-0.296043000	-2.728569000	2.910999000
1	5.673724000	2.391195000	1.310200000	1	-4.532727000	1.338241000	-0.582996000
1	6.876328000	1.942476000	0.098473000	6	-1.687345000	4.215192000	1.676420000
1	5.752612000	-0.080525000	1.028926000	1	-1.240433000	4.247239000	2.669432000
1	3.602926000	0.976860000	1.613916000	1	-0.924992000	4.470238000	0.939344000
1	3.330695000	-0.436079000	0.600867000	1	-2.455663000	4.987646000	1.648689000

X,Y,Z Coordinates for **R,11**

6	-4.315784000	0.652437000	0.295299000	6	4.791708000	3.603128000	0.414531000
6	-4.453620000	1.317245000	1.659359000	6	4.511900000	2.978722000	1.780246000
6	-3.735936000	2.669602000	1.471626000	6	3.016307000	2.669482000	1.901844000
6	-3.391792000	2.749582000	-0.031992000	6	2.617863000	1.699737000	0.788417000
6	-4.447568000	1.819986000	-0.684471000	6	2.897471000	2.310102000	-0.580376000
7	-2.079249000	2.086450000	-0.278680000	6	5.316225000	1.689565000	1.928903000
7	-2.977690000	0.038974000	0.158213000	6	5.191182000	1.329687000	-0.538598000
6	-4.176559000	1.475937000	-2.138925000	6	3.415605000	0.408236000	0.944475000
6	-5.855514000	2.408441000	-0.597026000	6	4.908048000	0.713748000	0.829585000
6	-1.905075000	0.762204000	-0.170789000	1	-5.508157000	1.456611000	1.900467000
6	-0.862218000	2.857756000	-0.458301000	1	-4.025101000	0.709402000	2.452089000
6	0.025824000	2.837911000	0.775744000	1	-2.830996000	2.749379000	2.075272000
45	-0.070549000	0.161361000	-0.817204000	1	-4.379408000	3.504815000	1.753388000
17	-1.080391000	-0.423406000	-2.879657000	1	-4.166022000	2.388399000	-2.740599000
6	-2.876632000	-1.299986000	0.642403000	1	-4.979928000	0.841405000	-2.521173000
6	-2.385190000	-1.539307000	1.927499000	1	-3.234572000	0.954192000	-2.295294000
6	-2.380024000	-2.849781000	2.401777000	1	-5.996631000	3.161426000	-1.374357000
6	-2.840341000	-3.911709000	1.642345000	1	-6.591089000	1.618265000	-0.767150000
6	-3.340846000	-3.637275000	0.373130000	1	-6.077155000	2.873765000	0.363034000
6	-3.378836000	-2.349775000	-0.140339000	1	-1.116408000	3.888260000	-0.690167000
6	-3.940077000	-2.111785000	-1.505439000	1	-0.309573000	2.492644000	-1.337261000
6	-1.855447000	-0.465101000	2.830362000	1	-0.557810000	3.057748000	1.669102000
6	-2.778708000	-5.317042000	2.155747000	1	0.801267000	3.596477000	0.685827000
16	0.847771000	1.231165000	1.018808000	1	-1.989713000	-3.038498000	3.397730000
6	4.392641000	2.623811000	-0.686836000	1	-3.713411000	-4.452259000	-0.240039000

1	-3.263394000	-1.498518000	-2.102083000	6	5.135890000	-3.698758000	-0.383493000
1	-4.101676000	-3.061058000	-2.017084000	6	4.179303000	-3.448318000	0.587810000
1	-4.901228000	-1.591226000	-1.461638000	6	4.867257000	-3.389492000	-1.710639000
1	-0.861849000	-0.728847000	3.198849000	1	4.380001000	-3.691309000	1.626708000
1	-1.775133000	0.500790000	2.338688000	1	5.606949000	-3.590392000	-2.477609000
1	-2.499243000	-0.351723000	3.706805000	6	2.951078000	-2.879399000	0.248947000
1	-2.679372000	-5.338126000	3.242023000	6	3.644412000	-2.824966000	-2.053569000
1	-3.670954000	-5.881106000	1.876190000	1	3.431831000	-2.579198000	-3.089531000
1	-1.917183000	-5.843604000	1.733476000	6	2.686002000	-2.558338000	-1.088071000
1	4.587796000	3.068488000	-1.667660000	1	6.087564000	-4.138890000	-0.107575000
1	4.230310000	4.537965000	0.307412000	6	1.418945000	-1.835633000	-1.467539000
1	5.855298000	3.850272000	0.330199000	6	1.945773000	-2.606816000	1.258640000
1	4.786424000	3.682037000	2.572803000	1	2.194018000	-2.842771000	2.289384000
1	2.452734000	3.604349000	1.833807000	6	0.737370000	-2.110161000	0.956428000
1	2.798593000	2.221407000	2.876732000	1	0.010946000	-1.977997000	1.744606000
1	2.601016000	1.596006000	-1.350530000	6	0.327212000	-1.814992000	-0.410842000
1	2.321324000	3.230128000	-0.718694000	1	-0.617249000	-2.286973000	-0.680799000
1	5.133122000	1.244535000	2.913014000	8	1.689774000	-0.460874000	-1.652076000
1	6.387354000	1.908839000	1.863353000	1	1.023445000	-2.279087000	-2.393776000
1	6.261836000	1.535355000	-0.644484000	1	-5.048916000	-0.134714000	0.121206000
1	4.911128000	0.625503000	-1.328765000	6	-3.410799000	4.167203000	-0.557543000
1	3.109275000	-0.282862000	0.161160000	1	-4.424774000	4.556501000	-0.469279000
1	3.196292000	-0.050451000	1.913847000	1	-2.764530000	4.826497000	0.025017000
1	5.462952000	-0.223855000	0.934979000	1	-3.114312000	4.220475000	-1.607028000

For calculating the enantioselectivity of a reaction it is possible to apply a simplified Eyring equation as given below:

$$\boldsymbol{ee} \approx \frac{-\boldsymbol{e}^{-1\times\frac{\Delta\Delta G^{\ddagger}}{RT}} - \mathbf{1}}{-\boldsymbol{e}^{-1\times\frac{\Delta\Delta G^{\ddagger}}{RT}} + \mathbf{1}} \times \mathbf{100}\%$$

For a temperature of 298.15 K an *ee* of 94% can be calculated with would be an e.r. 97:3.

For a temperature of 353.15 K an *ee* of 89% can be calculated which would be an e.r. of 94.5 : 5.5.

5. Experimental procedures and characterization data

5.1. General Procedures

5.1.1 General procedure for preparation of thioether bromides (**G.P. A**)

General Procedure A-1 An example was prepared for compound **1a**. A 100 mL oven-dried, single-necked round-bottom flask under a nitrogen atmosphere was charged with NaH (21.4 mmol, 1.2 eq.), followed by three cycles of nitrogen and vacuum pumping. To the flask, 50 mL of dry THF was added, and the suspension was stirred vigorously. The corresponding thiol (17.8 mmol, 1.0 eq.) was then added dropwise as a THF solution (5 mL) using a syringe, and the mixture was stirred at room temperature for 2 hours. After this time, 2-chloroethanol (19.6 mmol, 1.1 eq.) was added dropwise via syringe to the resulting suspension. The reaction mixture was stirred for an additional hour at room temperature. The mixture was then taken to evaporate the THF and was extracted three times with DCM. The combined organic phases were dried with $MgSO_4$, filtered through a cotton plug, and the solvent was evaporated under reduced pressure to afford the desired thioether alcohol compound.

General Procedure A-2 An example was prepared for compound **1c**. In a 250 mL flask under nitrogen gas, dissolve the corresponding alcohol in 160 mL of dry DCM and stir for 5 minutes at room temperature. To the mixture, add PPh_3 (24.3 mmol, 1.3eq.) first, and then slowly add CBr_4 (24.3 mmol, 1.3 eq.) in portions. After the addition of CBr_4, the reaction mixture changes color to slightly yellow and begins to warm up slightly (in the case of a larger reaction scale, it is necessary to cool it down with an ice bath). The prepared mixture should be stirred overnight (for 16 hours). After the reaction, filter the DCM solution through Celite to remove most of the yellow impurity. Concentrate the filtrate to around 20 mL of DCM and add 150 mL of petroleum ether to precipitate most of the insoluble product. After the filtration, evaporate the solvent. Dissolve the solid product in 20 mL of DCM again and added 200 mL of hexane to precipitate the $POPh_3$ byproduct. The final filtration over SiO_2 yields a liquid that is concentrated under reduced pressure to give a clean product as an oil.

2-((adamantan-1-yl)thio)ethan-1-ol (1a)

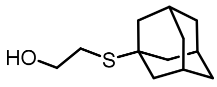

$C_{12}H_{20}OS$
212,35 g/mol

Following **G.P. A-1**, sodium hydride (NaH, 0.855 g, 21.39 mmol, 1.2 eq., 60% in oil) was added to the flask under a nitrogen atmosphere. Dry THF (50 mL) was introduced via canula, and a solution of adamant-1-thiol (3 g, 17.82 mmol, 1 eq.) in THF (5 mL) was added dropwise. The reaction mixture was stirred for 2 hours at room temperature. After this, 2-chloroethanol (1.32 mL, 19.6 mmol, 1.1 eq.) was added, and the mixture was allowed to react for an additional hour. The crude mixture was then subjected to evaporation and extraction to yield product **1a** (3.8 g, quantitative) as a light yellow oil.

^{1}H NMR (400 MHz, $CDCl_3$) δ 3.70 (td, *J* = 6.2, 1.5 Hz, 2H), 2.75 (td, *J* = 6.2, 1.9 Hz, 2H), 2.05 (s, 4H), 1.87 (s, 6H), 1.69 (s, 6H).

^{13}C NMR (101 MHz, $CDCl_3$) δ 61.8, 47.6, 44.6, 43.8, 36.2, 29.7.

IR (ATR neat) ṽ =: 3360, 2901, 2848, 1449, 1342, 1300, 1101, 1040, 1010, 975, 686, 475 cm^{-1}.

HRMS (ESI): *m/z* calculated for $C_{12}H_{20}NaOS^+$ [M + Na^+] 235.1127; found 235.1129.

2-(phenylthio)ethan-1-ol (1b)

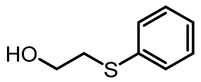

$C_8H_{10}OS$
154,23 g/mol

Following **G.P. A-1**, sodium hydride (NaH, 1.3 g, 32.67 mmol, 1.2 eq., 60% in oil) was added to the flask under a nitrogen atmosphere. Dry THF (50 mL) was introduced via canula, and a solution of thiophenol (3 g, 27.22 mmol, 1 eq.) in THF (5 mL) was added dropwise. The reaction mixture was stirred for 2 hours at room temperature. After this, 2-chloroethanol (2 mL, 29.95 mmol, 1.1 eq.) was added, and the mixture was allowed to react for an additional hour. The crude mixture was then subjected to evaporation and extraction to yield product **1b** (4.2 g, quantitative) as a light yellow oil.

^{1}H NMR (400 MHz, $CDCl_3$) δ 7.38 – 7.30 (m, 2H), 7.29 – 7.21 (m, 2H), 7.21 – 7.12 (m, 1H), 3.69 (t, *J* = 6.3 Hz, 2H), 3.05 (t, *J* = 6.3 Hz, 2H), 2.98 (s, 1H).

^{13}C NMR (101 MHz, $CDCl_3$) δ 135.2, 129.9, 129.1, 126.5, 60.5, 36.8.

The spectral data were consistent with the literature.[16]

(adamantan-1-yl)(2-bromoethyl)sulfane (1c)

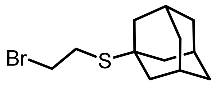

$C_{12}H_{19}BrS$
275,25 g/mol

Following **G.P. A-2**, 2-((adamantan-1-yl)thio)ethan-1-ol **1a** (3.8 g, 18.71 mmol, 1.0 eq.) was dissolved in 160 mL of dry DCM, followed by the addition of PPh_3 (6.38 g, 24.32 mmol, 1.3 eq.) and CBr_4 (8.06 g, 24.32 mmol, 1.3 eq.) in portions. The reaction mixture was stirred for 16 hours at room temperature. Filtration was performed to remove unreacted phosphine and $POPh_3$, provided product **1c** (5.2 g, quantitative) as a light yellow syrup without further purification.

^{1}H NMR (400 MHz, $CDCl_3$) δ 3.47 – 3.38 (m, 2H), 2.97 – 2.88 (m, 2H), 2.05 (s, 3H), 1.84 (d, *J* = 2.8 Hz, 6H), 1.77 – 1.61 (m, 6H).

^{13}C NMR (101 MHz, $CDCl_3$) δ 45.5, 43.8, 36.3, 31.8, 29.8, 28.6.

IR (ATR neat) ṽ =: 3360, 2901, 2848, 1449, 1342, 1300, 1101, 1040, 1010, 975, 686, 475 cm^{-1}.

HRMS (ESI): *m*/*z* calculated for $C_{12}H_{20}BrS^+$ [M + H^+] 275.0464; found 275.0487.

(2-bromoethyl)(phenyl)sulfane (1d)

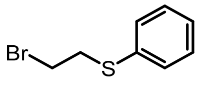

C_8H_9BrS
217,12 g/mol

Following **G.P. A-2**, 2-(phenylthio)ethan-1-ol **1b** (4.2 g, 27.23 mmol, 1.0 eq.) was dissolved in 160 mL of dry DCM, followed by the addition of PPh_3 (10.61 g, 40.46 mmol, 1.3 eq.) and CBr_4 (13.41 g, 40.46 mmol, 1.3 eq.) in portions. The reaction mixture was stirred for 16 hours at room temperature. Filtration was performed to remove unreacted phosphine and $POPh_3$, provided product **1d** (6 g, quantitative) as a light yellow oil without further purification.

^{1}H NMR (400 MHz, $CDCl_3$) δ 7.44 – 7.36 (m, 2H), 7.35 – 7.28 (m, 2H), 7.28 – 7.18 (m, 1H), 3.55 – 3.40 (m, 2H), 3.36 – 3.22 (m, 2H).

^{13}C NMR (101 MHz, $CDCl_3$) δ 134.1, 130.6, 129.3, 127.2, 36.1, 29.9.

The spectral data were consistent with the literature.[16]

5.1.2 General procedure for preparation of camphor ligands (**G.P. B**)

Scheme S2. Preparation of Camphor-Based Sulfur-Functionalized *N*-Heterocyclic Carbene Ligand Precursors.

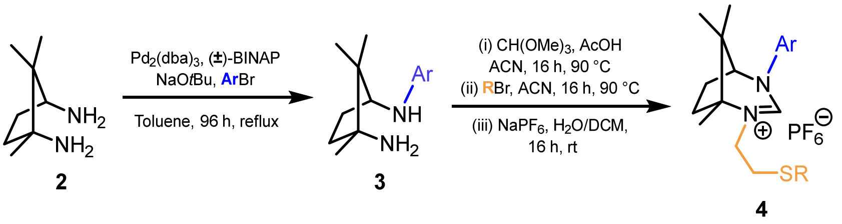

General procedure B-1 Preparation of camphor diamine **2**. To a vigorously stirred mixture of (1*R*,3*S*)-camphoric acid (15.00 g, 75 mmol), in concentrated H_2SO_4 (75 mL) and ethanol-free chloroform (300 mL), at 60 °C, was added sodium azide (14.61 g, 0.23 mol) in small amounts over a period of 2 h. The mixture was then stirred for a further 24 h at 60 °C (oil bath). The mixture was cooled, poured into H_2O (500 mL), and the aqueous phase extracted 3 times with $CHCl_3$. Aqueous phase made strongly basic with 12 M NaOH. The amine was extracted into $CHCl_3$ (6 × 200 mL), the organic extracts dried with anhydrous $MgSO_4$ and the chloroform removed in vacuo to give a clear oil. The oil was dissolved in diethyl ether (100 mL), the solution filtered, and the solvent removed in vacuo to give **2** as a white solid (9 g, 84 %).

^{1}H NMR (400 MHz, $CDCl_3$) δ 2.95 (dd, J = 8.5, 6.7 Hz, 1H), 2.05 – 1.92 (m, 1H), 1.73 – 1.54 (m, 2H), 1.53 (s, 4H), 1.27 (ddt, J = 13.2, 10.8, 6.7 Hz, 1H), 0.98 (s, 3H), 0.77 (s, 3H), 0.75 (s, 3H).

^{13}C NMR (101 MHz, $CDCl_3$) δ 61.0, 60.7, 46.1, 38.3, 30.2, 25.9, 22.2, 16.3.

The spectral data were consistent with the literature.[17]

General procedure B-2 An example was prepared for compound **3a**. Preparation of Cross Coupling Reaction Buchwald–Hartwig amination. In a Glovebox 100 mL vial charged with $Pd_2(dba)_3$ (0.42 mmol, 5 mol%), (±)-BINAP (0.84 mmol, 10 mol%) and NaO*t*Bu (25.3 mmol, 3 eq.) were dissolved in toluene and stirred for 20 min. Diamine **2** (8.43 mmol, 1 equiv.) and a bromoaryl (9.28 mmol, 1.1 eq.) were added and the solution was stirred for 72 h at 125°C (oil bath). The solution was filtered through a plug of silica and the plug was eluted with additional toluene. Thereafter, the plug was eluted with DCM/MeOH (9:1 v/v) until all the product was removed from the silica plug. The solvent was evaporated under reduced pressure and the crude product was dissolved in DCM. The organic phase was extracted with 12 M HCl. The aqueous phase was separated and washed three times with DCM. Thereafter, the aqueous phase was cooled to 0 °C and 12 M NaOH was slowly added until the solution reached a pH of 14. The basic solution was extracted five times with DCM and the combined organic phases were dried over $MgSO_4$, filtered and the solvent was removed. The crude product was dissolved in pentane and the solution was filtered to remove insoluble impurities. The solvent was removed and the product was further purified via chromatography if necessary.

General Procedure B-3: Preparation of Sulfur-Functionalized Camphor NHC Ligands. Camphor ligands were prepared through a three-step reaction from amines **3**, involving cyclization, alkylation, and finally, anion exchange. Based on our experience, only the second step has an impact on the reaction yields; cyclization and anion exchange are usually quantitative.

An example was prepared for compound **4a.** In the first reaction, the corresponding amine **3** (1.98 mmol, 1 eq.) was charged into a 50 mL Schlenk flask and dissolved in 10 mL of dry ACN. To the reaction solution, trimethyl orthoformate (9.9 mmol, 5 eq.) and acetic acid (9.9 mmol, 5 eq.) were added. The reaction flask, equipped with a condenser and an open Schlenk stub pipe (for the slow evaporation of the produced methanol), was set to 90 °C (oil bath) for 16 hours (overnight). The crude reaction mixture was concentrated under reduced pressure to remove unreacted orthoformate and ACN. The mixture was then dissolved in DCM and extracted with a 30% KOH solution. After separating the fractions, the aqueous phase was washed two more times with DCM. The combined

organic layers were dried with $MgSO_4$, filtered, and the solvent was evaporated to obtain the cyclic product in quantitative yield. The product was used in the next step without further purification.

The cyclic product was charged into a 50 mL Schlenk flask, dissolved in dry ACN (10 mL), and alkyl bromide (9.9 mmol, 5 eq) was added. The reaction mixture was stirred at 90 °C (oil bath) for 16 hours under a nitrogen atmosphere. The crude reaction mixture was then concentrated under reduced pressure to remove solvent. In this step, a controlled TLC was typically prepared to detect the formation of organic salt. The crude mixture was used in the next step without further purification.

For the ion exchange step, the previously prepared crude mixture was dissolved in DCM (10 mL), and H_2O (10 mL), followed by addition of $NaPF_6$ (4.95 mmol, 2.5 eq). The mixture was vigorously stirred overnight at room temperature. After completion of the reaction, the mixture was extracted three times with dichloromethane. The combined organic fractions were dried with $MgSO_4$, filtered through a cotton plug, and concentrated under reduced pressure. The crude reaction mixture was purified via column chromatography to obtain the final ligand **4** as the product.

(1*R*,3*S*)-1,2,2-trimethyl-*N*³-(o-tolyl)cyclopentane-1,3-diamine (3a)

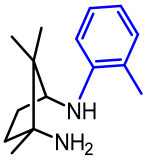

$C_{15}H_{24}N_2$
232,37 g/mol

Following **G.P. B-2**, $Pd_2(dba)_3$ (385 mg, 0.421 mmol, 5 mol%), (±)-BINAP (516 mg, 0.843 mmol, 10 mol%), and NaO*t*Bu (2.43 g, 25.3 mmol, 3 eq.) were added to anhydrous toluene (100 mL), and the reaction mixture was stirred at room temperature for 20 minutes. After this, **2** (1.2 g, 8.43 mmol, 1 eq.) and 2-bromotoluene (0.9 mL, 9.28 mmol 1.2 eq.) were added, and the solution was stirred at 125 °C for 72 hours. After the reaction was complete, the mixture was first filtered through SiO_2 with DCM (150 mL), and the cake was washed with an additional DCM/MeOH (200 mL). The crude reaction mixture was concentrated under reduced pressure and redissolved in pure DCM for acid-base extraction. After extraction, the crude product was dried with $MgSO_4$, filtered through a cotton plug, and concentrated under reduced pressure to yield product **3a** (1.93 g, 98%) as a light yellow syrup.

^{1}H NMR (400 MHz, $CDCl_3$) δ 7.06 (dt, 1H), 7.01 (d, *J* = 7.2 Hz, 1H), 6.63 – 6.50 (m, 2H), 5.15 (s, 1H), 3.66 (d, *J* = 6.2 Hz, 1H), 2.30 – 2.15 (m, 1H), 2.11 (s, 3H), 1.89 – 1.73 (m, 1H), 1.66 – 1.56 (m, 2H), 1.14 (s, 3H), 0.99 (s, 3H), 0.95 (s, 3H).

^{13}C NMR (101 MHz, $CDCl_3$) δ 146.6, 130.1, 126.9, 122.3, 115.6, 109.6, 77.4, 77.1, 76.8, 62.6, 61.5, 47.2, 38.4, 29.4, 26.8, 24.9, 17.9, 17.2.

The spectral data were consistent with the literature.[17]

(1*S*,3*R*)-*N*¹-mesityl-2,2,3-trimethylcyclopentane-1,3-diamine (3b)

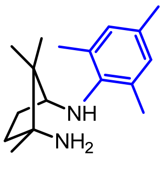

$C_{17}H_{28}N_2$
260,43 g/mol

Following **G.P. B-2**, $Pd_2(dba)_3$ (480 mg, 0.527 mmol, 5 mol%), (±)-BINAP (660 mg, 1.055 mmol, 10 mol%), and NaO*t*Bu (3.04 g, 31.64 mmol, 3 eq.) were added to anhydrous toluene (100 mL), and the reaction mixture was stirred at room temperature for 20 minutes. After this, **2** (1.5 g, 10.55 mmol, 1 eq.) and 2-bromomesitylen (1.91 mL, 12.65 mmol, 1.2 eq.) were added, and the solution was stirred at 125 °C for 72 hours. After the reaction was complete, the mixture was first filtered through SiO_2 with DCM (150 mL), and the cake was washed with an additional DCM/MeOH (200 mL). The crude reaction mixture was concentrated under reduced pressure and redissolved in pure DCM for acid-base extraction. After extraction, the crude product was dried with $MgSO_4$, filtered through a cotton plug, and concentrated under reduced pressure to yield product **3b** (2.6 g, 95%) as a light yellow syrup.

^{1}H NMR (400 MHz, $CDCl_3$) δ 6.79 – 6.74 (m, 2H), 4.18 (s, 1H), 3.41 (dd, *J* = 7.7, 4.9 Hz, 1H), 2.24 (s, 6H), 2.20 (s, 3H), 1.91 – 1.78 (m, 1H), 1.65 (dddd, *J* = 32.2, 13.5, 10.5, 5.3 Hz, 2H), 1.54 – 1.41 (m, 1H), 1.10 (s, 3H), 1.07 (s, 3H), 0.93 (s, 3H).

^{13}C NMR (101 MHz, $CDCl_3$) δ 143.6, 129.6, 129.6, 128.6, 77.4, 77.1, 76.7, 66.3, 61.1, 47.0, 38.2, 28.6, 26.9, 23.9, 20.5, 19.4, 17.1.

The spectral data were consistent with the literature.[17]

(1*R*,3*S*)-1,2,2-trimethyl-*N*³-(naphthalen-2-yl)cyclopentane-1,3-diamine (3c)

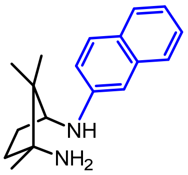

$C_{18}H_{24}N_2$
268,40 g/mol

Following **G.P. B-2**, $Pd_2(dba)_3$ (195 mg, 0.212 mmol, 5 mol%), (±)-BINAP (280 mg, 0.422 mmol, 10 mol%), and NaO*t*Bu (1.22 g, 12.65 mmol, 3 eq.) were added to anhydrous toluene (100 mL), and the reaction mixture was stirred at room temperature for 20 minutes. After this, **2** (600 mg, 4.22 mmol 1 eq.) and 2-bromonaphthalene (1.05 g, 5.06 mmol, 1.2 eq.) were added, and the solution was stirred at 125 °C for 72 hours. After the reaction was complete, the mixture was first filtered through SiO_2 with DCM (150 mL), and the cake was washed with an additional DCM/MeOH (200 mL). The crude reaction mixture was concentrated under reduced pressure and redissolved in pure DCM for acid-base extraction. After extraction, the crude product was dried with $MgSO_4$, filtered through a cotton plug, and concentrated under reduced pressure to yield product **3c** (965 mg, 85%) as a light yellow syrup.

Rf 0.41 (SiO_2, DCM/MeOH 9:1, v/v).

¹H NMR (600 MHz, $CDCl_3$) δ 7.64 (d, *J* = 7.9 Hz, 1H), 7.58 (t, *J* = 8.8 Hz, 2H), 7.33 (ddd, *J* = 8.2, 6.8, 1.3 Hz, 1H), 7.15 (ddd, *J* = 8.1, 6.9, 1.2 Hz, 1H), 6.88 (dd, *J* = 8.7, 2.4 Hz, 1H), 6.78 (d, *J* = 2.3 Hz, 1H), 5.34 (s, 1H), 3.78 (q, *J* = 7.2, 6.6 Hz, 1H), 2.33 – 2.23 (m, 1H), 1.88 – 1.79 (m, 1H), 1.70 – 1.59 (m, 2H), 1.17 (s, 3H), 1.00 (d, *J* = 2.2 Hz, 6H).

¹³C NMR (151 MHz, $CDCl_3$) δ 146.5, 135.6, 128.9, 127.7, 127.2, 126.2, 125.8, 121.4, 118.8, 104.2, 62.9, 61.6, 47.5, 38.4, 29.2, 26.8, 25.0, 17.4.

¹⁵N NMR (61 MHz, $CDCl_3$) δ -290.39, -325.9.

$[\alpha]^{25}_D$ = +107 (c = 1.00, $CHCl_3$).

IR (ATR neat) ṽ =: 3290, 3049, 2958, 2866, 1737, 1625, 1601, 1516, 1483 , 1228, 1186, 1141 cm^{-1}.

HRMS (ESI): *m*/*z* calculated for $C_{18}H_{25}N_2^+$ [M + H⁺] 269.2012; found 269.2021.

(1*R*,5*S*)-2-(2-(((3*R*,5*R*,7*R*)-adamantan-1-yl)thio)ethyl)-1,8,8-trimethyl-4-(o-tolyl)-2,4-diazabicyclo[3.2.1]oct-2-en-2-ium Hexafluorophosphate (4a)

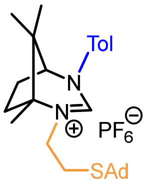

$C_{28}H_{41}F_6N_2PS$
582,67 g/mol

Following **G.P. B-3**, a 50 mL Schlenk flask was charged with **3a** (478 mg, 1.98 mmol, 1 eq.), methyl orthoformate (1.08 mL, 9.9 mmol, 5 eq.), and acetic acid (0.57 mL, 9.9 mmol, 5 eq.) in 10 mL of dry ACN. The reaction mixture was stirred for 16 hours at 90 °C. After an acid-base work-up, the crude product was used in the next step without further purification. In the second step, the crude product was dissolved in 10 mL of dry ACN, followed by the addition of **1c** (2.72 g, 9.9 mmol, 5 eq.). The reaction mixture was stirred for 16 hours at 90 °C under nitrogen. After the work-up, the crude product was redissolved in DCM (10 mL) and water (10 mL), followed by the addition of $NaPF_6$ (830 mg, 4.95 mmol, 2.5 eq.). The reaction mixture was stirred for 16 hours at room temperature. Column chromatography (SiO_2, DCM/petroleum ether/EtOAc 7:2:1, v/v) of the crude mixture yielded **4a** as a yellow solid (763 mg, 66% yield).

Rf 0.43 (SiO_2, DCM/MeOH 9:1, v/v).

m.p. 103 °C

¹H NMR (600 MHz, $CDCl_3$) δ 7.67 (s, 1H), 7.41 – 7.34 (m, 1H), 7.34 – 7.30 (m, 3H), 3.94 (dt, *J* = 15.3, 4.7 Hz, 1H), 3.74 (dq, *J* = 15.1, 5.1 Hz, 1H), 3.54 (d, *J* = 4.9 Hz, 1H), 2.91 – 2.81 (m, 2H), 2.73 (ddd, *J* = 14.3, 10.2, 5.4 Hz, 1H), 2.51 (ddd, *J* = 14.2, 9.5, 4.4 Hz, 1H), 2.43 (s, 3H), 2.17 (ddd, *J* = 14.6, 7.3, 4.9 Hz, 1H), 2.07 – 2.04 (m, 4H), 1.86 – 1.83 (m, 6H), 1.69 – 1.66 (m, 6H), 1.46 (s, 3H), 1.37 (s, 3H), 1.21 (s, 3H).

^{13}C NMR (151 MHz, $CDCl_3$) δ 154.5, 139.0, 133.5, 132.4, 130.0, 128.0, 127.5, 70.9, 70.8, 51.8, 46.2, 43.6, 42.0, 40.0, 36.0, 31.8, 29.6, 26.0, 21.9, 18.5, 17.6, 14.4.

^{15}N NMR (61 MHz, $CDCl_3$) δ -232.6.

$[\alpha]^{25}_{D}$ = -10 (c = 1.00, $CHCl_3$).

IR (ATR neat) ṽ =: 2903, 2848, 1642, 1581, 1450, 1376, 1321, 1298, 1188 cm^{-1}.

HRMS (ESI): *m*/*z* calculated for $C_{28}H_{41}N_2S^+$ [M - PF_6^-] 437.2985; found 437.2991.

(1*R*,5*S*)-2-(2-(((3*R*,5*R*,7*R*)-adamantan-1-yl)thio)ethyl)-4-mesityl-1,8,8-trimethyl-2,4-diazabicyclo[3.2.1]oct-2-en-2-ium Hexafluorophosphate (4b)

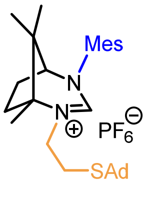

$C_{30}H_{45}F_6N_2PS$
610,73 g/mol

Following **G.P. B-3**, a 50 mL Schlenk flask was charged with **3b** (1.2 g, 4.22 mmol, 1 eq.), methyl orthoformate (2.31 mL, 21.12 mmol, 5 eq.), and acetic acid (1.2 mL, 21.12 mmol, 5 eq.) in 10 mL of dry ACN. The reaction mixture was stirred for 16 hours at 90 °C. After an acid-base work-up, the crude product was used in the next step without further purification. In the second step, the crude product was dissolved in 10 mL of dry ACN, followed by the addition of **1c** (4.4 g, 16 mmol, 3.8 eq.). The reaction mixture was stirred for 16 hours at 90 °C under nitrogen. After the work-up, the crude product was redissolved in DCM (10 mL) and water (10 mL), followed by the addition of $NaPF_6$ (1.77 g, 10.55 mmol, 2.5 eq.). The reaction mixture was stirred for 16 hours at room temperature. Column chromatography (SiO_2, DCM/petroleum ether/EtOAc 7:2:1, v/v) of the crude mixture yielded **4b** as a yellow solid (2.5 g, 89% yield).

Rf 0.593w2 (SiO_2, DCM/petroleum ether/EtOAc 7:2:1, v/v).

m.p. 128 °C

^{1}H NMR (600 MHz, $CDCl_3$) δ 7.60 (s, 1H), 7.02 (s, 1H), 6.99 (s, 1H), 3.92 (dt, *J* = 15.4, 4.6 Hz, 1H), 3.78 (ddd, *J* = 15.2, 9.2, 4.2 Hz, 1H), 3.54 (d, *J* = 3.6 Hz, 1H), 2.87 (dtt, *J* = 18.1, 9.2, 4.6 Hz, 2H), 2.72 (dt, *J* = 14.1, 4.8 Hz, 1H), 2.52 (s, 3H), 2.46 – 2.39 (m, 1H), 2.34 (s, 3H), 2.33 (s, 3H), 2.23 – 2.14 (m, 2H), 2.09 (s, 3H), 1.87 (s, 6H), 1.72 (s, 6H), 1.53 (s, 3H), 1.42 (s, 3H), 1.24 (s, 3H).

^{13}C NMR (151 MHz, $CDCl_3$) δ 155.1, 140.0, 135.0, 134.7, 134.6, 131.3, 130.6, 70.9, 70.3, 52.0, 45.9, 43.7, 43.6, 42.3, 40.2, 36.2, 36.1, 31.9, 29.7, 26.1, 22.1, 20.9, 19.3, 19.2, 18.0, 14.7.

^{15}N NMR (61 MHz, $CDCl_3$) δ -235.5.

$[\alpha]^{25}_{D}$ = -13 (c = 1.00, $CHCl_3$).

IR (ATR neat) ṽ =: 2905, 2850, 1641, 1450, 1375, 1316, 1300, 1212, 1042, 830 cm^{-1}.

HRMS (ESI): *m*/*z* calculated for $C_{30}H_{45}N_2S^+$ [M - PF_6^-] 465.3298; found 465.3295.

(1*R*,5*S*)-2-(2-(((3*R*,5*R*,7*R*)-adamantan-1-yl)thio)ethyl)-1,8,8-trimethyl-4-(naphthalen-2-yl)-2,4-diazabicyclo[3.2.1]oct-2-en-2-ium Hexafluorophosphate (4c)

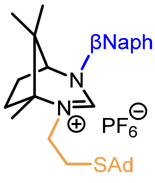

$C_{31}H_{41}F_6N_2PS$
618,71 g/mol

Following **G.P. B-3**, a 50 mL Schlenk flask was charged with **3c** (400 mg, 1.44 mmol, 1 eq.), methyl orthoformate (0.79 mL, 7.19 mmol, 5 eq.), and acetic acid (0.41 mL, 7.19 mmol, 5 eq.) in 10 mL of dry ACN. The reaction mixture was stirred for 16 hours at 90 °C. After an acid-base work-up, the crude product was used in the next step without further purification. In the second step, the crude product was dissolved in 10 mL of dry ACN, followed by the addition of **1c** (1.18 g, 4.31 mmol, 3 eq.). The reaction mixture was stirred for 16 hours at 90 °C under nitrogen. After the work-up, the crude product was redissolved in DCM (10 mL) and water (10 mL), followed by the addition of $NaPF_6$ (610 mg, 3.59 mmol, 2.5 eq.). The reaction mixture was stirred for 16 hours at room temperature. Column chromatography (SiO_2, DCM/petroleum ether/EtOAc 7:2:1, v/v) of the crude mixture yielded **4c** as a yellow solid (730 mg, 80% yield).

Rf 0.30 (SiO_2, DCM/petroleum ether/EtOAc 7:2:1, v/v).

m.p. 117 °C

^{1}H NMR (600 MHz, $CDCl_3$) δ 8.05 (s, 1H), 7.99 – 7.94 (m, 2H), 7.91 (d, *J* = 1.8 Hz, 1H), 7.85 (d, *J* = 7.2 Hz, 1H), 7.59 – 7.51 (m, 2H), 7.38 (dd, *J* = 8.8, 2.0 Hz, 1H), 3.97 – 3.90 (m, 1H), 3.89 (d, *J* = 5.0 Hz, 1H), 3.87 – 3.81 (m, 1H), 2.92 – 2.83 (m, 2H), 2.74 (dt, *J* = 11.5, 5.3 Hz, 1H), 2.54 (td, *J* = 9.7, 9.3, 4.5 Hz, 1H), 2.37 – 2.28 (m, 1H), 2.13 – 1.98 (m, 4H), 1.85 (s, 6H), 1.67 (t, *J* = 15.4 Hz, 6H), 1.45 (s, 3H), 1.26 (s, 3H), 1.23 (s, 3H).

^{13}C NMR (151 MHz, $CDCl_3$) δ 153.0, 137.7, 133.5, 132.6, 130.9, 128.5, 127.8, 127.8, 127.5, 121.4, 119.8, 71.4, 70.9, 51.6, 46.1, 43.6, 41.7, 40.5, 36.0, 32.2, 29.7, 26.0, 21.8, 17.0, 14.4.

^{15}N NMR (61 MHz, $CDCl_3$) δ -230.6.

$[\alpha]^{25}_D$ = +30 (c = 1.00, $CHCl_3$).

IR (ATR neat) ṽ =: 2903, 2849, 1644, 1628, 1598, 1451, 1378, 1324, 1298, 1213 cm^{-1}.

HRMS (ESI): *m/z* calculated for $C_{31}H_{41}N_2S^+$ [M - PF_6^-] 473.2985; found 473.2975.

(1*R*,5*S*)-1,8,8-trimethyl-2-(2-(phenylthio)ethyl)-4-(o-tolyl)-2,4-diazabicyclo[3.2.1]oct-2-en-2-ium Hexafluorophosphate (4d)

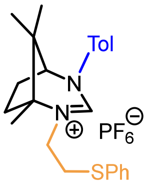

$C_{24}H_{31}F_6N_2PS$
524,55 g/mol

Following **G.P. B-3**, a 50 mL Schlenk flask was charged with **3a** (400 mg, 1.72 mmol, 1 eq.), methyl orthoformate (0.94 mL, 8.61 mmol, 5 eq.), and acetic acid (0.49 mL, 8.61 mmol, 5 eq.) in 10 mL of dry ACN. The reaction mixture was stirred for 16 hours at 90 °C. After an acid-base work-up, the crude product was used in the next step without further purification. In the second step, the crude product was dissolved in 10 mL of dry ACN, followed by the addition of **1d** (1.12 g, 5.16 mmol, 3 eq.). The reaction mixture was stirred for 16 hours at 90 °C under nitrogen. After the work-up, the crude product was redissolved in DCM (10 mL) and water (10 mL), followed by the addition of $NaPF_6$ (725 mg, 4.3 mmol, 2.5 eq.). The reaction mixture was stirred for 16 hours at room temperature. Column chromatography (SiO_2, DCM/petroleum ether/EtOAc 7:2:1, v/v) of the crude mixture yielded **4d** as a yellow wax (315 mg, 35% yield).

Rf 0.63 (SiO_2, DCM/MeOH 9:1, v/v).

^{1}H NMR (600 MHz, $CDCl_3$) δ 7.52 (s, 1H), 7.42 – 7.27 (m, 8H), 7.24 (tt, *J* = 7.2, 1.3 Hz, 1H), 3.94 (dt, *J* = 15.4, 5.4 Hz, 1H), 3.78 (ddd, *J* = 15.5, 8.1, 5.6 Hz, 1H), 3.61 (d, *J* = 4.9 Hz, 1H), 3.35 – 3.24 (m, 2H), 2.68 (ddd, *J* = 14.4, 9.5, 4.7 Hz, 1H), 2.51 (ddd, *J* = 14.0, 9.5, 4.3 Hz, 1H), 2.42 (s, 3H), 2.22 (ddt, *J* = 14.7, 12.2, 4.7 Hz, 1H), 2.12 (ddd, *J* = 14.7, 12.3, 4.3 Hz, 1H), 1.44 (s, 3H), 1.35 (s, 3H), 1.23 (s, 3H).

^{13}C NMR (151 MHz, $CDCl_3$) δ 154.5, 138.9, 133.5, 133.2, 132.6, 130.2, 130.1, 129.8, 128.1, 127.6, 71.5, 71.0, 49.5, 42.0, 40.4, 34.4, 32.0, 21.8, 18.4, 17.6, 14.3.

^{15}N NMR (61 MHz, $CDCl_3$) δ -231.7.

$[\alpha]^{25}_D$ = +2 (c = 1.00, $CHCl_3$).

IR (ATR neat) ṽ =: 3074, 2983, 1642, 1581, 1439, 1402, 1375, 1321, 1186, 874, 826, 765 cm^{-1}.

HRMS (ESI): *m/z* calculated for $C_{24}H_{31}N_2S^+$ [M - PF_6^-] 379.2202; found 379.2211.

(1*R*,5*S*)-4-mesityl-1,8,8-trimethyl-2-(2-(phenylthio)ethyl)-2,4-diazabicyclo[3.2.1]oct-2-en-2-ium Hexafluorophosphate (4e)

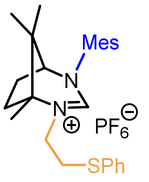

$C_{26}H_{35}F_6N_2PS$
552,60 g/mol

Following **G.P. B-3**, a 50 mL Schlenk flask was charged with **3b** (580 mg, 2.23 mmol, 1 eq.), methyl orthoformate (1.22 mL, 11.14 mmol, 5 eq.), and acetic acid (0.64 mL, 11.14 mmol, 5 eq.) in 10 mL of dry ACN. The reaction mixture was stirred for 16 hours at 90 °C. After an acid-base work-up, the crude product was used in the next step without further purification. In the second step, the crude product was dissolved in 10 mL of dry ACN, followed by the addition of **1d** (1.45 g, 6.68 mmol, 3 eq.). The reaction mixture was stirred for 16 hours at 90 °C under nitrogen. After the work-up, the crude product was redissolved in DCM (10 mL) and water (10 mL), followed by the addition of $NaPF_6$ (970 mg, 5.57 mmol, 2.5 eq.). The reaction mixture was stirred for 16 hours at room temperature. Column chromatography (SiO_2, DCM/petroleum ether/EtOAc 7:2:1, v/v) of the crude mixture yielded **4e** as a yellow solid (120 mg, 10% yield).

Rf 0.64 (SiO_2, DCM/MeOH 9:1, v/v).

m.p. 112 °C

^{1}H NMR (600 MHz, $CDCl_3$) δ 7.54 (s, 1H), 7.37 – 7.32 (m, 4H), 7.26 (tt, *J* = 6.3, 1.8 Hz, 1H), 7.01 (s, 1H), 6.98 (s, 1H), 3.87 (t, *J* = 6.2 Hz, 2H), 3.55 (d, *J* = 3.9 Hz, 1H), 3.32 – 3.21 (m, 2H), 2.71 – 2.63 (m, 1H), 2.46 (s, 3H), 2.43 – 2.36 (m, 1H), 2.33 (s, 3H), 2.32 (s, 3H), 2.25 – 2.13 (m, 2H), 1.45 (s, 3H), 1.38 (s, 3H), 1.23 (s, 3H).

^{13}C NMR (151 MHz, $CDCl_3$) δ 155.3, 140.0, 134.8, 134.5, 134.4, 133.3, 131.3, 130.6, 130.1, 129.7, 127.4, 70.9, 70.7, 49.6, 42.3, 40.4, 34.5, 31.9, 22.0, 20.9, 19.1, 18.7, 17.9, 14.5.

^{15}N NMR (61 MHz, $CDCl_3$) δ -236.8.

$[\alpha]^{25}_D$ = -143 (c = 1.00, $CHCl_3$).

IR (ATR neat) ṽ =: 3081, 2963, 2918, 1641, 1472, 1440, 1375, 1316, 1188, 1023 cm^{-1}.

HRMS (ESI): *m*/*z* calculated for $C_{26}H_{35}N_2S^+$ [M - PF_6^-] 407.2515; found 407.2528.

(1*R*,5*S*)-2-(cyclohexylmethyl)-4-mesityl-1,8,8-trimethyl-2,4-diazabicyclo[3.2.1]oct-2-en-2-ium Hexafluorophosphate (4f)

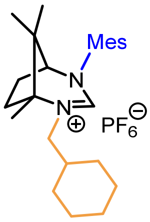

$C_{25}H_{39}F_6N_2P$
512,57 g/mol

Following **G.P. B-3**, a 50 mL Schlenk flask was charged with **3b** (500 mg, 1.92 mmol, 1 eq.), methyl orthoformate (1.05 mL, 9.61 mmol, 5 eq.), and acetic acid (0.55 mL, 9.61 mmol, 5 eq.) in 10 mL of dry ACN. The reaction mixture was stirred for 16 hours at 90 °C. After an acid-base work-up, the crude product was used in the next step without further purification. In the second step, the crude product was dissolved in 10 mL of dry ACN, followed by the addition of Cyclohexylmethyl bromide (2.68 mL, 19.22 mmol, 10 eq.). The reaction mixture was stirred for 16 hours at 90 °C under nitrogen. After the work-up, the crude product was redissolved in DCM (10 mL) and water (10 mL), followed by the addition of $NaPF_6$ (810 mg, 4.8 mmol, 2.5 eq.). The reaction mixture was stirred for 16 hours at room temperature. Column chromatography (SiO_2, Et_2O, followed by DCM) of the crude mixture yielded **4f** (570 mg, 58%) as a light yellow solid.

Rf 0.67 (SiO_2, DCM/MeOH 9:1, v/v).

m.p. 98 °C

^{1}H NMR (600 MHz, $CDCl_3$) δ 7.54 (s, 1H), 6.95 (s, 1H), 6.93 (s, 1H), 3.50 (d, *J* = 4.0 Hz, 1H), 3.41 (d, *J* = 6.9 Hz, 2H), 2.52 (ddd, *J* = 13.5, 9.4, 4.8 Hz, 1H), 2.34 (s, 3H), 2.27 (d, *J* = 4.8 Hz, 6H), 2.25 – 2.17 (m, 4H), 1.84 (d, *J* = 12.2 Hz, 1H), 1.81 – 1.72 (m, 2H), 1.70 – 1.62 (m, 2H), 1.62 – 1.54 (m, 1H), 1.47 (s, 3H), 1.34 (s, 3H), 1.31 – 1.20 (m, 3H), 1.21 (s, 3H), 1.03 – 0.99 (m, 1H).

^{13}C NMR (151 MHz, $CDCl_3$) δ 155.0, 139.9, 134.9, 134.6, 134.2, 131.4, 130.6, 70.6, 70.5, 57.8, 42.2, 40.5, 38.1, 31.9, 31.8, 30.4, 30.0, 26.2, 25.9, 25.7, 25.6, 22.0, 20.9, 20.6, 19.2, 19.1, 18.7, 17.9, 14.8.

^{15}N NMR (61 MHz, $CDCl_3$) δ -235.8.

$[\alpha]^{25}_D$ = -116 (c = 1.00, $CHCl_3$).

IR (ATR neat) ṽ =: 3335, 2925, 2854, 1686, 1642, 1479, 1449 cm^{-1}.

HRMS (ESI): *m/z* calculated for $C_{25}H_{39}N_2^+$ [M - PF_6^-] 367.3108; found 367.3113.

5.1.3 General procedures for preparation of camphor catalysts (**G.P.C**)

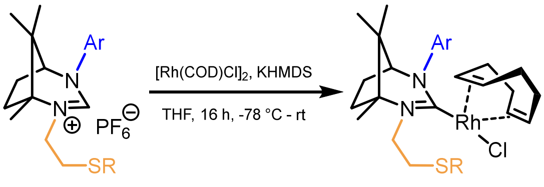

4 **Rh5**

General procedure C: An example was prepared for compound **5a.** In the glovebox, a 10 mL vial was charged with $[Rh(COD)Cl]_2$ (41.1 µmol, 1 eq.), camphor ligand **4** (90.5 µmol, 2.2 eq), and KHMDS (90.5 µmol, 2.2 eq). The vial was then sealed with a Teflon cap and taken out of the glovebox. The reaction flask was cooled to -78 °C, and dry THF (3 - 5 mL) was slowly added. After the addition of the solvent, the reaction mixture was maintained at -78 °C for an additional hour, and then slowly warmed to room temperature. The reaction mixture was immediately subjected to column chromatography. After the separation of fractions, the collected product **Rh5** was transferred to a vial and stored under nitrogen in the refrigerator. All complex showed a decomposition above 250 °C.

Complex Rh5a

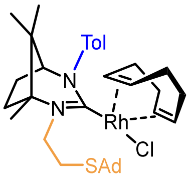

$C_{36}H_{52}ClN_2RhS$
683,24 g/mol

Following **G.P. C**, $[Rh(COD)Cl]_2$ (20 mg, 41.13 µmol, 1 eq.), **4a** (53 mg, 90.5 µmol, 2.2 eq.), and KHMDS (18 mg, 90.5 µmol, 2.2 eq.) were dissolved in anhydrous THF (5 mL). The reaction mixture was stirred at -78 °C for 1 hour and then slowly warmed to room temperature over 16 hours. Column chromatography (SiO_2, petroleum ether/EtOAc 1:1, followed by DCM/pentane/Et_2O 7:2:1, v/v) of the crude mixture yielded **Rh5a** as a yellow solid (17 mg, 31%). Mixture of rotamers ca. 9:1, not assigned.

Rf 0.65 (SiO_2, DCM/MeOH 9:1, v/v).

^{1}H NMR (600 MHz, $CDCl_3$) δ 7.83 (d, *J* = 7.0 Hz, 1H), 7.51 (td, *J* = 7.5, 1.1 Hz, 1H), 7.47 (d, *J* = 6.7 Hz, 1H), 7.39 (dt, *J* = 7.4, 1.2 Hz, 1H), 5.04 (t, *J* = 7.4 Hz, 1H), 4.81 (q, *J* = 8.1 Hz, 1H), 4.49 (dt, *J* = 15.5, 2.8 Hz, 1H), 4.22 – 4.12 (m, 2H), 3.36 (d, *J* = 4.6 Hz, 1H), 3.26 (d, *J* = 12.6 Hz, 1H), 3.04 (td, *J* = 12.6, 2.5 Hz, 1H), 2.84 – 2.79 (m, 1H), 2.44 (ddd, *J* = 20.3, 9.8, 4.8 Hz, 1H), 2.40 (s, 3H), 2.32 (ddd, *J* = 14.9, 9.6, 4.8 Hz, 1H), 2.22 (d, *J* = 5.7 Hz, 3H), 2.15 – 2.04 (m, 3H), 2.00 (dd, *J* = 39.7, 12.6 Hz, 6H), 1.96 – 1.86 (m, 3H), 1.84 – 1.76 (m, 6H), 1.48 (s, 3H), 1.42 – 1.38 (m, 2H), 1.35 – 1.31 (m, 1H), 1.28 – 1.24 (m, 1H), 1.14 (s, 3H), 1.11 (s, 3H).

^{13}C NMR (151 MHz, $CDCl_3$) δ 203.6 (d, *J* = 41 Hz, 1C), 142.9, 133.4, 132.1, 129.1, 126.9, 124.3, 99.6 (d, *J* = 6.5 Hz, 1C), 97.2 (d, *J* = 6 Hz, 1C), 78.3 (d, *J* = 16 Hz, 1C), 71.4 (d, *J* = 12 Hz, 1C), 70.8, 69.6, 51.8, 50.7, 42.7, 42.5, 42.5, 40.4, 36.3, 36.0, 31.9, 31.0, 30.6, 30.0, 30.0, 29.8, 28.8, 26.5, 25.8, 22.6, 18.5, 18.0, 16.7.

^{15}N NMR (61 MHz, $CDCl_3$) δ -223.6.

IR (ATR neat) ṽ =: 2918, 2851, 1764, 1627, 1487, 1463, 1036, 829, 758 cm^{-1}.

HRMS (ESI): *m/z* calculated for $C_{36}H_{52}N_2RhS^+$ [M – Cl^-] 647.2901; found 647.2905.

Complex Rh5b

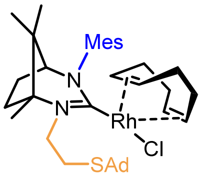

$C_{38}H_{56}ClN_2RhS$
711,30 g/mol

Following **G.P. C**, $[Rh(COD)Cl]_2$ (70 mg, 140 µmol, 1 eq.), **4b** (191 mg, 320 µmol, 2.2 eq.), and KHMDS (62 mg, 320 µmol, 2.2 eq.) were dissolved in anhydrous THF (5 mL). The reaction mixture was stirred at -78 °C for 1 hour and then slowly warmed to room temperature over 48 hours. Column chromatography (SiO_2, petroleum ether/EtOAc 1:1, followed by DCM/pentane/Et_2O 7:2:1, v/v) of the crude mixture yielded **Rh5b** as a yellow solid (180 mg, 89%). The product was designated as a single rotamer.

Rf 0.12 (SiO_2, DCM/pentane/Et_2O 7:2:1, v/v).

^{1}H NMR (^{1}H NMR (400 MHz, $CDCl_3$) δ 7.13 (s, 1H), 6.97 (s, 1H), 5.27 (t, *J* = 7.5 Hz, 1H), 5.00 (q, *J* = 8.0 Hz, 1H), 4.73 (ddd, *J* = 15.5, 12.9, 2.9 Hz, 1H), 4.46 (d, *J* = 15.2 Hz, 1H), 4.32 (s, 1H), 3.23 (d, *J* = 3.8 Hz, 1H), 3.16 (d, *J* = 11.8 Hz, 1H), 3.05 – 2.92 (m, 2H), 2.58 (s, 3H), 2.43 (ddd, *J* = 20.5, 9.7, 5.4 Hz, 1H), 2.36 (s, 3H), 2.33 – 2.17 (m, 4H), 2.17 – 2.10 (m, 4H), 2.08 – 1.99 (m, 3H), 1.97 – 1.92 (m, 7H), 1.94 – 1.83 (m, 1H), 1.79 – 1.67 (m, 7H), 1.45 – 1.41 (m, 4H), 1.16 (t, *J* = 8.4 Hz, 1H), 1.12 (s, 3H), 1.02 (s, 3H), 0.92 – 0.80 (m, 1H).

^{13}C NMR (101 MHz, $CDCl_3$) δ 202.0 (d, J = 44 Hz, 1C), 140.1, 138.2, 136.0, 134.0, 130.7, 130.4, 95.2 (d, J = 6.9 Hz, 1C), 91.9 (d, J = 6.5 Hz, 1C), 80.1 (d, J = 14.5 Hz, 1C), 73.5(d, J = 12.7 Hz, 1C), 72.6, 69.1, 52.8, 52.6, 42.5, 41.5, 40.8, 35.9, 35.8, 32.0, 31.3, 30.0, 28.3, 26.9, 25.9, 23.3, 22.4, 20.8, 20.3, 19.0, 17.6.

^{15}N NMR (61 MHz, $CDCl_3$) δ -225.32

$[\alpha]^{25}_D$ = -36 (c = 1.00, $CHCl_3$).

IR (ATR neat) ṽ =: 2912, 2853, 1478, 1450, 1374, 1297, 1036, 838, 752, 555 cm^{-1}.

HRMS (ESI): *m*/*z* calculated for $C_{38}H_{56}N_2RhS^+$ [M – Cl^-] 675.3214; found 675.3210.

Complex Rh5c

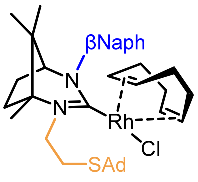

$C_{39}H_{52}ClN_2RhS$
719,27 g/mol

Following **G.P. C**, $[Rh(COD)Cl]_2$ (15 mg, 30.4 µmol, 1 eq.), **4c** (41 mg, 66.9 µmol, 2.2 eq.), and KHMDS (13 mg, 66.9 µmol, 2.2 eq.) were dissolved in anhydrous THF (3 mL). The reaction mixture was stirred at -78 °C for 1 hour and then slowly warmed to room temperature over 16 hours. Column chromatography (SiO_2, petroleum ether/EtOAc 1:1, followed by DCM/pentane/Et_2O 7:2:1, v/v) of the crude mixture yielded **Rh5c** as a yellow solid (27 mg, 61%). Mixture of rotamers ca. 8:2, not assigned.

Rf 0.16 (SiO_2, DCM/pentane/Et_2O 7:2:1, v/v).

^{1}H NMR (600 MHz, $CDCl_3$) δ 8.45 (d, *J* = 1.7 Hz, 0.8 $H_{maj.}$), 8.27 (s, 0.2 $H_{min.}$), 8.08 (d, *J* = 8.8 Hz, 0.8 $H_{maj.}$), 8.05 (d, *J* = 8.8 Hz, 0.2 $H_{min.}$), 8.00 (d, *J* = 7.8 Hz, 0.8 $H_{maj.}$), 7.98 (d, *J* = 8.2 Hz, 0.2 $H_{min.}$), 7.88 (d, *J* = 8.1 Hz, 0.8 $H_{maj.}$), 7.85 (d, *J* = 8.1 Hz, 0.2 $H_{min.}$), 7.72 – 7.66 (m, 1H), 7.64 (ddd, *J* = 8.1, 6.9, 1.1 Hz, 0.8 $H_{maj.}$), 7.61 (ddd, *J* = 8.1, 6.9, 1.1 Hz, 0.2 $H_{min.}$), 7.49 (dd, *J* = 8.8, 2.1 Hz, 1H), 5.01 (t, *J* = 6.4 Hz, 0.2 $H_{min.}$), 4.95 (t, *J* = 6.1 Hz, 0.8 $H_{maj.}$), 4.83 (q, *J* = 8.1 Hz, 0.2 $H_{min.}$), 4.77 (q, *J* = 8.0 Hz, 0.8 $H_{maj.}$), 4.57 – 4.42 (m, 1H), 4.14 (s, 0.8 $H_{maj.}$), 4.07 (dd, *J* = 14.3, 12.2 Hz, 0.2 $H_{min.}$), 4.03 – 3.95 (m, 1H), 3.88 (d, *J* = 4.9 Hz, 0.8 $H_{maj.}$), 3.80 (d, *J* = 5.3 Hz, 0.2 $H_{min.}$), 3.27 (t, *J* = 10.9 Hz, 1H), 2.96 (td, *J* = 12.8, 2.1 Hz, 0.8 $H_{maj.}$), 2.63 (td, *J* = 12.2, 1.9 Hz, 0.2 $H_{min.}$), 2.59 – 2.46 (m, 1H), 2.46 – 2.35 (m, 2H), 2.28 (s, 2.4 $H_{maj.}$), 2.25 (s, 0.6 $H_{min.}$), 2.16 – 2.01 (m, 8H), 2.01 – 1.90 (m, 3H), 1.84 (s, 6H), 1.70 – 1.53 (m, 2H), 1.53 (s, 0.6 $H_{min.}$), 1.50 (s, 2.4 $H_{maj.}$), 1.48 – 1.43 (m, 1H), 1.41 – 1.35 (m, 1H), 1.27 – 1.21 (m, 1H), 1.18 (s, 2.4 $H_{maj.}$), 1.11 (s, 0.6 $H_{min.}$), 1.05 (s, 2.4 $H_{maj.}$), 1.05 (s, 0.6 $H_{min.}$).

^{13}C NMR (151 MHz, $CDCl_3$) δ 200.7, 200.4, 145.1, 143.5, 134.1, 133.8, 132.5, 130.9, 128.5, 128.4, 127.0, 126.6, 120.7, 115.2, 104.1, 98.2, 98.2, 79.9, 79.8, 73.4, 73.1, 73.0, 71.6, 71.0,

51.3, 49.5, 42.7, 42.2, 41.8, 41.2, 41.0, 40.7, 36.2, 36.0, 36.0, 33.2, 31.2, 29.9, 29.9, 29.3, 26.8, 26.2, 22.8, 22.4, 18.1, 17.4, 16.6, 15.9.

^{15}N NMR (61 MHz, $CDCl_3$) δ -223.9.

IR (ATR neat) ṽ =: 2913, 2851, 1732, 1627, 1596, 1486, 1463, 1379, 1295, 665, 555, 477 cm^{-1}.

HRMS (ESI): *m*/*z* calculated for $C_{39}H_{52}N_2RhS^+$ [M – Cl^-] 683.2901; found 683.2905.

Complex Rh5d

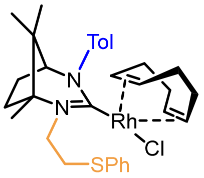

$C_{32}H_{42}ClN_2RhS$
625,12 g/mol

Following **G.P. C**, $[Rh(COD)Cl]_2$ (20 mg, 41.13 µmol, 1 eq.), **4d** (48 mg, 90.5 µmol, 2.2 eq.), and KHMDS (18 mg, 90.5 µmol, 2.2 eq.) were dissolved in anhydrous THF (5 mL). The reaction mixture was stirred at -78 °C for 1 hour and then slowly warmed to room temperature over 16 hours. Column chromatography (SiO_2, petroleum ether/EtOAc 1:1, followed by DCM/pentane/Et_2O 7:2:1, v/v) of the crude mixture yielded **Rh5d** as a yellow solid (25 mg, 49%). Mixture of rotamers ca. 85:15, not assigned.

Rf 0.65 (SiO_2, DCM/MeOH 9:1, v/v).

^{1}H NMR (600 MHz, $CDCl_3$) δ 7.65 – 7.55 (m, 2H), 7.54 – 7.48 (m, 3H), 7.48 – 7.36 (m, 4H), 4.90 (ddd, *J* = 15.2, 12.3, 2.7 Hz, 0.15 $H_{min.}$), 4.80 (ddd, *J* = 15.4, 12.5, 3.0 Hz, 0.85 $H_{maj.}$), 4.71 (dd, *J* = 12.7, 2.5 Hz, 0.15 $H_{min.}$), 4.65 (d, *J* = 15.4 Hz, 0.85 $H_{maj.}$), 4.56 – 4.41 (m, 1H), 4.38 (t, *J* = 7.2 Hz, 0.85 $H_{maj.}$), 4.33 (t, *J* = 7.7 Hz, 0.15 $H_{min.}$), 3.81 (q, *J* = 8.0 Hz, 1H), 3.77 (dt, *J* = 13.6, 2.6 Hz, 1H), 3.68 (td, *J* = 12.8, 2.6 Hz, 0.85 $H_{maj.}$), 3.44 (td, *J* = 12.6, 2.5 Hz, 0.15 $H_{min.}$), 3.36 (d, *J* = 4.7 Hz, 0.85 $H_{maj.}$), 3.32 (d, *J* = 5.6 Hz, 0.15 $H_{min.}$), 3.02 (q, *J* = 7.2 Hz, 0.85 $H_{maj.}$), 2.74 (q, *J* = 7.2 Hz, 0.15 $H_{min.}$), 2.65 (ddt, *J* = 20.5, 9.7, 4.8 Hz, 0.15 $H_{min.}$), 2.57 (ddt, *J* = 15.8, 9.7, 4.8 Hz, 0.85 $H_{maj.}$), 2.38 (s, 3H), 2.31 (dtd, *J* = 19.1, 9.4, 9.0, 4.7 Hz, 1H), 2.24 – 2.01 (m, 3H), 2.02 – 1.86 (m, 2H), 1.83 (td, *J* = 15.2, 12.7, 8.9 Hz, 1H), 1.50 (s, 0.15 $H_{min.}$), 1.49 (s, 2.5 $H_{maj.}$), 1.48 – 1.37 (m, 3H), 1.25 – 1.20 (m, 1H), 1.19 (s, 2.5 $H_{maj.}$), 1.12 (s, 2.5 $H_{maj.}$), 1.07 (s, 0.15 $H_{min.}$), 0.98 (s, 0.15 $H_{min.}$).

^{13}C NMR (151 MHz, $CDCl_3$) δ 205.0, 204.7, 142.5, 132.8, 132.5, 130.1, 130.0, 129.8, 129.4, 129.0, 128.9, 128.3, 126.9, 124.9, 98.9, 98.9, 95.2, 95.1, 81.8, 81.7, 75.1, 75.0, 70.4, 69.8, 53.2, 42.5, 40.6, 39.2, 35.6, 30.8, 30.4, 26.8, 25.8, 22.4, 22.3, 18.5, 18.1, 16.8.

^{15}N NMR (61 MHz, $CDCl_3$) δ -222.8.

IR (ATR neat) ṽ =: 2954, 2922, 2853, 1769, 1481, 1462, 1440, 1376, 1322, 663, 555, 478 cm^{-1}.

HRMS (ESI): *m*/*z* calculated for $C_{32}H_{42}N_2RhS^+$ [M – Cl^-] 589.2118; found 589.2124.

Complex Rh5e

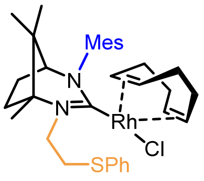

$C_{34}H_{46}ClN_2RhS$
653,17 g/mol

Following **G.P. C**, $[Rh(COD)Cl]_2$ (15 mg, 30 µmol, 1 eq.), **4d** (37 mg, 66 µmol, 2.2 eq.), and KHMDS (14 mg, 66 µmol, 2.2 eq.) were dissolved in anhydrous THF (5 mL). The reaction mixture was stirred at -78 °C for 1 hour and then slowly warmed to room temperature over 16 hours. Column chromatography (SiO_2, petroleum ether/EtOAc 1:1, followed by DCM/pentane/Et_2O 7:2:1, v/v) of the crude mixture yielded **Rh5e** as a yellow oil (9 mg, 23%). The product was designated as a single rotamer.

Rf 0.70 (SiO_2, DCM/MeOH 9:1, v/v).

^{1}H NMR (600 MHz, $CDCl_3$) δ 7.52 (dd, *J* = 8.3, 1.0 Hz, 2H), 7.44 (t, *J* = 7.8 Hz, 2H), 7.35 (t, *J* = 7.4 Hz, 1H), 7.13 (s, 1H), 6.96 (s, 1H), 5.00 (ddd, *J* = 15.3, 12.6, 2.6 Hz, 1H), 4.68 – 4.61 (m, 2H), 4.20 (t, *J* = 7.5 Hz, 1H), 3.65 (d, *J* = 13.3 Hz, 1H), 3.52 (q, *J* = 8.0 Hz, 1H), 3.49 – 3.43 (m, 1H), 3.26 (d, *J* = 4.0 Hz, 1H), 3.15 (q, *J* = 7.2 Hz, 1H), 2.62 (s, 3H), 2.61 – 2.52 (m, 1H), 2.35 (s, 3H), 2.26 – 2.20 (m, 4H), 2.17 (td, *J* = 9.8, 4.8 Hz, 1H), 2.11 – 2.03 (m, 1H), 1.93 – 1.85 (m, 2H), 1.77 (dt, *J* = 13.6, 8.8

Hz, 1H), 1.68 (dtd, J = 18.8, 10.1, 5.1 Hz, 1H), 1.45 (s, 3H), 1.37 – 1.27 (m, 3H), 1.19 – 1.12 (m, 4H), 1.05 (s, 3H).

^{13}C NMR (151 MHz, $CDCl_3$) δ 203.5 (d, J = 42 Hz, 1C), 140.2, 138.3, 137.7, 135.9, 134.2, 130.7, 130.4, 130.2, 129.7, 128.4, 97.6, 96.9 (d, J = 7.5 Hz, 1C), 94.9 (d, J = 7.5 Hz, 1C), 83.6 (d, J = 14 Hz, 1C), 72.4, 69.4, 54.6, 41.8, 41.1, 37.6, 35.8, 32.0, 31.1, 27.2, 25.6, 22.8, 22.5, 20.9, 20.2, 19.1, 17.8.

^{15}N NMR (61 MHz, $CDCl_3$) δ -225.5.

IR (ATR neat) ṽ =: 2924, 2880, 1625, 1579, 1476, 1440, 1397, 1373, 1300, 1212, 655, 585 cm^{-1}.

HRMS (ESI): *m*/*z* calculated for $C_{34}H_{46}N_2RhS^+$ [M – Cl] 617.2431; found 617.2441.

Complex Rh5f

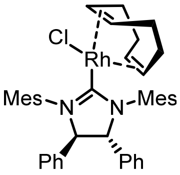

$C_{41}H_{46}ClN_2Rh$
705,19 g/mol

Following **G.P. C**, $[Rh(COD)Cl]_2$ (20 mg, 41 µmol, 1 eq.), **Ph_2SIMes BF_4** (53 mg, 97 µmol, 2.4 eq.), and KHMDS (19 mg, 97 µmol, 2.4 eq.) were dissolved in anhydrous THF (3 mL). The reaction mixture was stirred at -78 °C for 1 hour and then slowly warmed to room temperature over 16 hours. Column chromatography (SiO_2, petroleum ether/EtOAc 1:1, v/v) of the crude mixture yielded **Rh5f** as a yellow solid (58 mg, 99%). The product was designated as a single rotamer. The ligand was prepared in the standard way described in the literature.[18]

Rf 0.12 (SiO_2, DCM/MeOH 9:1, v/v).

^{1}H NMR (600 MHz, $CDCl_3$) δ 7.38 – 7.33 (m, 2H), 7.33 – 7.23 (m, 8H), 7.01 (s, 1H), 6.95 (s, 1H), 6.85 (s, 1H), 6.78 (s, 1H), 5.65 (d, J = 11.0 Hz, 1H), 5.57 (d, J = 11.0 Hz, 1H), 4.56 (td, J = 7.6, 3.4 Hz, 1H), 4.44 (q, J = 7.6, 6.8 Hz, 1H), 3.51 (s, 1H), 3.28 (t, J = 6.8 Hz, 1H), 2.90 (s, 3H), 2.57 (s, 3H), 2.36 (s, 3H), 2.31 (s, 3H), 2.30 (s, 3H), 1.95 (s, 3H), 1.94 – 1.88 (m, 2H), 1.70 – 1.61 (m, 4H), 1.58 – 1.48 (m, 2H).

^{13}C NMR (151 MHz, $CDCl_3$) δ 212.0, 211.7, 139.3, 138.2, 137.5, 137.5, 136.8, 136.7, 135.6, 135.5, 134.4, 134.3, 130.3, 130.1, 129.1, 129.1, 128.9, 128.6, 128.5, 128.5, 128.3, 98.0, 98.0, 96.5, 96.4, 73.6, 73.4, 70.3, 70.2, 65.4, 65.3, 34.2, 31.2, 29.4, 26.8, 22.3, 21.0, 21.0, 20.9, 20.0, 19.7.

^{15}N NMR (61 MHz, $CDCl_3$) δ -227.5.

IR (ATR neat) ṽ =: 2915, 2875, 2830, 1604, 1479, 1455, 1403, 1377, 1342, 1306 cm^{-1}.

HRMS (ESI): *m*/*z* calculated for $C_{41}H_{46}N_2Rh^+$ [M – Cl^-] 669.2711; found 669.2716.

Complex Rh5g

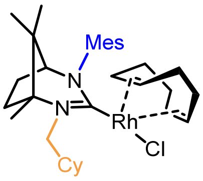

$C_{33}H_{50}ClN_2Rh$
613,13 g/mol

Following **G.P. B-1**, $[Rh(COD)Cl]_2$ (30 mg, 61 µmol, 1 eq.), **3af** (69 mg, 134 µmol, 2.2 eq.), and KHMDS (27 mg, 134 µmol, 2.2 eq.) were dissolved in anhydrous THF (5 mL). The reaction mixture was stirred at -78 °C for 1 hour and then slowly warmed to room temperature over 16 hours. Column chromatography (SiO_2, DCM/MeOH 95:5, v/v) of the crude mixture yielded **Rh5g** as a yellow solid (28 mg, 64%). Mixture of rotamers ca. 85:15, not assigned.

Rf 0.80 (SiO_2, DCM/MeOH v/v).

^{1}H NMR (600 MHz, $CDCl_3$) δ 6.95 (d, J = 5.3 Hz, 1H), 6.81 (d, J = 40.5 Hz, 1H), 4.39 (dt, J = 15.5, 6.5 Hz, 2H), 3.41 – 3.30 (m, 1H), 3.04 (d, J = 3.1 Hz, 1H), 2.89 (s, 1H), 2.77 (d, J = 4.0 Hz, 0H), 2.60 (s, 0H), 2.47 (s, 3H), 2.43 – 2.28 (m, 3H), 2.23 (t, J = 7.3 Hz, 6H), 2.20 – 2.14 (m, 1H), 1.94 – 1.80 (m, 3H), 1.80 – 1.63 (m, 6H), 1.60 (d, J = 12.6 Hz, 1H), 1.55 – 1.41 (m, 5H), 1.40 – 1.23 (m, 5H), 1.13 – 1.07 (m, 2H), 1.07 – 1.00 (m, 1H), 0.97 (s, 3H), 0.85 (d, J = 25.2 Hz, 3H), 0.81 – 0.71 (m, 1H).

^{13}C NMR (151 MHz, $CDCl_3$) δ 211.1, 210.7, 137.0, 133.6, 130.8, 130.7, 129.6, 128.9, 96.7, 96.6, 93.6, 93.6, 72.3, 71.2, 69.2, 69.1, 68.9, 43.0, 42.2, 39.9, 39.5, 39.4, 39.0, 35.4, 32.2, 32.1, 31.2, 31.1, 30.7,

29.8, 29.8, 29.8, 29.3, 27.3, 27.0, 26.8, 26.7, 26.7, 26.5, 24.4, 23.6, 23.6, 21.3, 20.9, 20.8, 20.3, 20.3, 19.2, 17.6.

^{15}N NMR (61 MHz, $CDCl_3$) δ -220.8.

IR (ATR neat) ṽ =: 2919, 2850, 1724, 1686, 1641, 1609, 1541, 1447 cm^{-1}.

HRMS (ESI): *m/z* calculated for $C_{33}H_{50}N_2Rh^+$ [M – Cl^-] 577.3024; found 577.3037.

Complex Rh(SIMes)

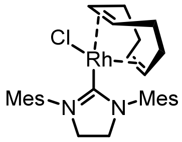

$C_{29}H_{38}ClN_2Rh$
552,99 g/mol

Following **G.P. C**, $[Rh(COD)Cl]_2$ (38 mg, 77 µmol, 1 eq.), **SIMesBr** (66 mg, 170 µmol, 2.2 eq.), and KHMDS (36 mg, 170 µmol, 2.4 eq.) were dissolved in anhydrous THF (3 mL). The reaction mixture was stirred at -78 °C for 1 hour and then slowly warmed to room temperature over 16 hours. Column chromatography (SiO_2, petroleum ether/EtOAc 1:1, v/v) of the crude mixture yielded **Rh(SIMes)** as a yellow solid (86 mg, 99%). Mixture of atropoisomers ca. 6:4, not assigned. The ligand was prepared in the standard way described in the literature.[19]

^{1}H NMR (400 MHz, $CDCl_3$) δ 6.97 – 6.82 (m, 4H), 4.55 – 4.33 (m, 2H), 3.86 – 3.68 (m, 4H), 3.49 – 3.19 (m, 2H), 2.52 (s, 6H), 2.25 (s, 12H), 1.75 – 1.63 (m, 4H), 1.51 – 1.33 (m, 4H).

^{13}C NMR (101 MHz, $CDCl_3$) δ 213.0, 212.8, 212.5, 212.3, 138.5, 138.3, 137.9, 137.9, 136.4, 136.4, 135.3, 135.3, 130.1, 130.0, 128.5, 128.5, 97.3, 97.2, 96.8, 96.7, 68.7, 68.6, 67.7, 67.6, 51.6, 51.5, 32.7, 32.6, 31.6, 29.8, 28.5, 28.2, 22.7, 21.1, 20.8, 20.0, 18.5, 18.4, 14.2.

The spectral data were consistent with the literature.[19]

5.1.4 Synthesis of starting materials for Asymmetric Ring Opening reaction (**G. P. D**)

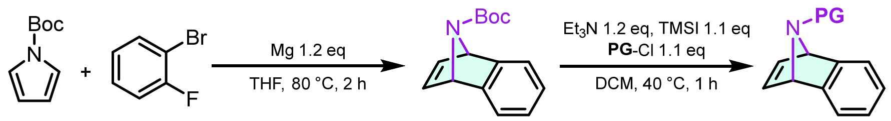

***tert*-butyl (1*R*,4*S*)-1,4-dihydro-1,4-epiminonaphthalene-9-carboxylate (6*N*-Boc)**

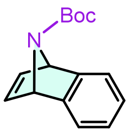

$C_{15}H_{17}NO_2$
243,31 g/mol

A 250 mL three-neck round-bottom flask equipped with a condenser, magnetic stir bar, and dropping funnel was charged with activated magnesium turnings (861 mg, 35.88 mmol, 1.2 eq.). The flask was flame-dried under vacuum, flushed with argon, and allowed to cool. *N*-Boc-pyrrole (5 g, 29.90 mmol, 1 eq.) in 100 mL of dry THF was then added to the flask and heated to a gentle reflux. A solution of *o*-Fluorobromobenzene (3.92 mL, 32.90 mmol, 1.1 eq.) in 50 mL of dry THF was introduced dropwise under an argon atmosphere over 30 minutes, followed by refluxing at 80 °C (oil bath) for additional 2 hours. The initiation of the reaction was indicated by the solution becoming turbid, then turning yellow. After cooling and evaporating THF, the mixture was poured into a flask containing 500 mL of an aqueous solution of ammonium chloride (300 g) and concentrated ammonium hydroxide (10 mL, 25.0% w/w NH_3). The aqueous layer was extracted with petroleum ether (3 × 50 mL), and the combined organic layers were dried over anhydrous magnesium sulfate, yielding a dark oil. This crude product was purified by column chromatography (SiO_2 petroleum ether/EtOAc 95:5 v/v). Producing compound **6*N*-Boc** as a yellow crystalline solid (3.75 g, 52% yield).

Rf 0.53 (SiO_2, Petroleum ether/EtOAc 9:1, v/v).

^{1}H NMR (400 MHz, $CDCl_3$) δ 7.25 (s, 2H), 6.95 (dd, *J* = 5.1, 3.0 Hz, 4H), 5.48 (s, 2H), 1.37 (s, 9H).

^{13}C NMR (101 MHz, $CDCl_3$) δ 155.2, 148.3, 125.0, 80.6, 28.2.

The spectral data were consistent with the literature.[20]

General Procedure for the Synthesis of *N*-Protected Compound (G.P. D).

An example was prepared for compound **6*N*-Ts. 6*N*-Boc** (4.93 mmol, 1 eq.) was dissolved in 100 mL of DCM in a round-bottom flask. To this solution, Et_3N (5.92 mmol, 1.2 eq.) was added, and the reaction mixture was heated to reflux. TMSI (5.42 mmol, 1.1 eq.) was added dropwise over 10 minutes, followed by an additional 15 minutes of heating under reflux at 40 °C (oil bath). The progress of the reaction was monitored by TLC, confirming complete consumption of the *N*-Boc starting material. After reflux, the reaction mixture was cooled to 0 °C using an ice bath. Methanol (1 mL) was added dropwise, and the mixture was stirred at 0 °C for 10 minutes. Sulfonyl chloride (4.93 mmol, 1 eq.) was then added, and the reaction mixture was allowed to gradually warm to room temperature. The reaction was stirred for an additional hour at room temperature. After the reaction time, 50 mL of water was added to the mixture, along with sufficient DCM to dissolve any precipitates formed. The organic and aqueous layers were separated, and the aqueous phase was extracted twice with DCM. The combined organic layers were dried over anhydrous $MgSO_4$, filtered, and concentrated under reduced pressure. The crude product was purified by column chromatography, resulting in the *N*-protected compound as a crystalline solid.

(*1R*,*4S*)-9-tosyl-1,4-dihydro-1,4-epiminonaphthalene (6*N*-Ts)

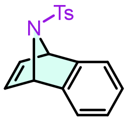

$C_{17}H_{15}NO_2S$
297,37 g/mol

Following **G.P. D**, **6*N*-Boc** (1.2 g, 4.93 mmol, 1 eq.) was dissolved in 100 mL of DCM, followed by the addition of Et_3N (0.820 mL, 5.92 mmol, 1.2 eq.). The mixture was heated to reflux, and TMSI (0.770 mL, 5.42 mmol, 1.1 eq.) was added dropwise over 10 minutes. The reaction was then refluxed for an additional 15 minutes. The reaction mixture was cooled to 0 °C, and MeOH (1 mL) was added dropwise. After stirring at 0 °C for 10 minutes, 4-Toluenesulfonyl chloride (940 mg, 4.93 mmol, 1 eq.) was added, and the reaction was allowed to warm to room temperature, stirring for an additional hour. Water (50 mL) and DCM were added to dissolve any precipitates, and the organic and aqueous layers were separated. The aqueous layer was extracted twice with DCM, and the combined organic layers were dried over $MgSO_4$ and concentrated. Column chromatography (SiO_2, petroleum ether/EtOAc 6:4, v/v) gave as dark brown solid (845 mg, 58%).

Rf 0.34 (SiO_2, Petroleum ether/EtOAc 1:1, v/v).

^{1}**H NMR** (400 MHz, $CDCl_3$) δ 7.46 (d, *J* = 7.9 Hz, 2H), 7.09 (d, *J* = 7.9 Hz, 2H), 7.02 (dd, *J* = 5.2, 3.1 Hz, 2H), 6.82 – 6.71 (m, 4H), 5.44 (t, *J* = 1.5 Hz, 2H), 2.33 (s, 3H).

^{13}**C NMR** (101 MHz, $CDCl_3$) δ 155.2, 148.3, 143.6, 142.4, 125.0, 121.1, 80.6, 66.2, 28.2.

The spectral data were consistent with the literature.[21]

(1*R*,4*S*)-9-((2-nitrophenyl)sulfonyl)-1,4-dihydro-1,4-epiminonaphthalene (6*N*-Ns)

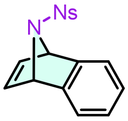

$C_{16}H_{12}N_2O_4S$
328,34 g/mol

Following **G.P. D**, **6*N*-Boc** (400 mg, 1.65 mmol, 1 eq.) was dissolved in 100 mL of DCM, followed by the addition of Et_3N (0.284 mL, 1.98 mmol, 1.2 eq.), and the mixture was heated to reflux. TMSI (0.260 mL, 1.80 mmol, 1.1 eq.) was then added dropwise over 10 minutes. The reaction was heated at reflux for an additional 15 minutes. The mixture was cooled to 0 °C, and MeOH (1 mL) was added dropwise. After 10 minutes at 0 °C, 2-nitrobenzene-1-sulfonyl chloride (365 mg, 1.65 mmol, 1 eq.) was introduced, leading to the formation of a white precipitate as the reaction warmed to room temperature. The reaction was stirred for an additional hour at room temperature. Water (50 mL) and DCM were added to dissolve the precipitates, and the organic and aqueous layers were separated. The aqueous phase was

extracted twice with DCM, and the combined organic layers were dried over $MgSO_4$ and concentrated. Column chromatography (SiO_2, Hex/EtOAc 8:2, v/v) gave as light brown solid (145 mg, 26%)

Rf 0.65 (SiO_2, Hex/EtOAc 8:2, v/v).

^{1}H NMR (400 MHz, $CDCl_3$) δ 7.75 – 7.70 (m, 1H), 7.53 (td, *J* = 1.5, 7.6 Hz, 1H), 7.49 – 7.40 (m, 2H), 7.16 (dd, *J* = 3.0, 5.2 Hz, 2H), 6.94 (t, *J* = 1.6 Hz, 2H), 6.80 (dd, *J* = 3.0, 5.2 Hz, 2H), 5.70 (t, *J* = 1.6 Hz, 2H).

^{13}C NMR (101 MHz, $CDCl_3$) δ 147.0, 142.8, 133.4, 131.7, 131.3, 131.2, 125.3, 123.8, 121.4, 77.3, 77.0, 76.7, 68.4.

IR (ATR neat) ṽ =: 3103, 3050, 3018, 2922, 2851, 1590, 1532, 1455, 1447, 1364, 1338, 1287, 1255, 1184, 1155, 1125, 1087, 1057, 1026, 1014, 905, 851, 789, 738, 728, 708, 692, 655, 645, 500 cm^{-1}.

HRMS (ESI): *m/z* calculated for $C_{16}H_{12}N_2NaO_4S^+$ [M + Na^+] 351.0410; found 351.0418.

(1*R*,4*S*)-9-((4-nitrophenyl)sulfonyl)-1,4-dihydro-1,4-epiminonaphthalene (6*N*-*p*Ns)

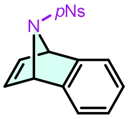

$C_{16}H_{12}N_2O_4S$
328,34 g/mol

Following **G.P. D**, **6*N*-Boc** (400 mg, 1.65 mmol, 1 eq.) was dissolved in 100 mL of DCM, followed by the addition of Et_3N (0.284 mL, 1.98 mmol, 1.2 eq.), and the mixture was heated to reflux. TMSI (0.260 mL, 1.80 mmol, 1.1 eq.) was then added dropwise over 10 minutes. The reaction was heated at reflux for an additional 15 minutes. The mixture was cooled to 0 °C, and MeOH (1 mL) was added dropwise. After 10 minutes at 0 °C, 4-nitrobenzene-1-sulfonyl chloride (365 mg, 1.65 mmol, 1 eq) was introduced, leading to the formation of a white precipitate as the reaction warmed to room temperature. The reaction was stirred for an additional hour at room temperature. Water (50 mL) and DCM were added to dissolve the precipitates, and the organic and aqueous layers were separated. The aqueous phase was extracted twice with DCM, and the combined organic layers were dried over $MgSO_4$ and concentrated. Column chromatography (SiO_2, Hex/EtOAc 8:2, v/v) gave as light brown solid (100 mg, 16%)

Rf 0.41 (SiO_2, Hex/EtOAc 8:2, v/v).

^{1}H NMR (400 MHz, $CDCl_3$) δ 7.73 (dd, *J* = 1.4, 7.6 Hz, 1H), 7.63 – 7.35 (m, 3H), 7.16 (dd, *J* = 3.0, 5.2 Hz, 2H), 6.94 (t, *J* = 1.6 Hz, 2H), 6.80 (dd, *J* = 3.0, 5.2 Hz, 2H), 5.70 (t, *J* = 1.6 Hz, 2H).

^{13}C NMR (101 MHz, $CDCl_3$) δ 147.0, 142.8, 133.4, 131.7, 131.3, 131.2, 125.3, 123.8, 121.4, 77.3, 77.0, 76.7, 68.4.

The spectral data were consistent with the literature.[21]

(1*R*,4*S*)-9-((4-bromophenyl)sulfonyl)-1,4-dihydro-1,4-epiminonaphthalene (6*N*-BsBr)

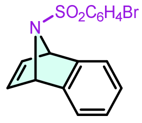

$C_{16}H_{12}BrNO_2S$
362,24

Following **G.P. D**, **6*N*-Boc** (800 mg, 3.3 mmol, 1 eq.) was dissolved in 100 mL of DCM, followed by the addition of Et_3N (0.568 mL, 3.96 mmol, 1.2 eq.), and the mixture was heated to reflux. TMSI (0.520 mL, 3.6 mmol, 1.1 eq.) was then added dropwise over 10 minutes. The reaction was heated at reflux for an additional 15 minutes. The mixture was cooled to 0 °C, and MeOH (1 mL) was added dropwise. After 10 minutes at 0 °C, 4-bromobenzene-1-sulfonyl chloride (843 mg, 3.3 mmol, 1 eq.) was introduced, leading to the formation of a white precipitate as the reaction warmed to room temperature. The reaction was stirred for an additional hour at room temperature. Water (50 mL) and DCM were added to dissolve the precipitates, and the organic and aqueous layers were separated. The aqueous phase was extracted twice with DCM, and the combined organic layers were dried over $MgSO_4$ and concentrated. Column chromatography (SiO_2, Hex/EtOAc 7:3, v/v) gave yellow crystalline (625 mg, 54%).

Rf 0.52 (SiO_2, Hex/EtOAc 7:3, v/v).

^{1}H NMR (^{1}H NMR (400 MHz, $CDCl_3$) δ 7.40 (s, 4H), 7.01 (dd, *J* = 5.1, 3.0 Hz, 2H), 6.86 (t, *J* = 1.6 Hz, 2H), 6.84 – 6.75 (m, 2H), 5.44 (t, *J* = 1.6 Hz, 2H).

^{13}C NMR (101 MHz, $CDCl_3$) δ 146.9, 142.6, 137.0, 131.9, 129.7, 127.5, 125.2, 121.4, 67.7.

The spectral data were consistent with the literature.[21]

(1*R*,4*S*)-9-((4-chlorophenyl)sulfonyl)-1,4-dihydro-1,4-epiminonaphthalene (6*N*-BsCl)

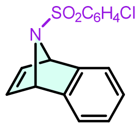

$C_{16}H_{12}ClNO_2S$
317,79 g/mol

Following **G.P. D**, **6*N*-Boc** (300 mg, 1.23mmol, 1 eq.) was dissolved in 100 mL of DCM, followed by the addition of Et_3N (0.205 mL, 1.48 mmol, 1.2 eq.), and the mixture was heated to reflux. TMSI (0.271 mL, 1.36 mmol, 1.1 eq.) was then added dropwise over 10 minutes. The reaction was heated at reflux for an additional 15 minutes. The mixture was cooled to 0 °C, and MeOH (1 mL) was added dropwise. After 10 minutes at 0 °C, 4-chlorobenzene-1-sulfonyl chloride (260 mg, 1.23 mmol, 1 eq.) was introduced, leading to the formation of a white precipitate as the reaction warmed to room temperature. The reaction was stirred for an additional hour at room temperature. Water (50 mL) and DCM were added to dissolve the precipitates, and the organic and aqueous layers were separated. The aqueous phase was extracted twice with DCM, and the combined organic layers were dried over $MgSO_4$ and concentrated. Column chromatography (SiO_2, Hex/EtOAc 8:2, v/v) gave brown solid (160 mg, 41%)

Rf 0.42 (SiO_2, Hex/EtOAc 8:2, v/v).

^{1}H NMR (400 MHz, $CDCl_3$) δ 7.50 – 7.44 (m, 1H), 7.26 – 7.19 (m, 1H), 7.01 (dd, *J* = 3.0, 5.1 Hz, 1H), 6.87 (t, *J* = 1.6 Hz, 1H), 6.79 (dd, *J* = 3.0, 5.2 Hz, 1H), 5.44 (t, *J* = 1.6 Hz, 1H).

^{13}C NMR (101 MHz, $CDCl_3$) δ 147.1, 142.7, 139.1, 129.8, 129.0, 125.4, 121.5, 67.8.

The spectral data were consistent with the literature.[21]

(1*R*,4*S*)-9-((4-fluorophenyl)sulfonyl)-1,4-dihydro-1,4-epiminonaphthalene (6*N*-BsF)

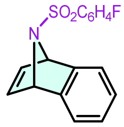

$C_{16}H_{12}FNO_2S$
301,34 g/mol

Following **G.P. D**, **6*N*-Boc** (300 mg, 1.23mmol, 1 eq.) was dissolved in 100 mL of DCM, followed by the addition of Et_3N (0.205 mL, 1.48 mmol, 1.2 eq.), and the mixture was heated to reflux. TMSI (0.271 mL, 1.36 mmol, 1.1 eq.) was then added dropwise over 10 minutes. The reaction was heated at reflux for an additional 15 minutes. The mixture was cooled to 0 °C, and MeOH (1 mL) was added dropwise. After 10 minutes at 0 °C, 4-flurobenzene-1-sulfonyl chloride (239 mg, 1.23 mmol, 1 eq.) was introduced, leading to the formation of a white precipitate as the reaction warmed to room temperature. The reaction was stirred for an additional hour at room temperature. Water (50 mL) and DCM were added to dissolve the precipitates, and the organic and aqueous layers were separated. The aqueous phase was extracted twice with DCM, and the combined organic layers were dried over $MgSO_4$ and concentrated. Column chromatography (SiO_2, Hex/EtOAc 8:2, v/v) gave as light brown solid (180 mg, 48%)

Rf 0.37 (SiO_2, Hex/EtOAc 8:2, v/v).

^{1}H NMR (400 MHz, $CDCl_3$) δ 7.55 (dd, *J* = 8.9, 5.1 Hz, 2H), 7.01 (dd, *J* = 5.2, 3.0 Hz, 2H), 6.94 (t, *J* = 8.6 Hz, 2H), 6.86 (d, *J* = 1.7 Hz, 2H), 6.78 (dd, *J* = 5.2, 3.0 Hz, 2H), 5.44 (t, *J* = 1.7 Hz, 2H).

^{13}C NMR (101 MHz, $CDCl_3$) δ 166.3, 163.8, 147.0, 142.7, 134.1, 134.1, 131.1, 131.0, 125.3, 121.5, 121.4, 116.2, 116.1, 115.8, 67.8.

^{19}F NMR (377 MHz, $CDCl_3$) δ -106.0.

IR (ATR neat) ṽ =: 3302, 3105, 3049, 1586, 1489, 1453, 1404, 1335, 1287 cm^{-1}.

HRMS (ESI): *m/z* calculated for $C_{16}H_{12}FNNaO_2S^+$ [M + Na^+] 324.0465; found 324.0476.

The spectral data were consistent with the literature.[22]

Synthesis of 6b and 6c

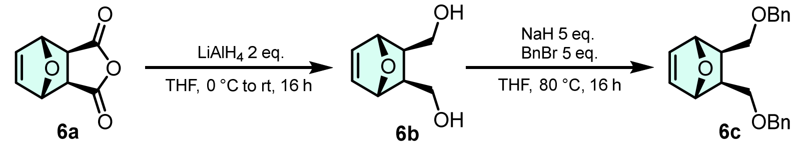

In a flame-dried 250 mL round-bottom flask, $LiAlH_4$ (1.52 g, 40 mmol, 2 eq.) was added, and the flask was evacuated and flushed with nitrogen twice. To the prepared $LiAlH_4$, 65 mL of dry THF was added, and the suspension was cooled to 0 °C in an ice bath. After 15 minutes at this temperature, a solution of **6a** (3.32 g, 20 mmol, 1 eq.) in 15 mL of dry THF was added dropwise over 15 minutes. The reaction was allowed to slowly warm up to room temperature over 16 hours. After this time, the reaction mixture was cooled down to 0 °C for 15 minutes and quenched with 15 mL of water, stirring for an additional 10 minutes. Subsequently, 35 mL of 15% NaOH was added, and the mixture was stirred for 20 minutes. Then, 15 mL of water was added, and the mixture was stirred again for 10 minutes. The reaction mixture was then filtered through Celite, and the fractions were separated. The aqueous fraction was washed twice with 20 mL of EtOAc, and the combined organic fractions were dried over $MgSO_4$. The solution was filtered through a cotton plug, and the solvents were evaporated under reduced pressure to obtain the clean product **6b** (1.95 g, 62%) as a light yellow oil.[23]

((1*R*,2*R*,3*S*,4*S*)-7-oxabicyclo[2.2.1]hept-5-ene-2,3-diyl)dimethanol 6b

^{1}H NMR (400 MHz, $CDCl_3$) δ 6.40 (t, *J* = 0.9 Hz, 2H), 4.69 (t, *J* = 0.9 Hz, 2H), 3.84 (ddp, *J* = 15.5, 11.0, 5.0 Hz, 4H), 3.45 (s, 2H), 2.01 – 1.88 (m, 2H).

^{13}C NMR (101 MHz, $CDCl_3$) δ 135.9, 81.4, 63.0, 42.7.

In a glovebox, prepare a suspension of NaH (640 mg, 16 mmol, 5 eq., 60% suspension in oil) in 20 mL of dry THF within a 100 mL vial. Added a solution of **6b** (500 mg, 3.2 mmol, 1 eq.) in 10 mL of dry THF. The vial was sealed with a septum cap, and the reaction was stirred at 80 °C (heating mantle) for 1 hour. After this time, benzyl bromide (1.9 mL, 16 mmol, 5 eq.) was added through the septum cap, and the reaction was stirred at 80 °C (heating mantle) for an additional 16 hours. The reaction mixture was then removed from the glovebox and quenched with 1 mL of water by slow addition at room temperature. Following the addition, the mixture was extracted with EtOAc (3 x 40 mL). The organic fractions were combined, dried with $MgSO_4$, filtered through a cotton plug, and the solvent was removed under reduced pressure. The crude product was purified by column chromatography (SiO_2, petroleum ether/EtOAc 7:3, v/v) to yield the final product **6c** (812 mg, 75%) as a light yellow oil.[23]

(1*R*,4*S*,5*S*,6*R*)-5,6-bis((benzyloxy)methyl)-7-oxabicyclo[2.2.1]hept-2-ene 6c

^{1}H NMR (400 MHz, $CDCl_3$) δ 7.44 – 7.27 (m, 10H), 6.35 (t, *J* = 0.9 Hz, 2H), 4.88 (t, *J* = 0.9 Hz, 2H), 4.52 (d, *J* = 11.9 Hz, 2H), 4.48 (d, *J* = 11.7 Hz, 2H), 3.60 (dd, *J* = 8.8, 5.1 Hz, 2H), 3.46 – 3.34 (m, 2H), 1.97 (ddd, *J* = 7.5, 5.2, 2.3 Hz, 2H).

^{13}C NMR (101 MHz, $CDCl_3$) δ 138.4, 135.7, 128.6, 127.9, 127.8, 80.9, 73.5, 70.0, 40.1.

5.2 Scope of products for ARO reaction (**G. P. E)**

General procedure E: Catalytic Asymmetric Ring Opening Reaction: A representative example of the catalytic ring-opening reaction of compound **7a**. To a flame-dried test vial (5 mL), catalyst (5.6 μmol, 4 mg) prepared from the stock solution ($CHCl_3$) is transferred. The vial containing the catalyst is then placed in a glovebox after the solvent has evaporated. Next, oxabenzonorbornadiene (0.112 mmol, 1 eq.) is added, followed by corresponding aniline or phenol (0.56 mmol, 5 eq), and finally, the solvent (0.22 mL, 0.5 M) is added. The vial is sealed with a Teflon cap and the temperature is set to 80 °C (heating mantle) for 16 to 24 hours. After the reaction is complete, the vial is removed from the glovebox and subsequent manipulations are performed under ambient air. The crude mixture is transferred to a flask and the solvent is evaporated under reduced pressure. The internal standard (IS) CH_2Br_2 is added to the residue, and a crude NMR is prepared to determine the conversion and yield of the reaction. Subsequently, column chromatography is carried out to purify the product and determine the isolated yield.

4-bromo-*N*-((1*S*,2*S*)-2-(1-methyl-1*H*-indol-3-yl)-1,2-dihydronaphthalen-1-yl) benzenesulfonamide (7aa)

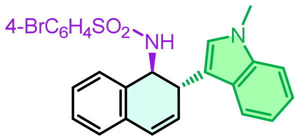

$C_{25}H_{21}BrN_2O_2S$
493,42 g/mol

Following **G. P. E** vial containing **Rh5b** (4 mg, 5 mol%) was charged to glovebox and into reaction vial added ***N*-BsBr** (40 mg, 0.112 mmol, 1 eq.) and *N*-methylindole (70 µL, 0.56 mmol, 5 eq.) followed by dry $MeNO_2$ (0.22 mL) reaction mixture was stirred at 80 °C for 16 h. After the reaction was completed, the vial was taken from glovebox and concentrated under reduced pressure, from the crude reaction mixture prepared NMR sample. Column chromatography (SiO_2, Hex/EtOAc 8:2, v/v) of the crude mixture gave **7aa** as a light brown solid (50 mg, 92%). The *ee* was determined to be 84 % using chiral HPLC (OD-H, *i*-propanol/*n*-hexane = 15/85, flow rate = 0.5 mL/min, l = 254 nm) t_R = 33.7 (minor), 41.9 (major).

Rf 0.73 (SiO_2, Hex/EtOAc 8:2, v/v).

^{1}H NMR (400 MHz, $CDCl_3$) δ 7.68 (d, *J* = 8.0 Hz, 1H), 7.51 (d, *J* = 8.3 Hz, 2H), 7.41 (d, *J* = 8.2 Hz, 2H), 7.30 – 7.19 (m, 3H), 7.15 (t, *J* = 7.2 Hz, 2H), 7.08 (t, *J* = 7.5 Hz, 1H), 6.84 (d, *J* = 7.6 Hz, 1H), 6.67 (d, *J* = 9.6 Hz, 1H), 6.52 (s, 1H), 6.11 (dd, *J* = 9.5, 4.8 Hz, 1H), 5.17 (d, *J* = 8.2 Hz, 1H), 4.68 (dd, *J* = 8.1, 5.4 Hz, 1H), 4.16 (t, *J* = 5.3 Hz, 1H), 3.56 (s, 3H).

^{13}C NMR (101 MHz, $CDCl_3$) δ 139.9, 137.4, 132.9, 132.8, 131.9, 129.7, 128.7, 128.4, 128.3, 128.0, 127.3, 127.3, 127.1, 126.9, 126.7, 121.9, 119.3, 119.2, 111.4, 109.6, 57.2, 39.2, 32.7.

$[\alpha]^{25}_D$ = +98 (c = 1.00, $CHCl_3$).

IR (ATR neat) ṽ =: 3277, 3049, 2923, 2852, 1573, 1470, 1325, 1276, 1153, 1066 cm^{-1}.

HRMS (ESI): *m/z* calculated for $C_{25}H_{21}BrN_2NaO_2S^+$ [M + Na$^+$] 515.0399; found 515.0393.

***ee* determination:**

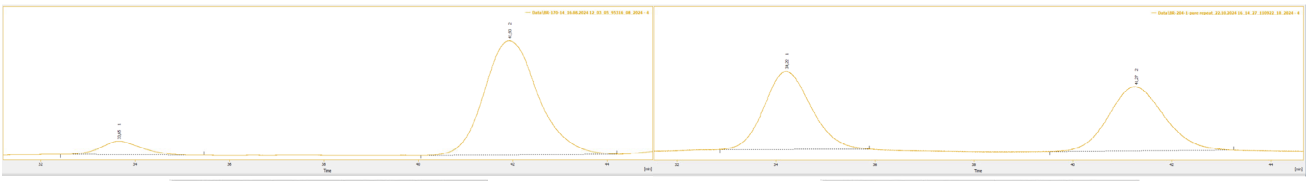

	Reten. Time [min]	Area [mAU.s]	Height [mAU]	Area [%]
1	33,653	4484,425	77,007	7,8
2	41,934	53234,417	672,424	92,2
	Total	57718,842	749,431	100,0

	Reten. Time [min]	Area [mAU.s]	Height [mAU]	Area [%]
1	34,215	4735,603	72,097	50,7
2	41,275	4602,122	59,078	49,3
	Total	9337,725	131,175	100,0

4-bromo-*N*-((1*S*,2*S*)-6,7-difluoro-2-(1-methyl-1*H*-indol-3-yl)-1,2-dihydronaphthalen-1-yl) benzenesulfonamide (7ab)

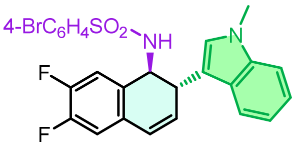

$C_{25}H_{19}BrF_2N_2O_2S$
529,40 g/mol

Following **G. P. E** vial containing **Rh5b** (4 mg, 5 mol%) was charged to glovebox and into reaction vial added (1*R*,4*S*)-9-((4-bromophenyl)sulfonyl)-6,7-difluoro-1,4-dihydro-1,4-epiminonaphthalene[21] (44 mg, 0.112 mmol, 1 eq.) and *N*-methylindole (70 µL, 0.56 mmol, 5 eq.) followed by dry $MeNO_2$ (0.22 mL) reaction mixture was stirred at 80 °C for 16 h. After the reaction was completed, the vial was taken from glovebox and concentrated under reduced pressure, from the crude reaction mixture prepared NMR sample. Column chromatography (SiO_2, Hex/EtOAc 9:1, v/v) of the crude mixture gave **7ab** as a light brown solid (56 mg, 95%). The *ee* was determined to be 72 % using chiral HPLC (OD-H, *i*-propanol/*n*-hexane = 15/85, flow rate = 0.5 mL/min, l = 254 nm) t_R = 34.0 (minor), 38.1 (major).

Rf 0.23 (SiO_2, Hex/EtOAc 8:2, v/v).

^{1}H NMR (400 MHz, $CDCl_3$) δ 7.43 (d, *J* = 7.9 Hz, 2H), 7.30 (dt, *J* = 18.7, 9.2 Hz, 4H), 7.16 (d, *J* = 11.6 Hz, 2H), 7.07 – 6.98 (m, 1H), 6.85 (dd, *J* = 10.2, 7.9 Hz, 1H), 6.76 – 6.69 (m, 1H), 6.51 – 6.42 (m, 2H), 6.01 (dd, *J* = 9.5, 4.3 Hz, 1H), 5.19 (s, 1H), 4.52 (t, *J* = 7.4 Hz, 1H), 3.94 (t, *J* = 5.2 Hz, 1H), 3.50 (s, 2H).

^{13}C NMR (101 MHz, $CDCl_3$) δ 151.5, 151.4, 150.7, 150.6, 149.1, 148.9, 148.2, 148.1, 139.4, 137.4, 131.9, 130.6, 128.3, 128.2, 127.5, 127.2, 126.7, 125.9, 122.1, 119.5, 119.0, 117.8, 117.6, 115.4, 115.2, 110.8, 109.8, 56.5, 38.8, 32.7.

^{19}F NMR (377 MHz, $CDCl_3$) δ -138.0, -138.1, -139.0, -139.0.

$[\alpha]^{25}_D$ = +67 (c = 1.00, $CHCl_3$).

IR (ATR neat) ṽ =: 3261, 1503, 1389, 1306, 1153, 1067 cm^{-1}.

HRMS (ESI): *m/z* calculated for $C_{25}H_{19}BrF_2N_2NaO_2S^+$ [M + Na^+] 551.0211; found 551.0213.

***ee* determination:**

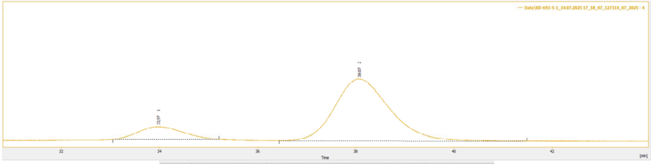

	Reten. Time [min]	Area [mAU.s]	Height [mAU]	Area [%]
1	33,972	2062,220	33,089	14,0
2	38,069	12621,108	163,250	86,0
	Total	14683,328	196,339	100,0

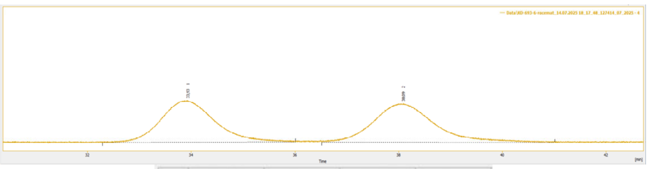

	Reten. Time [min]	Area [mAU.s]	Height [mAU]	Area [%]
1	33,929	3157,830	43,983	48,9
2	38,092	3299,271	40,858	51,1
	Total	6457,101	84,840	100,0

N-((1*S*,2*S*)-2-(1*H*-indol-3-yl)-1,2-dihydronaphthalen-1-yl)-4-bromobenzenesulfonamide (7b)

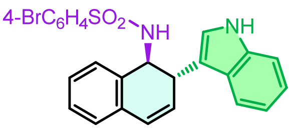

$C_{24}H_{19}BrN_2O_2S$
479,39 g/mol

Following **G. P. E** vial containing **Rh5b** (4 mg, 5 mol%) was charged to glovebox and into reaction vial added ***N*-BsBr** (40 mg, 0.112 mmol, 1 eq.) and indole (66 mg, 0.56 mmol, 5 eq) followed by dry $MeNO_2$ (0.22 mL) reaction mixture was stirred at 80 °C for 16 h. After the reaction was completed, the vial was taken from glovebox and concentrated under reduced pressure, from the crude reaction mixture prepared NMR sample. Column chromatography (SiO_2, Hex/EtOAc 8:2, v/v) of the crude mixture gave **7b** as a brown oil (42 mg, 79%). The *ee* was determinated to be 79 % using chiral HPLC (OD-H, *i*-propanol/*n*-hexane = 15/85, flow rate = 1.0 mL/min, l = 254 nm) t_R = 21.3 (minor), 31.1 (major).

Rf 0.78 (SiO_2, Hex/EtOAc 8:2, v/v).

^{1}H NMR (400 MHz, $CDCl_3$) δ 7.73 (s, 1H), 7.57 (d, *J* = 7.7 Hz, 1H), 7.43 (d, *J* = 8.3 Hz, 2H), 7.33 (d, *J* = 8.4 Hz, 2H), 7.19 – 7.09 (m, 3H), 7.08 – 7.01 (m, 2H), 6.94 – 6.90 (m, 1H), 6.64 (d, *J* = 7.6 Hz,

1H), 6.57 (d, J = 9.6 Hz, 1H), 6.53 (d, J = 2.4 Hz, 1H), 6.00 (dd, J = 9.6, 5.0 Hz, 1H), 5.05 (dd, J = 8.0, 2.6 Hz, 1H), 4.54 (dd, J = 8.1, 4.9 Hz, 1H), 4.08 (t, J = 5.0 Hz, 1H).

^{13}C NMR (101 MHz, $CDCl_3$) δ 139.8, 136.6, 132.7, 132.6, 132.1, 129.4, 128.8, 128.5, 128.5, 128.4, 128.0, 127.4, 127.3, 126.7, 126.4, 122.7, 122.3, 119.8, 119.1, 112.7, 111.5, 56.8, 39.2.

$[\alpha]^{25}_D$ = +69 (c = 1.00, $CHCl_3$).

IR (ATR neat) ṽ =: 3405, 3386, 3269, 3057, 3035, 2922, 2854, 1714, 1573, 1090, 1067 cm^{-1}.

HRMS (ESI): *m/z* calculated for $C_{24}H_{19}BrN_2NaO_2S^+$ [M + Na^+] 501.0243; found 501.0243.

***ee* determination:**

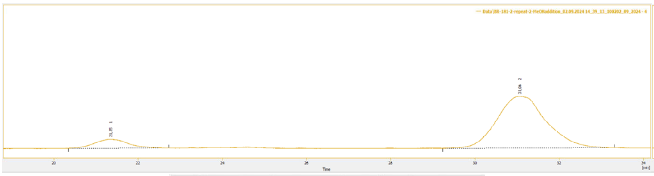

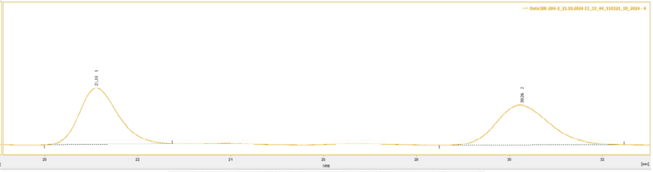

	Reten. Time [min]	Area [mAU.s]	Height [mAU]	Area [%]
1	21,347	502,256	8,839	10,7
2	31,058	4171,210	51,557	89,3
	Total	4673,466	60,397	100,0

	Reten. Time [min]	Area [mAU.s]	Height [mAU]	Area [%]
1	21,104	8502,569	147,157	50,3
2	30,263	8402,798	104,333	49,7
	Total	16905,367	251,490	100,0

4-bromo-*N*-((1*S*,2*S*)-2-(2-methyl-1*H*-indol-3-yl)-1,2-dihydronaphthalen-1-yl) benzenesulfonamide (7c)

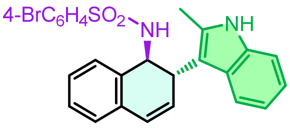

$C_{25}H_{21}BrN_2O_2S$
493,42 g/mol

Following **G. P. E** vial containing **Rh5b** (4 mg, 5 mol%) was charged to glovebox and into reaction vial added ***N*-BsBr** (40 mg, 0.112 mmol, 1 eq) and 2-methyl-1*H*-indole (73.5 mg, 0.56 mmol, 5 eq.) followed by dry $MeNO_2$ (0.22 mL) reaction mixture was stirred at 80 °C for 16 h. After the reaction was completed, the vial was taken from glovebox and concentrated under reduced pressure, from the crude reaction mixture prepared NMR sample. Column chromatography (SiO_2, Hex/EtOAc 8:2, v/v) of the crude mixture gave **7c** as a brown solid (50 mg, 90%). The *ee* was determined to be 72 % using chiral HPLC (OD-H, *i*-propanol/*n*-hexane = 15/85, flow rate = 1.0 mL/min, l = 254 nm) t_R = 15.1 (minor), 23.6 (major).

Rf 0.30 (SiO_2, petroleum ether/EtOAc 1:1, v/v).

^{1}H NMR (400 MHz, Acetone) δ 9.63 (s, 1H), 7.59 (dd, 1H), 7.42 – 7.34 (m, 1H), 7.32 – 7.26 (m, 2H), 7.22 – 7.14 (m, 4H), 7.08 (d, 2H), 6.99 (t, J = 7.9, 0.9 Hz, 1H), 6.92 – 6.86 (m, 2H), 6.63 (dd, J = 9.7, 2.9 Hz, 1H), 5.94 (dd, J = 9.7, 2.8 Hz, 1H), 5.10 – 5.00 (m, 1H), 4.00 (dt, J = 12.8, 2.9 Hz, 1H), 2.27 (s, 3H).

^{13}C NMR (101 MHz, Acetone) δ 141.8, 137.2, 136.5, 134.8, 133.6, 133.4, 131.9, 128.6, 128.4, 128.1, 127.8, 127.4, 127.0, 126.2, 120.9, 119.4, 119.1, 111.6, 110.6, 58.9, 39.5, 12.0.

$[\alpha]^{25}_D$ = +67 (c = 0.5, $CHCl_3$).

IR (ATR neat) ṽ =: 3358, 3072, 2955, 1946, 1693, 1595, 1507, 1491, 1338, 1316 cm^{-1}.

HRMS (ESI): *m/z* calculated for $C_{25}H_{21}BrN_2NaO_2S^+$ [M + Na^+] 515.0399; found 515.0398.

***ee* determination:**

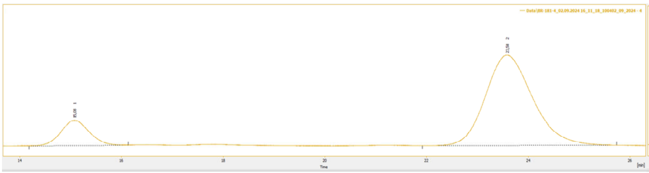

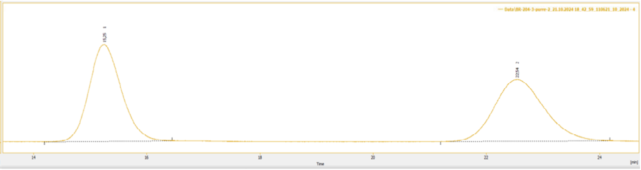

	Reten. Time [min]	Area [mAU.s]	Height [mAU]	Area [%]
1	15,079	1063,508	27,662	14,2
2	23,581	6447,001	100,363	85,8
	Total	7510,509	128,025	100,0

	Reten. Time [min]	Area [mAU.s]	Height [mAU]	Area [%]
1	15,250	2830,736	71,823	50,3
2	22,544	2801,213	45,856	49,7
	Total	5631,949	117,679	100,0

4-bromo-*N*-((1*S*,2*R*)-2-(3-methyl-1*H*-indol-2-yl)-1,2-dihydronaphthalen-1-yl) benzenesulfonamide (7d)

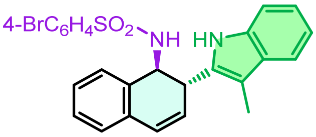

$C_{25}H_{21}BrN_2O_2S$
493,42 g/mol

Following **G. P. E** vial containing **Rh5b** (4 mg, 5 mol%) was charged to glovebox and into reaction vial added ***N*-BsBr** (40 mg, 0.112 mmol, 1 eq.) and 3-methyl-1*H*-indole (73 mg, 0.56 mmol, 5 eq.) followed by dry $MeNO_2$ (0.22 mL) reaction mixture was stirred at 80 °C for 16 h. After the reaction was completed, the vial was taken from glovebox and concentrated under reduced pressure, from the crude reaction mixture prepared NMR sample. Column chromatography (SiO_2, petroleum ether /EtOAc 15:1, followed by 12:1, followed by 9:1 v/v) of the crude mixture gave **7d** as a brown oil (16 mg, 37%). The *ee* was determined to be 82 % using chiral HPLC (OD-H, *i*-propanol/*n*-hexane = 15/85, flow rate = 1.0 mL/min, l = 254 nm) t_R = 18.6 (minor), 26.8 (major).

Rf 0.34 (SiO_2, petroleum ether/EtOAc 8:2, v/v).

^{1}H NMR (600 MHz, $CDCl_3$) δ 7.55 – 7.48 (m, 2H), 7.47 – 7.37 (m, 4H), 7.31 (td, *J* = 7.5, 1.1 Hz, 1H), 7.21 (d, *J* = 7.4 Hz, 1H), 7.13 (dt, *J* = 7.4, 2.1, 1.3 Hz, 1H), 7.06 (dddd, *J* = 9.2, 6.6, 3.8, 1.4 Hz, 3H), 6.85 (t, *J* = 6.3 Hz, 1H), 6.76 (d, *J* = 9.6 Hz, 1H), 6.05 (dd, *J* = 9.5, 5.0 Hz, 1H), 5.05 (t, *J* = 6.6 Hz, 1H), 4.55 (dd, *J* = 8.0, 5.2 Hz, 1H), 4.23 (dt, *J* = 5.0, 2.5 Hz, 1H), 2.25 (s, 3H).

^{13}C NMR (151 MHz, $CDCl_3$) δ 139.6, 135.0, 132.3, 132.2, 131.0, 129.4, 129.2, 128.9, 128.8, 128.5, 128.3, 127.7, 127.5, 127.2, 122.0, 119.5, 118.6, 110.6, 108.8, 56.9, 39.6, 8.6.

$[\alpha]^{25}_D$ = +113 (c = 0.33, $CHCl_3$).

IR (ATR neat) ṽ =: 3390, 3278, 3054, 2919, 2852, 1573, 1458, 1388, 1318, 1152 cm^{-1}.

HRMS (ESI): *m/z* calculated for $C_{25}H_{21}BrN_2NaO_2S^+$ [M + Na^+] 515.0399; found 515.0399

***ee* determination:**

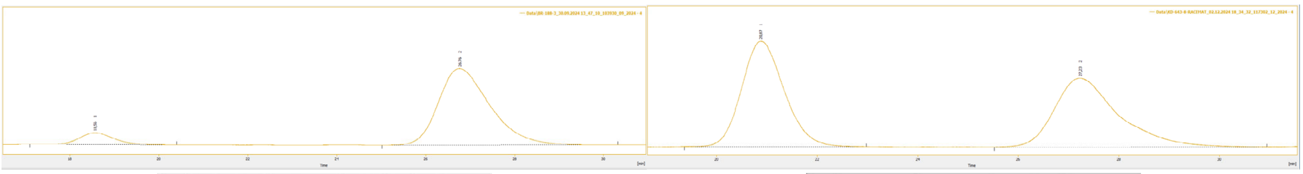

	Reten. Time [min]	Area [mAU.s]	Height [mAU]	Area [%]
1	18,561	2384,933	44,699	9,0
2	26,760	23996,546	298,872	91,0
	Total	26381,479	343,571	100,0

	Reten. Time [min]	Area [mAU.s]	Height [mAU]	Area [%]
1	20,871	7334,976	123,983	50,4
2	27,226	7224,956	81,286	49,6
	Total	14559,932	205,269	100,0

4-bromo-*N*-((1*S*,2*S*)-2-(5-bromo-1*H*-indol-3-yl)-1,2-dihydronaphthalen-1-yl) benzenesulfonamide (7ea)

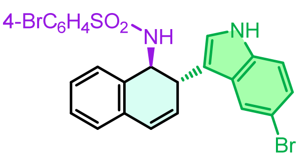

$C_{24}H_{18}Br_2N_2O_2S$
558,29 g/mol

Following **G. P. E** vial containing **Rh5b** (4 mg, 5 mol%) was charged to glovebox and into reaction vial added ***N*-BsBr** (40 mg, 0.112 mmol, 1 eq.) and 5-bromo-1*H*-indole (110 µL, 0.56 mmol, 5 eq.) followed by dry $MeNO_2$ (0.22 mL) reaction mixture was stirred at 80 °C for 16 h. After the reaction was completed, the vial was taken from glovebox and concentrated under reduced pressure, from the crude reaction mixture prepared NMR sample. Column chromatography (SiO_2, petroleum ether/EtOAc 8:2, v/v) of the crude mixture gave **7ea** as a light brown solid (35 mg, 57%). The *ee* was determined to be 75 % using chiral HPLC (OD-H, *i*-propanol/*n*-hexane = 15/85, flow rate = 1.0 mL/min, l = 254 nm) t_R = 22.0 (minor), 27.6 (major).

Rf 0.24 (SiO_2, petroleum ether/EtOAc 8:2, v/v).

^{1}H NMR (400 MHz, $CDCl_3$) δ 7.93 (s, 1H), 7.79 (d, *J* = 7.3 Hz, 1H), 7.58 – 7.50 (m, 2H), 7.50 – 7.41 (m, 2H), 7.33 – 7.18 (m, 2H), 7.11 (d, *J* = 8.5 Hz, 1H), 7.03 (td, *J* = 7.5, 1.1 Hz, 2H), 6.79 (dd, *J* =

15.4, 7.6 Hz, 1H), 6.70 – 6.62 (m, 2H), 6.05 (dd, *J* = 9.5, 4.8 Hz, 1H), 5.14 (t, *J* = 8.8 Hz, 1H), 4.55 (dd, *J* = 7.7, 5.2 Hz, 1H), 4.09 (dd, *J* = 6.4, 2.9 Hz, 1H).

^{13}C NMR (101 MHz, $CDCl_3$) δ 139.5, 135.3, 132.7, 132.5, 132.2, 128.9, 128.5, 128.4, 128.2, 128.1, 127.8, 127.6, 126.8, 125.2, 124.0, 121.8, 113.0, 112.7, 56.6, 38.9.

$[\alpha]^{25}_D$ = -5 (c = 1.00, $CHCl_3$).

IR (ATR neat) ṽ =: 3410, 3270, 2924, 2854, 1573, 1455, 1389, 1317,1152, 1090, 1067 cm^{-1}.

HRMS (ESI): *m/z* calculated for $C_{24}H_{18}Br_2N_2NaO_2S^+$ [M + Na^+] 580.9328; found 580.9334.

***ee* determination:**

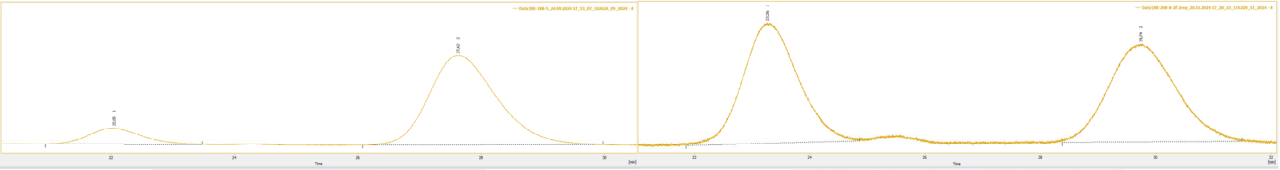

	Reten. Time [min]	Area [mAU.s]	Height [mAU]	Area [%]
1	22,046	946,322	15,838	12,5
2	27,618	6613,242	86,856	87,5
	Total	7559,564	102,695	100,0

	Reten. Time [min]	Area [mAU.s]	Height [mAU]	Area [%]
1	23,264	3026,438	45,601	50,3
2	29,738	2991,710	37,046	49,7
	Total	6018,147	82,647	100,0

4-bromo-*N*-((1*S*,2*S*)-2-(5-fluoro-1*H*-indol-3-yl)-1,2-dihydronaphthalen-1-yl) benzenesulfonamide (7eb)

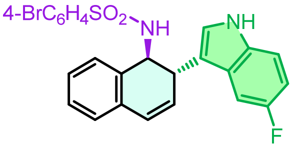

$C_{24}H_{18}BrFN_2O_2S$
497,38 g/mol

Following **G. P. E** vial containing **Rh5b** (4 mg, 5 mol%) was charged to glovebox and into reaction vial added ***N*-BsBr** (40 mg, 0.112 mmol, 1 eq.) and 5-fluoro-1*H*-indole (75.7 mg, 0.56 mmol, 5 eq.) followed by dry $MeNO_2$ (0.22 mL) reaction mixture was stirred at 80 °C for 16 h. After the reaction was completed, the vial was taken from glovebox and concentrated under reduced pressure, from the crude reaction mixture prepared NMR sample. Column chromatography (SiO_2, petroleum ether /EtOAc 1:1, v/v) of the crude mixture gave **7eb** as a light brown solid (49 mg, 89%). The *ee* was determined to be 80 % using chiral HPLC (OD-H, *i*-propanol/*n*-hexane = 15/85, flow rate = 1.0 mL/min, l = 254 nm) t_R = 21.9 (minor), 27.4 (major).

Rf 0.45 (SiO_2, petroleum ether /EtOAc 1:1, v/v).

^{1}H NMR (400 MHz, $CDCl_3$) δ 7.89 (s, 1H), 7.52 (d, *J* = 8.5 Hz, 2H), 7.42 (d, *J* = 8.5 Hz, 2H), 7.33 – 7.26 (m, 1H), 7.26 – 7.18 (m, 1H), 7.18 – 7.07 (m, 2H), 7.02 (t, *J* = 7.4 Hz, 1H), 6.91 (td, *J* = 9.0, 2.1 Hz, 1H), 6.82 – 6.72 (m, 1H), 6.68 – 6.61 (m, 2H), 6.05 (dd, *J* = 9.5, 4.9 Hz, 1H), 5.18 (d, *J* = 7.9 Hz, 1H), 4.56 (dd, *J* = 7.6, 5.3 Hz, 1H), 4.07 (t, *J* = 4.5 Hz, 1H).

^{13}C NMR (101 MHz, $CDCl_3$) δ 159.0, 156.6, 139.6, 133.1, 132.6, 132.4, 132.1, 129.0, 128.8, 128.4, 128.3, 128.1, 127.6, 127.4, 126.7, 126.7, 124.5, 113.0, 112.9, 112.2, 112.1, 110.8, 110.5, 104.1, 103.9, 56.6, 39.1.

^{19}F NMR (377 MHz, $CDCl_3$) δ -123.7.

$[\alpha]^{25}_D$ = +68 (c = 1.00, $CHCl_3$).

IR (ATR neat) ṽ =: 3392, 3279, 3064, 2925, 2853, 1718, 1628, 1574, 1484, 1453 cm^{-1}.

HRMS (ESI): *m/z* calculated for $C_{24}H_{18}BrFN_2NaO_2S^+$ [M + Na^+] 519.0149; found 519.0145.

***ee* determination:**

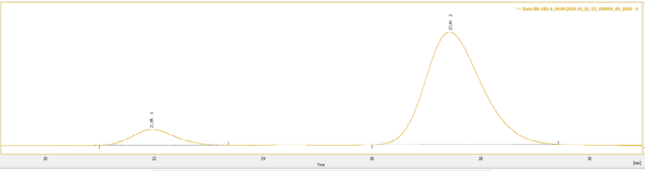

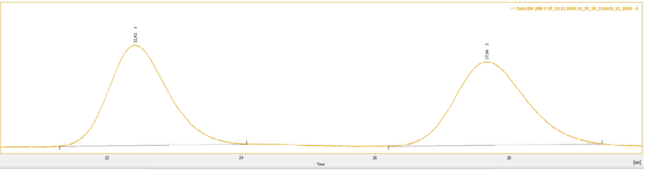

	Reten. Time [min]	Area [mAU.s]	Height [mAU]	Area [%]
1	21,947	2373,366	41,531	10,0
2	27,435	21374,611	297,077	90,0
	Total	23747,978	338,608	100,0

	Reten. Time [min]	Area [mAU.s]	Height [mAU]	Area [%]
1	22,418	2622,722	42,693	49,9
2	27,664	2634,700	35,567	50,1
	Total	5257,422	78,260	100,0

4-bromo-*N*-((1*S*,2*S*)-2-(5-nitro-1*H*-indol-3-yl)-1,2-dihydronaphthalen-1-yl)benzenesulfonamide (7f)

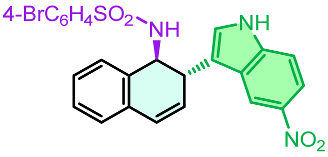

$C_{24}H_{18}BrN_3O_4S$
524,39 g/mol

Following **G. P. E** vial containing **Rh5b** (4 mg, 5 mol%) was charged to glovebox and into reaction vial added ***N*-BsBr** (40 mg, 0.112 mmol, 1 eq.) and 5-nitro-1*H*-indole (90.8 mg, 0.56 mmol, 5 eq.) followed by dry $MeNO_2$ (0.22 mL) reaction mixture was stirred at 80 °C for 16 h. After the reaction was completed, the vial was taken from glovebox and concentrated under reduced pressure, from the crude reaction mixture prepared NMR sample. Column chromatography (SiO_2, petroleum ether/EtOAc 1:1, v/v) of the crude mixture gave **7f** as a yellow solid (29 mg, 50%). The *ee* was determined to be 87 % using chiral HPLC (AD-H, *i*-propanol/*n*-hexane = 15/85, flow rate = 1.0 mL/min, l = 254 nm) t_R = 25.6 (minor), 29.1 (major).

Rf 0.49 (SiO_2, petroleum ether/EtOAc 1:1, v/v).

^{1}H NMR (400 MHz, Acetone) δ 10.73 (s, 1H), 8.88 (d, *J* = 2.1 Hz, 1H), 8.15 (dd, *J* = 9.0, 2.2 Hz, 1H), 7.77 – 7.67 (m, 2H), 7.64 – 7.50 (m, 3H), 7.39 (td, *J* = 7.4, 1.0 Hz, 1H), 7.36 – 7.28 (m, 3H), 7.25 (td, *J* = 7.5, 1.4 Hz, 1H), 7.13 (d, *J* = 7.2 Hz, 1H), 6.88 (dd, *J* = 9.6, 1.6 Hz, 1H), 6.26 (dd, *J* = 9.6, 4.3 Hz, 1H), 4.85 (t, *J* = 7.4 Hz, 1H), 4.41 (d, 1H).

^{13}C NMR (101 MHz, Acetone) δ 141.3, 141.0, 139.9, 133.5, 133.3, 131.7, 129.5, 128.4, 128.1, 127.8, 127.8, 127.0, 126.5, 126.1, 126.0, 116.8, 116.4, 116.0, 112.0, 57.7, 38.8.

$[\alpha]^{25}_D$ = -11 (c = 0.5, $CHCl_3$).

IR (ATR neat) ṽ =: 3379, 3262, 2923, 1621, 1573, 1512, 1469, 1453, 1319, 1155 cm^{-1}.

HRMS (ESI): *m/z* calculated for $C_{24}H_{18}BrN_3NaO_4S^+$ [M + Na^+] 546.0094; found 546.0091.

***ee* determination:**

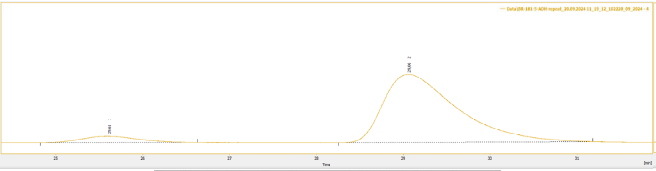

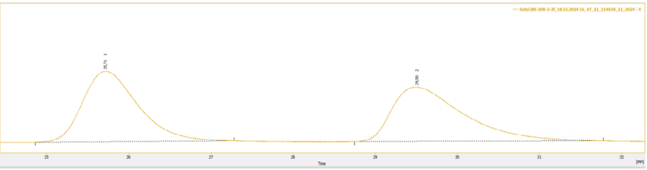

	Reten. Time [min]	Area [mAU.s]	Height [mAU]	Area [%]
1	25,614	369,446	8,132	6,6
2	29,064	5232,954	89,489	93,4
	Total	5602,400	97,621	100,0

	Reten. Time [min]	Area [mAU.s]	Height [mAU]	Area [%]
1	25,708	2788,176	59,497	50,0
2	29,503	2789,795	46,020	50,0
	Total	5577,970	105,517	100,0

(3-((1*S*,2*S*)-1-((4-bromophenyl)sulfonamido)-1,2-dihydronaphthalen-2-yl)-1*H*-indol-5-yl) boronic acid (7g)

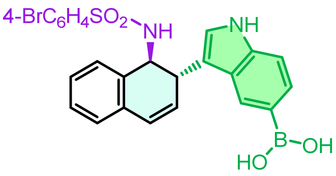

$C_{24}H_{20}BBrN_2O_4S$
523,21 g/mol

Following **G. P. E** vial containing **Rh5b** (4 mg, 5 mol%) was charged to glovebox and into reaction vial added ***N*-BsBr** (40 mg, 0.112 mmol, 1 eq.) and (1*H*-indol-5-yl)boronic acid (90 mg, 0.56 mmol, 5 eq.) followed by dry $MeNO_2$ (0.22 mL) reaction mixture was stirred at 80 °C for 16 h. After the reaction was completed, the vial was taken from glovebox and concentrated under reduced pressure, from the crude reaction mixture prepared NMR sample. Column chromatography (SiO_2, petroleum ether/EtOAc 7:3, v/v) of the crude mixture gave **7g** as a white solid (27 mg, 46%). The *ee* was determined to be 83% using chiral HPLC (OD-H, *i*-propanol/*n*-hexane = 15/85, flow rate = 1.0 mL/min, l = 254 nm) t_R = 21.1 (minor), 29.8 (major).

Rf 0.32 (SiO_2, petroleum ether/EtOAc 7:3, v/v).

^{1}H NMR (400 MHz, $CDCl_3$) δ 7.79 (s, 1H), 7.67 (d, *J* = 7.7 Hz, 1H), 7.59 – 7.50 (m, 2H), 7.49 – 7.40 (m, 2H), 7.32 – 7.18 (m, 3H), 7.18 – 7.10 (m, 2H), 7.02 (td, *J* = 7.5, 1.3 Hz, 1H), 6.69 (dd, *J* = 16.8, 8.4 Hz, 2H), 6.64 (d, *J* = 2.2 Hz, 1H), 6.11 (dd, *J* = 9.5, 5.0 Hz, 1H), 5.08 (d, *J* = 8.0 Hz, 1H), 4.96 (s, 1H), 4.62 (dd, *J* = 8.0, 4.7 Hz, 1H), 4.19 (t, *J* = 4.8 Hz, 1H).

^{13}C NMR (101 MHz, $CDCl_3$) δ 139.7, 136.5, 132.6, 132.5, 132.4, 132.0, 129.2, 128.7, 128.4, 128.4, 128.1, 128.0, 127.4, 127.3, 126.6, 126.4, 122.5, 122.3, 119.8, 119.0, 112.7, 111.4, 56.7, 39.1.

$[\alpha]^{25}_D$ = +33 (c = 0.5, $CHCl_3$).

IR (ATR neat) ṽ =: 3370, 3266, 3058, 2923, 2853, 1574, 1486, 1470 cm^{-1}.

HRMS (ESI): *m/z* calculated for $C_{24}H_{18}BrN_2NaO_2S^+$ [M – $B(OH)_2$ + Na^+] 501.0243; found 501.0243.

***ee* determination:**

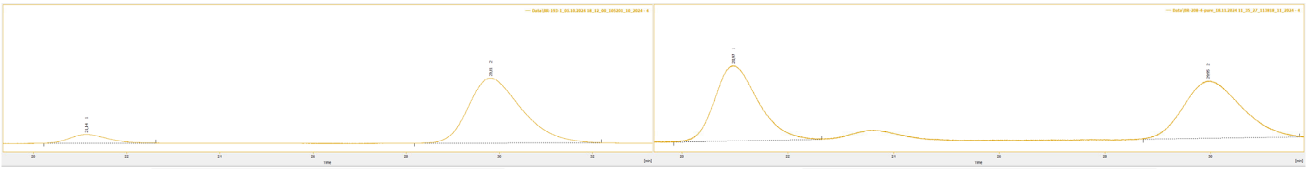

	Reten. Time [min]	Area [mAU.s]	Height [mAU]	Area [%]
1	21,137	1204,411	21,720	8,5
2	29,811	12946,834	163,590	91,5
	Total	14151,245	185,310	100,0

	Reten. Time [min]	Area [mAU.s]	Height [mAU]	Area [%]
1	20,967	548,201	9,621	49,9
2	29,948	551,451	7,280	50,1
	Total	1099,652	16,902	100,0

4-bromo-*N*-((1*S*,2*S*)-2-(5-methoxy-1*H*-indol-3-yl)-1,2-dihydronaphthalen-1-yl) benzenesulfonamide (7h)

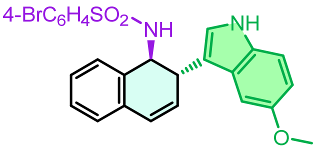

$C_{25}H_{21}BrN_2O_3S$
509,42 g/mol

Following **G. P. E** vial containing **Rh5b** (4 mg, 5 mol%) was charged to glovebox and into reaction vial added ***N*-BsBr** (40 mg, 0.112 mmol, 1 eq.) and 5-methoxy-1*H*-indole (82 mg, 0.56 mmol, 5 eq.) followed by dry $MeNO_2$ (0.22 mL) reaction mixture was stirred at 80 °C for 16 h. After the reaction was completed, the vial was taken from glovebox and concentrated under reduced pressure, from the crude reaction mixture prepared NMR sample. Column chromatography (SiO_2, Hex/EtOAc 8:2, v/v) of the crude mixture gave **7h** as a light brown solid (45 mg, 80%). The *ee* was determinated to be 77 % using chiral HPLC (OD-H, *i*-propanol/*n*-hexane = 15/85, flow rate = 1.0 mL/min, l = 254 nm) t_R = 22.1 (minor), 31.2 (major).

Rf 0.22 (SiO_2, Hex/EtOAc 8:2, v/v).

^{1}H NMR (400 MHz, $CDCl_3$) δ 7.67 (s, 1H), 7.44 (d, *J* = 8.6 Hz, 2H), 7.35 (d, *J* = 8.7 Hz, 2H), 7.21 – 6.99 (m, 4H), 6.95 – 6.85 (m, 1H), 6.75 (dd, *J* = 8.8, 2.3 Hz, 1H), 6.58 (d, *J* = 9.5 Hz, 1H), 6.51 (d, *J*

= 7.7 Hz, 2H), 6.01 (dd, *J* = 9.5, 5.1 Hz, 1H), 5.03 (d, *J* = 8.0 Hz, 1H), 4.54 (dd, *J* = 7.9, 4.2 Hz, 1H), 4.11 (t, *J* = 4.6 Hz, 1H), 3.81 (s, 3H).

^{13}C NMR (101 MHz, $CDCl_3$) δ 154.2, 139.9, 132.7, 132.3, 132.1, 131.7, 129.1, 128.8, 128.5, 128.4, 127.9, 127.3, 126.8, 126.6, 123.2, 112.7, 112.3, 112.2, 100.6, 56.5, 56.0, 39.5.

$[\alpha]^{25}_D$ = +44 (c = 1.00, $CHCl_3$).

IR (ATR neat) ṽ =: 3400, 3279, 3265, 2924, 1715, 1623, 1601, 1483, 1319, 1153 cm^{-1}.

HRMS (ESI): *m/z* calculated for $C_{25}H_{21}BrN_2NaO_3S^+$ [M + Na$^+$] 531.0348; found 531.0348.

***ee* determination:**

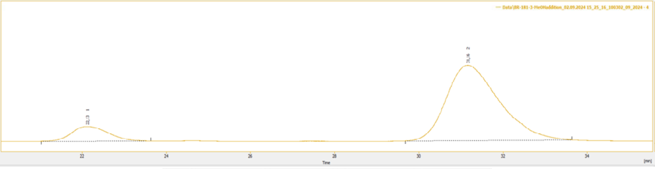

	Reten. Time [min]	Area [mAU.s]	Height [mAU]	Area [%]
1	22,125	2674,141	44,444	11,7
2	31,159	20222,212	233,064	88,3
	Total	22896,354	277,507	100,0

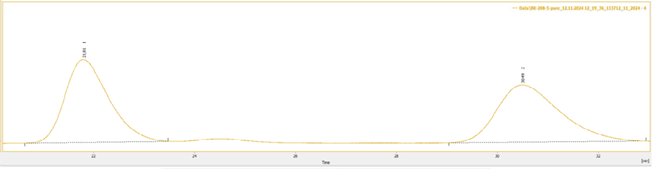

	Reten. Time [min]	Area [mAU.s]	Height [mAU]	Area [%]
1	21,812	3929,894	65,468	50,1
2	30,494	3911,794	45,086	49,9
	Total	7841,687	110,554	100,0

3-((1*S*,2*S*)-1-((4-bromophenyl)sulfonamido)-1,2-dihydronaphthalen-2-yl)-1*H*-indol-4-yl acetate (7i)

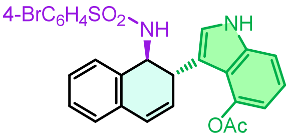

$C_{26}H_{21}BrN_2O_4S$
537,43 g/mol

Following **G. P. E** vial containing **Rh5b** (4 mg, 5 mol%) was charged to glovebox and into reaction vial added ***N*-BsBr** (40 mg, 0.112 mmol, 1 eq.) and 1*H*-indol-4-yl acetate (98 mg, 0.56 mmol, 5 eq.) followed by dry $MeNO_2$ (0.22 mL) reaction mixture was stirred at 80 °C for 16 h. After the reaction was completed, the vial was taken from glovebox and concentrated under reduced pressure, from the crude reaction mixture prepared NMR sample. Column chromatography (SiO_2, Hex/EtOAc 8:2, v/v) of the crude mixture gave **7i** as a white solid (20 mg, 34%). The *ee* was determinated to be 76 % using chiral HPLC (OD-H, *i*-propanol/*n*-hexane = 15/85, flow rate = 1.0 mL/min, l = 254 nm) t_R = 37.8 (minor), 57.6 (major).

Rf 0.34 (SiO_2, Hex/EtOAc 8:2, v/v).

^{1}H NMR (400 MHz, $CDCl_3$) δ 7.96 (s, 1H), 7.25 – 7.05 (m, 9H), 7.02 (t, *J* = 7.4 Hz, 1H), 6.84 (dd, *J* = 7.5, 0.9 Hz, 1H), 6.59 – 6.52 (m, 2H), 6.00 (dd, *J* = 9.6, 4.0 Hz, 1H), 5.37 (d, *J* = 8.7 Hz, 1H), 4.82 (t, *J* = 8.4 Hz, 1H), 4.07 – 3.97 (m, 1H), 2.37 (s, 3H).

^{13}C NMR (101 MHz, $CDCl_3$) δ 170.3, 143.6, 139.6, 138.7, 133.8, 132.5, 131.5, 130.6, 128.4, 128.0, 127.7, 127.5, 126.7, 126.5, 123.6, 122.5, 118.8, 113.2, 112.3, 109.6, 58.4, 39.7, 21.9.

$[\alpha]^{25}_D$ = +54 (c = 0.5, $CHCl_3$).

IR (ATR neat) ṽ =: 3371, 3104, 2921, 2850, 1736, 1623, 1578, 1497, 1423, 1348, 1218, 1192 cm^{-1}.

HRMS (ESI): *m/z* calculated for $C_{26}H_{21}BrN_2NaO_4S^+$ [M + Na$^+$] 559.0298; found 559.0297

***ee* determination:**

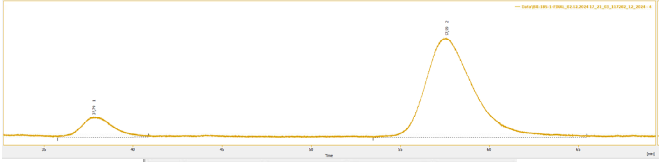

	Reten. Time [min]	Area [mAU.s]	Height [mAU]	Area [%]
1	37,787	1155,734	9,306	12,1
2	57,588	8394,312	44,912	87,9
	Total	9550,046	54,218	100,0

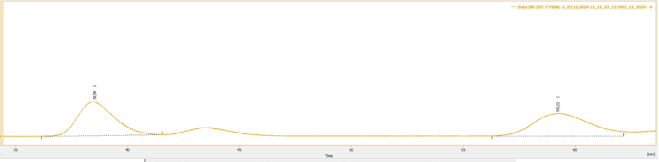

	Reten. Time [min]	Area [mAU.s]	Height [mAU]	Area [%]
1	38,504	4326,548	37,890	49,3
2	59,220	4451,006	25,488	50,7
	Total	8777,554	63,378	100,0

4-bromo-*N*-((1*S*,2*S*)-2-(4-bromo-1*H*-indol-3-yl)-1,2-dihydronaphthalen-1-yl) benzenesulfonamide (7j)

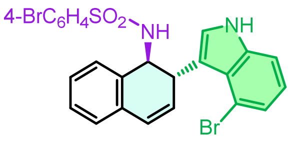

$C_{24}H_{18}Br_2N_2O_2S$
558,29 g/mol

Following **G. P. E** vial containing **Rh5b** (4 mg, 5 mol%) was charged to glovebox and into reaction vial added ***N*-BsBr** (40 mg, 0.112 mmol, 1 eq.) and 4-bromo-1*H*-indole (70 μL, 0.56 mmol, 5 eq.) followed by dry $MeNO_2$ (0.22 mL) reaction mixture was stirred at 80 °C for 16 h. After the reaction was completed, the vial was taken from glovebox and concentrated under reduced pressure, from the crude reaction mixture prepared NMR sample. Column chromatography (SiO_2, petroleum ether/EtOAc 7:3, v/v) of the crude mixture gave **7j** as a light brown oil (61 mg, 99%). The *ee* was determined to be 86 % using chiral HPLC (OD-H, *i*-propanol/*n*-hexane = 15/85, flow rate = 1.0 mL/min, l = 254 nm) t_R = 20.4 (minor), 22.8 (major).

Rf 0.34 (SiO_2, petroleum ether/EtOAc 7:3, v/v).

^{1}H NMR (400 MHz, $CDCl_3$) δ 8.07 (s, 1H), 7.28 – 7.24 (m, 3H), 7.23 – 7.21 (m, 2H), 7.21 – 7.19 (m, 1H), 7.18 – 7.11 (m, 3H), 7.10 – 7.01 (m, 2H), 6.96 (t, *J* = 7.8 Hz, 1H), 6.73 (d, *J* = 2.5 Hz, 1H), 6.56 (d, *J* = 9.5 Hz, 1H), 6.06 (dd, *J* = 9.5, 4.6 Hz, 1H), 5.49 (d, *J* = 8.5 Hz, 1H), 4.83 (d, *J* = 7.0 Hz, 1H).

^{13}C NMR (101 MHz, $CDCl_3$) δ 140.0, 137.4, 132.7, 131.5, 130.7, 128.4, 127.9, 127.9, 127.7, 127.5, 126.6, 126.4, 124.5, 124.5, 124.4, 122.9, 114.8, 113.4, 111.1, 59.5, 37.5.

$[\alpha]^{25}_D$ = +21 (c = 1.00, $CHCl_3$).

IR (ATR neat) ṽ =: 3400, 3355, 3045, 1573, 1471, 1418 cm^{-1}.

HRMS (ESI): *m/z* calculated for $C_{24}H_{18}Br_2N_2NaO_2S^+$ [M + Na$^+$] 580.9328; found 580.9314.

***ee* determination:**

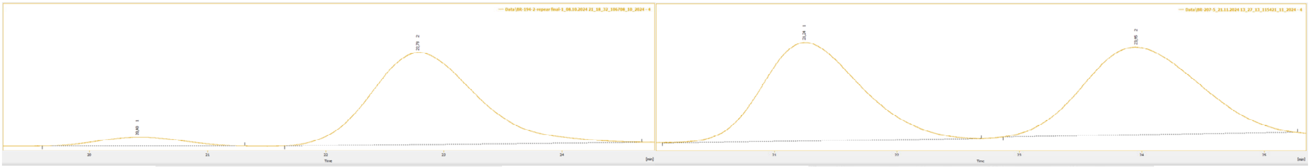

	Reten. Time [min]	Area [mAU.s]	Height [mAU]	Area [%]
1	20,402	533,603	10,813	6,9
2	22,778	7230,742	116,253	93,1
	Total	7764,345	127,066	100,0

	Reten. Time [min]	Area [mAU.s]	Height [mAU]	Area [%]
1	21,241	1950,305	34,724	49,2
2	23,949	2015,650	30,976	50,8
	Total	3965,955	65,700	100,0

4-bromo-*N*-((1*S*,2*S*)-2-(4-methoxy-1*H*-indol-3-yl)-1,2-dihydronaphthalen-1-yl) benzenesulfonamide (7k)

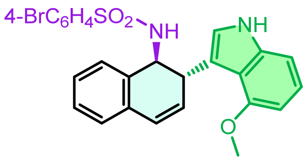

$C_{25}H_{21}BrN_2O_3S$
509,42 g/mol

Following **G. P. E** vial containing **Rh5b** (4 mg, 5 mol%) was charged to glovebox and into reaction vial added ***N*-BsBr** (40 mg, 0.112 mmol, 1 eq.) and 4-methoxy-1*H*-indole (83 mg, 0.56 mmol, 5 eq.) followed by dry $MeNO_2$ (0.22 mL) reaction mixture was stirred at 80 °C for 16 h. After the reaction was completed, the vial was taken from glovebox and concentrated under reduced pressure, from the crude reaction mixture prepared NMR sample. Column chromatography (SiO_2, Hex/EtOAc 8:2, v/v) of the crude mixture gave **7k** as a dark brown oil (38 mg, 68%). The *ee* was determined to be 89 % using chiral HPLC (OD-H, *i*-propanol/*n*-hexane = 15/85, flow rate = 1.0 mL/min, l = 254 nm) t_R = 17.7 (minor), 25.7 (major).

Rf 0.30 (SiO_2, Hex/EtOAc 8:2, v/v).

^{1}H NMR (400 MHz, $CDCl_3$) δ 7.87 (s, 1H), 7.69 – 7.62 (m, 1H), 7.25 (dd, *J* = 5.5, 3.3 Hz, 2H), 7.14 – 7.00 (m, 4H), 6.98 (d, *J* = 8.5 Hz, 2H), 6.88 (d, *J* = 8.2 Hz, 1H), 6.69 – 6.64 (m, 1H), 6.61 – 6.50 (m, 2H), 6.26 (d, *J* = 7.2 Hz, 1H), 6.03 (dd, *J* = 9.6, 2.9 Hz, 1H), 4.60 (dd, *J* = 12.0, 7.3 Hz, 1H), 4.38 (d, *J* = 12.0 Hz, 1H), 3.97 (s, 3H).

^{13}C NMR (101 MHz, $CDCl_3$) δ 153.3, 139.0, 137.5, 135.7, 133.1, 131.9, 130.9, 128.2, 127.9, 127.9, 127.0, 126.1, 126.0, 122.9, 121.2, 117.1, 116.1, 105.5, 100.2, 61.0, 55.4, 38.5.

$[\alpha]^{25}_D$ = +15 (c = 0.33, $CHCl_3$).

IR (ATR neat) ṽ =: 3389, 3257, 2925, 2852, 1573, 1506, 1452, 1325 cm^{-1}.

HRMS (ESI): *m*/*z* calculated for $C_{25}H_{21}BrN_2NaO_3S^+$ [M + Na^+] 531.0348; found 531.0345.

***ee* determination:**

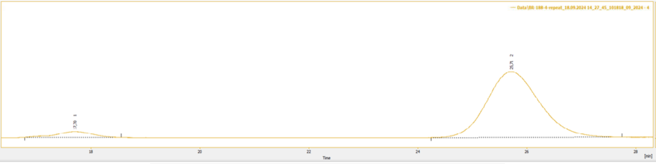

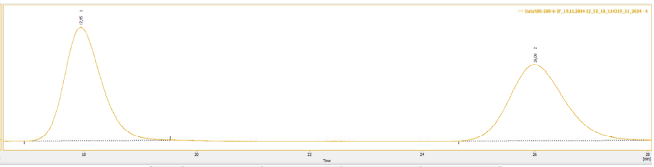

	Reten. Time [min]	Area [mAU.s]	Height [mAU]	Area [%]
1	17,703	552,448	12,137	5,4
2	25,712	9767,668	147,071	94,6
	Total	10320,115	159,208	100,0

	Reten. Time [min]	Area [mAU.s]	Height [mAU]	Area [%]
1	17,948	5961,683	130,733	50,0
2	26,002	5958,376	87,886	50,0
	Total	11920,059	218,619	100,0

4-bromo-*N*-((1*S*,2*S*)-2-(7-bromo-1*H*-indol-3-yl)-1,2-dihydronaphthalen-1-yl) benzenesulfonamide (7l)

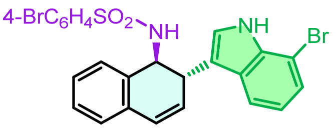

$C_{24}H_{18}Br_2N_2O_2S$
558,29 g/mol

Following **G. P. E** vial containing **Rh5b** (4 mg, 5 mol%) was charged to glovebox and into reaction vial added ***N*-BsBr** (40 mg, 0.112 mmol, 1 eq.) and 7-bromo-1*H*-indole (110 mg, 0.56 mmol, 5 eq.) followed by dry $MeNO_2$ (0.22 mL) reaction mixture was stirred at 80 °C for 16 h. After the reaction was completed, the vial was taken from glovebox and concentrated under reduced pressure, from the crude reaction mixture prepared NMR sample. Column chromatography (SiO_2, petroleum ether/EtOAc 7:3, v/v) of the crude mixture gave **7l** as a colorless oil (54 mg, 88%). The *ee* was determined to be 81 % using chiral HPLC (OD-H, *i*-propanol/*n*-hexane = 15/85, flow rate = 1.0 mL/min, l = 254 nm) t_R = 17.2 (minor), 21.9 (major).

Rf 0.32 (SiO_2, petroleum ether/EtOAc 7:3, v/v).

^{1}H NMR (400 MHz, $CDCl_3$) δ 7.91 (s, 1H), 7.56 (d, *J* = 7.9 Hz, 1H), 7.42 (d, *J* = 8.7 Hz, 2H), 7.36 (d, *J* = 8.7 Hz, 2H), 7.26 (d, *J* = 7.6 Hz, 1H), 7.19 – 7.09 (m, 1H), 7.04 (d, *J* = 7.3 Hz, 1H), 7.00 – 6.88 (m, 2H), 6.73 – 6.55 (m, 3H), 5.99 (dd, *J* = 9.6, 4.9 Hz, 1H), 5.08 (d, *J* = 8.0 Hz, 1H), 4.50 (dd, *J* = 7.8, 5.2 Hz, 1H), 4.06 (t, *J* = 4.8 Hz, 1H).

^{13}C NMR (101 MHz, $CDCl_3$) δ 139.6, 135.2, 132.5, 132.3, 132.1, 128.8, 128.8, 128.4, 128.3, 128.1, 127.7, 127.5, 127.4, 126.7, 124.7, 123.2, 121.0, 118.3, 114.2, 105.0, 56.7, 39.3.

$[\alpha]^{25}_D$ = +106 (c = 1.00, $CHCl_3$).

IR (ATR neat) ṽ =: 3416, 3347, 3283, 3058, 1573, 1433, 1324 cm^{-1}.

HRMS (ESI): *m*/*z* calculated for $C_{24}H_{18}Br_2N_2NaO_2S^+$ [M + Na^+] 580.9328; found 580.9329.

***ee* determination:**

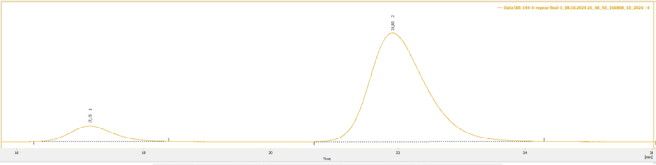

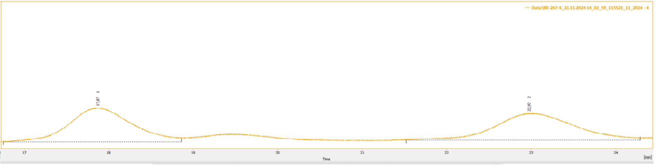

	Reten. Time [min]	Area [mAU.s]	Height [mAU]	Area [%]
1	17,153	3107,095	66,947	9,7
2	21,917	28897,309	473,273	90,3
	Total	32004,404	540,220	100,0

	Reten. Time [min]	Area [mAU.s]	Height [mAU]	Area [%]
1	17,867	266,618	5,121	50,5
2	22,970	261,105	4,006	49,5
	Total	527,723	9,127	100,0

4-methyl-*N*-((1*S*,2*S*)-2-(1-methyl-1*H*-indol-3-yl)-1,2-dihydronaphthalen-1-yl) benzenesulfonamide (7m)

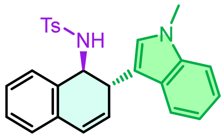

$C_{26}H_{24}N_2O_2S$
428,55 g/mol

Following **G. P. E** vial containing **Rh5b** (4 mg, 5 mol%) was charged to glovebox and into reaction vial added ***N*-Ts** (33.3 mg, 0.112 mmol, 1 eq.), 1-methyl-1*H*-indole (70 µL, 0.56 mmol, 5 eq.) and ZnI_2 (7 mg, 0.022 mmol, 20 mol%) followed by dry THF (0.22 mL) reaction mixture was stirred at 80 °C for 16 h. After the reaction was completed, the vial was taken from glovebox and concentrated under reduced pressure, from the crude reaction mixture prepared NMR sample. Column chromatography (SiO_2, Hex/EtOAc 8:2, v/v) of the crude mixture gave **7m** as a dark brown oil (21 mg, 44%). The *ee* was determined to be 65 % using chiral HPLC (OD-H, *i*-propanol/*n*-hexane = 10/90, flow rate = 0.5 mL/min, l = 254 nm) t_R = 43.9 (minor), 54.0 (major).

Rf 0.35 (SiO_2, Hex/EtOAc 8:2, v/v).

^{1}H NMR (400 MHz, $CDCl_3$) δ 7.62 (t, *J* = 8.4 Hz, 3H), 7.16 – 7.09 (m, 5H), 7.08 – 7.03 (m, 2H), 6.89 (td, *J* = 7.5, 1.4 Hz, 1H), 6.60 (d, *J* = 9.5 Hz, 1H), 6.48 (d, *J* = 7.4 Hz, 1H), 6.38 (s, 1H), 6.04 (dd, *J* = 9.6, 5.4 Hz, 1H), 4.83 (d, *J* = 7.7 Hz, 1H), 4.49 (dd, *J* = 7.6, 2.9 Hz, 1H), 4.19 (d, *J* = 8.5 Hz, 1H), 3.45 (s, 3H), 2.33 (s, 3H).

^{13}C NMR (101 MHz, $CDCl_3$) δ 143.3, 138.0, 137.4, 132.6, 132.5, 129.7, 129.3, 129.0, 128.8, 128.0, 127.4, 127.2, 127.1, 127.1, 126.7, 121.8, 119.4, 119.3, 111.0, 109.3, 56.5, 39.0, 32.7, 21.7.

$[\alpha]^{25}_D$ = +56 (c = 0.5, $CHCl_3$).

IR (ATR neat) ṽ =: 3514, 3280, 3038, 2967, 2924, 1597, 1466, 1452, 1422, 1373 cm^{-1}.

HRMS (ESI): *m/z* calculated for $C_{26}H_{24}N_2NaO_2S^+$ [M + Na^+] 451.1451; found 451.1454.

***ee* determination:**

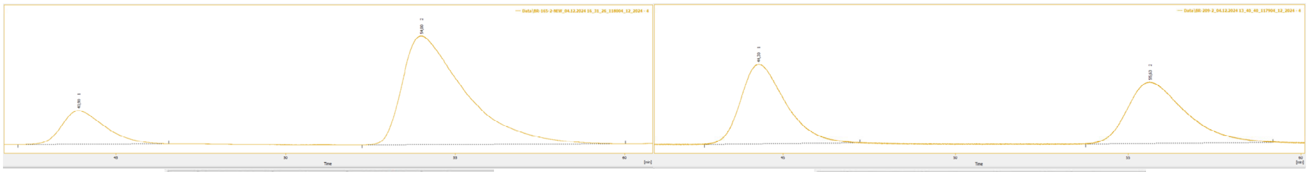

	Reten. Time [min]	Area [mAU.s]	Height [mAU]	Area [%]
1	43,903	21664,617	244,635	17,6
2	54,003	101401,055	788,526	82,4
	Total	123065,672	1033,161	100,0

	Reten. Time [min]	Area [mAU.s]	Height [mAU]	Area [%]
1	44,295	4453,816	49,389	50,1
2	55,629	4433,027	38,074	49,9
	Total	8886,843	87,463	100,0

4-fluoro-*N*-((1*S*,2*S*)-2-(1-methyl-1*H*-indol-3-yl)-1,2-dihydronaphthalen-1-yl) benzenesulfonamide (7n)

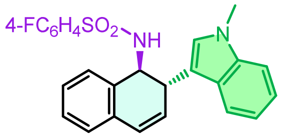

$C_{25}H_{21}FN_2O_2S$
432,51 g/mol

Following **G. P. E** vial containing **Rh5b** (2 mg, 5 mol%) was charged to glovebox and into reaction vial added ***N*-BsF** (17 mg, 0.056 mmol, 1 eq.), and 1-methyl-1*H*-indole (37 µL, 0.28 mmol, 5 eq.) followed by dry THF (0.12 mL) reaction mixture was stirred at 80 °C for 16 h. After the reaction was completed, the vial was taken from glovebox and concentrated under reduced pressure, from the crude reaction mixture prepared NMR sample. Column chromatography (SiO_2, Hex/EtOAc 8:2, v/v) of the crude mixture gave **7n** as a light brown oil (25 mg, 99%). The *ee* was determined to be 66 % using chiral HPLC (OD-H, *i*-propanol/*n*-hexane = 15/85, flow rate = 1.0 mL/min, l = 254 nm) t_R = 14.6 (minor), 18.9 (major).

Rf 0.24 (SiO_2, Hex/EtOAc 8:2, v/v).

^{1}H NMR (400 MHz, $CDCl_3$) δ 7.77 – 7.68 (m, 3H), 7.27 – 7.19 (m, 3H), 7.17 – 7.11 (m, 2H), 7.07 – 6.96 (m, 3H), 6.72 – 6.64 (m, 2H), 6.50 (s, 1H), 6.12 (ddd, *J* = 9.5, 5.1, 0.9 Hz, 1H), 5.05 – 4.96 (m, 1H), 4.62 (dd, *J* = 8.1, 4.3 Hz, 1H), 4.23 (tt, *J* = 5.1, 1.0 Hz, 1H), 3.56 (s, 3H).

^{13}C NMR (101 MHz, $CDCl_3$) δ 166.2, 163.7, 137.4, 137.0, 136.9, 132.7, 132.6, 129.8, 129.7, 129.5, 128.8, 128.6, 128.0, 127.3, 127.0, 126.7, 121.9, 119.4, 119.2, 116.2, 115.9, 111.2, 109.5, 56.9, 39.2, 32.7.

^{19}F NMR (377 MHz, $CDCl_3$) δ -106.1

$[\alpha]^{25}_D$ = +56 (c = 1.00, $CHCl_3$).

IR (ATR neat) ṽ =: 3327, 3250, 2918, 1590, 1492, 1453, 1417, 1374 cm^{-1}.

HRMS (ESI): *m/z* calculated for $C_{25}H_{21}FN_2NaO_2S^+$ [M + Na^+] 455.1200; found 455.1202.

***ee* determination:**

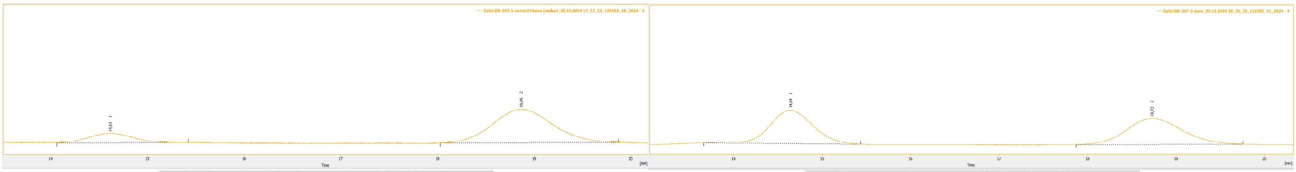

	Reten. Time [min]	Area [mAU.s]	Height [mAU]	Area [%]
1	14,612	222,500	7,099	16,9
2	18,863	1092,191	25,371	83,1
	Total	1314,691	32,470	100,0

	Reten. Time [min]	Area [mAU.s]	Height [mAU]	Area [%]
1	14,643	4078,335	121,328	49,9
2	18,724	4100,977	94,544	50,1
	Total	8179,312	215,872	100,0

4-chloro-*N*-((1*S*,2*S*)-2-(1-methyl-1*H*-indol-3-yl)-1,2-dihydronaphthalen-1-yl) benzenesulfonamide (7o)

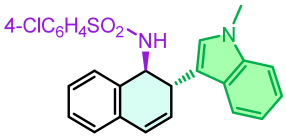

$C_{25}H_{21}ClN_2O_2S$
448,97 g/mol

Following **G. P. E** vial containing **Rh5b** (2 mg, 5 mol%) was charged to glovebox and into reaction vial added ***N*-BsCl** (18 mg, 0.056 mmol, 1 eq.), and 1-methyl-1*H*-indole (37 µL, 0.28 mmol, 5 eq.) followed by dry THF (0.12 mL) reaction mixture was stirred at 80 °C for 16 h. After the reaction was completed, the vial was taken from glovebox and concentrated under reduced pressure, from the crude reaction mixture prepared NMR sample. Column chromatography (SiO_2, Hex/EtOAc 8:2, v/v) of the crude mixture gave **7o** as a light brown solid (15 mg, 60%). The *ee* was determined to be 65 % using chiral HPLC (OD-H, *i*-propanol/*n*-hexane = 15/85, flow rate = 1.0 mL/min, l = 254 nm) t_R = 16.0 (minor), 19.9 (major).

Rf 0.32 (SiO_2, Hex/EtOAc 8:2, v/v).

^{1}H NMR (400 MHz, $CDCl_3$) δ 7.66 (dd, *J* = 7.9, 4.0 Hz, 1H), 7.62 – 7.52 (m, 2H), 7.29 – 7.24 (m, 2H), 7.24 – 7.19 (m, 3H), 7.17 – 7.10 (m, 2H), 7.04 (td, *J* = 7.5, 1.3 Hz, 1H), 6.76 (d, *J* = 7.7 Hz, 1H), 6.66 (d, *J* = 9.6 Hz, 1H), 6.49 (s, 1H), 6.09 (dd, *J* = 9.6, 4.9 Hz, 1H), 5.02 (d, *J* = 7.8 Hz, 1H), 4.63 (dd, *J* = 8.0, 5.1 Hz, 1H), 4.17 (t, *J* = 4.6 Hz, 1H), 3.54 (s, 3H).

^{13}C NMR (^{13}C NMR (101 MHz, $CDCl_3$) δ 139.3, 138.6, 137.3, 132.6, 129.5, 128.9, 128.7, 128.3, 128.0, 127.2, 127.1, 126.9, 126.6, 121.8, 119.3, 119.1, 111.2, 109.4, 57.0, 39.1, 32.6.

$[\alpha]^{25}_D$ = +73 (c = 1.00, $CHCl_3$).

IR (ATR neat) ṽ =: 3280, 3053, 2923, 2853, 1585, 1474 cm^{-1}.

HRMS (ESI): *m/z* calculated for $C_{25}H_{21}ClN_2NaO_2S^+$ [M + Na^+] 471.0904; found 471.0907.

***ee* determination:**

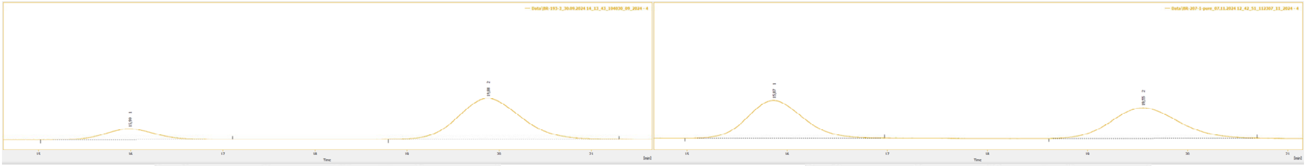

	Reten. Time [min]	Area [mAU.s]	Height [mAU]	Area [%]
1	15,992	947,746	24,692	17,6
2	19,879	4442,801	92,862	82,4
	Total	5390,547	117,553	100,0

	Reten. Time [min]	Area [mAU.s]	Height [mAU]	Area [%]
1	15,871	2412,503	62,444	50,5
2	19,554	2360,721	50,616	49,5
	Total	4773,225	113,060	100,0

Methyl 3-((1*S*,2*S*)-1-((4-bromophenyl)sulfonamido)-1,2-dihydronaphthalen-2-yl)-1*H*-indole-6-carboxylate (7p)

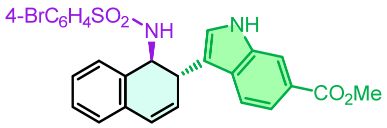

$C_{26}H_{21}BrN_2O_4S$
537,43 g/mol

Following **G. P. E** vial containing **Rh5b** (4 mg, 5 mol%) was charged to glovebox and into reaction vial added ***N*-BsBr** (40 mg, 0.112 mmol, 1 eq.) and methyl 1*H*-indole-6-carboxylate (98 mg, 0.56 mmol, 5 eq.) followed by dry $MeNO_2$ (0.22 mL) reaction mixture was stirred at 80 °C for 16 h. After the reaction was completed, the vial was taken from glovebox and concentrated under reduced pressure, from the crude reaction mixture prepared NMR sample. Column chromatography (SiO_2, petroleum ether/EtOAc 1:1, v/v) of the crude mixture gave **7p** as a light brown solid (53 mg, 89%). The *ee* was determined to be 89 % using chiral HPLC (OD-H, *i*-propanol/*n*-hexane = 15/85, flow rate = 0.5 mL/min, l = 254 nm) t_R = 33.1 (minor), 36.2 (major).

Rf 0.61 (SiO_2, petroleum ether/EtOAc 1:1, v/v).

^{1}H NMR (400 MHz, $CDCl_3$) δ 8.28 (s, 1H), 7.95 (s, 1H), 7.73 (d, *J* = 8.5 Hz, 1H), 7.62 (d, *J* = 8.3 Hz, 1H), 7.44 (d, *J* = 6.7 Hz, 2H), 7.33 (d, *J* = 5.9 Hz, 2H), 7.15 (dd, *J* = 15.3, 7.8 Hz, 1H), 7.04 (d, *J* = 7.5 Hz, 1H), 6.94 (t, *J* = 7.4 Hz, 1H), 6.75 (d, *J* = 2.3 Hz, 1H), 6.61 (dd, *J* = 18.2, 8.5 Hz, 2H), 6.00 (dd, *J* = 9.1, 4.7 Hz, 1H), 5.18 (d, *J* = 7.9 Hz, 1H), 4.51 (dd, *J* = 7.9, 5.1 Hz, 1H), 4.11 (s, 1H), 3.85 (s, 3H).

^{13}C NMR (101 MHz, $CDCl_3$) δ 168.2, 139.6, 135.9, 132.6, 132.3, 132.0, 129.9, 128.9, 128.8, 128.3, 128.2, 128.1, 127.7, 127.3, 126.7, 126.1, 123.9, 120.7, 118.6, 113.9, 113.3, 56.8, 52.1, 39.0.

$[\alpha]^{25}_D$ = +19 (c = 1.00, $CHCl_3$).

IR (ATR neat) ṽ =: 3353, 3245, 2990, 2950, 1689, 1622, 1573, 1500 cm^{-1}.

HRMS (ESI): *m*/*z* calculated for $C_{26}H_{21}BrN_2NaO_4S^+$ [M + Na^+] 559.0298; found 559.0296.

***ee* determination:**

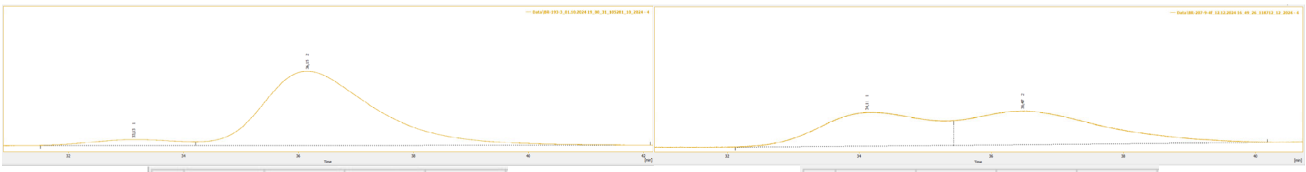

	Reten. Time [min]	Area [mAU.s]	Height [mAU]	Area [%]
1	33,129	1137,111	11,488	5,4
2	36,152	19791,730	146,959	94,6
	Total	20928,841	158,446	100,0

	Reten. Time [min]	Area [mAU.s]	Height [mAU]	Area [%]
1	34,208	3446,331	32,035	50,5
2	36,523	3376,069	25,245	49,5
	Total	6822,400	57,280	100,0

4-bromo-*N*-((1*S*,2*S*)-2-(2,5-dimethyl-1*H*-pyrrol-3-yl)-1,2-dihydronaphthalen-1-yl) benzenesulfonamide (7q)

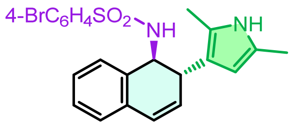

$C_{22}H_{21}BrN_2O_2S$
457,39 g/mol

Following **G. P. E** vial containing **Rh5b** (4 mg, 5 mol%) was charged to glovebox and into reaction vial added ***N*-BsBr** (40 mg, 0.112 mmol, 1 eq.) and 2,5-dimethyl-1*H*-pyrrole (57 µL, 0.56 mmol, 5 eq.) followed by dry $MeNO_2$ (0.22 mL) reaction mixture was stirred at 80 °C for 16 h. After the reaction was completed, the vial was taken from glovebox and concentrated under reduced pressure, from the crude reaction mixture prepared NMR sample. Column chromatography (SiO_2, Hex/EtOAc 8:2, v/v) of the crude mixture gave **7q** as a brown oil (34 mg, 67%). The *ee* was determined to be 66 % using chiral HPLC (OD-H, *i*-propanol/*n*-hexane = 15/85, flow rate = 1.0 mL/min, l = 254 nm) t_R = 12.4 (minor), 17.7 (major).

Rf 0.24 (SiO_2, Hex/EtOAc 8:2, v/v).

^{1}H NMR (400 MHz, $CDCl_3$) δ 7.70 – 7.36 (m, 4H), 7.35 (s, 1H), 7.33 – 7.14 (m, 1H), 7.14 – 6.99 (m, 2H), 6.85 (d, *J* = 7.4 Hz, 1H), 6.53 (d, *J* = 9.6 Hz, 1H), 5.95 (dd, *J* = 9.6, 5.1 Hz, 1H), 5.07 (d, *J* = 2.1 Hz, 1H), 4.94 (d, *J* = 8.0 Hz, 1H), 4.36 (dd, *J* = 7.9, 4.7 Hz, 1H), 3.67 (t, *J* = 4.2 Hz, 1H), 2.10 (s, 3H), 2.00 (s, 3H).

^{13}C NMR (101 MHz, $CDCl_3$) δ 140.2, 133.0, 132.7, 132.0, 130.8, 128.7, 128.6, 128.2, 127.8, 127.1, 126.4, 126.3, 125.1, 122.7, 116.6, 105.4, 58.0, 39.4, 12.9, 11.0.

$[\alpha]^{25}_D$ = +140 (c = 0.33, $CHCl_3$).

IR (ATR neat) ṽ =: 3384, 3267, 3032, 2919, 1573, 1389, 1322, 1153 cm^{-1}.

HRMS (ESI): *m/z* calculated for $C_{22}H_{21}BrN_2NaO_2S^+$ [M + Na^+] 479.0399; found 479.0392.

***ee* determination:**

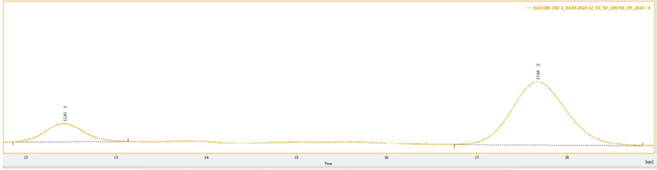

	Reten. Time [min]	Area [mAU.s]	Height [mAU]	Area [%]
1	12,430	156,919	5,199	16,8
2	17,678	776,000	18,157	83,2
	Total	932,919	23,356	100,0

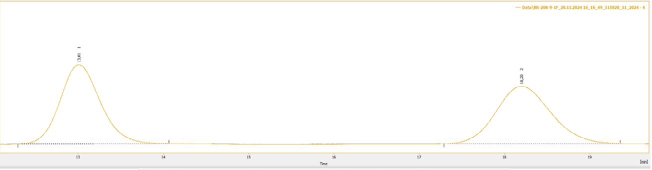

	Reten. Time [min]	Area [mAU.s]	Height [mAU]	Area [%]
1	13,008	5195,348	159,104	50,3
2	18,198	5132,412	115,410	49,7
	Total	10327,760	274,514	100,0

4-bromo-*N*-((1*S*,2*S*)-2-(1,2,5-trimethyl-1*H*-pyrrol-3-yl)-1,2-dihydronaphthalen-1-yl) benzenesulfonamide (7r)

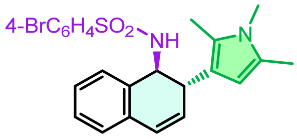

$C_{23}H_{23}BrN_2O_2S$
471,41 g/mol

Following **G. P. E** vial containing **Rh5b** (4 mg, 5 mol%) was charged to glovebox and into reaction vial added ***N*-BsBr** (40 mg, 0.112 mmol, 1 eq.) and 1,2,5-trimethyl-1*H*-pyrrole (66 µL, 0.56 mmol, 5 eq.) followed by dry $MeNO_2$ (0.22 mL) reaction mixture was stirred at 80 °C for 16 h. After the reaction was completed, the vial was taken from glovebox and concentrated under reduced pressure, from the crude reaction mixture prepared NMR sample. Column chromatography (SiO_2, Hex/EtOAc 8:2, v/v) of the crude mixture gave **7r** as a dark brown solid (45 mg, 86%). The *ee* was determined to be 60 % using chiral HPLC (OD-H, *i*-propanol/*n*-hexane = 15/85, flow rate = 1.0 mL/min, l = 254 nm) t_R = 10.1 (minor), 11.9 (major).

Rf 0.43 (SiO_2, Hex/EtOAc 8:2, v/v).

^{1}H NMR (400 MHz, $CDCl_3$) δ 7.65 – 7.46 (m, 4H), 7.29 – 7.19 (m, 1H), 7.08 (d, *J* = 8.5 Hz, 2H), 6.94 – 6.83 (m, 1H), 6.52 (d, *J* = 9.5 Hz, 1H), 5.94 (dd, *J* = 9.5, 4.9 Hz, 1H), 5.13 (s, 1H), 4.95 (d, *J* = 7.9 Hz, 1H), 4.36 (dd, *J* = 8.0, 5.1 Hz, 1H), 3.69 (td, *J* = 5.0, 1.4 Hz, 1H), 3.23 (s, 3H), 2.08 (s, 3H), 1.97 (s, 3H).

^{13}C NMR (101 MHz, $CDCl_3$) δ 140.2, 133.1, 132.9, 131.9, 131.0, 128.7, 128.6, 128.1, 127.8, 127.0, 126.8, 126.4, 126.2, 124.5, 115.5, 104.5, 58.2, 39.6, 30.2, 12.3, 10.1.

$[\alpha]^{25}_D$ = +69 (c = 0.33, $CHCl_3$).

IR (ATR neat) ṽ =: 3289, 2967, 2922, 2855, 1633, 1574, 1525 cm^{-1}.

HRMS (ESI): *m/z* calculated for $C_{23}H_{23}BrN_2NaO_2S^+$ [M + Na^+] 493.0556; found 493.0555.

***ee* determination:**

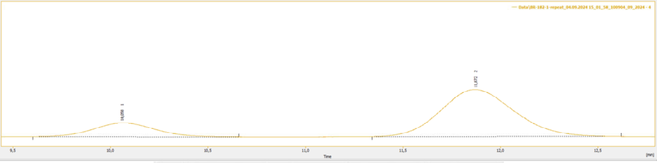

	Reten. Time [min]	Area [mAU.s]	Height [mAU]	Area [%]
1	10,058	627,667	28,385	20,1
2	11,872	2499,229	94,867	79,9
	Total	3126,896	123,252	100,0

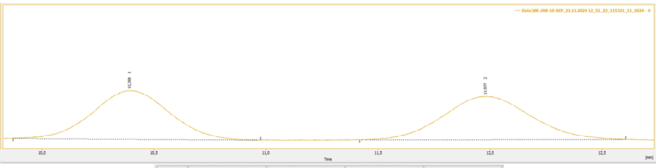

	Reten. Time [min]	Area [mAU.s]	Height [mAU]	Area [%]
1	10,388	455,464	19,145	49,9
2	11,977	458,195	16,968	50,1
	Total	913,659	36,113	100,0

4-bromo-*N*-((1*R*,2*R*)-2-(methyl(phenyl)amino)-1,2-dihydronaphthalen-1-yl) benzenesulfonamide (7s)

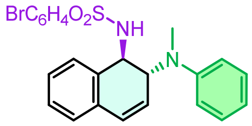

$C_{23}H_{21}BrN_2O_2S$
469,40 g/mol

Following **G. P. E** vial containing **Rh5b** (4 mg, 5 mol%) was charged to glovebox and into reaction vial added ***N*-BsBr** (40 mg, 0.112 mmol, 1 eq) and N-methylaniline (60 µL, 0.56 mmol, 5 eq.) followed by dry THF (0.22 mL) reaction mixture was stirred at 80 °C for 16 h. After the reaction was completed, the vial was taken from glovebox and concentrated under reduced pressure, from the crude reaction mixture prepared NMR sample. Column chromatography (SiO_2, petroleum ether/EtOAc 9:1, v/v) of the crude mixture gave **7s** as a brown solid (40 mg, 76%). The *ee* was determined to be 86 % using chiral HPLC (OD-H, *i*-propanol/*n*-hexane = 10/90, flow rate = 0.5 mL/min, l = 254 nm) t_R = 30.1 (minor), 36.3 (major).

Rf 0.37 (SiO_2, petroleum ether/EtOAc 9:1, v/v).

^{1}H NMR (400 MHz, $CDCl_3$) δ 7.51 (d, *J* = 8.6 Hz, 2H), 7.44 (d, *J* = 8.5 Hz, 2H), 7.29 – 7.17 (m, 3H), 7.18 – 7.07 (m, 2H), 7.05 (t, *J* = 6.4 Hz, 1H), 6.76 (t, *J* = 7.3 Hz, 1H), 6.67 (dd, *J* = 13.8, 9.2 Hz, 3H), 5.82 (dd, *J* = 9.1, 2.8 Hz, 1H), 5.04 – 4.88 (m, 1H), 4.75 – 4.59 (m, 2H), 2.37 (s, 3H).

^{13}C NMR (101 MHz, $CDCl_3$) δ 148.9, 139.8, 133.7, 132.4, 132.2, 130.5, 129.4, 128.9, 128.6, 128.6, 127.6, 127.5, 127.2, 126.5, 117.8, 113.8, 59.6, 55.1, 32.8.

$[\alpha]^{25}_D$ = +81 (c = 1.00, $CHCl_3$).

IR (ATR neat) ṽ =: 3252, 3049, 3023, 2920, 2812, 1595, 1575 cm^{-1}.

HRMS (ESI): *m*/*z* calculated for $C_{23}H_{21}BrN_2NaO_2S^+$ [M + Na^+] 491.0399; found 491.0402

***ee* determination:**

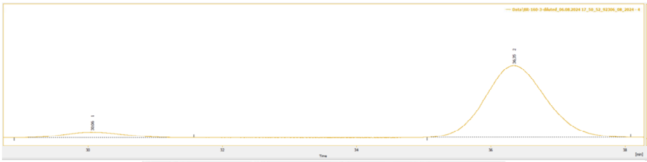

	Reten. Time [min]	Area [mAU.s]	Height [mAU]	Area [%]
1	30,063	1954,269	30,854	6,9
2	36,346	26349,226	420,152	93,1
	Total	28303,495	451,006	100,0

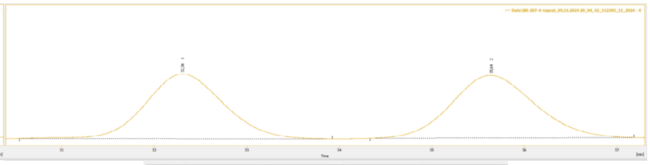

	Reten. Time [min]	Area [mAU.s]	Height [mAU]	Area [%]
1	32,301	20256,340	344,653	49,2
2	35,637	20922,985	333,937	50,8
	Total	41179,324	678,591	100,0

tert-butyl ((1*R*,2*R*)-2-(methyl(phenyl)amino)-1,2-dihydronaphthalen-1-yl)carbamate (7t)

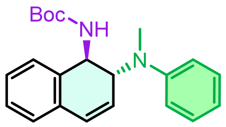

$C_{22}H_{26}N_2O_2$
350,46 g/mol

Following **G. P. E** vial containing **Rh5b** (4 mg, 5 mol%) was charged to glovebox and into reaction vial added ***N*-Boc** (27 mg, 0.112 mmol, 1 eq) and N-methylaniline (60 µL, 0.56 mmol, 5 eq.) followed by dry THF (0.22 mL) reaction mixture was stirred at 80 °C for 16 h. After the reaction was completed, the vial was taken from glovebox and concentrated under reduced pressure, from the crude reaction mixture prepared NMR sample. Column chromatography (SiO_2, Pentan/EtOAc 15:1, v/v) of the crude mixture gave **7t** as a light brown oil (24 mg, 61%). The *ee* was determined to be 79 % using chiral HPLC (AS-H, *i*-propanol/*n*-hexane = 5/95, flow rate = 0.5 mL/min, l = 254 nm) t_R = 12.7 (minor), 14.1 (major).

Rf 0.25 (SiO_2, Pentan/EtOAc 9:1, v/v).

^{1}H NMR (400 MHz, $CDCl_3$) δ 7.33 (d, *J* = 8.5 Hz, 1H), 7.23 (dd, *J* = 7.1, 2.3 Hz, 2H), 7.09 (dd, *J* = 6.5, 2.2 Hz, 1H), 6.84 (d, *J* = 8.2 Hz, 2H), 6.72 (t, *J* = 7.2 Hz, 1H), 6.60 (dd, *J* = 9.6, 2.0 Hz, 1H), 5.92 (dd, *J* = 9.8, 3.0 Hz, 1H), 5.20 (t, *J* = 10.2 Hz, 1H), 4.78 (d, *J* = 10.7 Hz, 1H), 4.50 (d, *J* = 9.6 Hz, 1H), 2.83 (s, 3H), 1.34 (s, 11H).

^{13}C NMR (101 MHz, $CDCl_3$) δ 155.4, 150.2, 135.9, 132.8, 130.1, 129.9, 129.2, 128.0, 127.9, 126.7, 125.8, 117.1, 113.6, 79.5, 60.8, 52.4, 33.1, 28.3.

[α]$^{25}_{D}$ = +60 (c = 1.00, $CHCl_3$).

IR (ATR neat) ṽ =: 3436, 3016, 2978, 2927, 1703, 1597, 1503 cm^{-1}.

HRMS (ESI): *m*/*z* calculated for $C_{22}H_{26}N_2NaO_2^+$ [M + Na^+] 373.1886; found 373.1887.

***ee* determination:**

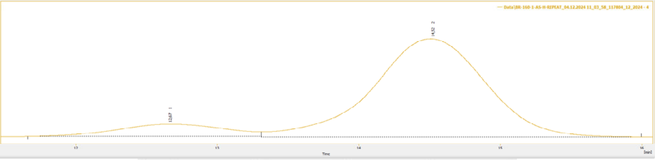

	Reten. Time [min]	Area [mAU.s]	Height [mAU]	Area [%]
1	12,667	4188,782	80,313	10,6
2	14,521	35454,120	642,303	89,4
	Total	39642,902	722,616	100,0

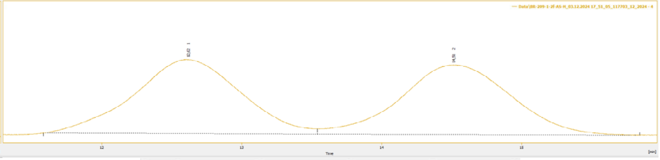

	Reten. Time [min]	Area [mAU.s]	Height [mAU]	Area [%]
1	12,618	2578,385	48,564	50,7
2	14,512	2506,754	45,675	49,3
	Total	5085,139	94,239	100,0

(1*S*,2*S*)-2-(1-methyl-1*H*-indol-3-yl)-1,2-dihydronaphthalen-1-ol (7u)

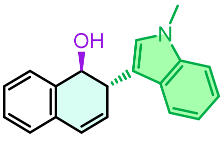

$C_{19}H_{17}NO$
275,35 g/mol

Following **G. P. E** vial containing **Rh5b** (4 mg, 5 mol%) was charged to glovebox and into reaction vial added 1,4-dihydro-1,4-epoxynaphthalene (16.2 mg, 0.112 mmol, 1 eq.) and 1-methyl-1*H*-indole (70 µL, 0.56 mmol, 5 eq.) followed by dry THF (0.22 mL) reaction mixture was stirred at 80 °C for 16 h. After the reaction was completed, the vial was taken from glovebox and concentrated under reduced pressure, from the crude reaction mixture prepared NMR sample. Column chromatography (SiO_2, Hex/EtOAc 8:2, v/v) of the crude mixture gave **7u** as a dark brown oil (23 mg, 73%). The *ee* was determined to be 70 % using chiral HPLC (AS-H, *i*-propanol/*n*-hexane = 10/90, flow rate = 0.5 mL/min, l = 254 nm) t_R = 23.2 (major), 26.1 (minor).

Rf 0.25 (SiO_2, Hex/EtOAc 8:2, v/v).

^{1}H NMR (400 MHz, $CDCl_3$) δ 7.74 (dd, *J* = 8.0, 1.0 Hz, 1H), 7.44 – 7.37 (m, 1H), 7.32 – 7.20 (m, 4H), 7.16 (dd, *J* = 7.2, 1.6 Hz, 1H), 7.12 (ddd, *J* = 8.0, 6.9, 1.2 Hz, 1H), 6.81 (s, 1H), 6.64 (dd, *J* = 9.6, 2.2 Hz, 1H), 6.15 (dd, *J* = 9.6, 3.8 Hz, 1H), 5.00 (d, *J* = 8.1 Hz, 1H), 4.05 (ddd, *J* = 8.2, 3.9, 2.2 Hz, 1H), 3.69 (s, 3H), 2.07 (s, 1H).

^{13}C NMR (101 MHz, $CDCl_3$) δ 137.6, 136.3, 132.7, 130.5, 128.2, 127.9, 127.3, 127.2, 127.1, 126.6, 126.4, 122.0, 119.7, 119.2, 113.0, 109.6, 73.1, 41.4, 32.8.

[α]$^{25}_{D}$ = -80 (c = 1.00, $CHCl_3$).

IR (ATR neat) ṽ =: 3538, 3388, 3029, 2929, 1730, 1613, 1544, 1482, 1471 cm^{-1}.

HRMS (ESI): *m*/*z* calculated for $C_{19}H_{17}NNaO^+$ [M + Na^+] 298.1202; found 298.1204.

***ee* determination:**

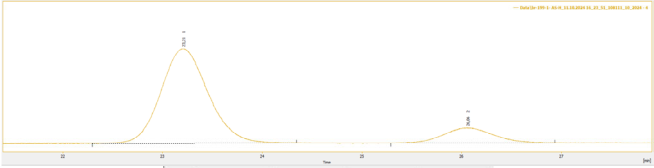

	Reten. Time [min]	Area [mAU.s]	Height [mAU]	Area [%]
1	23,211	4190,971	127,403	85,0
2	26,063	737,975	20,375	15,0
	Total	4928,946	147,778	100,0

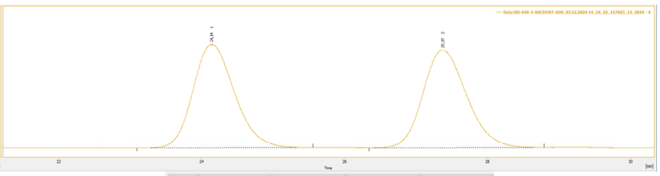

	Reten. Time [min]	Area [mAU.s]	Height [mAU]	Area [%]
1	24,135	29653,413	742,524	50,2
2	27,368	29426,363	699,420	49,8
	Total	59079,776	1441,944	100,0

(1*R*,2*R*)-2-(methyl(phenyl)amino)-1,2-dihydronaphthalen-1-ol (7v)

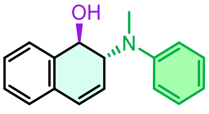

$C_{17}H_{17}NO$
251,33 g/mol

Following **G. P. E** vial containing **Rh5b** (4 mg, 5 mol%) was charged to glovebox and into reaction vial added 1,4-dihydro-1,4-epoxynaphthalene (16.2 mg, 0.112 mmol, 1 eq.) and *N*-methylaniline (58 µL, 0.56 mmol, 5 eq.) followed by dry THF (0.22 mL) reaction mixture was stirred at 80 °C for 16 h. After the reaction was completed, the vial was taken from glovebox and concentrated under reduced pressure, from the crude reaction mixture prepared NMR sample. Column chromatography (SiO_2, PE/EtOAc 10:1, v/v) of the crude mixture gave **7v** as a dark brown oil (23 mg, 82%). The *ee* was determined to be 72 % using chiral HPLC (OD-H, *i*-propanol/*n*-hexane = 10/90, flow rate = 0.5 mL/min, l = 254 nm) t_R = 19.9 (major), 21.7 (minor).

Rf 0.32 (SiO_2, PE/EtOAc 10:1, v/v).

^{1}H NMR (400 MHz, $CDCl_3$) δ 7.54 (dd, *J* = 6.6, 2.5 Hz, 1H), 7.32 – 7.20 (m, 4H), 7.15 – 7.06 (m, 1H), 6.95 (d, *J* = 7.8 Hz, 2H), 6.79 (td, *J* = 7.3, 0.9 Hz, 1H), 6.58 (dd, *J* = 9.8, 2.5 Hz, 1H), 5.92 (dd, *J* = 9.8, 3.0 Hz, 1H), 5.10 (d, *J* = 9.8 Hz, 1H), 4.73 (dt, *J* = 9.8, 2.8 Hz, 1H), 2.84 (s, 3H), 2.34 (s, 1H).

^{13}C NMR (101 MHz, $CDCl_3$) δ 150.4, 136.6, 132.1, 129.8, 129.4, 128.2, 128.1, 127.9, 126.6, 125.7, 118.3, 114.8, 70.2, 63.7, 33.5.

$[\alpha]^{25}_D$ = +35 (c = 1.00, $CHCl_3$).

IR (ATR neat) ṽ =: 3618, 3356, 3056, 3034, 2921, 2859, 2805, 1595 cm^{-1}.

HRMS (ESI): *m/z* calculated for $C_{17}H_{17}NNaO^+$ [M + Na^+] 274.1202; found 274,1206.

***ee* determination:**

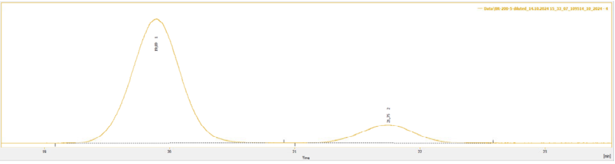

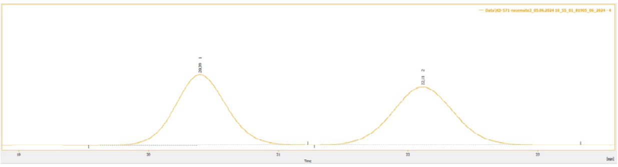

	Reten. Time [min]	Area [mAU.s]	Height [mAU]	Area [%]
1	19,892	19326,861	696,112	85,9
2	21,747	3185,493	101,847	14,1
	Total	22512,354	797,959	100,0

	Reten. Time [min]	Area [mAU.s]	Height [mAU]	Area [%]
1	20,394	25965,371	932,866	50,1
2	22,111	25881,438	771,345	49,9
	Total	51846,809	1704,211	100,0

(1*R*,2*R*)-2-((4-chlorophenyl)(methyl)amino)-1,2-dihydronaphthalen-1-ol (7w)

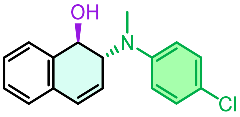

$C_{17}H_{16}ClNO$
285,77 g/mol

Following **G. P. E** vial containing **Rh5b** (4 mg, 5 mol%) was charged to glovebox and into reaction vial added 1,4-dihydro-1,4-epoxynaphthalene (16.2 mg, 0.112 mmol, 1 eq.) and 4-chloro-*N*-methylaniline (67 µL, 0.56 mmol, 5 eq.) followed by dry THF (0.22 mL) reaction mixture was stirred at 80 °C for 16 h. After the reaction was completed, the vial was taken from glovebox and concentrated under reduced pressure, from the crude reaction mixture prepared NMR sample. Column chromatography (SiO_2, pentane/Et_2O 9:1, v/v) of the crude mixture gave **7w** as a brown oil (31 mg, 89%). The *ee* was determined to be 65 % using chiral HPLC (AD-H, *i*-propanol/*n*-hexane = 5/95, flow rate = 0.5 mL/min, l = 254 nm) t_R = 31.8 (major), 36.3 (minor).

Rf 0.25 (SiO_2, Hex/Et_2O 3:1, v/v).

^{1}H NMR (400 MHz, $CDCl_3$) δ 7.56 – 7.49 (m, 1H), 7.32 – 7.26 (m, 2H), 7.23 – 7.16 (m, 2H), 7.16 – 7.11 (m, 1H), 6.90 – 6.84 (m, 2H), 6.61 (dd, *J* = 9.8, 2.5 Hz, 1H), 5.90 (dd, *J* = 9.8, 3.1 Hz, 1H), 5.07 (d, *J* = 9.4 Hz, 1H), 4.67 (dt, *J* = 9.5, 2.8 Hz, 1H), 2.81 (s, 3H), 2.39 (s, 1H).

^{13}C NMR (101 MHz, $CDCl_3$) δ 148.8, 136.4, 132.0, 130.1, 129.1, 128.3, 128.2, 127.3, 126.7, 125.8, 123.0, 115.8, 70.2, 63.7, 33.7.

$[\alpha]^{25}_D$ = +44 (c = 1.00, $CHCl_3$).

The spectral data were consistent with the literature.[24]

ee determination:

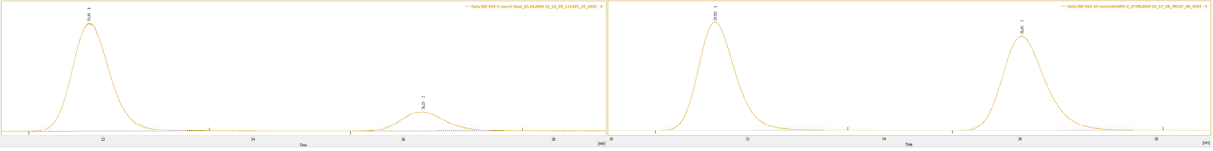

	Reten. Time [min]	Area [mAU.s]	Height [mAU]	Area [%]
1	31,808	27111,899	766,656	82,6
2	36,263	5694,952	136,527	17,4
	Total	32806,851	903,183	100,0

	Reten. Time [min]	Area [mAU.s]	Height [mAU]	Area [%]
1	31,518	23108,408	601,634	50,0
2	36,023	23130,727	524,982	50,0
	Total	46239,135	1126,616	100,0

(1*R*,2*R*)-2-((4-methoxyphenyl)(methyl)amino)-1,2-dihydronaphthalen-1-ol (7x)

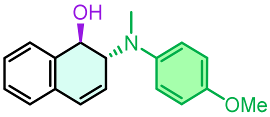

$C_{18}H_{19}NO_2$
281,36 g/mol

Following **G. P. E** vial containing **Rh5b** (4 mg, 5 mol%) was charged to glovebox and into reaction vial added 1,4-dihydro-1,4-epoxynaphthalene (16.2 mg, 0.112 mmol, 1 eq.) and *N*-methyl-*N*-(4-methoxyphenyl)amine (80 mg, 0,56 mmol, 5 eq.) followed by dry THF (0.22 mL) reaction mixture was stirred at 80 °C for 16 h. After the reaction was completed, the vial was taken from glovebox and concentrated under reduced pressure, from the crude reaction mixture prepared NMR sample. Column chromatography (SiO_2, pentane/Et_2O 9:1, v/v) of the crude mixture gave **7x** as a brown oil (30 mg, 96%). The *ee* was determined to be 70 % using chiral HPLC (OD-H, *i*-propanol/*n*-hexane = 10/90, flow rate = 0.5 mL/min, l = 254 nm) t_R = 23.1 (minor), 27.8 (major).

Rf 0.07 (SiO_2, pentane/Et_2O 9:1, v/v).

^{1}H NMR (400 MHz, $CDCl_3$) δ 7.60 – 7.52 (m, 1H), 7.30 – 7.22 (m, 2H), 7.09 (dd, *J* = 6.8, 2.0 Hz, 1H), 6.96 – 6.91 (m, 2H), 6.86 – 6.81 (m, 2H), 6.55 (dd, *J* = 9.8, 2.5 Hz, 1H), 5.94 (dd, *J* = 9.8, 2.8 Hz, 1H), 5.08 (d, *J* = 10.7 Hz, 1H), 4.52 (dt, *J* = 10.4, 2.7 Hz, 1H), 3.76 (s, 3H), 2.78 (s, 3H), 2.61 (s, 1H).

^{13}C NMR (101 MHz, $CDCl_3$) δ 153.1, 144.9, 136.8, 132.1, 129.7, 128.1, 127.9, 127.6, 126.5, 125.4, 117.6, 114.8, 69.8, 65.5, 55.8, 34.1.

$[\alpha]^{25}_D$ = +44 (c = 1.00, $CHCl_3$).

The spectral data were consistent with the literature.[25]

ee determination:

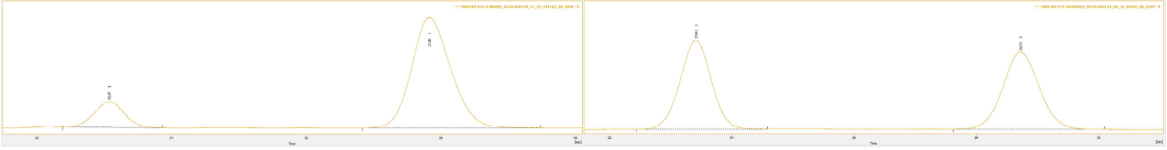

	Reten. Time [min]	Area [mAU.s]	Height [mAU]	Area [%]
1	23,066	6382,182	205,875	15,2
2	27,824	35679,803	887,100	84,8
	Total	42061,985	1092,976	100,0

	Reten. Time [min]	Area [mAU.s]	Height [mAU]	Area [%]
1	23,413	12806,479	359,787	49,8
2	28,721	12904,206	311,828	50,2
	Total	25710,685	671,615	100,0

(1*R*,2*R*,5*S*,6*R*)-5,6-bis((benzyloxy)methyl)-2-(1-methyl-1*H*-indol-3-yl)cyclohex-3-en-1-ol (7y)

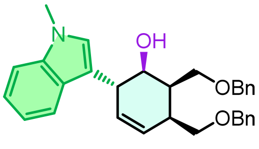

$C_{31}H_{33}NO_3$
467,61 g/mol

Following **G. P. E** vial containing **Rh5b** (4 mg, 5 mol%) was charged to glovebox and into reaction vial added **6c** (38 mg, 0.112 mmol, 1 eq.) and 1-methyl-1*H*-indole (140 μL, 1.12 mmol, 10 eq.) reaction mixture was stirred at 110 °C for 3 days. After the reaction was completed, the vial was taken from glovebox and concentrated under reduced pressure, from the crude reaction mixture prepared NMR sample. Column chromatography (SiO_2, petroleum ether/EtOAc 95:5, gradation till 8:2, v/v) of the crude mixture gave **7y** as a brown oil (29 mg, 55%). The *ee* was determined to be 69 % using chiral HPLC (OD-H, *i*-propanol/*n*-hexane = 15/85, flow rate = 0.5 mL/min, l = 254 nm) t_R = 29.1 (minor), 38.2 (major).

Rf 0.35 (SiO_2, petroleum ether/EtOAc 7:3, v/v).

^{1}H NMR (600 MHz, $CDCl_3$) δ 7.78 (d, *J* = 7.9 Hz, 1H), 7.50 – 7.31 (m, 9H), 7.31 – 7.27 (m, 3H), 7.21 – 7.15 (m, 1H), 6.82 (s, 1H), 6.04 – 5.98 (m, 1H), 5.91 (dd, *J* = 10.1, 2.4 Hz, 1H), 4.72 – 4.61 (m, 2H), 4.53 (d, *J* = 12.0 Hz, 1H), 4.40 (d, *J* = 11.7 Hz, 1H), 4.32 (d, *J* = 11.7 Hz, 1H), 4.02 (s, 1H), 3.88 (s, 1H), 3.78 (s, 3H), 3.62 (qd, *J* = 9.4, 3.4 Hz, 3H), 3.56 (dd, *J* = 9.2, 5.3 Hz, 1H), 2.80 (s, 1H), 2.63 (dtd, *J* = 9.9, 7.2, 6.3, 3.7 Hz, 1H).

^{13}C NMR (151 MHz, $CDCl_3$) δ 138.2, 137.5, 137.3, 128.7, 128.6, 128.5, 128.4, 128.1, 128.1, 127.9, 127.8, 127.6, 127.1, 121.8, 119.3, 119.0, 115.9, 109.4, 73.6, 73.4, 70.9, 70.7, 68.9, 41.6, 37.1, 36.6, 32.8.

$[\alpha]^{25}_D$ = -27 (c = 0.25, $CHCl_3$).

IR (ATR neat) ṽ =: 3390, 3058, 3026, 2918, 2853, 1719, 1613, 1453, 1364, 1056 cm^{-1}.

HRMS (ESI): *m*/*z* calculated for $C_{31}H_{33}NNaO_3{}^+$ [M + Na^+] 490.2353; found 490.2349.

***ee* determination:**

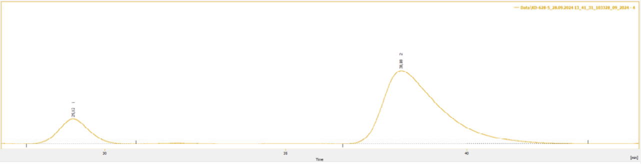

	Reten. Time [min]	Area [mAU.s]	Height [mAU]	Area [%]
1	29,122	4845,966	82,609	15,3
2	38,176	26915,472	244,314	84,7
	Total	31761,438	326,923	100,0

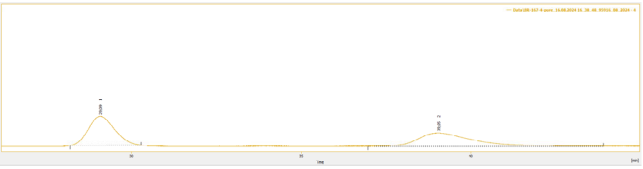

	Reten. Time [min]	Area [mAU.s]	Height [mAU]	Area [%]
1	29,085	2490,977	46,496	51,0
2	39,050	2396,802	21,283	49,0
	Total	4887,779	67,779	100,0

5.3 Prepared modifications for product **7j**.

5.3.1 Scale up for synthesis with **7j**.

4-bromo-*N*-((1*S*,2*S*)-2-(4-bromo-1*H*-indol-3-yl)-1,2-dihydronaphthalen-1-yl) benzenesulfonamide (7j)

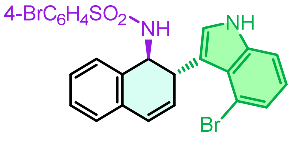

$C_{24}H_{18}Br_2N_2O_2S$
558,29 g/mol

Following **G. P. E** vial containing **Rh5b** (35 mg, 5 mol%) was charged to glovebox and into reaction vial added ***N*-Bos** (355 mg, 0.98 mmol, 1 eq.) and 4-bromo-1*H*-indole (0.61 mL, 4.9 mmol, 5 eq.) followed by dry $MeNO_2$ (2 mL) reaction mixture was stirred at 80 °C for 16 h. After the reaction was completed, the vial was taken from glovebox and concentrated under reduced pressure, from the crude reaction mixture prepared NMR sample. Column chromatography (SiO_2, petroleum ether/EtOAc 7:3, v/v) of the crude mixture gave **7j** as a light brown oil (375 mg, 62%). The *ee* was determinated to be 86 % using chiral HPLC (OD-H, *i*-propanol/*n*-hexane = 15/85, flow rate = 1.0 mL/min, l = 254 nm) t_R = 20.7 (minor), 22.6 (major).

***ee* determination:**

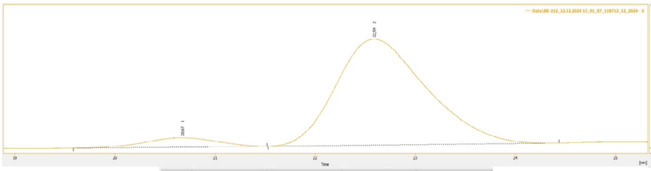

	Reten. Time [min]	Area [mAU.s]	Height [mAU]	Area [%]
1	20,673	1037,248	20,797	6,5
2	22,588	14955,361	238,985	93,5
	Total	15992,609	259,782	100,0

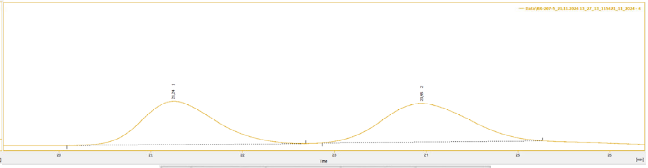

	Reten. Time [min]	Area [mAU.s]	Height [mAU]	Area [%]
1	21,241	1950,305	34,724	49,2
2	23,949	2015,650	30,976	50,8
	Total	3965,955	65,700	100,0

5.3.2 Reduction of **7j**.

***N*-((1*S*,2*S*)-2-(1*H*-indol-3-yl)-1,2,3,4-tetrahydronaphthalen-1-yl)benzenesulfonamide (8a)**

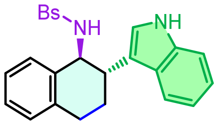

$C_{24}H_{22}N_2O_2S$
402,51 g/mol

In a 10 mL vial, **7j** (56 mg, 0.1 mmol, 1 eq.) and Pd/C (10 mg, 0.01 mmol, 10 mol%) were dissolved in dry and degassed MeOH (2 mL). This solution was transferred to a reaction flask in laminar and subjected to 15 bar of H_2 at room temperature for 16 hours. After the reaction, the solution was filtered through a short pad of Celite and washed with MeOH. The solvent was removed under reduced pressure to obtain the crude product. Column chromatography (SiO_2, petroleum ether/EtOAc 8:2, v/v) of the crude mixture yielded **8a** as a light yellow oil (12 mg, 20%). The ee was determined to be 84 % using chiral HPLC (OD-H, *i*-propanol/*n*-hexane = 15/85, flow rate = 1.0 mL/min, l = 254 nm) t_R = 21.6 (major), 27.2 (minor).

Rf 0.30 (SiO_2, petroleum ether/EtOAc 7:3, v/v).

^{1}H NMR (400 MHz, $CDCl_3$) δ 7.71 (s, 1H), 7.66 (dd, *J* = 8.4, 1.1 Hz, 2H), 7.51 (d, *J* = 7.8 Hz, 1H), 7.47 (tt, *J* = 7.5, 1.2 Hz, 1H), 7.33 (t, *J* = 7.8 Hz, 2H), 7.28 – 7.23 (m, 1H), 7.23 – 7.16 (m, 2H), 7.16 – 7.05 (m, 4H), 6.46 (d, *J* = 2.3 Hz, 1H), 4.76 (d, *J* = 7.2 Hz, 1H), 4.72 (dd, *J* = 11.5, 4.7 Hz, 1H), 3.52 (q, *J* = 4.4 Hz, 1H), 2.76 (dt, *J* = 17.2, 5.5 Hz, 1H), 2.71 – 2.58 (m, 1H), 2.22 (tdd, *J* = 12.9, 6.5, 3.6 Hz, 1H), 2.13 (dq, *J* = 12.0, 5.9 Hz, 1H).

^{13}C NMR (101 MHz, $CDCl_3$) δ 141.0, 137.6, 136.0, 135.7, 132.3, 129.7, 129.3, 128.9, 128.0, 126.9, 126.8, 122.2, 121.3, 119.5, 119.0, 116.6, 111.3, 57.2, 37.0, 26.4, 24.9.

$[\alpha]^{25}_D$ = +77 (c = 0.33, $CHCl_3$).

IR (ATR neat) ṽ: 3388, 3297, 3056, 2923, 2853, 1656, 1490, 1446, 1387, 1320, 1308, 1250, 1223, 1151 cm^{-1}.

HRMS (ESI): *m/z* calculated for $C_{24}H_{22}N_2NaO_2S^+$ [M + Na$^+$] 425.1294; found 425.1303.

***ee* determination:**

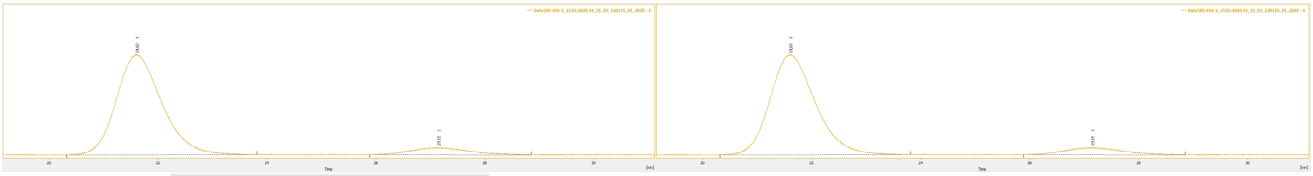

	Reten. Time [min]	Area [mAU.s]	Height [mAU]	Area [%]
1	21,616	8657,308	149,590	92,0
2	27,153	755,668	10,170	8,0
	Total	9412,976	159,760	100,0

5.3.3 Debromination of **7j**.

***N*-((1*S*,2*S*)-2-(1*H*-indol-3-yl)-1,2-dihydronaphthalen-1-yl)benzenesulfonamide (8b)**

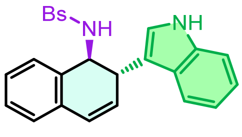

$C_{24}H_{20}N_2O_2S$
400,50 g/mol

In a 10 mL vial, **7j** (56 mg, 0.1 mmol, 1 eq.) and Mg (10 mg, 0.5 mmol, 5 eq.) were dissolved in dry and degassed MeOH (2 mL). Mg was activated by the addition of a drop of dibromoethane. The solution was stirred at room temperature for 16 hours. After the reaction, extraction was performed using DCM (3 x 20 mL), and the combined organic fractions were dried over $MgSO_4$, filtered, and the solvent was removed under reduced pressure. Column chromatography (SiO_2, petroleum ether/EtOAc 7:3, v/v) of the crude mixture yielded **8b** as a light yellow oil (32 mg, 80%). The ee was determined to be 83 % using chiral HPLC (OD-H, *i*-propanol/*n*-hexane = 15/85, flow rate = 1.0 mL/min, l = 254 nm) t_R = 22.5 (minor), 26.9 (major).

^{1}H NMR (400 MHz, $CDCl_3$) δ 7.96 (s, 1H), 7.56 (dd, *J* = 8.4, 1.1 Hz, 2H), 7.38 (t, *J* = 7.4 Hz, 1H), 7.28 – 7.20 (m, 3H), 7.18 – 7.12 (m, 3H), 7.05 (d, *J* = 7.4 Hz, 1H), 7.01 – 6.96 (m, 2H), 6.94 (t, *J* = 7.8 Hz, 1H), 6.65 (d, *J* = 2.6 Hz, 1H), 6.59 (d, *J* = 9.5 Hz, 1H), 6.19 – 6.10 (m, 1H), 5.31 (d, *J* = 8.3 Hz, 1H), 4.84 (dd, *J* = 9.5, 3.6 Hz, 2H).

^{13}C NMR (101 MHz, $CDCl_3$) δ 141.3, 137.5, 133.2, 132.5, 132.1, 130.4, 128.7, 128.6, 128.5, 128.0, 127.4, 126.7, 126.4, 124.6, 124.5, 124.3, 123.0, 113.9, 113.8, 110.9, 58.3, 37.8.

$[\alpha]^{25}_D$ = +51 (c = 1.00, $CHCl_3$).

IR (ATR neat) ṽ: 3349, 3058, 2923, 2852, 1478, 1446, 1417, 1333 cm^{-1}.

HRMS (ESI): *m/z* calculated for $C_{24}H_{20}N_2NaO_2S^+$ [M + Na$^+$] 423.1138; found 423.1139.

***ee* determination:**

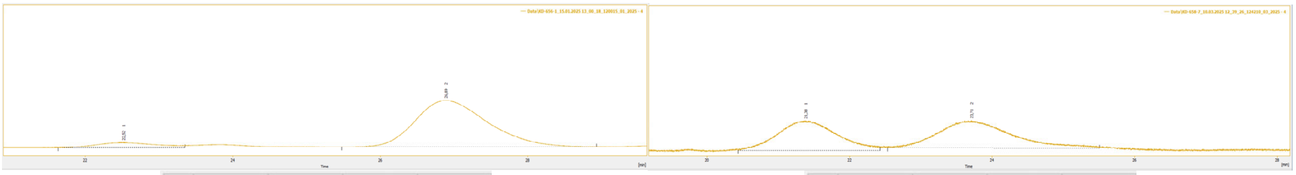

	Reten. Time [min]	Area [mAU.s]	Height [mAU]	Area [%]
1	22,517	380,393	6,658	8,6
2	26,887	4018,594	58,806	91,4
	Total	4398,987	65,464	100,0

	Reten. Time [min]	Area [mAU.s]	Height [mAU]	Area [%]
1	21,383	1384,601	23,163	50,3
2	23,713	1367,245	20,785	49,7
	Total	2751,846	43,948	100,0

5.3.4 Oxydation of **7j**.

4-bromo-*N*-((1*S*,2*S*,3*S*,4*R*)-2-(4-bromo-1*H*-indol-3-yl)-3,4-dihydroxy-1,2,3,4-tetrahydronaphthalen-1-yl) benzenesulfonamide (8c)

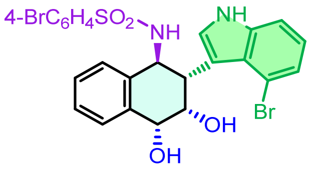

$C_{24}H_{20}Br_2N_2O_4S$
592,30 g/mol

In a 10 mL vial, **7j** (28 mg, 0.005 mmol, 1 eq.) and NMO (30 mg, 0.25 mmol, 5 eq.) were dissolved in a mixture of *t*BuOH/THF/H_2O (7:2:1, 2 mL), and then OsO_4 (30 µL, 10 mol%, 2.5% *t*BuOH) was added. The reaction vial was sealed with a Teflon cap and stirred at room temperature for 16 hours. After that, the solution was quenched with $Na_2S_2O_3$ and extracted with DCM (4 x 20 mL). The combined organic fractions were dried over $MgSO_4$, filtered, and the solvent was removed under reduced pressure. Column chromatography (SiO_2, petroleum ether/EtOAc 1:1 followed by DCM/MeOH 98:2, v/v) of the crude mixture yielded **8c** as an oil (12 mg, 41%). The *ee* was determined to be 82% using chiral HPLC (AD-H, *i*-propanol/*n*-hexane = 15/85, flow rate = 1.0 mL/min, l = 254 nm) t_R = 36.0 (minor), 42.6 (major).

Rf 0.25 (SiO_2, DCM/MeOH 98:2, v/v).

^{1}H NMR (^{1}H NMR (400 MHz, CD_3CN) δ 9.19 (s, 1H), 7.55 (s, 1H), 7.43 – 7.31 (m, 2H), 7.29 (d, *J* = 7.3 Hz, 1H), 7.27 – 7.10 (m, 4H), 7.07 (d, *J* = 7.6 Hz, 1H), 6.87 (t, *J* = 7.8 Hz, 1H), 6.79 (s, 1H), 6.27 (d, *J* = 9.1 Hz, 1H), 4.77 (s, 1H), 4.43 (s, 1H), 4.21 (s, 1H), 4.11 (s, 1H), 3.50 (d, *J* = 15.7 Hz, 1H), 3.43 (d, *J* = 4.7 Hz, 1H), 3.18 (s, 1H).

^{13}C NMR (^{13}C NMR (101 MHz, CD_3CN) δ 141.7, 137.7, 132.5, 130.1, 129.7, 129.2, 128.9, 128.1, 126.1, 124.8, 123.3, 112.4, 73.8, 69.2, 59.5, 40.1.

$[\alpha]^{25}_D$ = +21 (c = 1.00, $CHCl_3$).

IR (ATR neat) ṽ: 3326, 3065, 2924, 1573, 1470, 1453, 1419, 1388, 1330, 1275 cm^{-1}.

HRMS (ESI): *m*/*z* calculated for $C_{24}H_{20}Br_2N_2NaO_4S^+$ [M + Na^+] 614.9382; found 614.9385.

***ee* determination:**

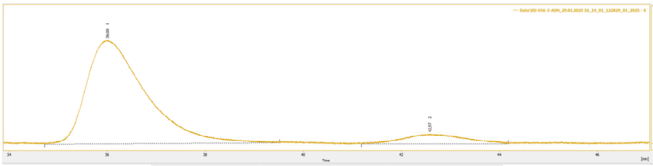

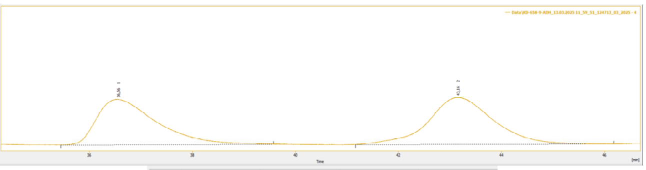

	Reten. Time [min]	Area [mAU.s]	Height [mAU]	Area [%]
1	35,999	3669,520	43,990	91,2
2	42,573	354,316	4,240	8,8
	Total	4023,836	48,231	100,0

	Reten. Time [min]	Area [mAU.s]	Height [mAU]	Area [%]
1	36,557	5439,165	68,095	50,6
2	43,157	5306,723	66,461	49,4
	Total	10745,888	134,556	100,0

5.3.5 Suzuki coupling of **7j**.

4'-methoxy-*N*-((1*S*,2*S*)-2-(4-(4-methoxyphenyl)-1*H*-indol-3-yl)-1,2-dihydronaphthalen-1-yl)-[1,1'-biphenyl]-4-sulfonamide (8d)

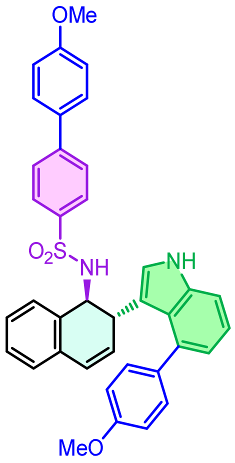

$C_{38}H_{32}N_2O_4S$
612,74 g/mol

In a 10 mL vial, **7j** (35 mg, 0.063 mmol, 1 eq.) was dissolved in 1 mL of dry, degassed Tol, followed by the addition of $Pd(PPh_3)_4$ (7 mg, 0.006 mmol, 10 mol%) and 4-methoxyphenylboronic acid (24 mg, 0.157 mmol, 2.5 eq.). After stirring the solution for 15 minutes, aqueous Na_2CO_3 (0.4 mL, 0.38 mmol, 6 eq., 1 mol/dm³) was added, and the reaction mixture was stirred at 110 °C for 2 days. Once the reaction was complete and the mixture was cooled down to room temperature, it was quenched by the addition of water (1 mL), followed by filtration over Celite and washing with EtOAc. The crude mixture was then extracted with EtOAc (2 x 10 mL). The combined organic fractions were dried over $MgSO_4$, filtered, and the solvent was removed under reduced pressure. Column chromatography (SiO_2, petroleum ether/EtOAc 2:1, v/v) of the crude mixture yielded **8d** as an oil (22 mg, 57%). The *ee* was determined to be 84% using chiral HPLC (AD-H, *i*-propanol/*n*-hexane = 15/85, flow rate = 1.0 mL/min, l = 254 nm) t_R = 49.0 (minor), 61.1 (major).

Rf 0.42 (SiO_2, petroleum ether/EtOAc 2:1, v/v).

^{1}H NMR (400 MHz, $CDCl_3$) δ 7.94 – 7.89 (m, 1H), 7.54 – 7.46 (m, 2H), 7.43 – 7.30 (m, 5H), 7.22 – 7.14 (m, 3H), 7.12 – 6.96 (m, 7H), 6.91 (ddd, *J* = 7.2, 5.0, 3.2 Hz, 2H), 6.58 (d, *J* = 2.4 Hz, 1H), 6.36 (d, *J* = 9.5 Hz, 1H), 5.71 (dd, *J* = 9.6, 5.1 Hz, 1H), 4.62 – 4.52 (m, 2H), 3.89 (s, 3H), 3.87 (s, 3H), 3.72 (t, *J* = 4.2 Hz, 1H).

^{13}C NMR (101 MHz, $CDCl_3$) δ 160.0, 159.1, 144.1, 139.2, 136.5, 135.2, 133.8, 132.5, 132.4, 132.0, 130.7, 130.3, 129.0, 128.4, 128.3, 127.6, 127.0, 126.4, 126.2, 124.0, 122.8, 122.2, 121.8, 114.6, 114.4, 110.4, 57.7, 55.5, 55.5, 38.3.

$[\alpha]^{25}_D$ = +9 (c = 0.10, $CHCl_3$).

IR (ATR neat) ṽ: 3342, 3032, 2934, 2835, 1607, 1595, 1581, 1571, 1417, 1399, 1307, 1292 cm^{-1}.

HRMS (ESI): *m/z* calculated for $C_{38}H_{32}N_2NaO_4S^+$ [M + Na$^+$] 635.1975; found 635.1982.

***ee* determination:**

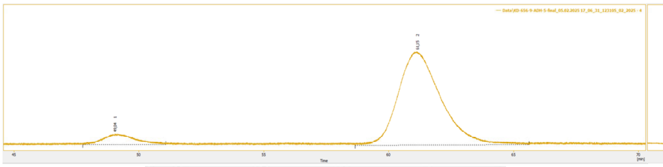

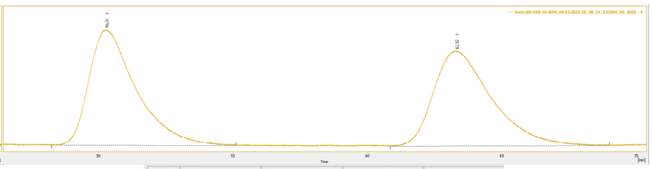

	Reten. Time [min]	Area [mAU.s]	Height [mAU]	Area [%]
1	49,044	256,186	3,059	7,8
2	61,145	3047,829	25,807	92,2
	Total	3304,015	28,866	100,0

	Reten. Time [min]	Area [mAU.s]	Height [mAU]	Area [%]
1	50,311	11640,148	95,149	49,9
2	63,322	11694,071	78,034	50,1
	Total	23334,219	173,184	100,0

6. References

(1) Neufeld, E.; Pounder, A.; Tam, W. Rhodium-Catalyzed Ring-Opening Reactions of Heterobicyclic Alkenes with Heteroarene Nucleophiles. *Tetrahedron Lett.* **2023**, *127*, 154685. https://doi.org/10.1016/j.tetlet.2023.154685.

(2) Gao, P.; Foster, D.; Sipos, G.; Skelton, B. W.; Sobolev, A. N.; Dorta, R. Chiral NHC-Iridium Complexes and Their Performance in Enantioselective Intramolecular Hydroamination and Ring-Opening Amination Reactions. *Organometallics* **2020**, *39* (4), 556–573. https://doi.org/10.1021/acs.organomet.9b00770.

(3) Pounder, A.; Farkas, M.; Chen, L. D.; Tam, W. Iridium/Zinc-Co-Catalyzed Ring-Opening Reactions of Oxabicyclic Alkenes with Indole Nucleophiles: A Combined Experimental and Theoretical Study. *Organometallics* **2023**, *42* (9), 780–792. https://doi.org/10.1021/acs.organomet.3c00017.

(4) Lautens, M.; Fagnou, K.; Yang, D. Rhodium-Catalyzed Asymmetric Ring Opening Reactions of Oxabicyclic Alkenes: Application of Halide Effects in the Development of a General Process. *J. Am. Chem. Soc.* **2003**, *125* (48), 14884–14892. https://doi.org/10.1021/ja034845x.

(5) Vila, J.; Solà, M.; Achard, T.; Bellemin-Laponnaz, S.; Pla-Quintana, A.; Roglans, A. Rh(I) Complexes with Hemilabile Thioether-Functionalized NHC Ligands as Catalysts for [2 + 2 + 2] Cycloaddition of 1,5-Bisallenes and Alkynes. *ACS Catal.* **2023**, *13* (5), 3201–3210. https://doi.org/10.1021/ACSCATAL.2C05790.

(6) Bannwarth, C.; Caldeweyher, E.; Ehlert, S.; Hansen, A.; Pracht, P.; Seibert, J.; Spicher, S.; Grimme, S. Extended TIGHT-BINDING Quantum Chemistry Methods. *WIREs Comput. Mol. Sci.* **2021**, *11* (2), e1493. https://doi.org/10.1002/wcms.1493.

(7) Pracht, P.; Bohle, F.; Grimme, S. Automated Exploration of the Low-Energy Chemical Space with Fast Quantum Chemical Methods. *Phys. Chem. Chem. Phys.* **2020**, *22* (14), 7169–7192. https://doi.org/10.1039/C9CP06869D.

(8) Grimme, S. Exploration of Chemical Compound, Conformer, and Reaction Space with Meta-Dynamics Simulations Based on Tight-Binding Quantum Chemical Calculations. *J. Chem. Theory Comput.* **2019**, *15* (5), 2847–2862. https://doi.org/10.1021/acs.jctc.9b00143.

(9) Bohle, F.; Grimme, S. Hydrocarbon Macrocycle Conformer Ensembles and^{13} C-NMR Spectra. *Angew. Chem. Int. Ed.* **2022**, *61* (14), e202113905. https://doi.org/10.1002/anie.202113905.

(10) Grimme, S.; Bohle, F.; Hansen, A.; Pracht, P.; Spicher, S.; Stahn, M. Efficient Quantum Chemical Calculation of Structure Ensembles and Free Energies for Nonrigid Molecules. *J. Phys. Chem. A* **2021**, *125* (19), 4039–4054. https://doi.org/10.1021/acs.jpca.1c00971.

(11) Neese, F. The ORCA Program System. *WIREs Comput. Mol. Sci.* **2012**, *2* (1), 73–78. https://doi.org/10.1002/wcms.81.

(12) Neese, F. Software Update: The ORCA Program System, Version 4.0. *WIREs Comput. Mol. Sci.* **2018**, *8* (1), e1327. https://doi.org/10.1002/wcms.1327.

(13) Grimme, S.; Ehrlich, S.; Goerigk, L. Effect of the Damping Function in Dispersion Corrected Density Functional Theory. *J. Comput. Chem.* **2011**, *32* (7), 1456–1465. https://doi.org/10.1002/jcc.21759.

(14) Grimme, S.; Antony, J.; Ehrlich, S.; Krieg, H. A Consistent and Accurate *Ab Initio* Parametrization of Density Functional Dispersion Correction (DFT-D) for the 94 Elements H-Pu. *J. Chem. Phys.* **2010**, *132* (15), 154104. https://doi.org/10.1063/1.3382344.

(15) Weigend, F.; Ahlrichs, R. Balanced Basis Sets of Split Valence, Triple Zeta Valence and Quadruple Zeta Valence Quality for H to Rn: Design and Assessment of Accuracy. *Phys. Chem. Chem. Phys.* **2005**, *7* (18), 3297. https://doi.org/10.1039/b508541a.

(16) Minozzi, C.; Grenier-Petel, J.-C.; Parisien-Collette, S.; Collins, S. K. Photocatalyic Appel Reaction Enabled by Copper-Based Complexes in Continuous Flow. *Beilstein J. Org. Chem.* **2018**, *14*, 2730–2736. https://doi.org/10.3762/bjoc.14.251.

(17) Uzarewicz-Baig, M.; Koppenwallner, M.; Tabassum, S.; Wilhelm, R. Highly Regioselective Synthesis of Chiral Diamines via a Buchwald–Hartwig Amination from Camphoric Acid and Their Application in the Henry Reaction. *Appl. Organomet. Chem.* **2014**, *28* (7), 552–558. https://doi.org/10.1002/aoc.3162.

(18) Seiders, T. J.; Ward, D. W.; Grubbs, R. H. Enantioselective Ruthenium-Catalyzed Ring-Closing Metathesis. *Org. Lett.* **2001**, *3* (20), 3225–3228. https://doi.org/10.1021/ol0165692.
(19) Wolf, S.; Plenio, H. Synthesis of (NHC)Rh(Cod)Cl and (NHC)RhCl(CO)2 Complexes – Translation of the Rh- into the Ir-Scale for the Electronic Properties of NHC Ligands. *J. Organomet. Chem.* **2009**, *694* (9–10), 1487–1492. https://doi.org/10.1016/j.jorganchem.2008.12.047.
(20) Pandey, G.; Varkhedkar, R.; Tiwari, D. Efficient Access to Enantiopure 1,3-Disubstituted Isoindolines from Selective Catalytic Fragmentation of an Original Desymmetrized Rigid Overbred Template. *Org. Biomol. Chem.* **2015**, *13* (15), 4438–4448. https://doi.org/10.1039/C5OB00229J.
(21) Banerjee, S.; Vivek Kumar, S.; Punniyamurthy, T. Site-Selective Rh-Catalyzed C-7 and C-6 Dual C–H Functionalization of Indolines: Synthesis of Functionalized Pyrrolocarbazoles. *J. Org. Chem.* **2020**, *85* (4), 2793–2805. https://doi.org/10.1021/acs.joc.9b03180.
(22) Chen, W.; Yang, W.; Wu, R.; Yang, D. Water-Promoted Synthesis of Fused Bicyclic Triazolines and Naphthols from Oxa(Aza)Bicyclic Alkenes and Transformation *via* a Novel Ring-Opening/Rearrangement Reaction. *Green Chem.* **2018**, *20* (11), 2512–2518. https://doi.org/10.1039/C7GC03772D.
(23) Maiti, M.; Jana, S. K.; Maji, B. Asymmetric Alkene–Alkene Reductive Cross-Coupling Reaction *via* Visible-Light Photoredox/Cobalt Dual Catalysis. *Chem. Commun.* **2023**, *59* (64), 9718–9721. https://doi.org/10.1039/D3CC02792A.
(24) Yang, D.; Hu, P.; Long, Y.; Wu, Y.; Zeng, H.; Wang, H.; Zuo, X. Iridium-Catalyzed Asymmetric Ring-Opening Reactions of Oxabicyclic Alkenes with Secondary Amine Nucleophiles. *Beilstein J. Org. Chem.* **2009**, *5*. https://doi.org/10.3762/bjoc.5.53.
(25) Zhou, Y.; Lu, Z.; Han, B.; Zeng, C.; Zhang, Z.; Fan, B. Iridium-Catalyzed Highly Enantioselective Ring Opening Reaction of Oxabenzonorbornadienes with Amines. *Tetrahedron Asymmetry* **2015**, *26* (23), 1354–1359. https://doi.org/10.1016/j.tetasy.2015.10.017.

7. Copy of NMR data Analysis

^1H NMR (400 MHz, $CDCl_3$) and ^{13}C NMR (101 MHz, $CDCl_3$) Analysis of Compound **1a**

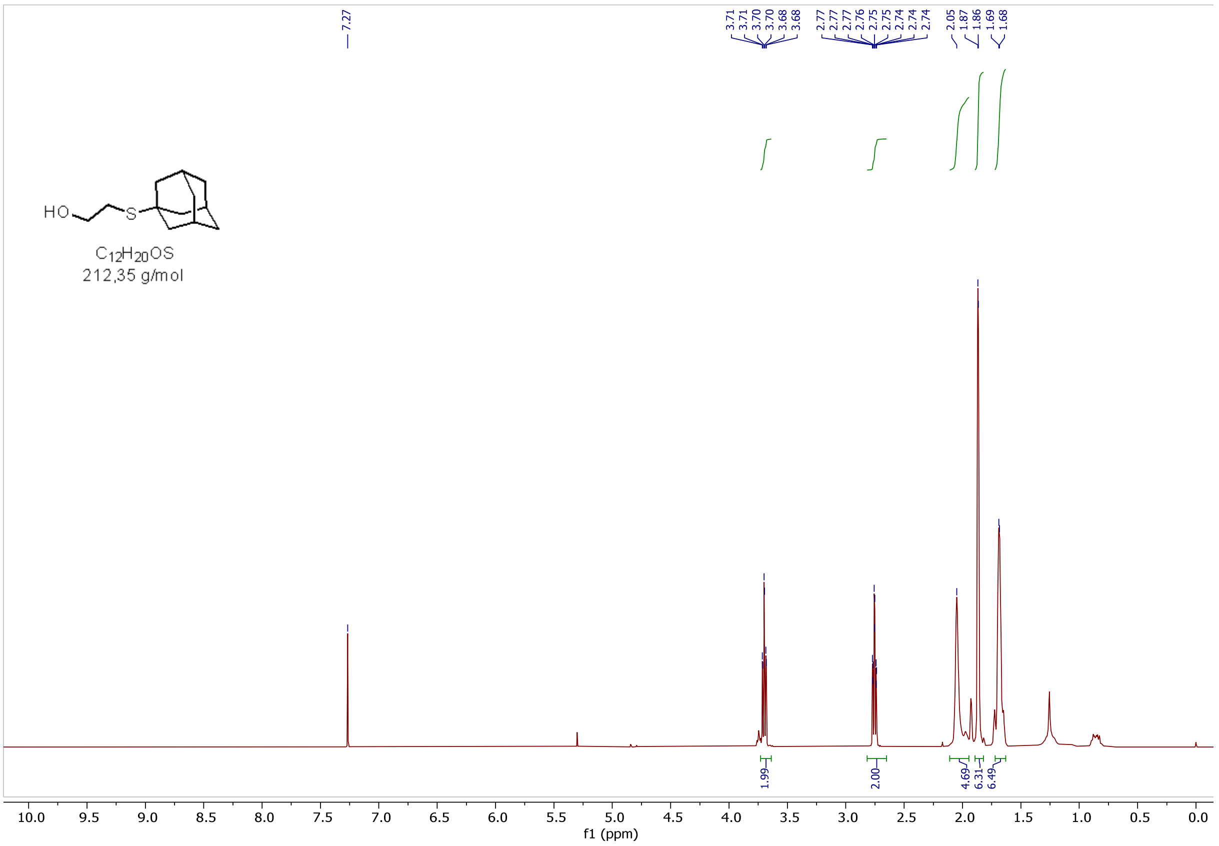

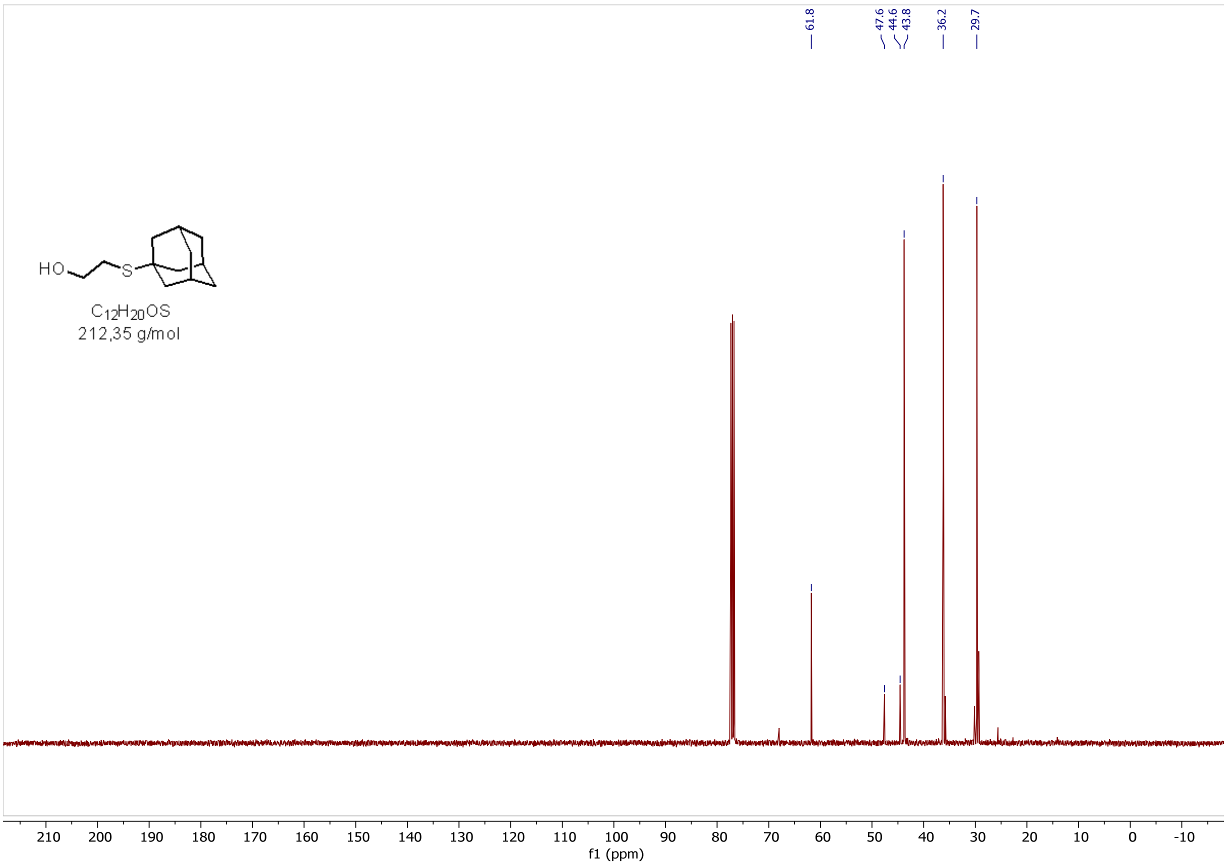

^{1}H NMR (400 MHz, $CDCl_3$) and ^{13}C NMR (101 MHz, $CDCl_3$) Analysis of Compound **1b**

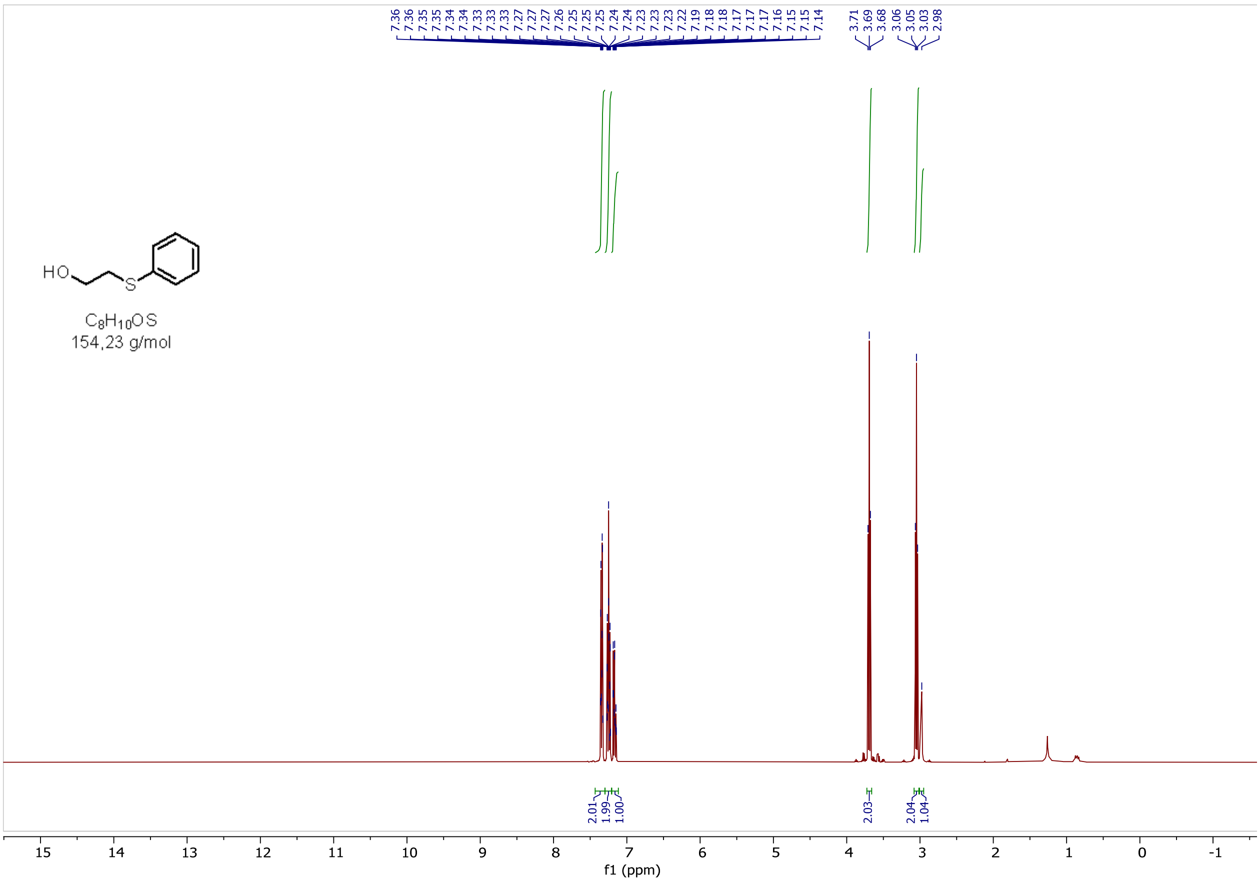

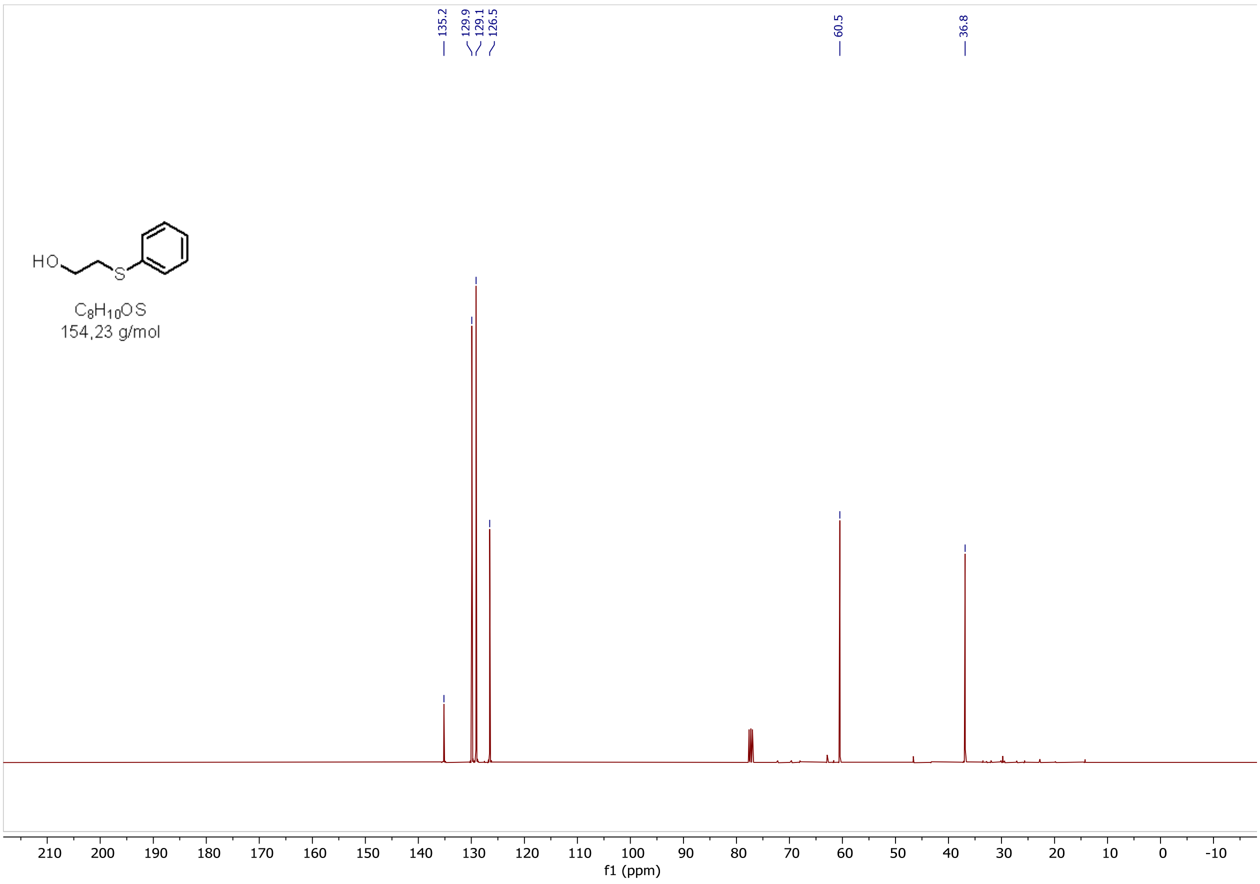

^{1}H NMR (400 MHz, $CDCl_3$) and ^{13}C NMR (101 MHz, $CDCl_3$) Analysis of Compound **1c**

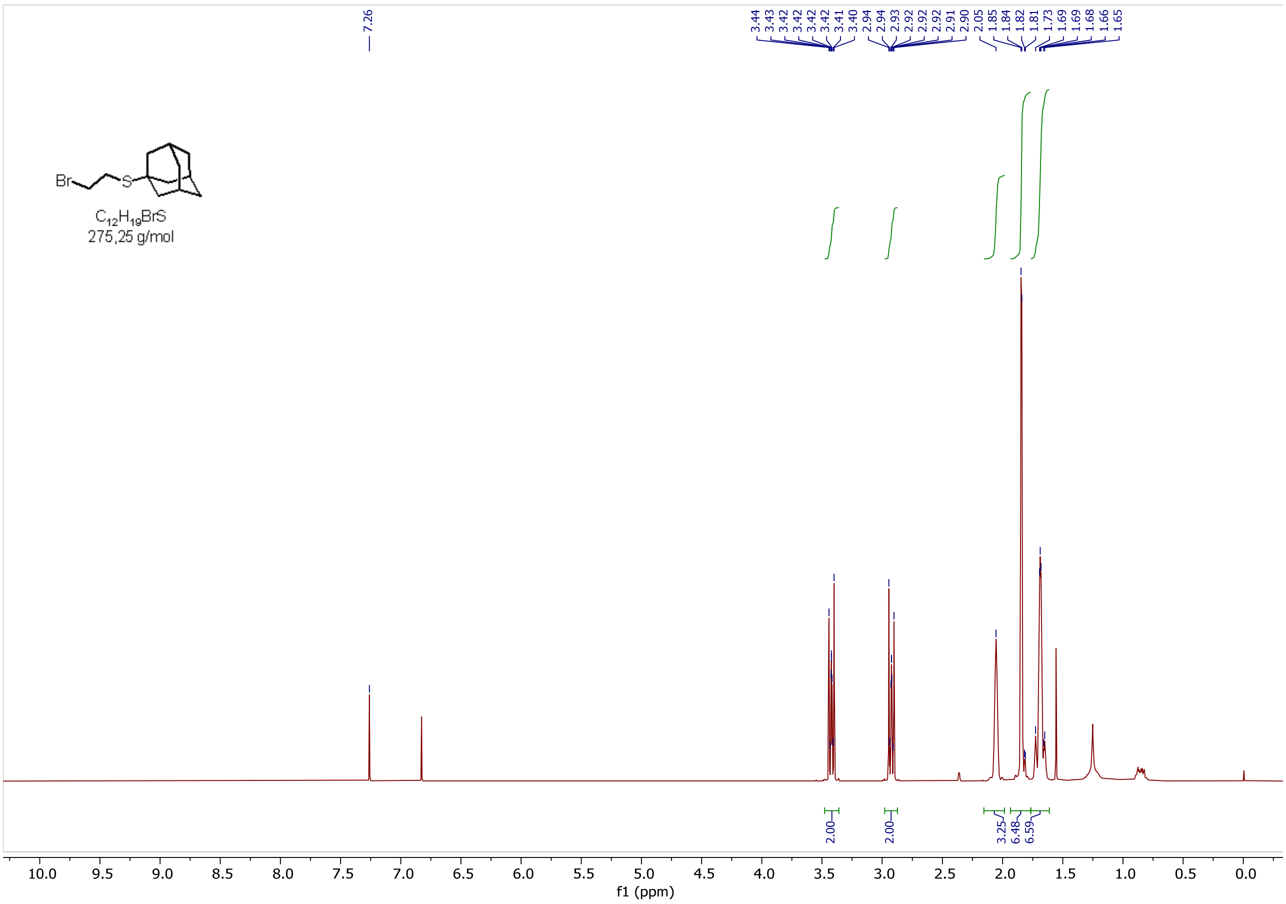

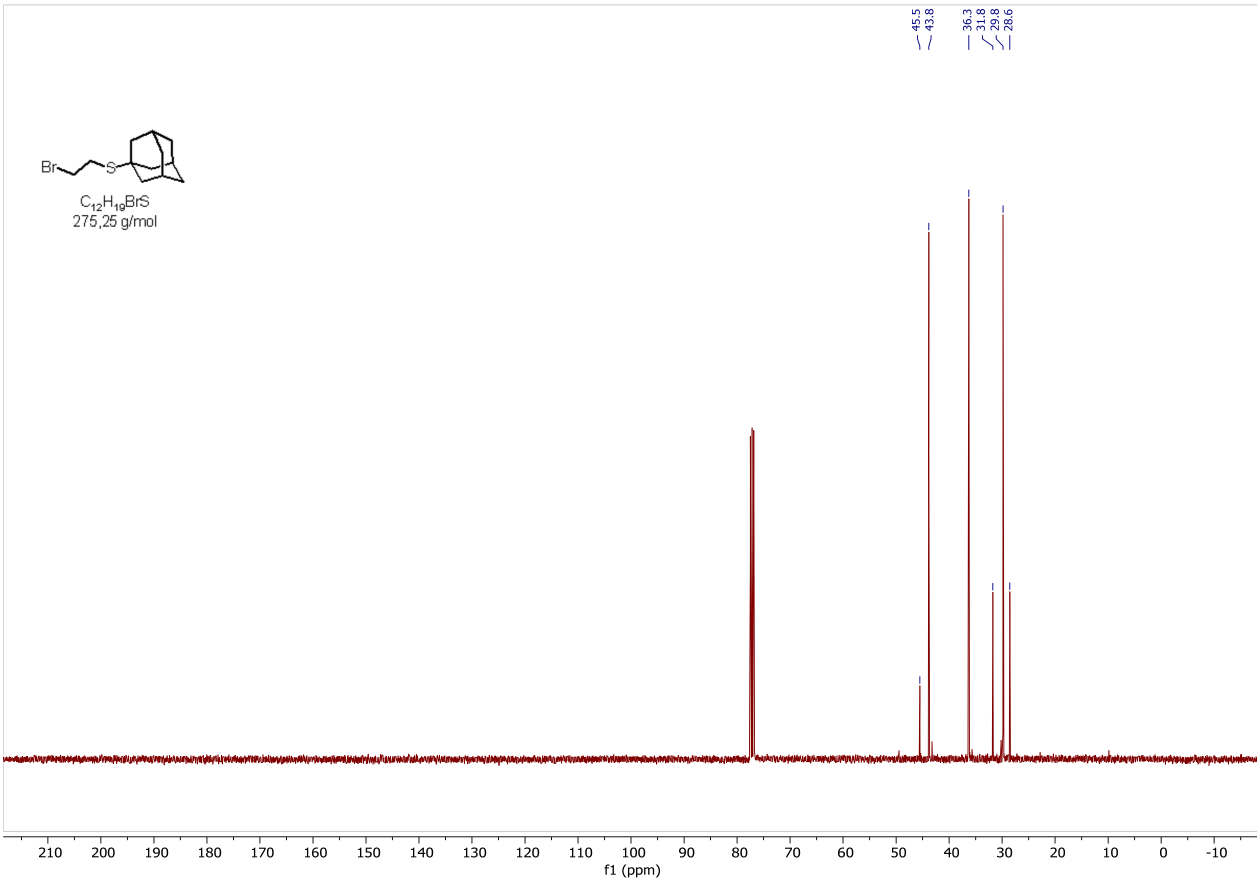

^{1}H NMR (400 MHz, $CDCl_3$) and ^{13}C NMR (101 MHz, $CDCl_3$) Analysis of Compound **1d**

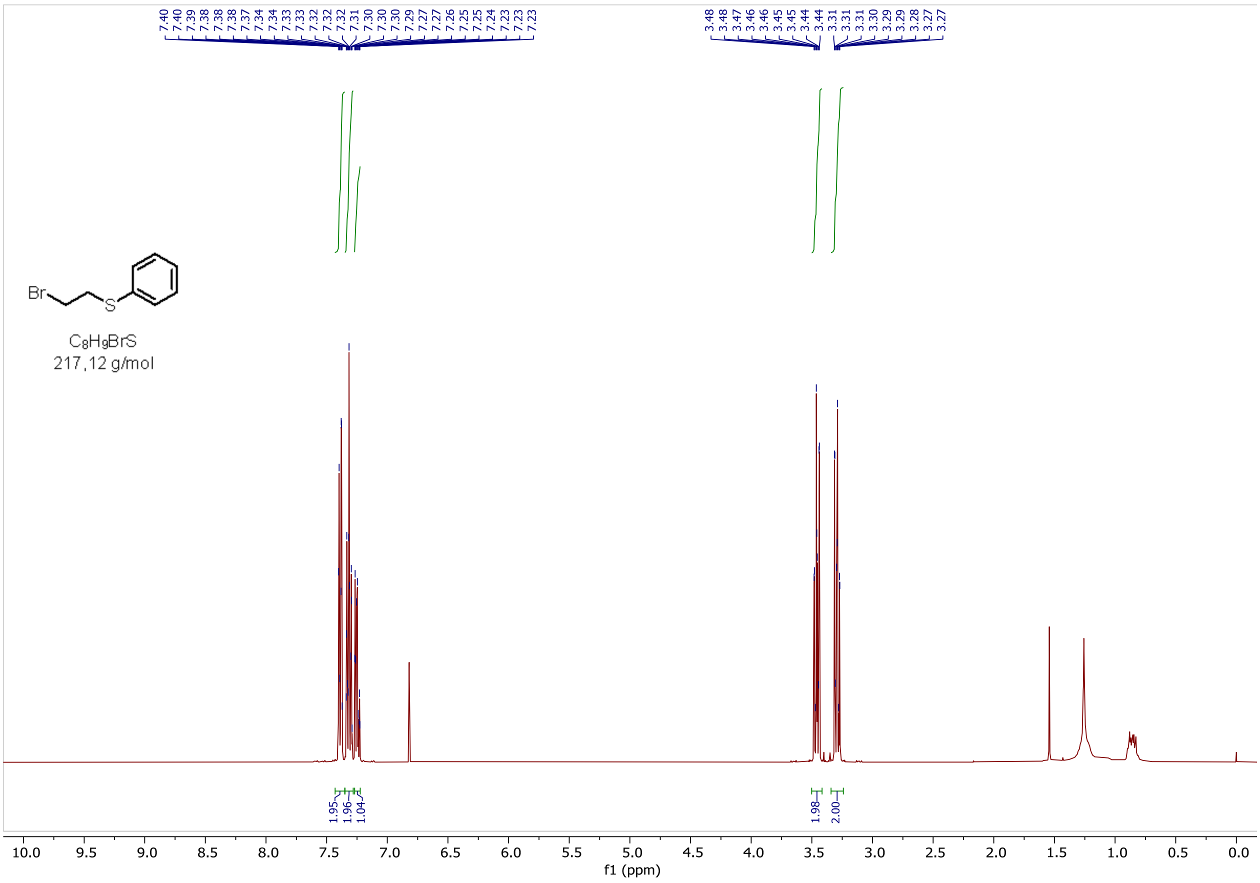

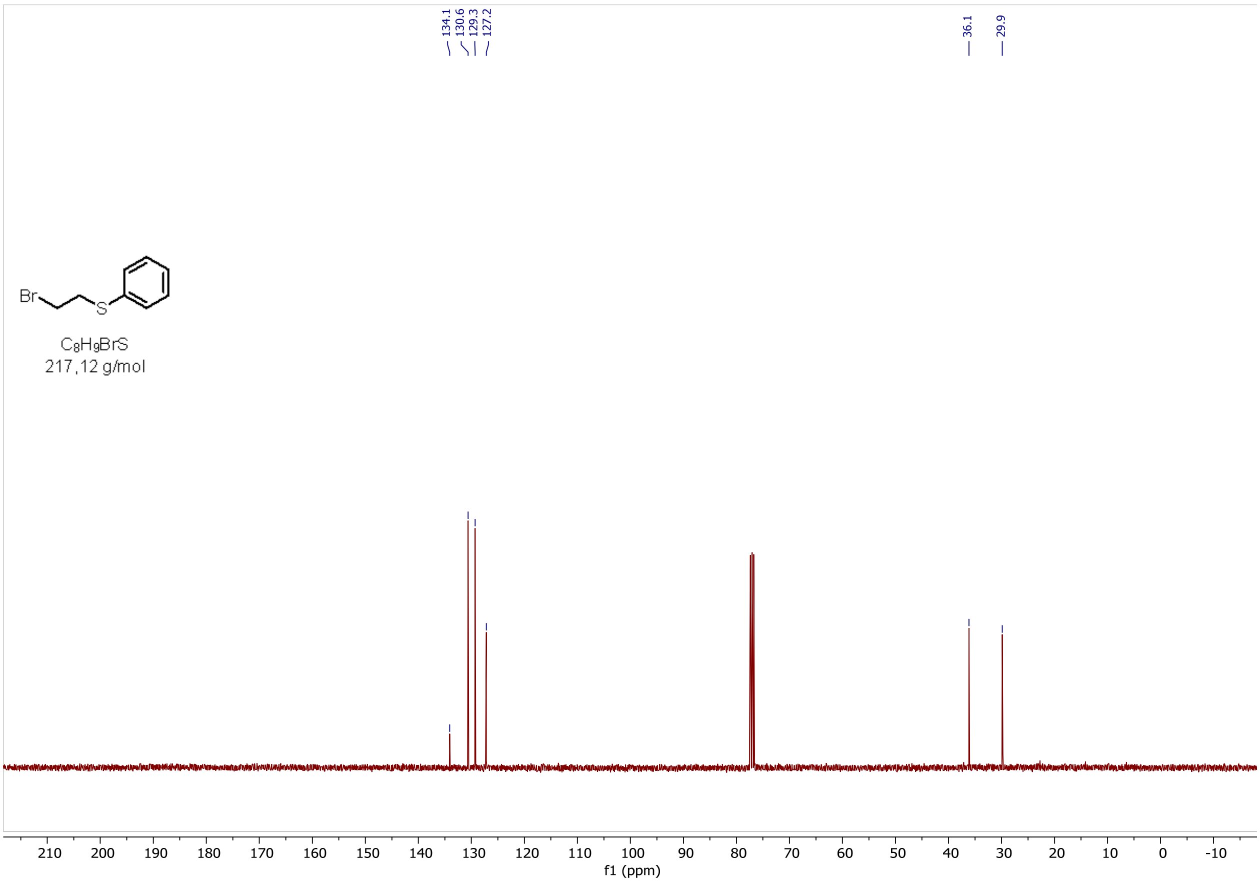

^{1}H NMR (400 MHz, $CDCl_3$) and ^{13}C NMR (101 MHz, $CDCl_3$) Analysis of Compound **2**

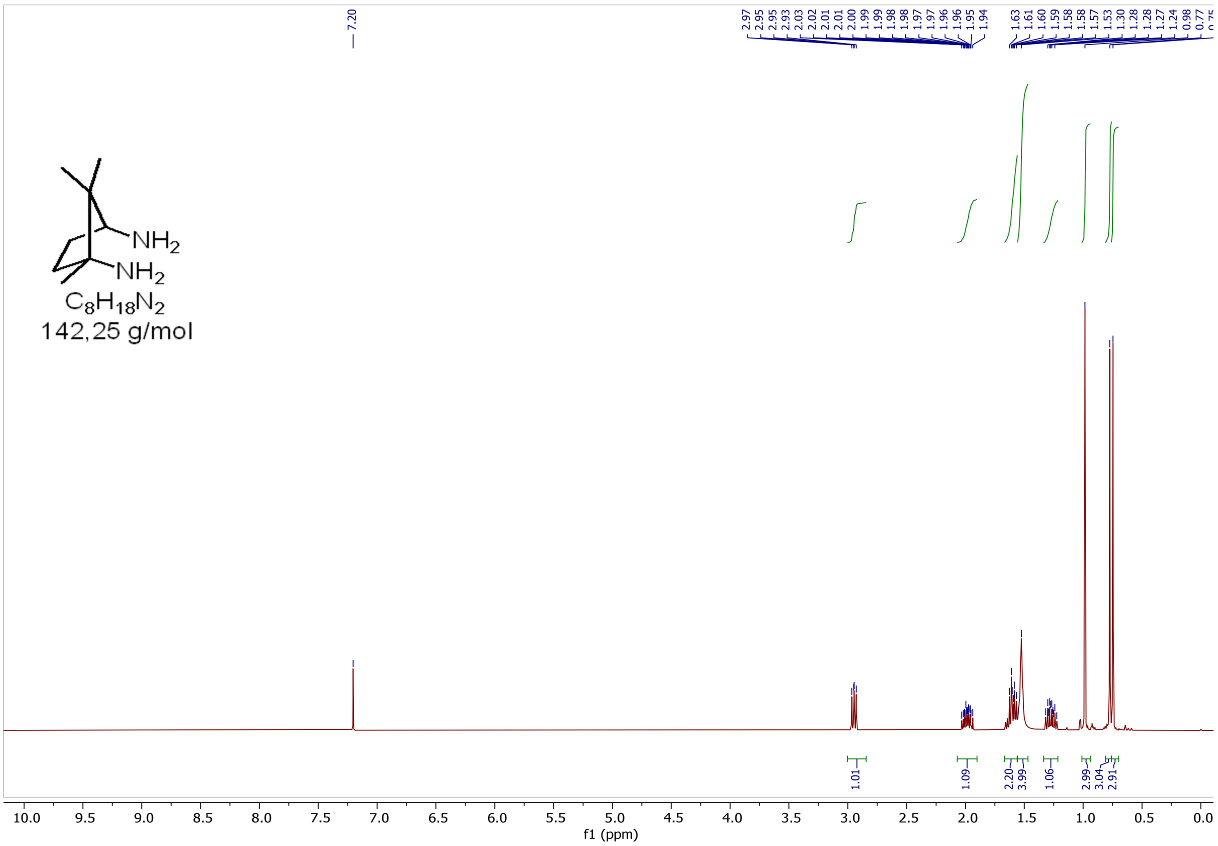

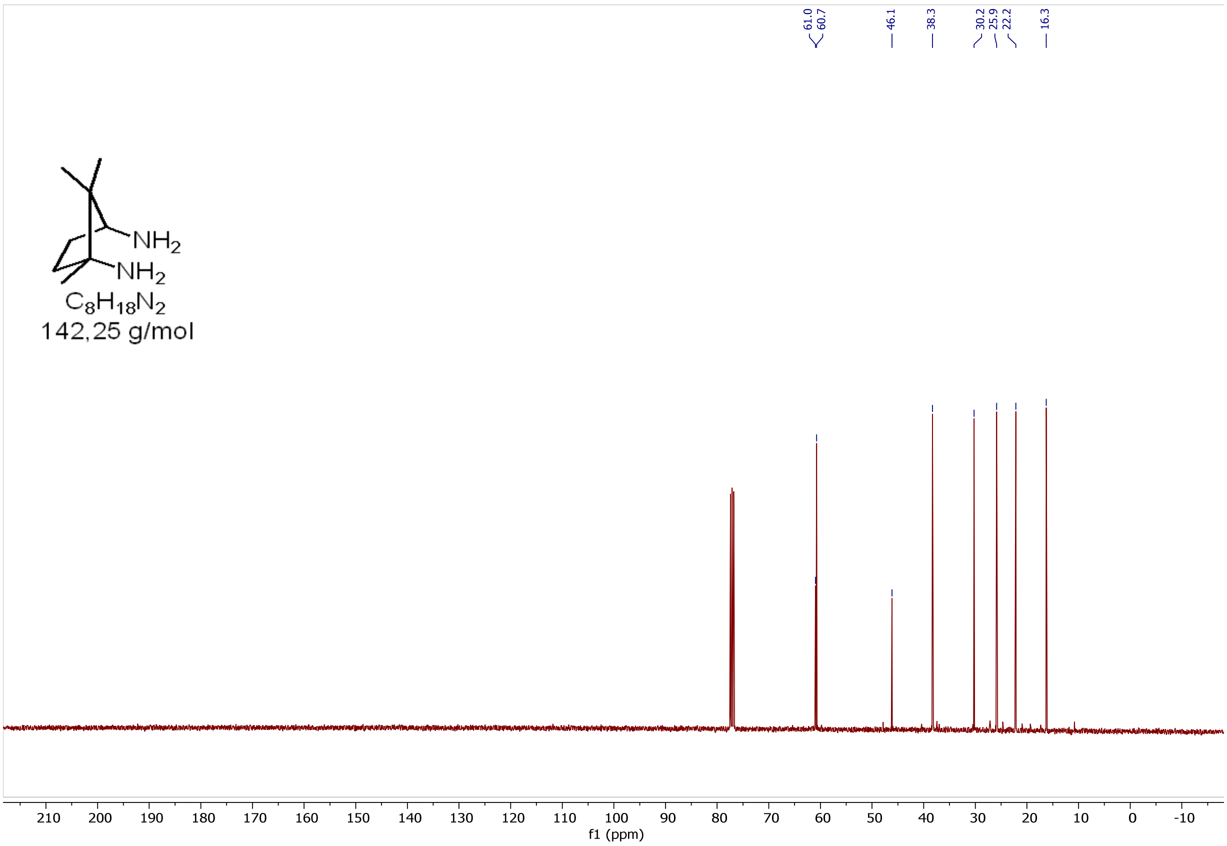

^1H NMR (400 MHz, $CDCl_3$) and ^{13}C NMR (101 MHz, $CDCl_3$) Analysis of Compound **3a**

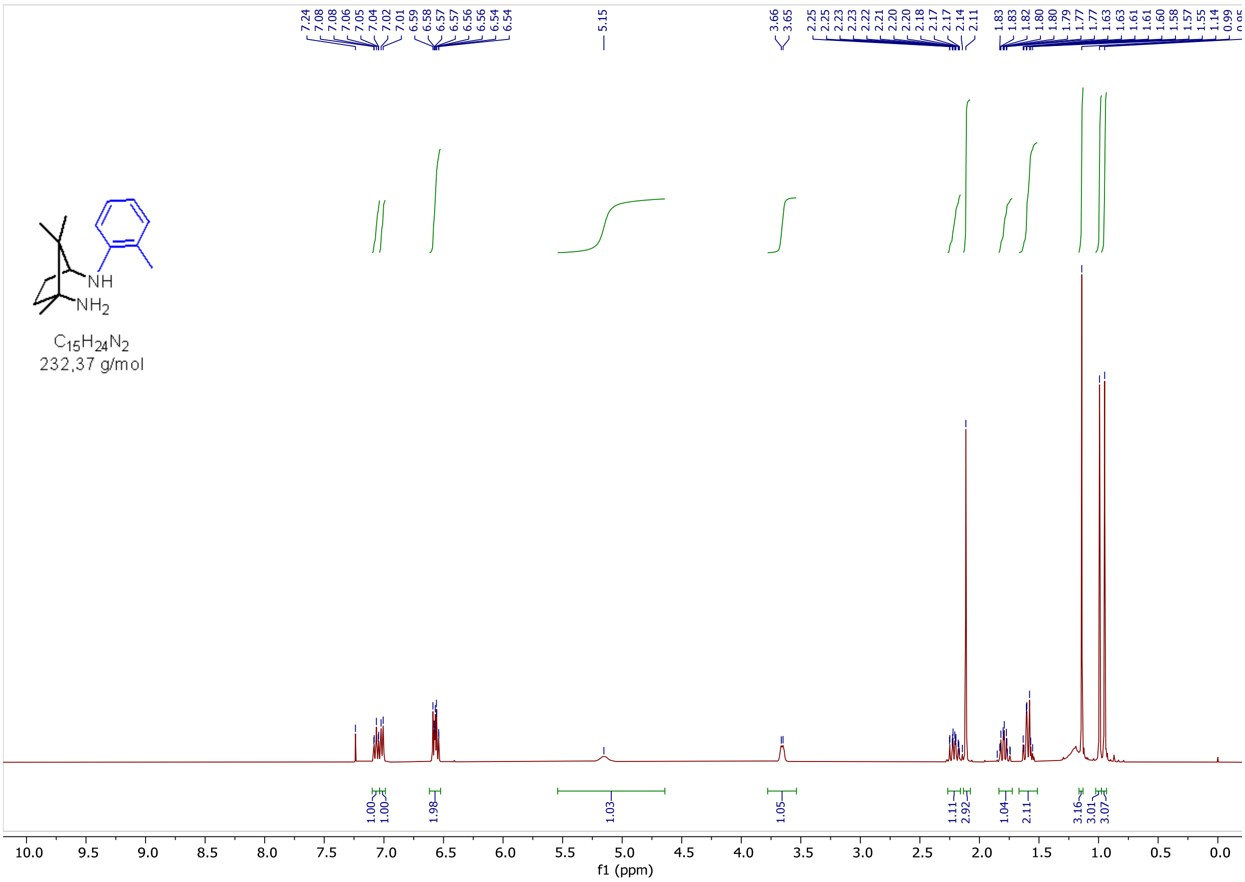

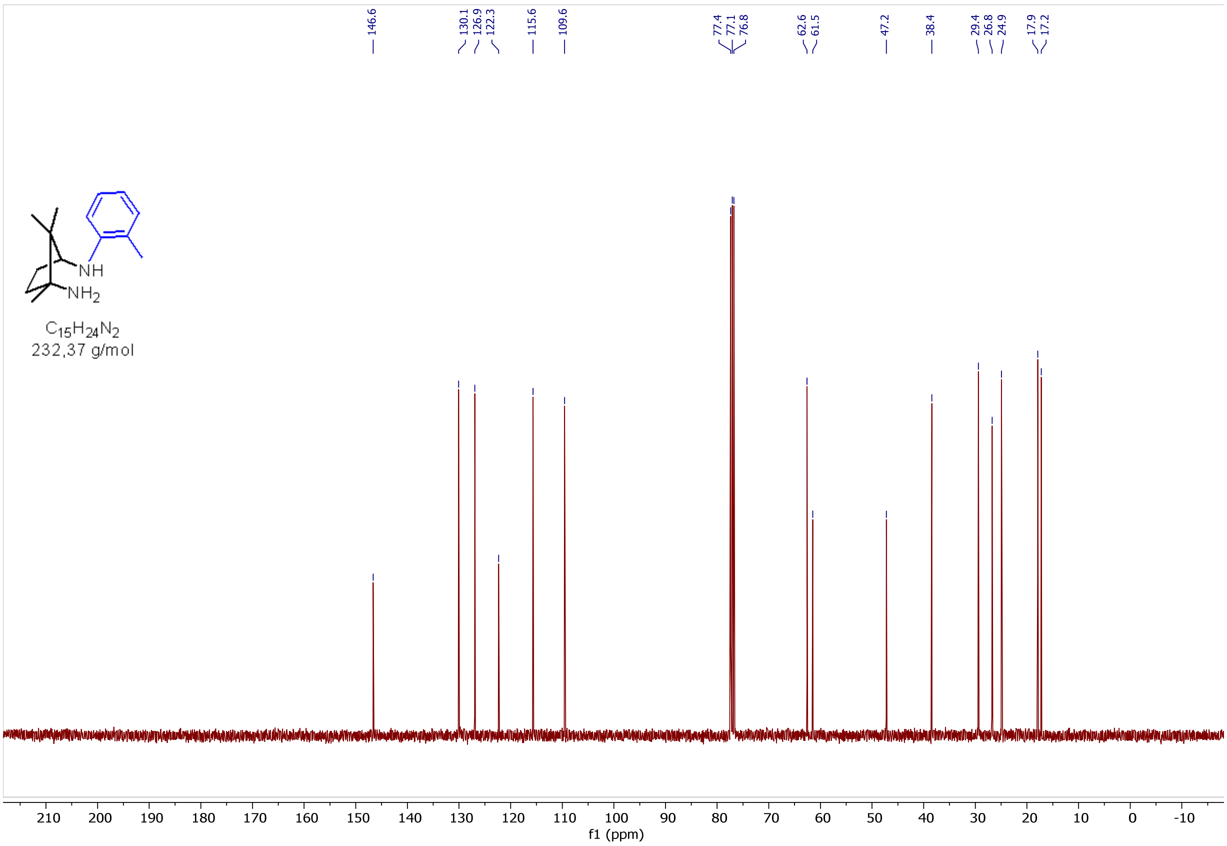

^{1}H NMR (400 MHz, $CDCl_3$) and ^{13}C NMR (101 MHz, $CDCl_3$) Analysis of Compound **3b**

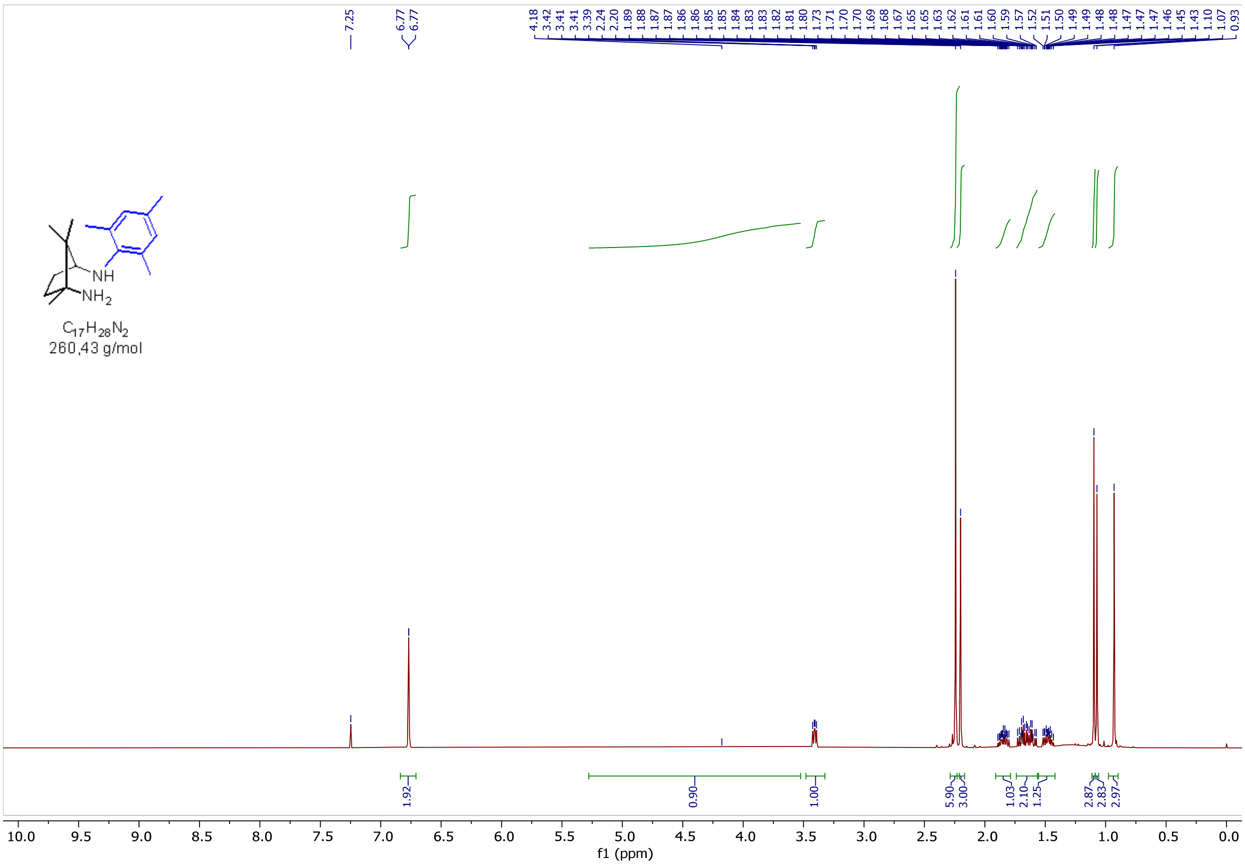

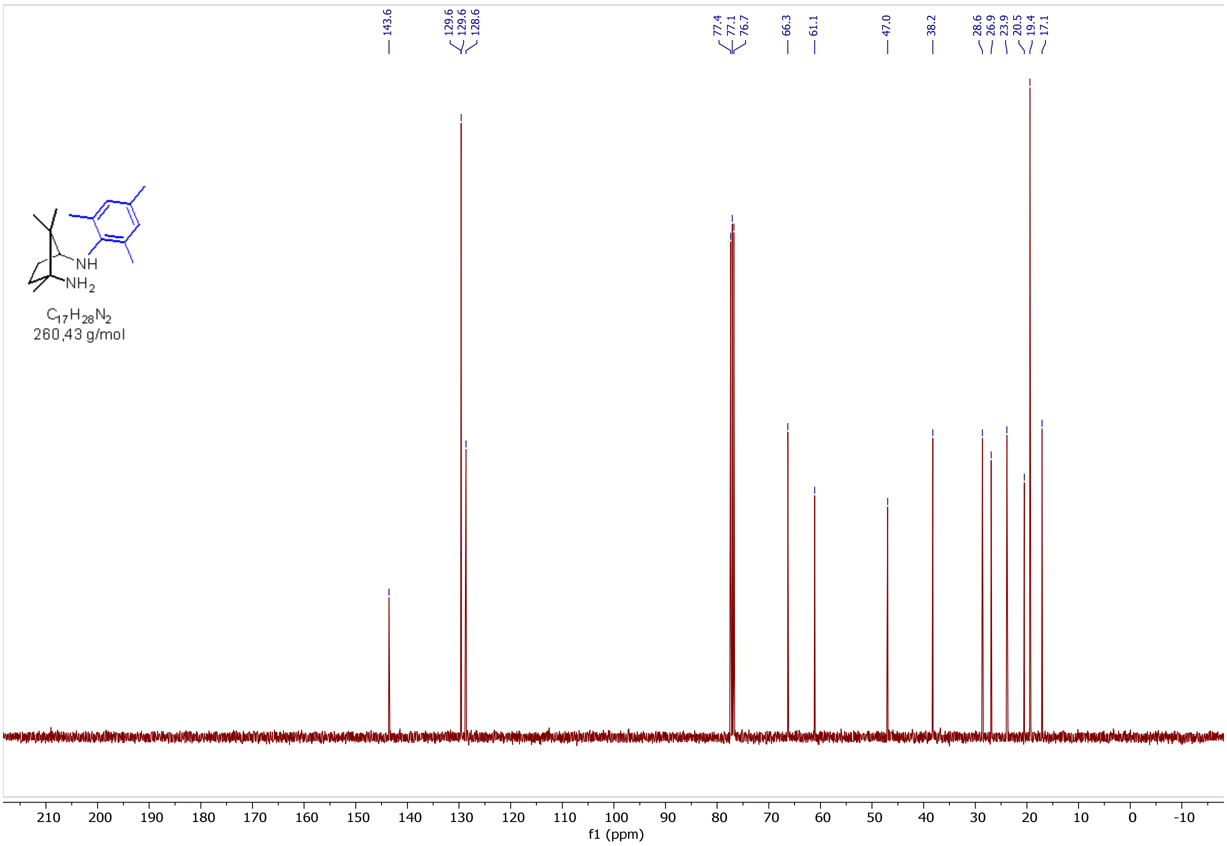

^{1}H NMR (600 MHz, $CDCl_3$), ^{13}C NMR (151 MHz, $CDCl_3$) and ^{15}N HSQC NMR (61 MHz, $CDCl_3$) Analysis of Compound **3c**

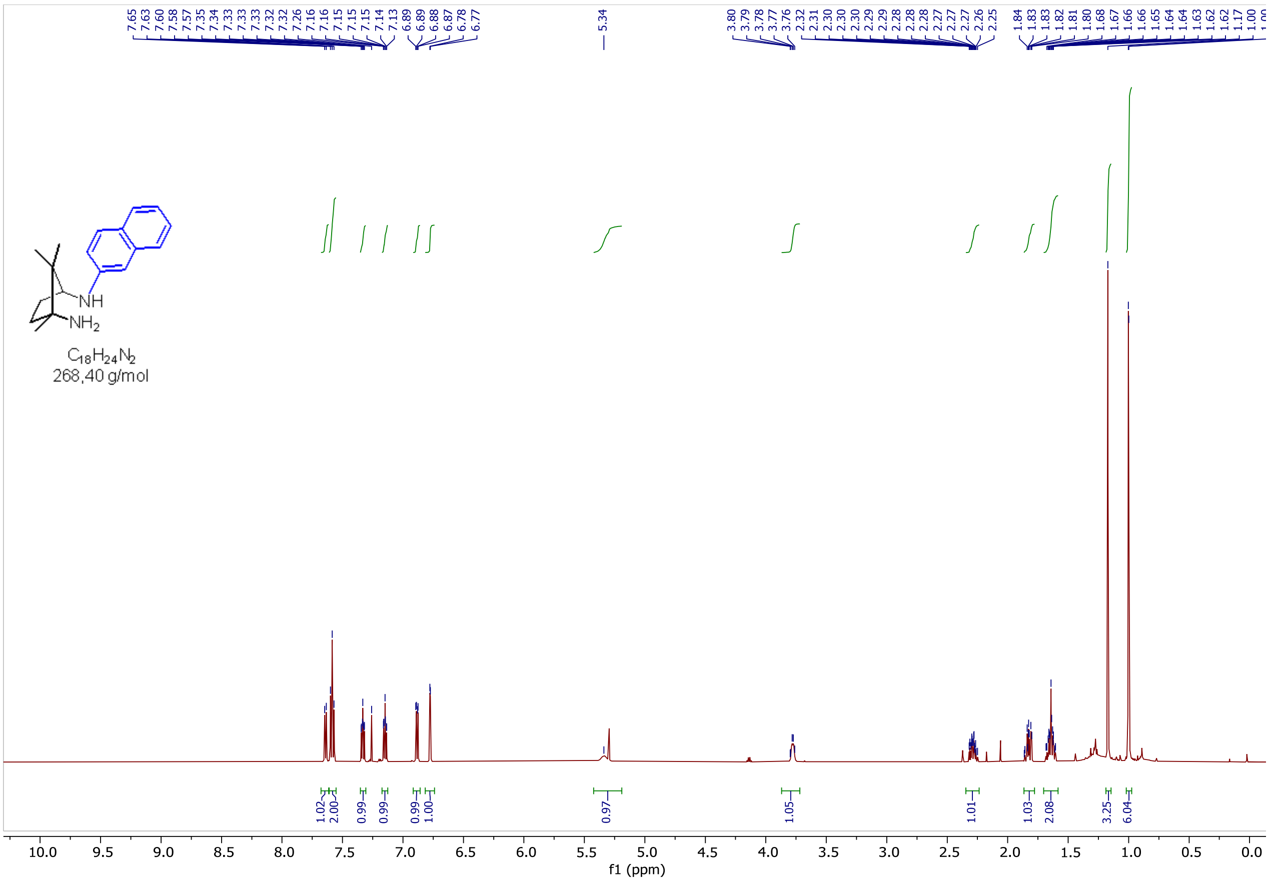

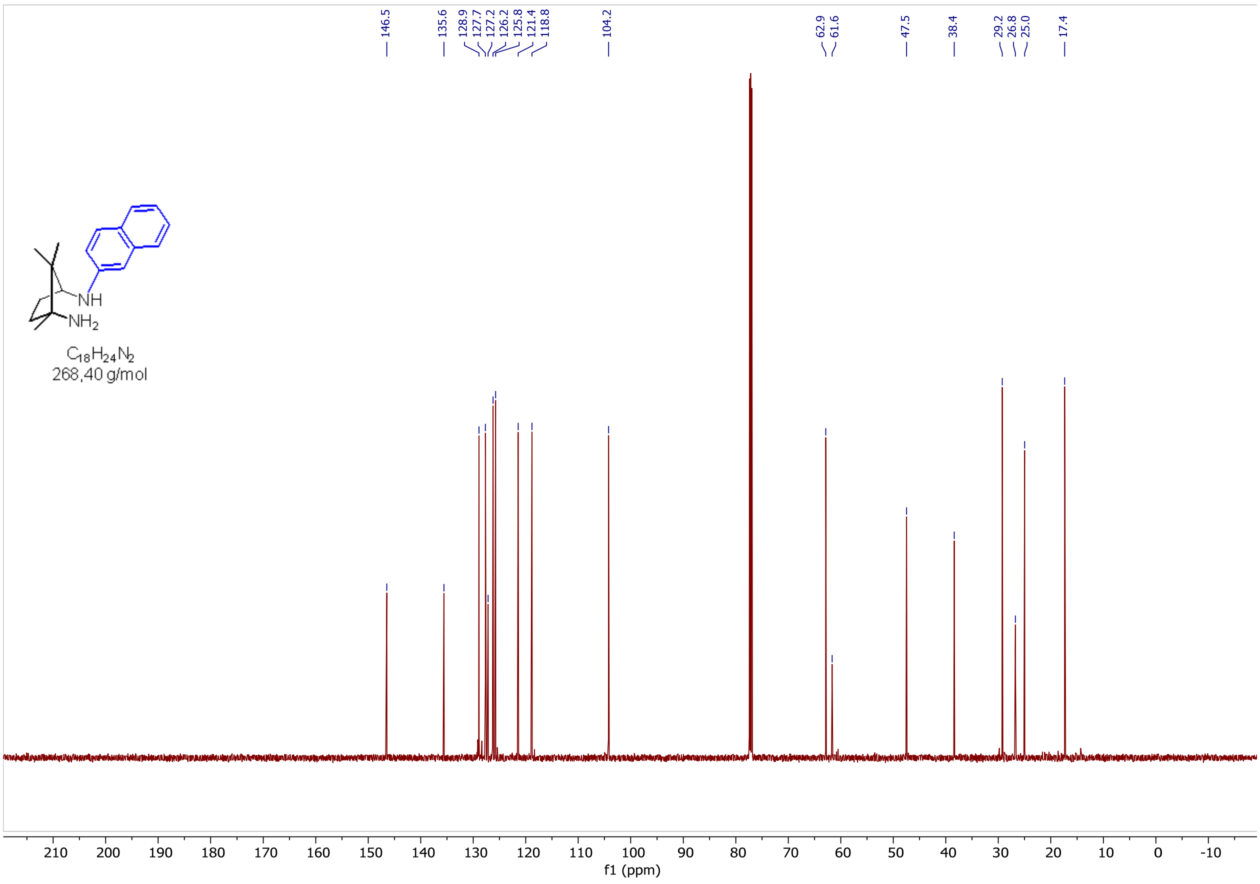

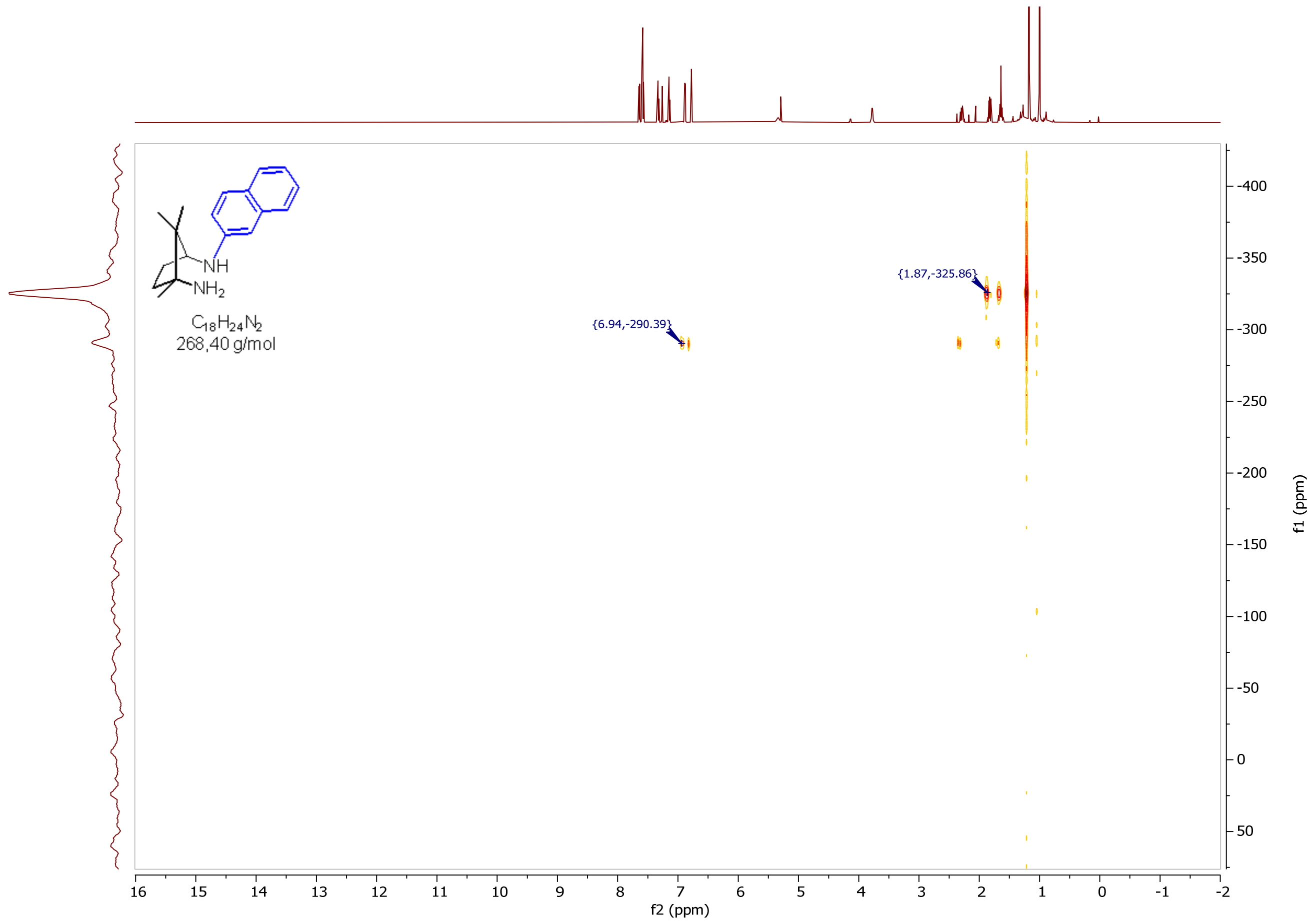
NH
NH2
C18H24N2
268,40 g/mol
{6.94,-290.39}
{1.87,-325.86}
f2 (ppm)
f1 (ppm)

^{1}H NMR (600 MHz, $CDCl_3$), ^{13}C NMR (151 MHz, $CDCl_3$) and ^{15}N HSQC NMR (61 MHz, $CDCl_3$) Analysis of Compound **4a**

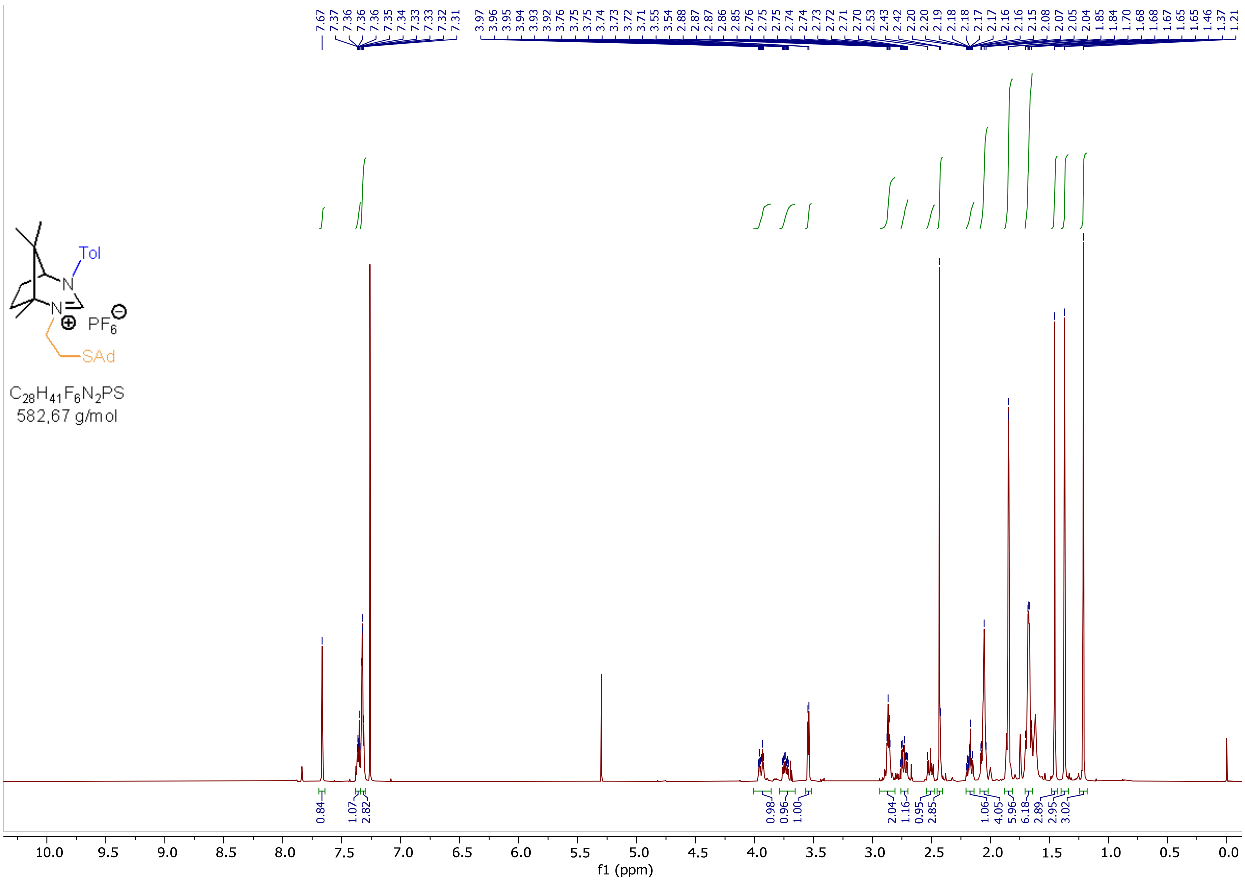

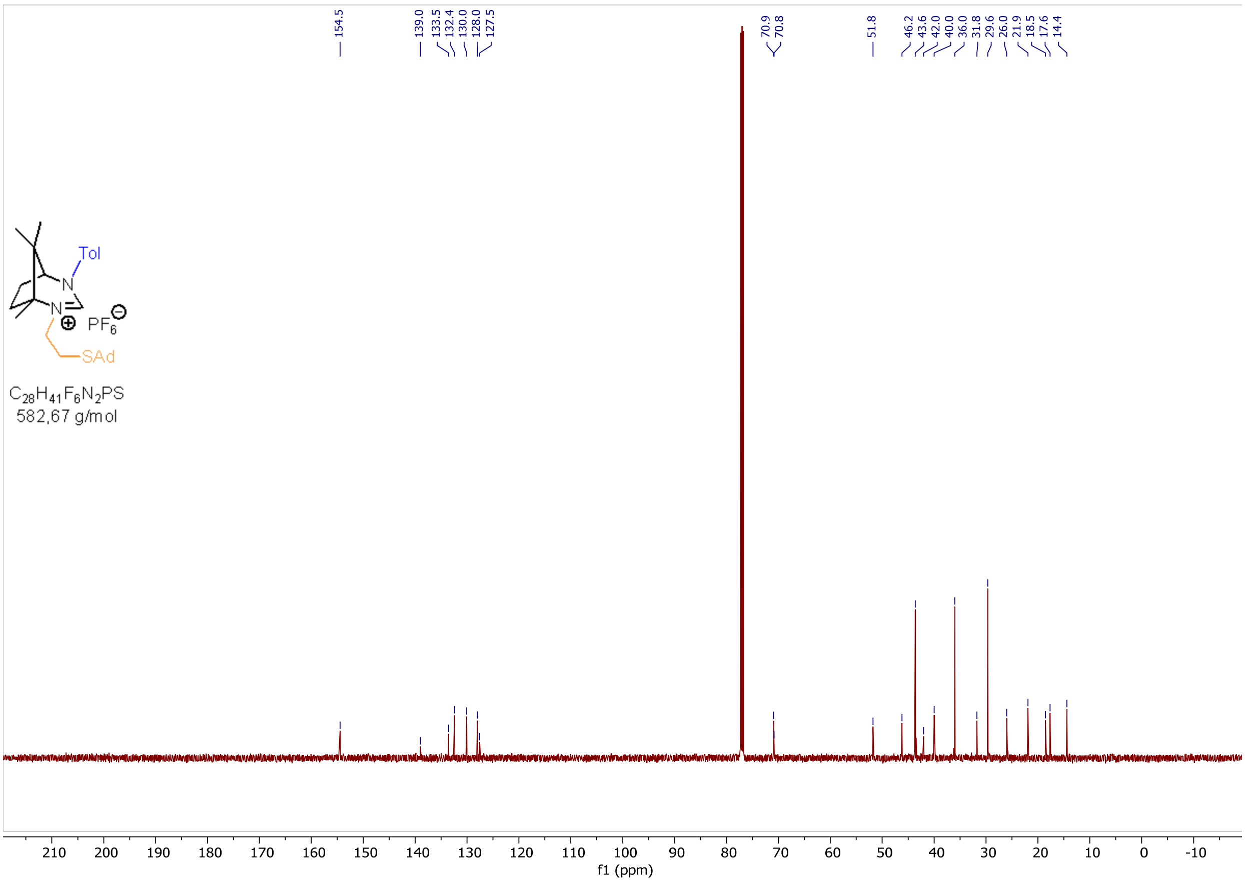

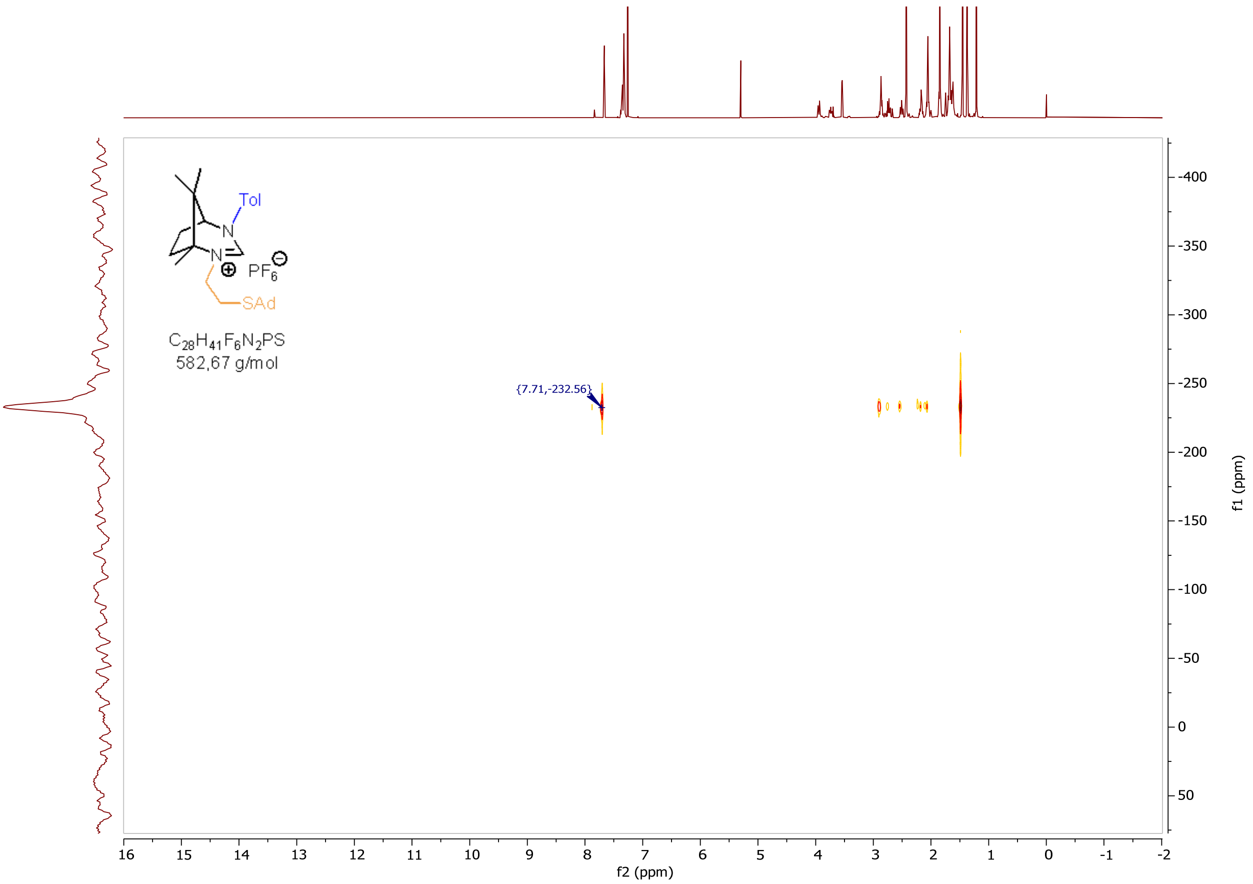

Tol
N
N
PF6
SAd
C28H41F6N2PS
582,67 g/mol
{7.71,-232.56}
f1 (ppm)
f2 (ppm)

^{1}H NMR (600 MHz, $CDCl_3$), ^{13}C NMR (151 MHz, $CDCl_3$) and ^{15}N HSQC NMR (61 MHz, $CDCl_3$)
Analysis of Compound **4b**

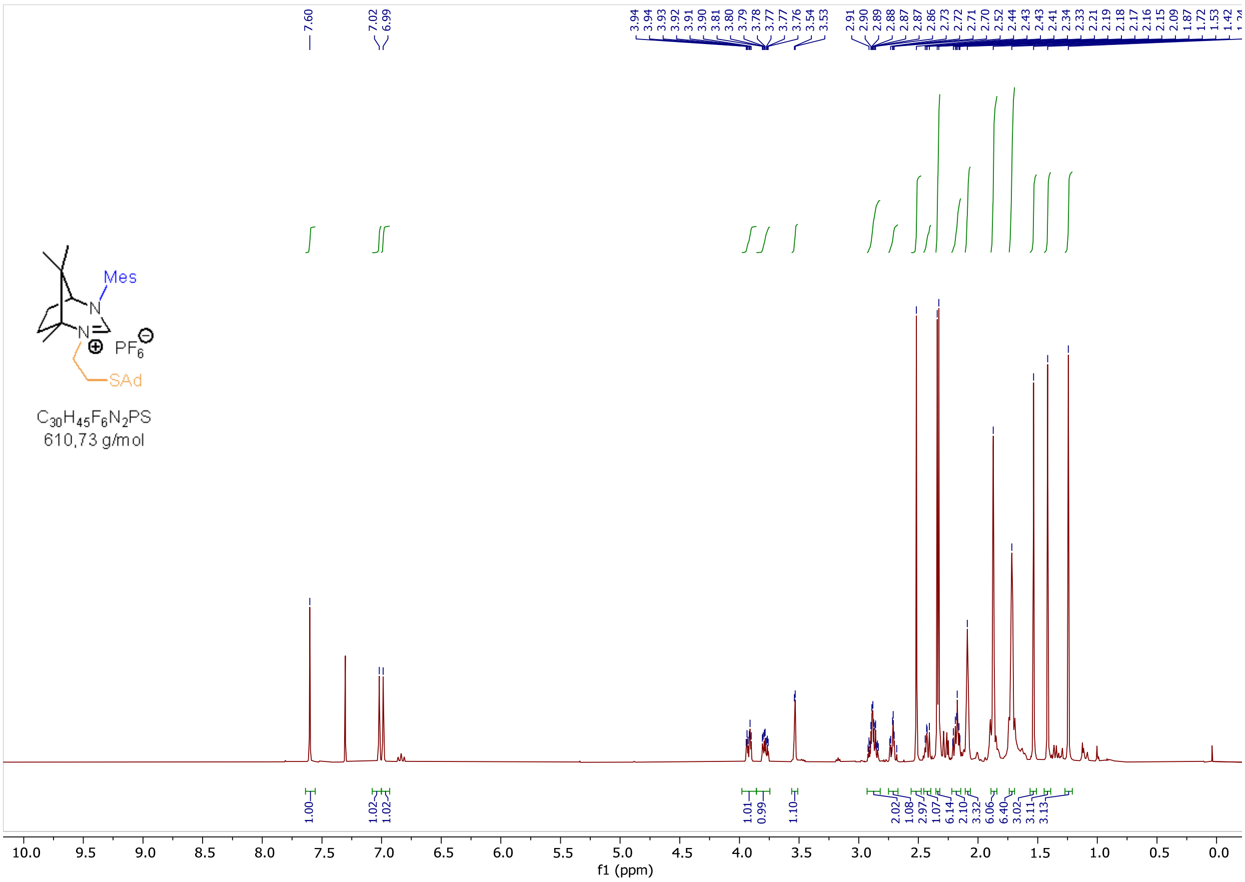

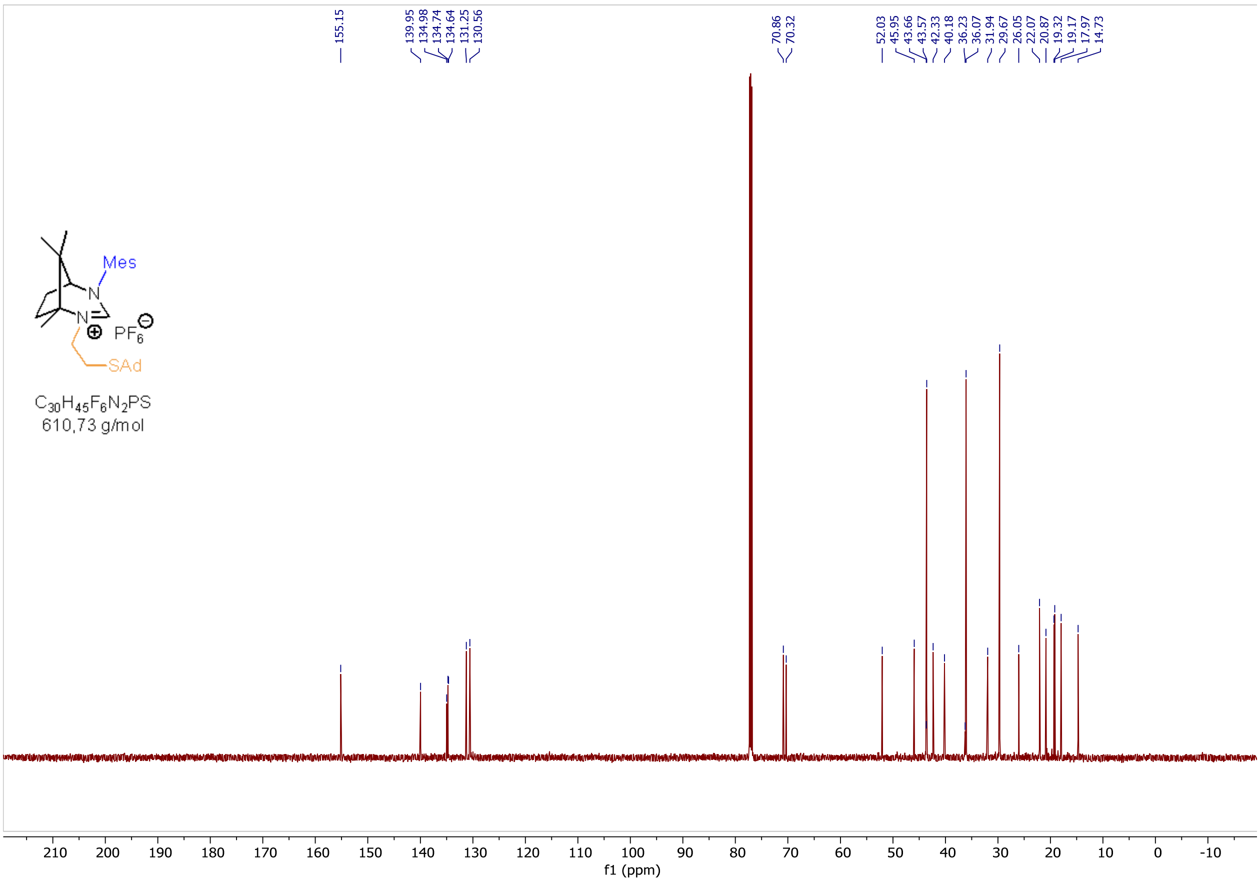

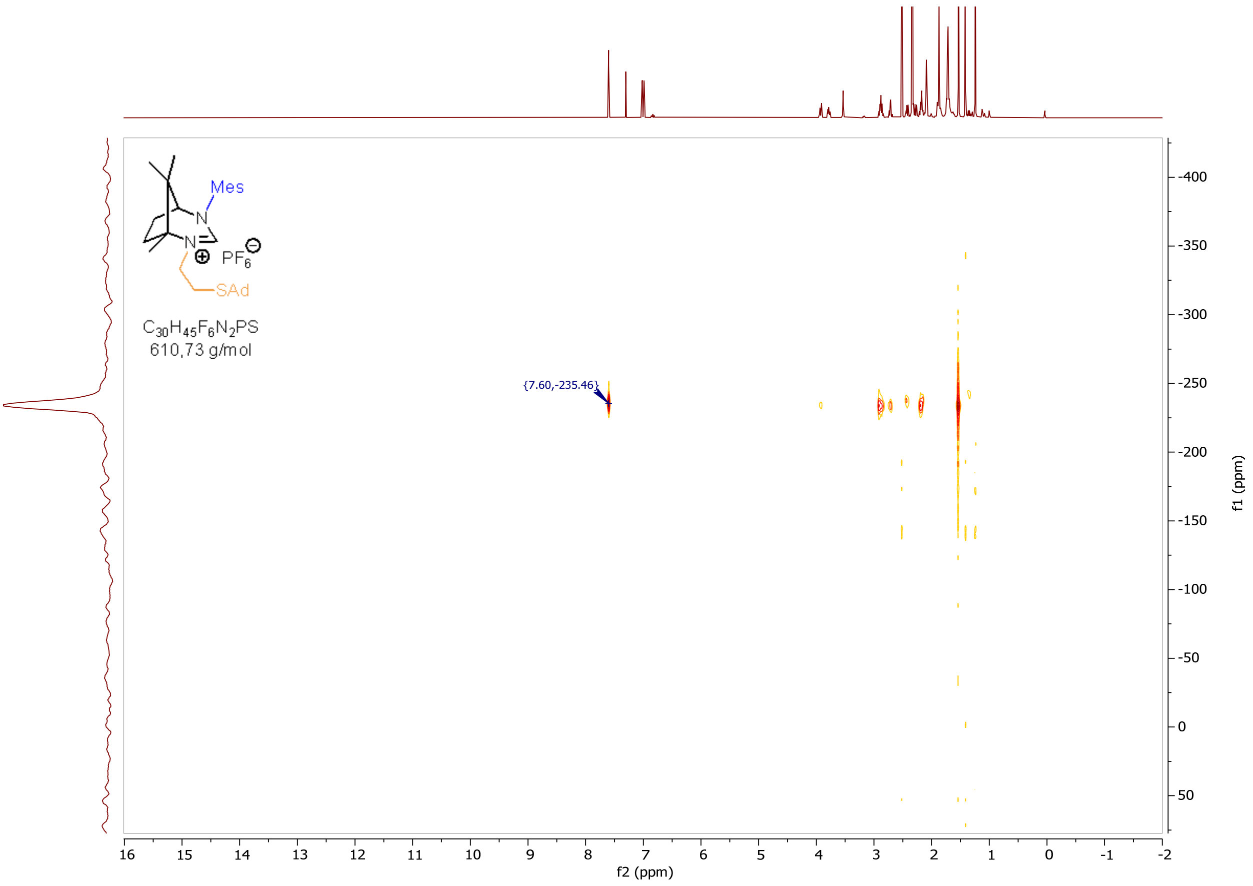

Mes
N
N
PF6
SAd
C30H45F6N2PS
610,73 g/mol
{7.60,-235.46}
f1 (ppm)
f2 (ppm)

^1H NMR (600 MHz, $CDCl_3$), ^{13}C NMR (151 MHz, $CDCl_3$) and ^{15}N HSQC NMR (61 MHz, $CDCl_3$) Analysis of Compound **4c**

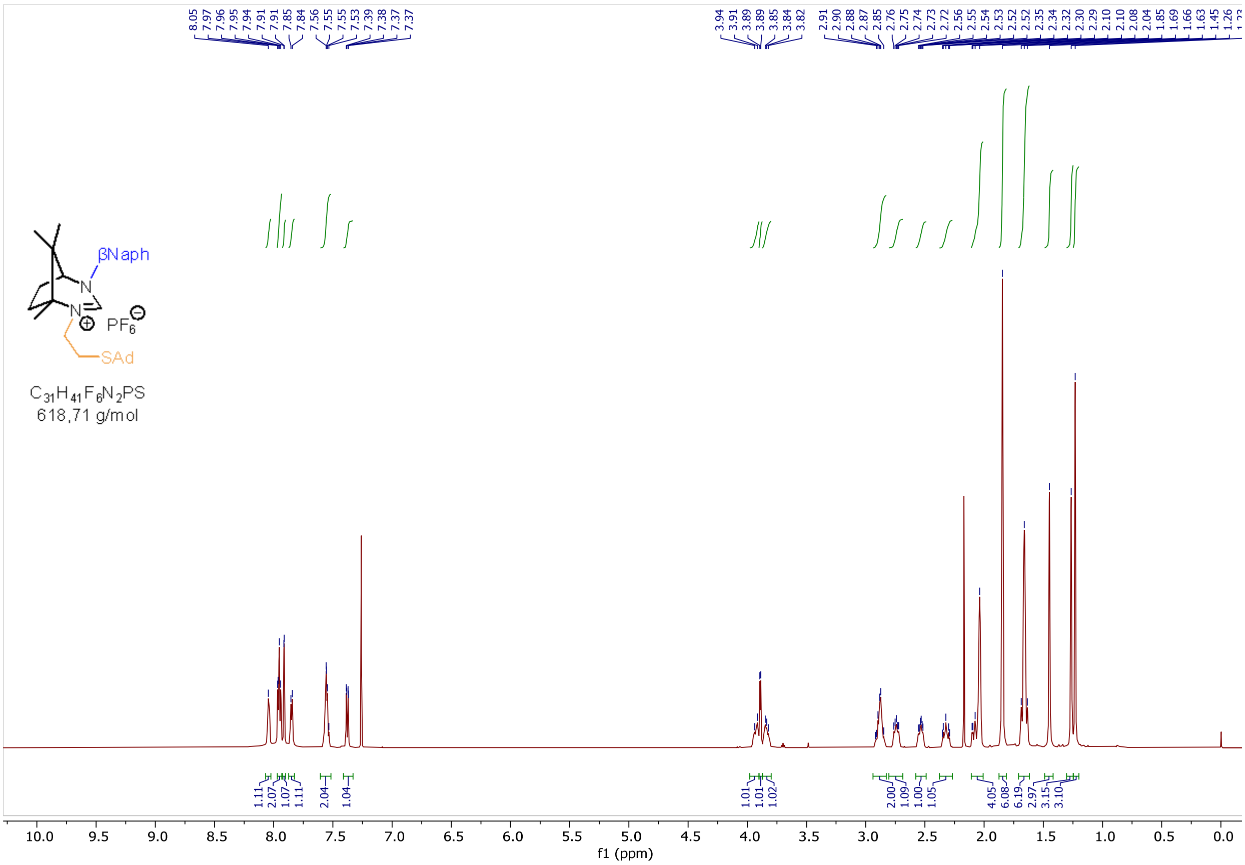

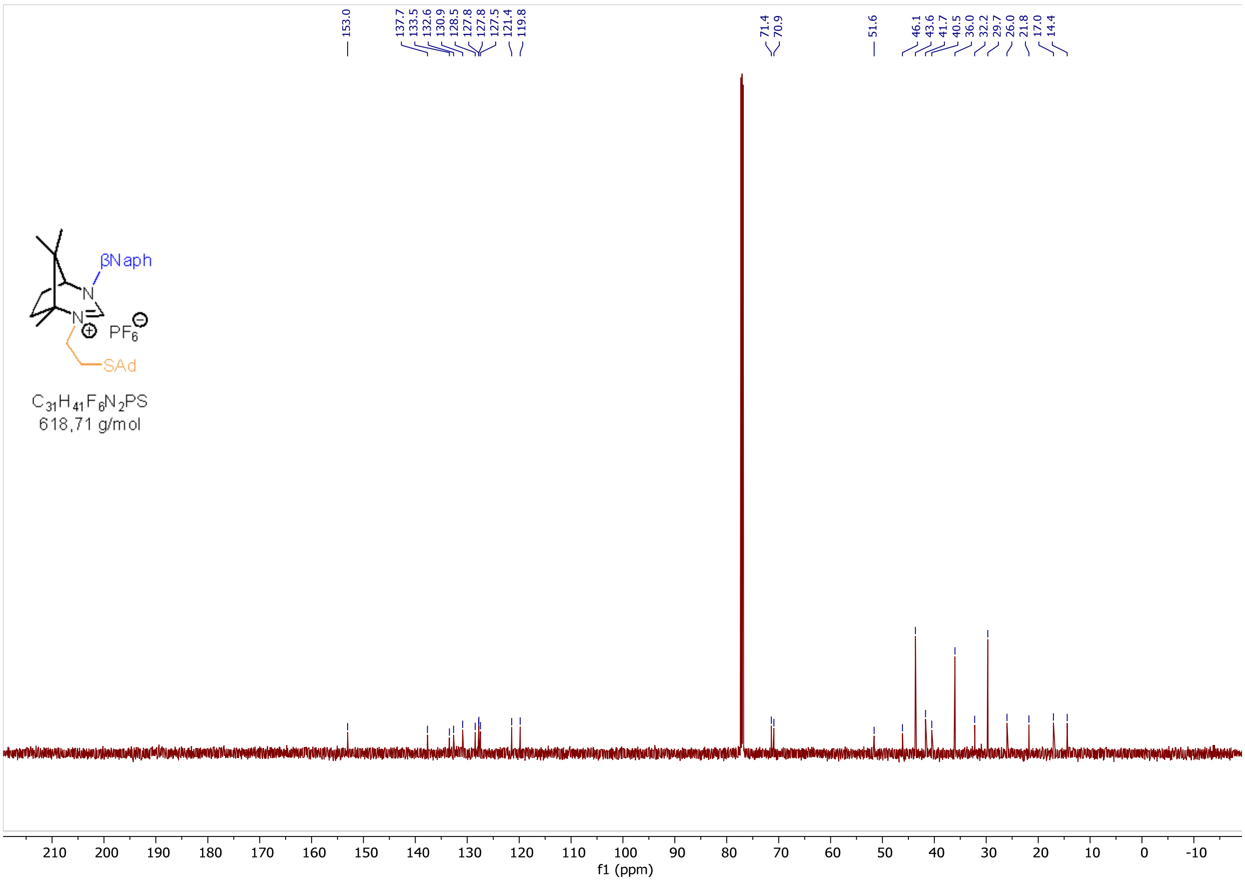

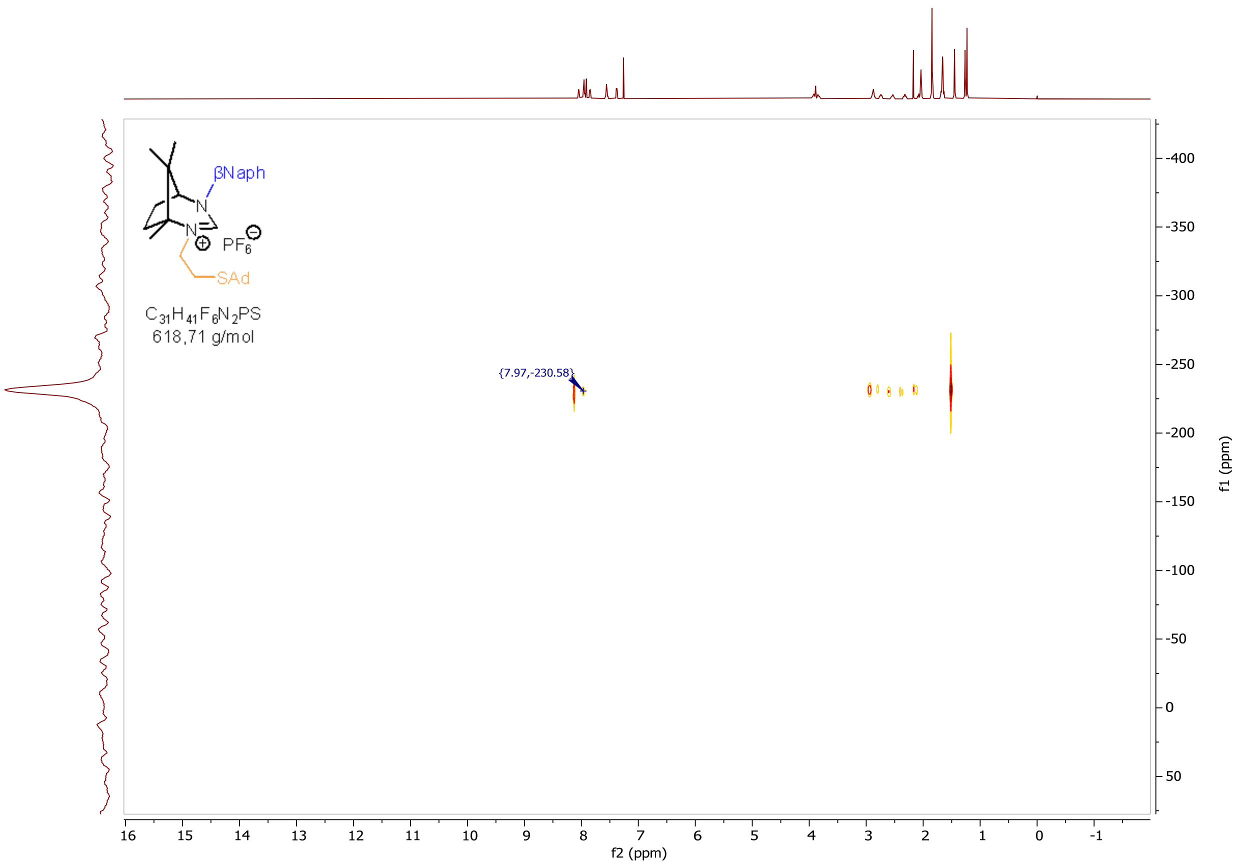

βNaph
PF6
SAd
C31H41F6N2PS
618,71 g/mol
{7.97,-230.58}
f1 (ppm)
f2 (ppm)

^1H NMR (600 MHz, $CDCl_3$), ^{13}C NMR (151 MHz, $CDCl_3$) and ^{15}N HSQC NMR (61 MHz, $CDCl_3$) Analysis of Compound **4d**

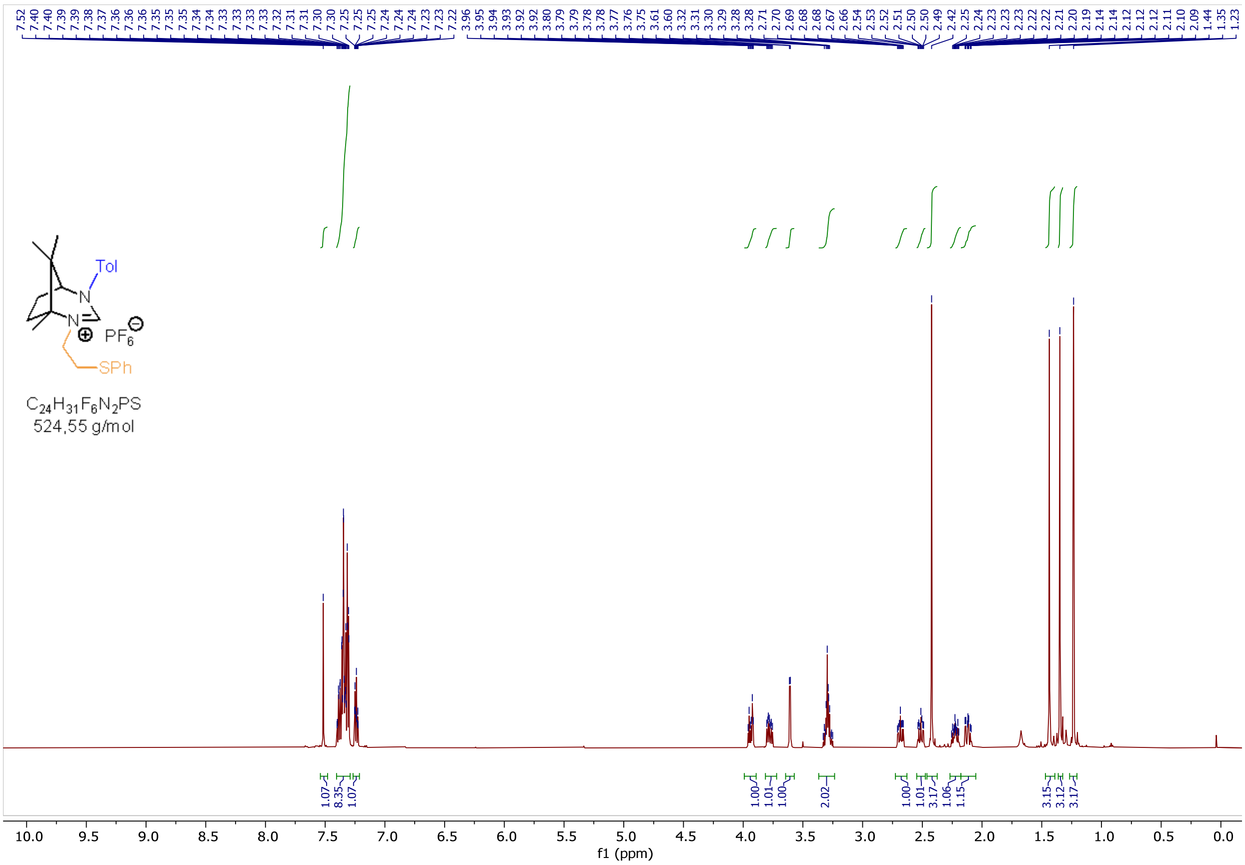

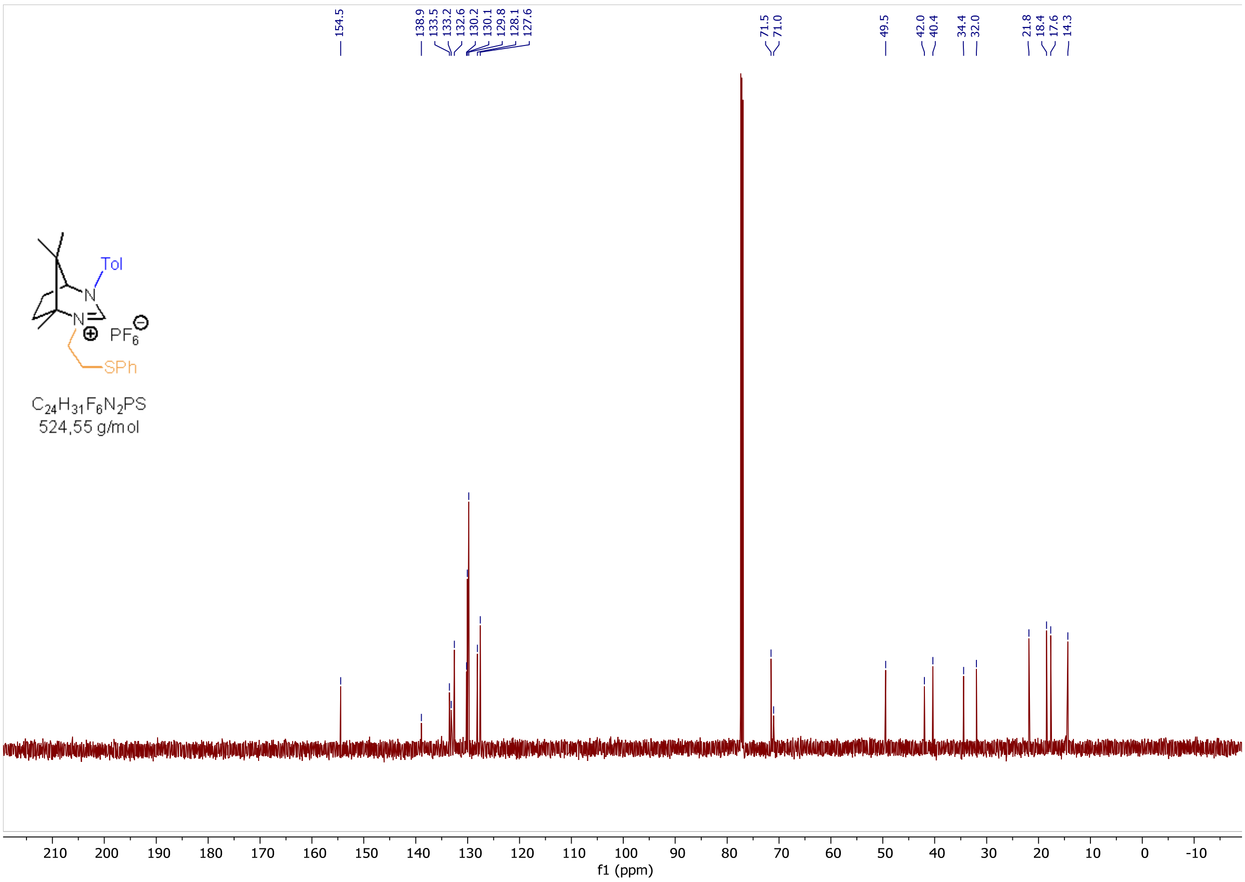

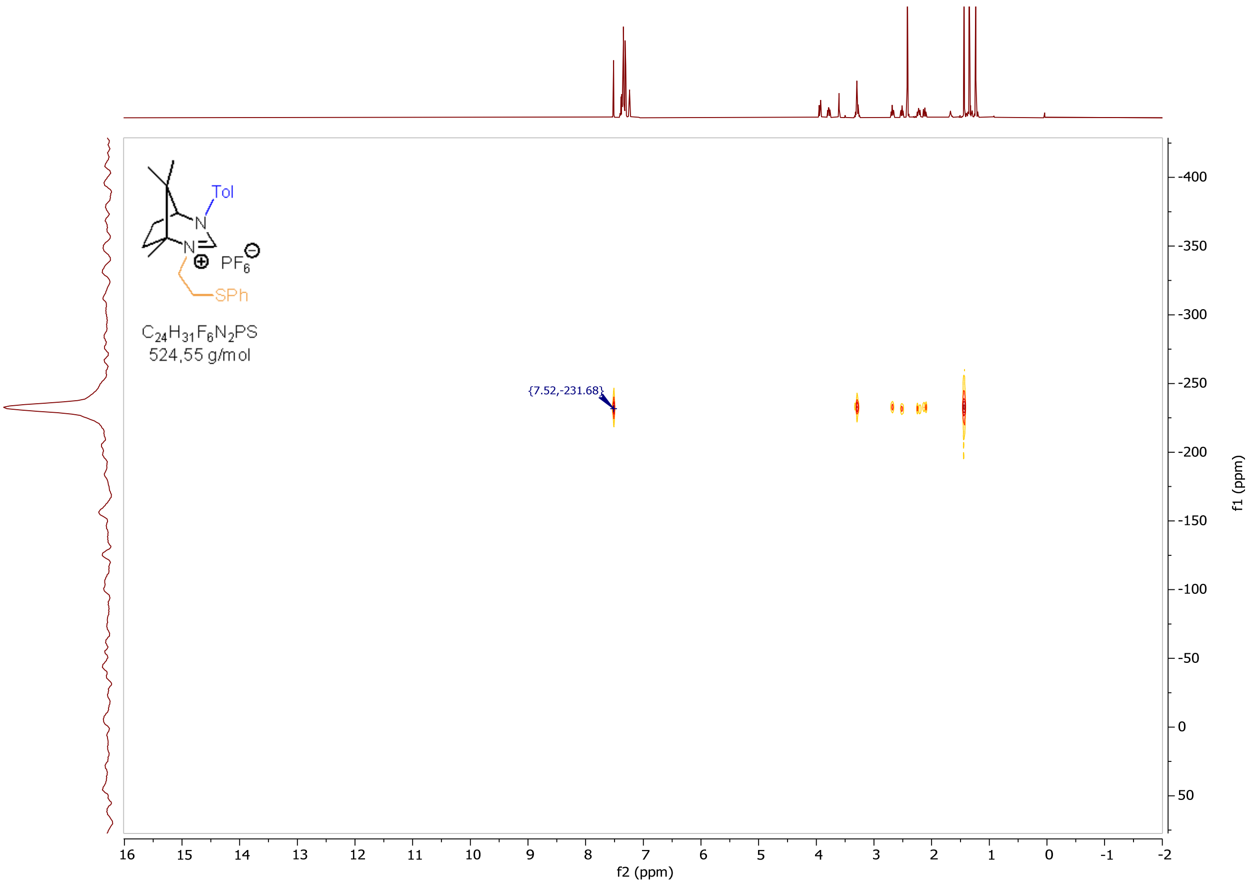
Tol
N
N
PF6
SPh
C24H31F6N2PS
524,55 g/mol
{7.52,-231.68}
f2 (ppm)
f1 (ppm)

^1H NMR (600 MHz, $CDCl_3$), ^{13}C NMR (151 MHz, $CDCl_3$) and ^{15}N HSQC NMR (61 MHz, $CDCl_3$) Analysis of Compound **4e**

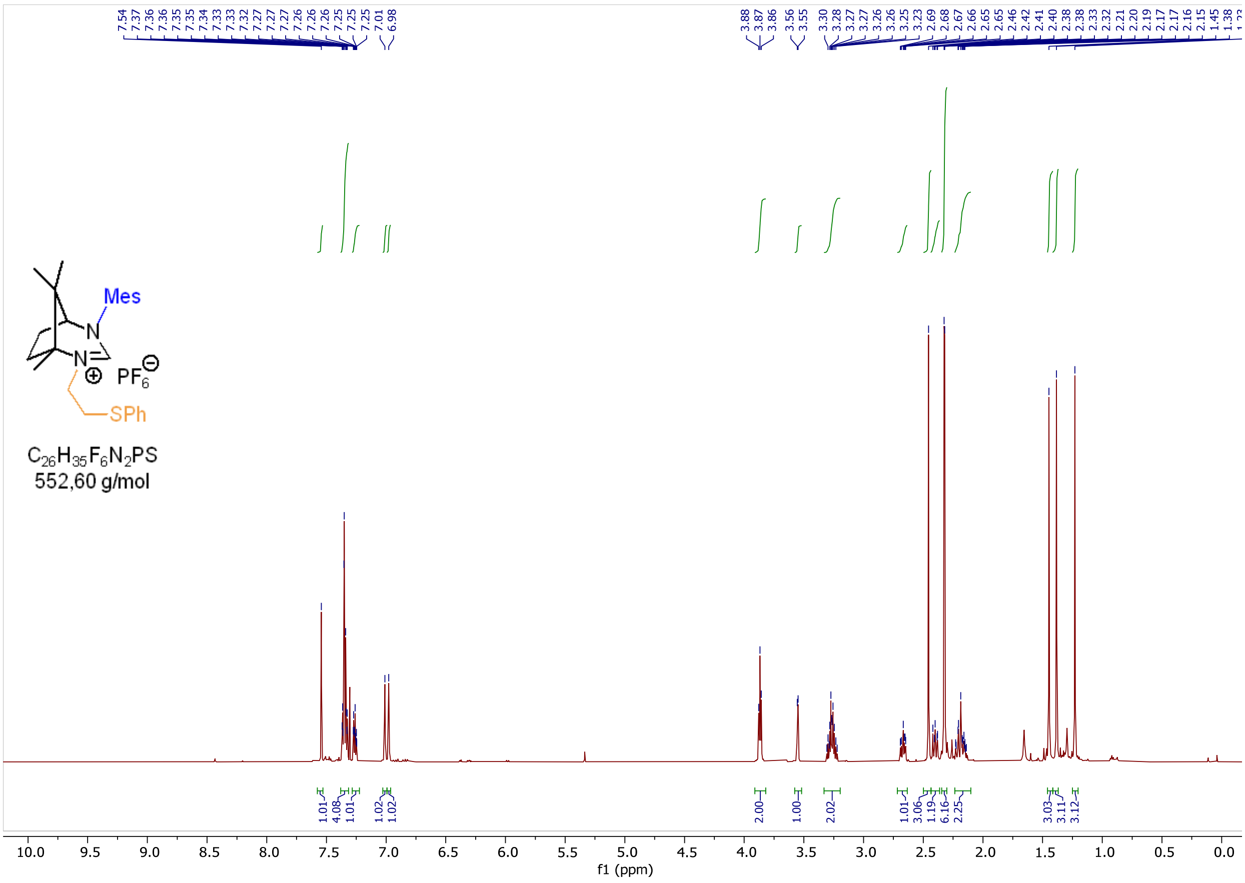

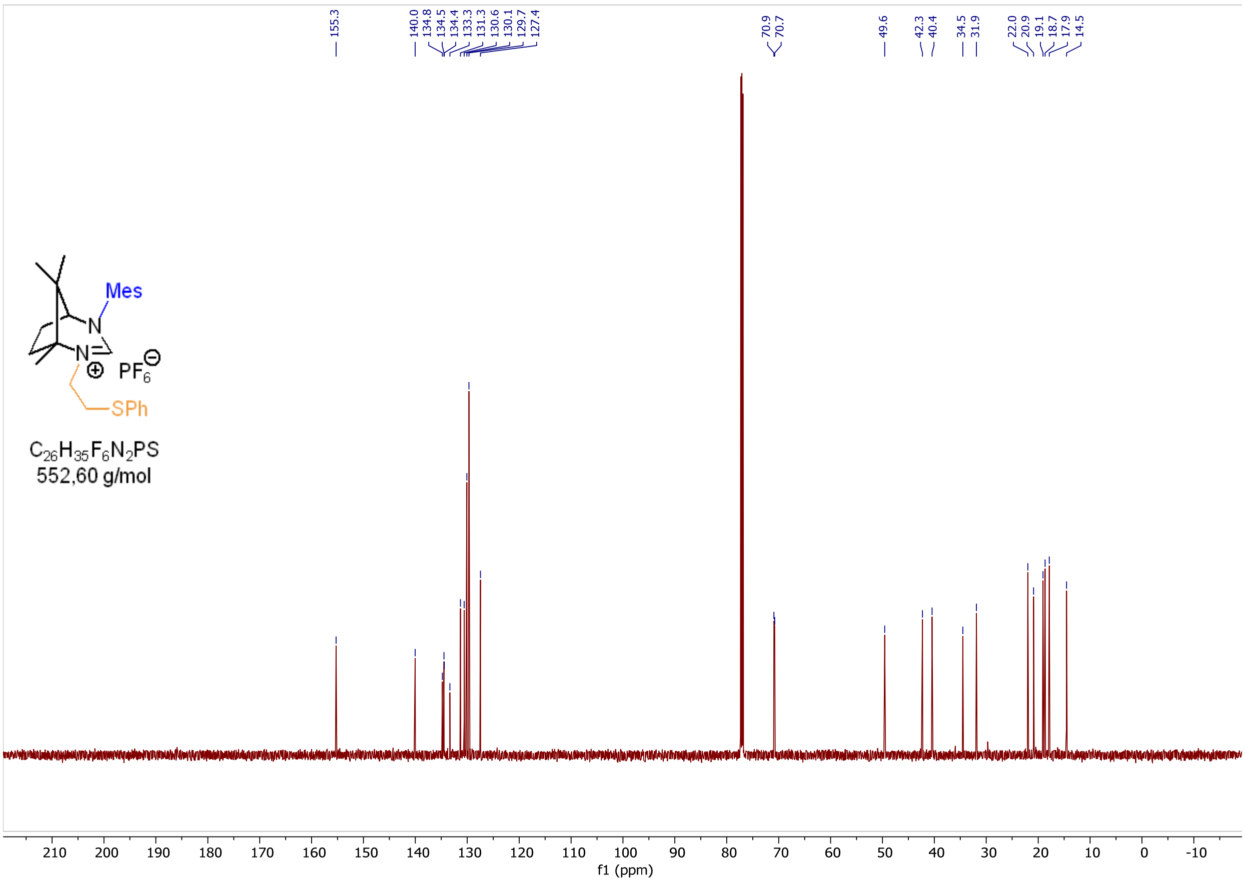

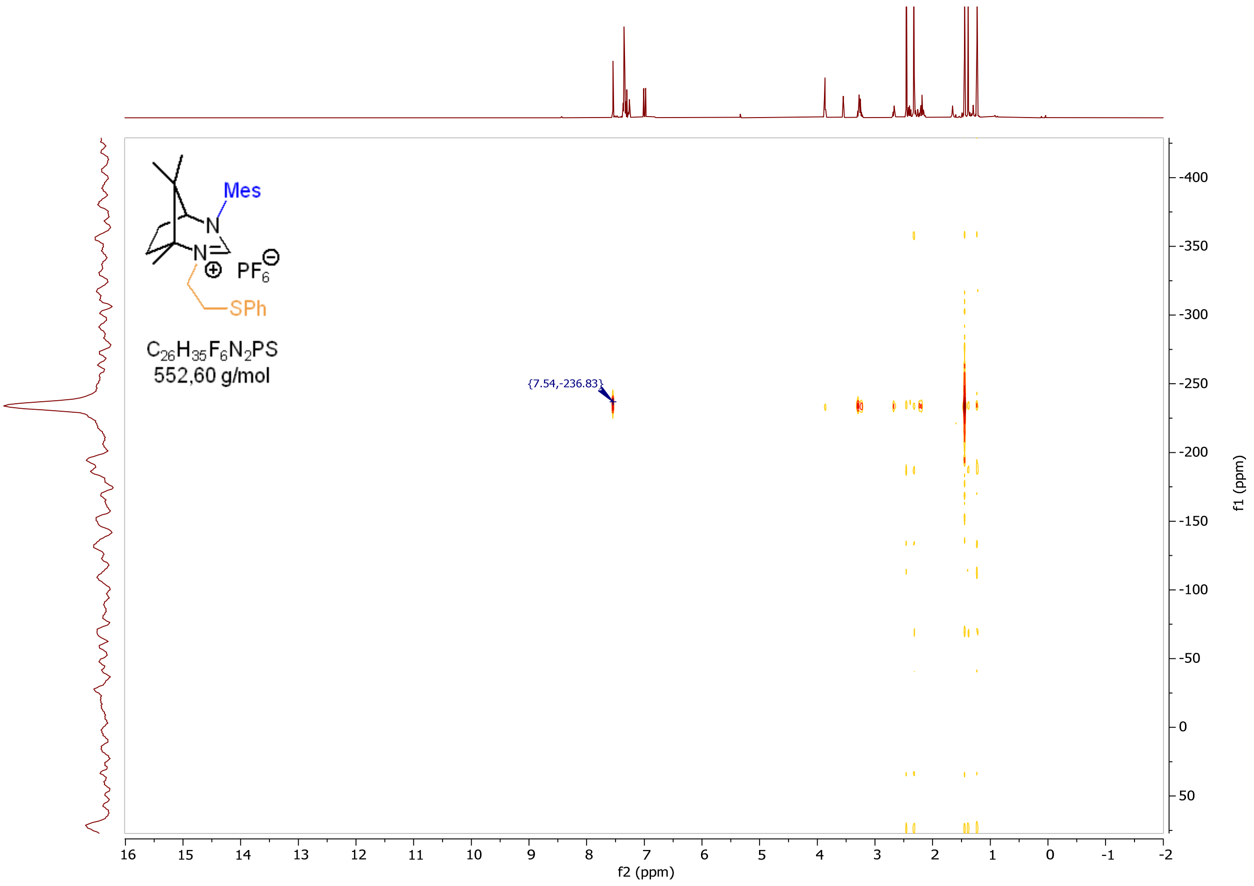
Mes
N
N
PF6
SPh
C26H35F6N2PS
552,60 g/mol
{7.54,-236.83}
f2 (ppm)
f1 (ppm)

^{1}H NMR (600 MHz, $CDCl_3$), ^{13}C NMR (151 MHz, $CDCl_3$) and ^{15}N HSQC NMR (61 MHz, $CDCl_3$) Analysis of Compound **4f**

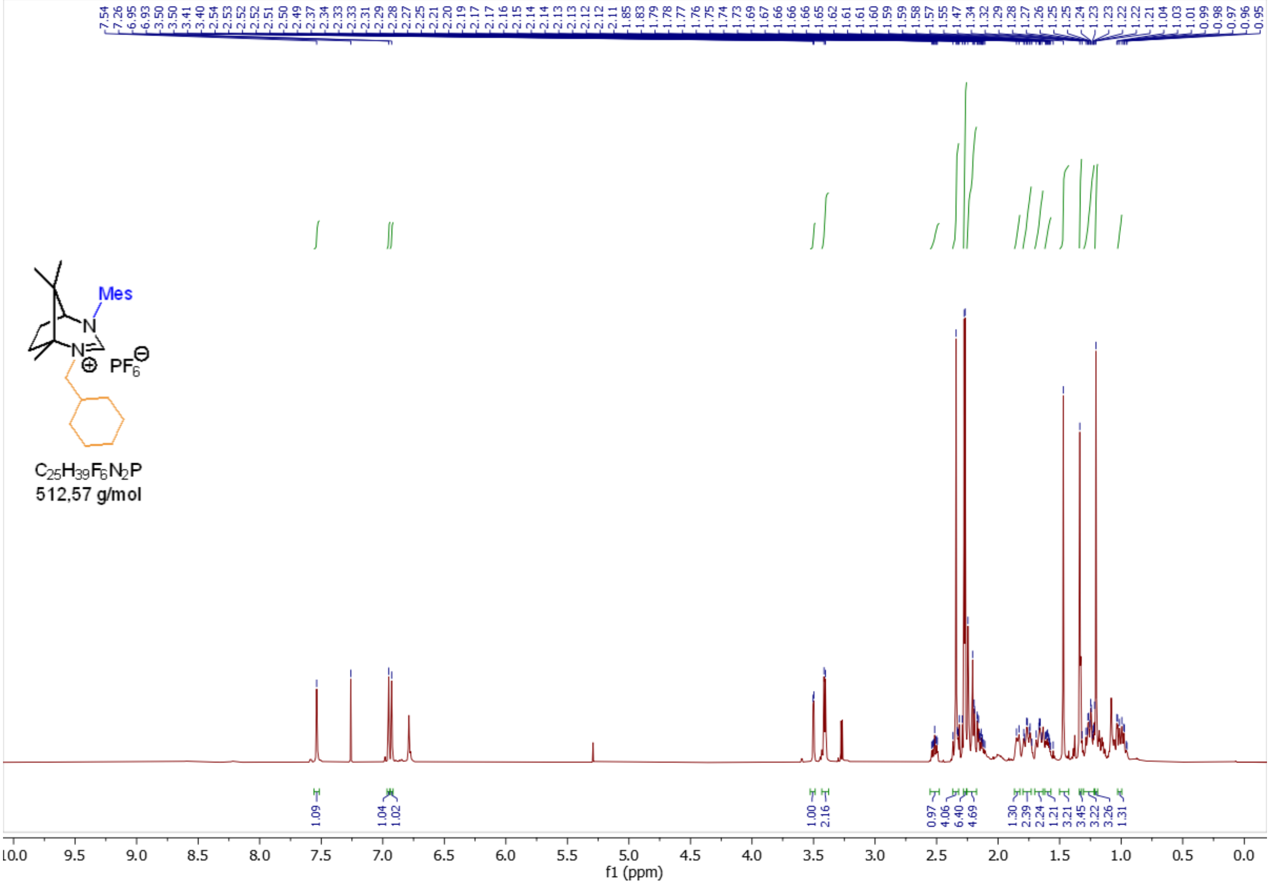

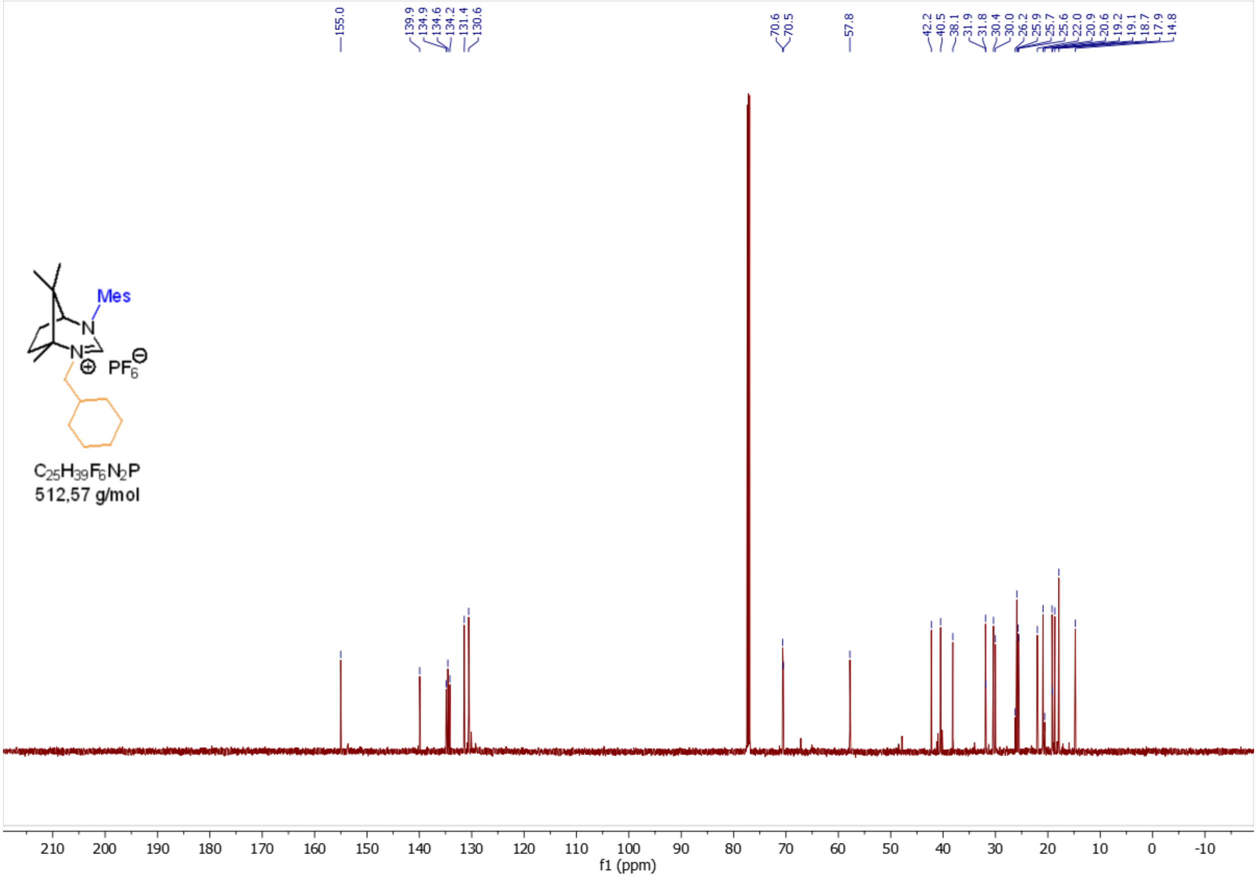

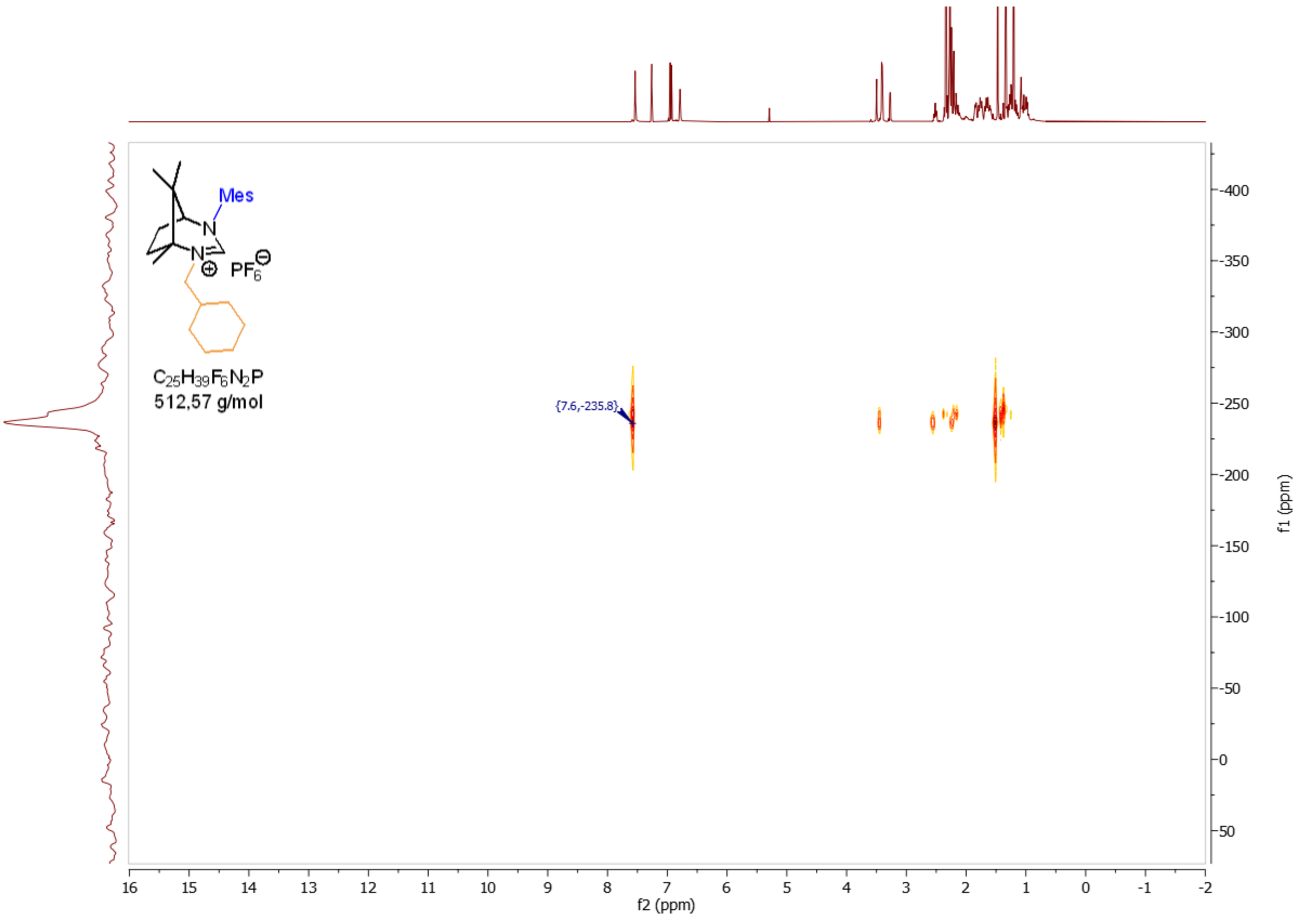
Mes
PF6
C25H39F6N2P
512,57 g/mol
{7.6,-235.8}
f2 (ppm)
f1 (ppm)

^{1}H NMR (600 MHz, $CDCl_3$), ^{13}C NMR (151 MHz, $CDCl_3$) and ^{15}N HSQC NMR (61 MHz, $CDCl_3$)Analysis of Compound **Rh5a**

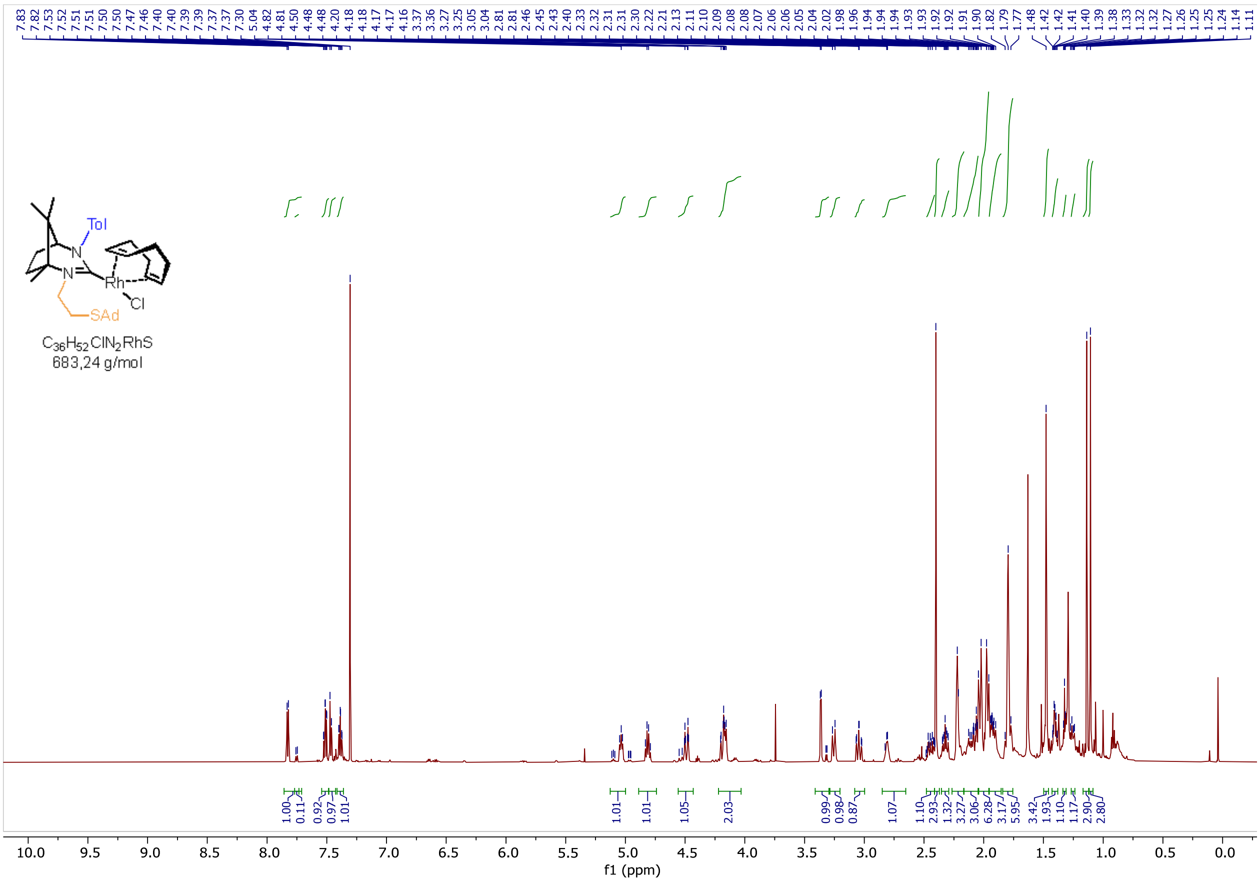

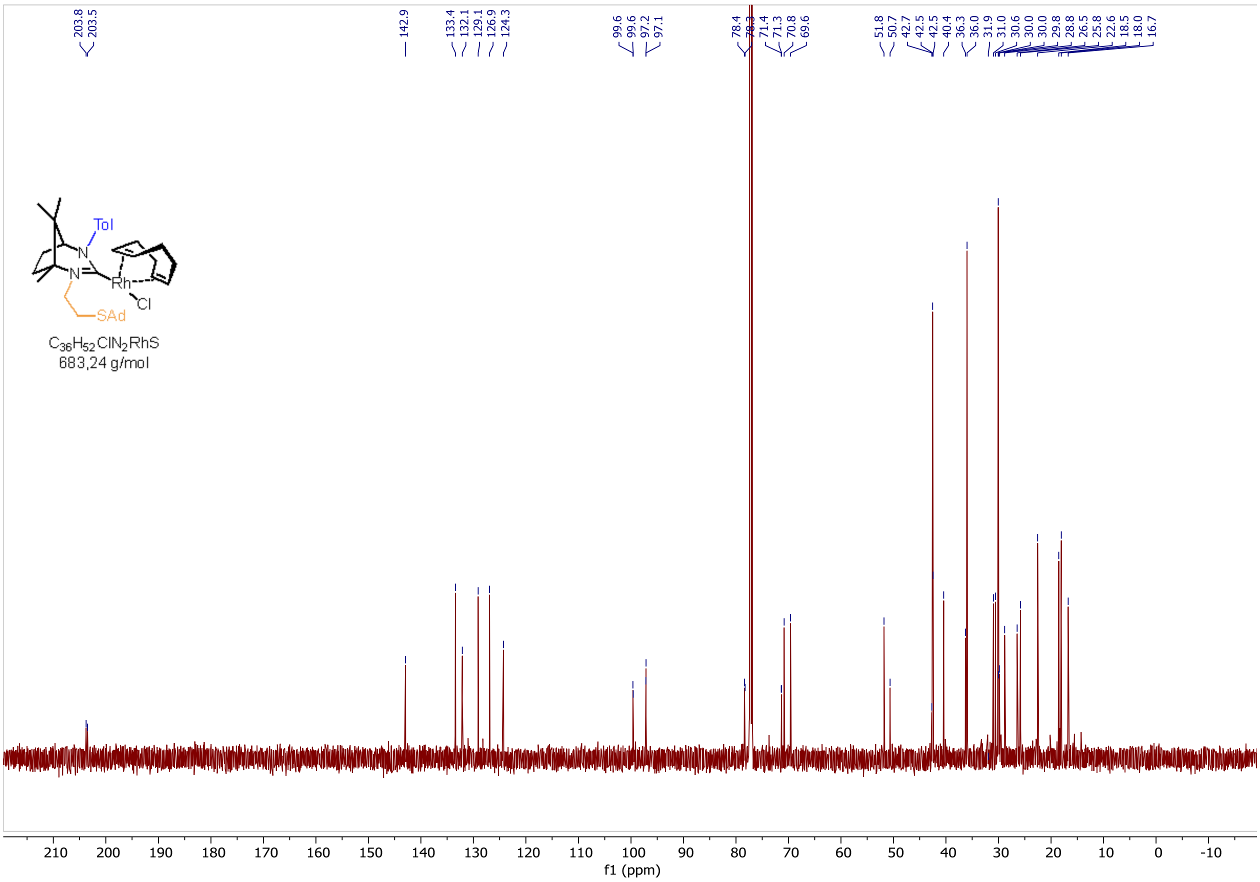

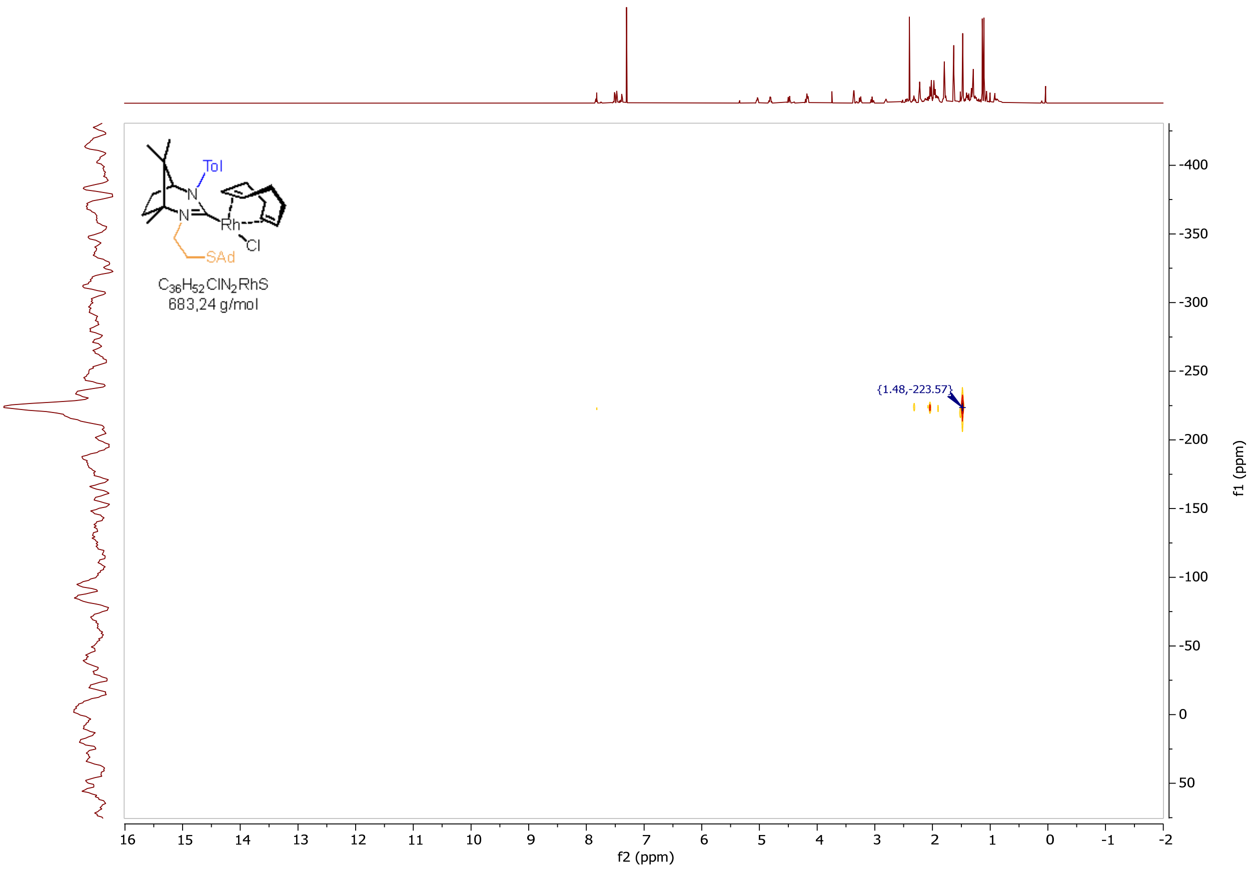
Tol
N
N
Rh
Cl
SAd
$C_{36}H_{52}ClN_2RhS$
683,24 g/mol
{1.48,-223.57}
f1 (ppm)
f2 (ppm)

^1H NMR (600 MHz, $CDCl_3$), ^{13}C NMR (151 MHz, $CDCl_3$) and ^{15}N HSQC NMR (61 MHz, $CDCl_3$)Analysis of Compound **Rh5b**

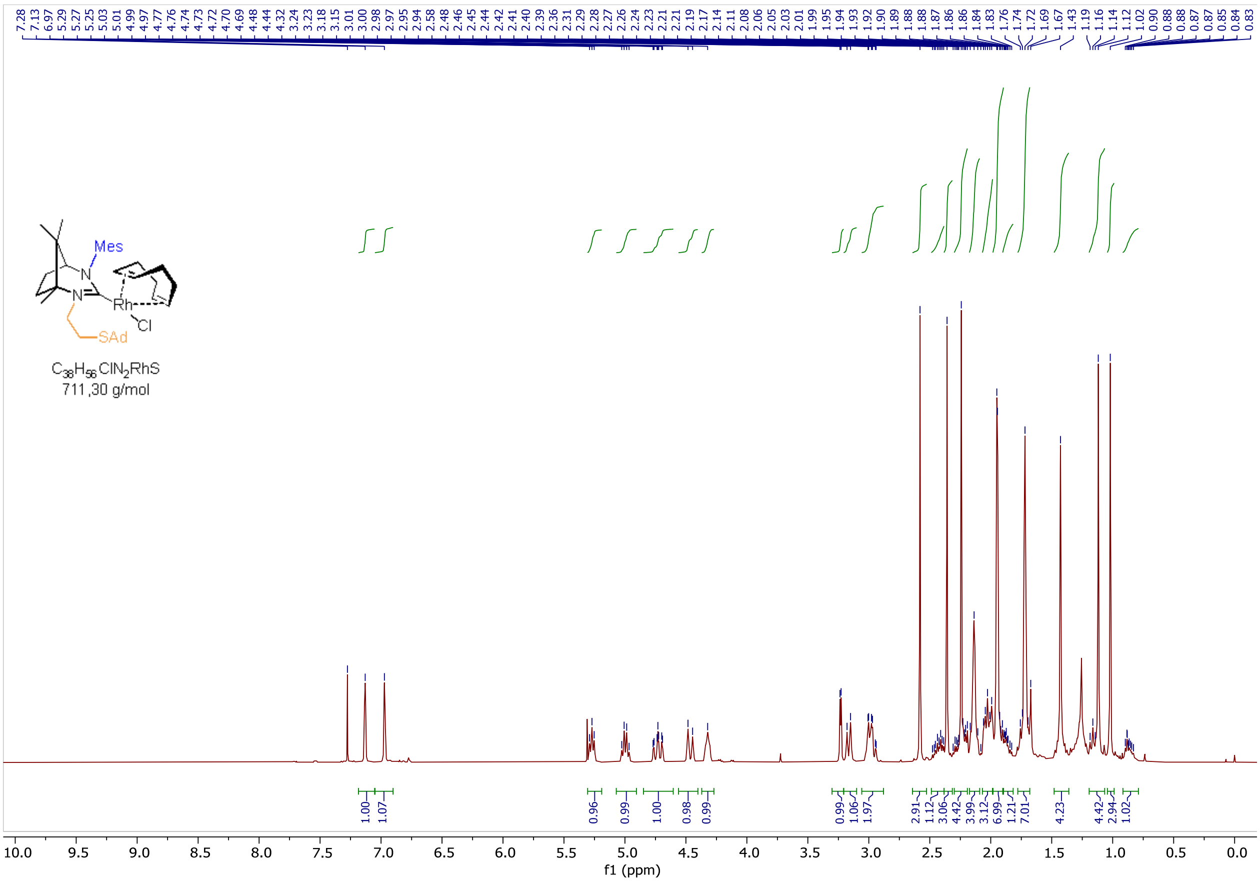

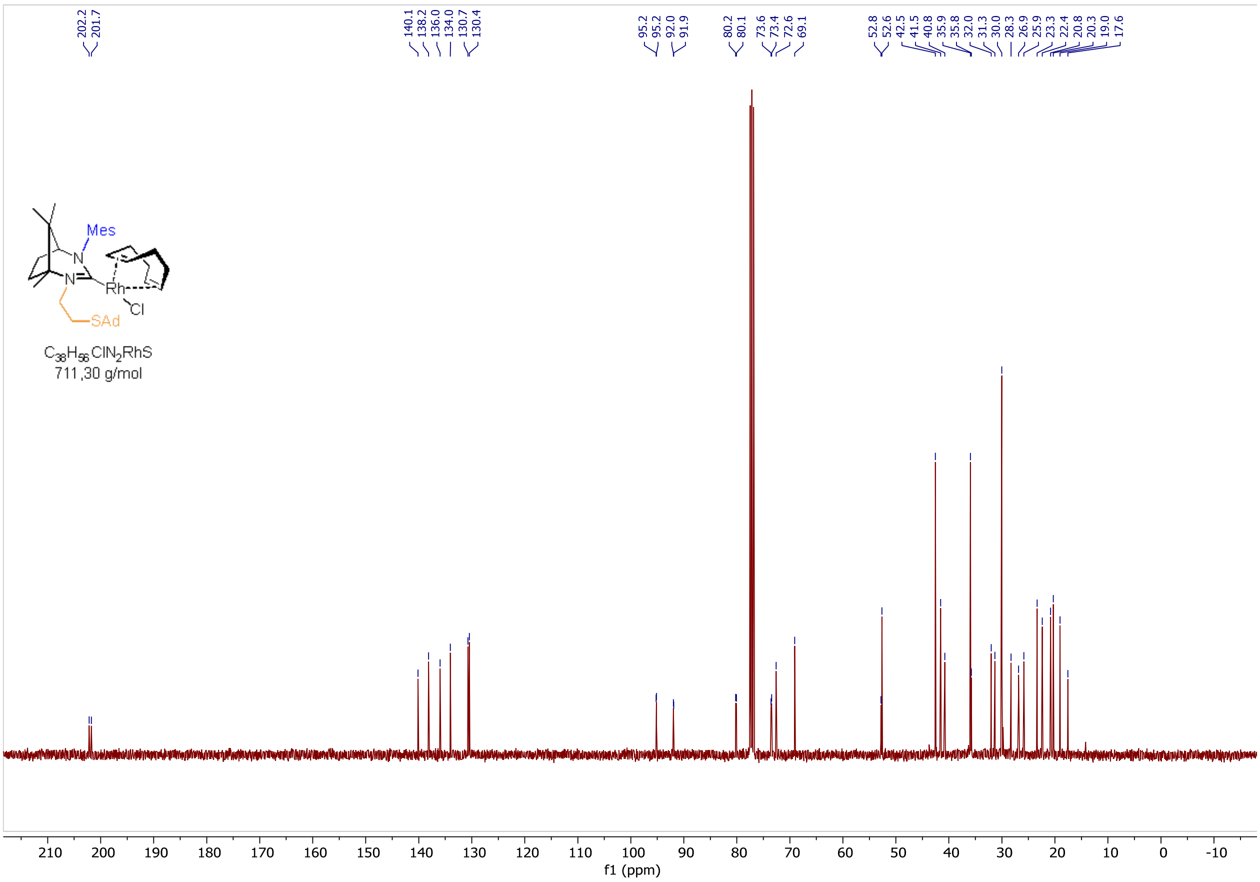

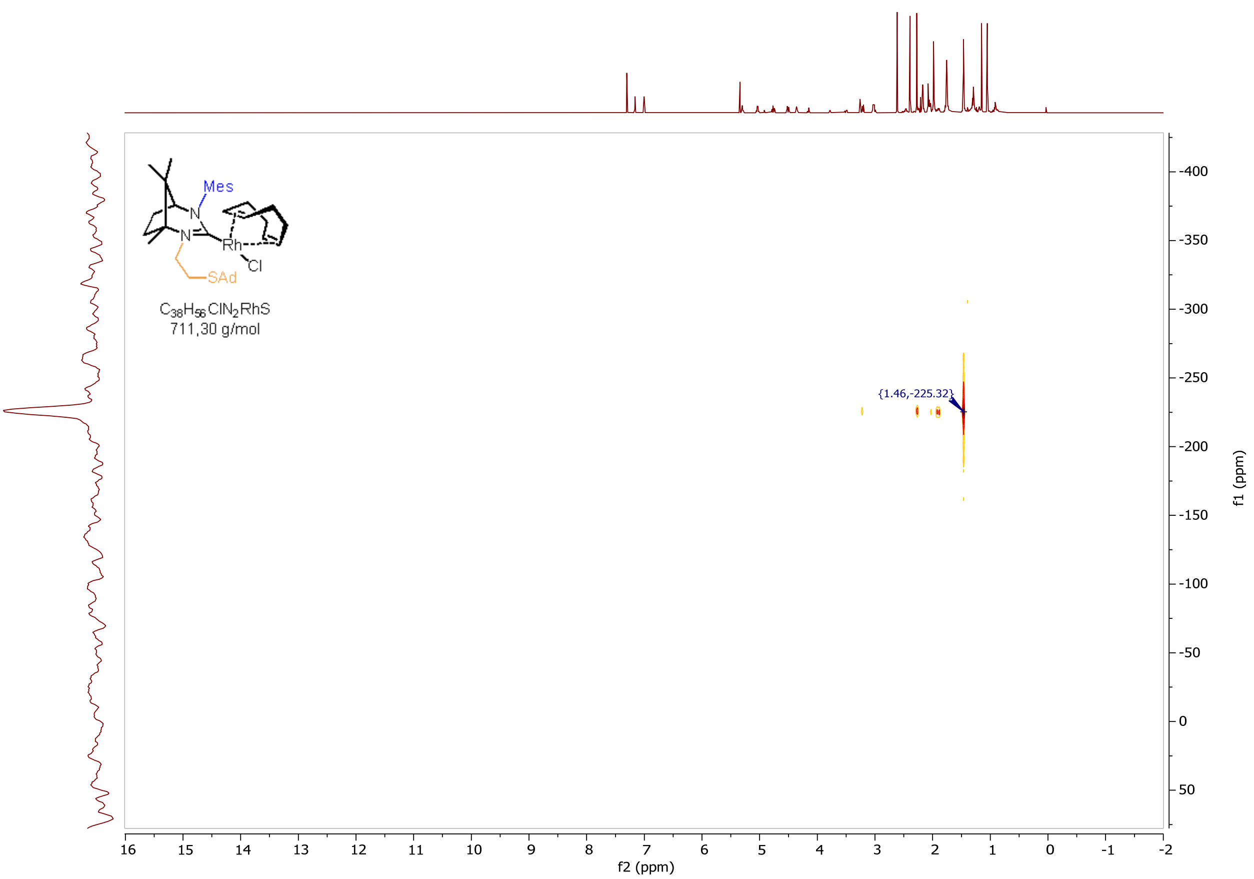
Mes
N
N
Rh
Cl
SAd
$C_{38}H_{56}ClN_2RhS$
711,30 g/mol
{1.46,-225.32}
f2 (ppm)
f1 (ppm)

^{1}H NMR (600 MHz, $CDCl_3$), ^{13}C NMR (151 MHz, $CDCl_3$) and ^{15}N HSQC NMR (61 MHz, $CDCl_3$) Analysis of Compound **Rh5c**

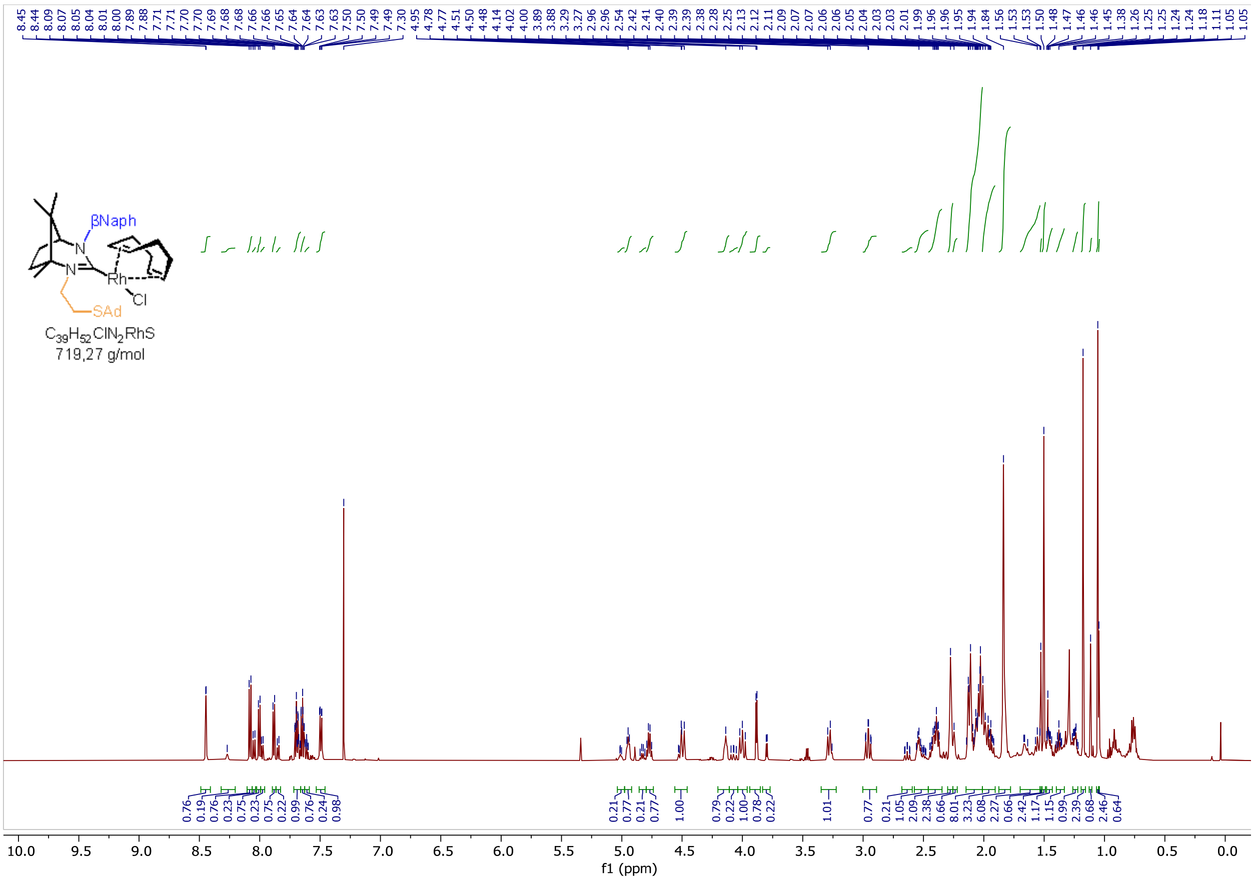

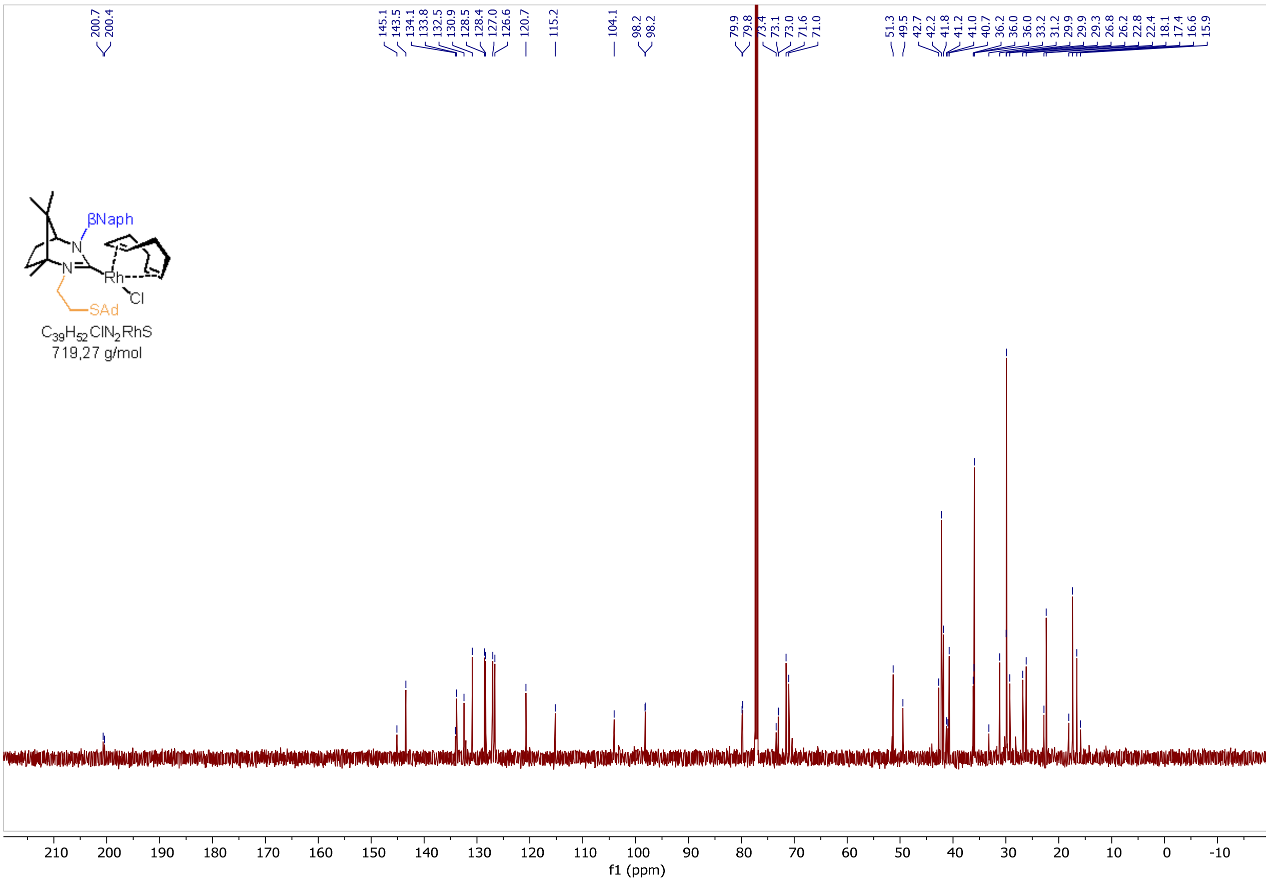

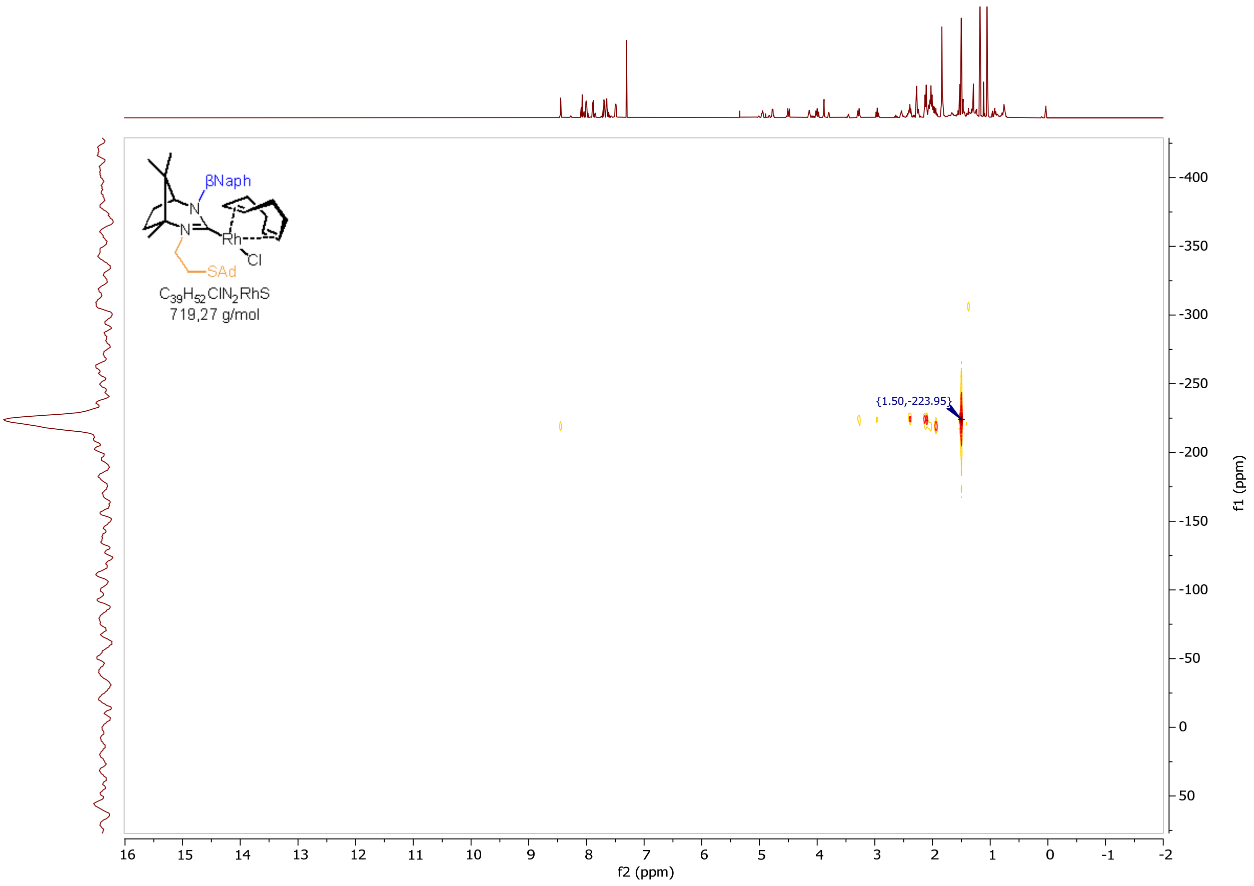
βNaph
N
N
Rh
Cl
SAd
$C_{39}H_{52}ClN_2RhS$
719,27 g/mol
{1.50,-223.95}
f1 (ppm)
f2 (ppm)

^{1}H NMR (600 MHz, $CDCl_3$), ^{13}C NMR (151 MHz, $CDCl_3$) and ^{15}N HSQC NMR (61 MHz, $CDCl_3$) Analysis of Compound **Rh5d**

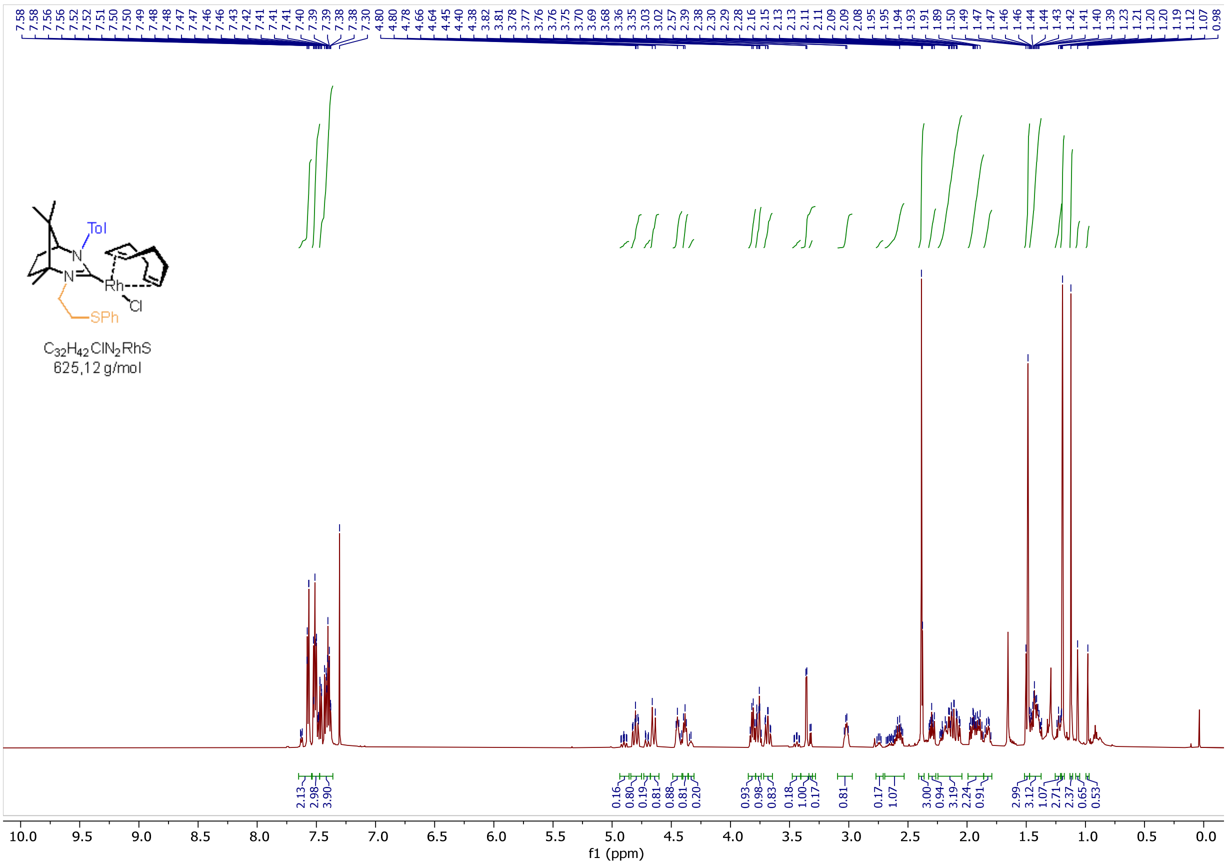

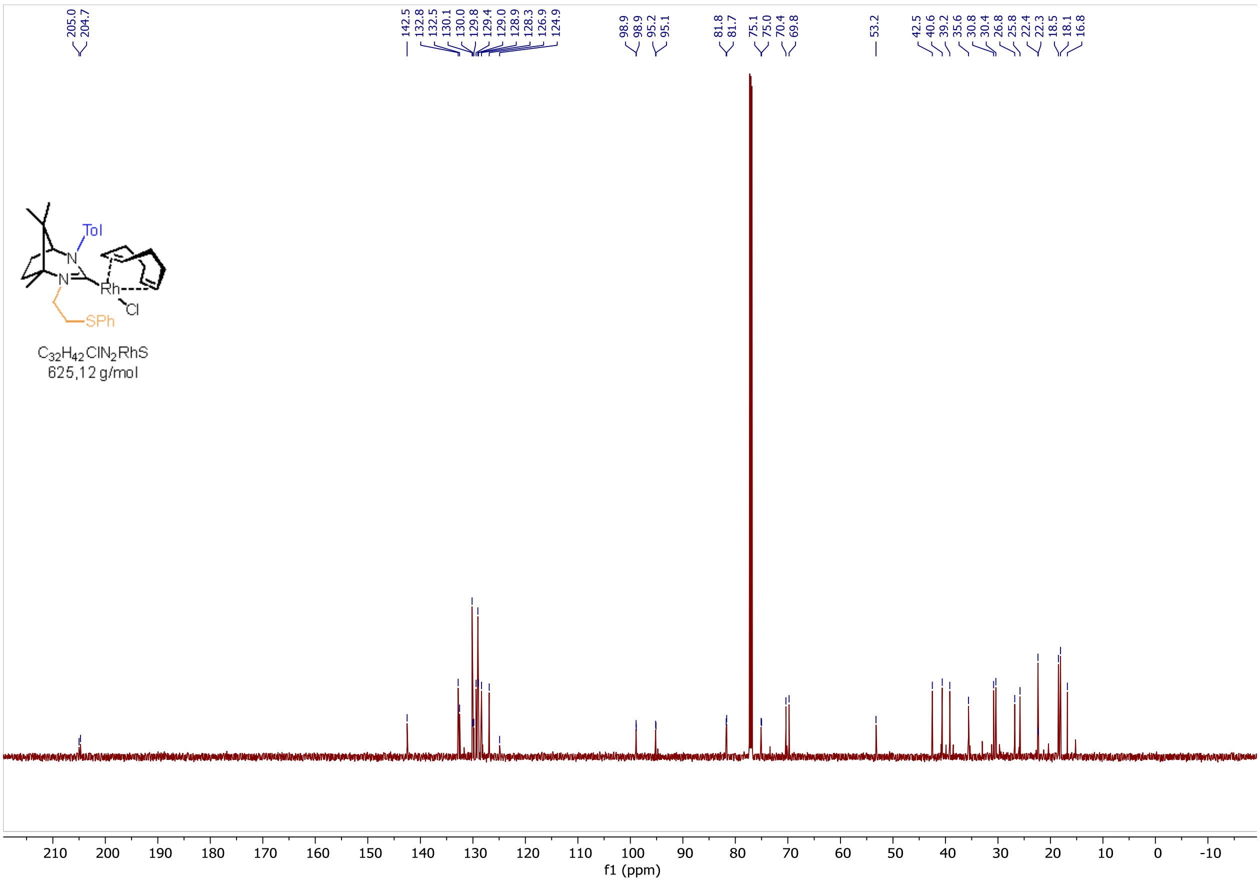

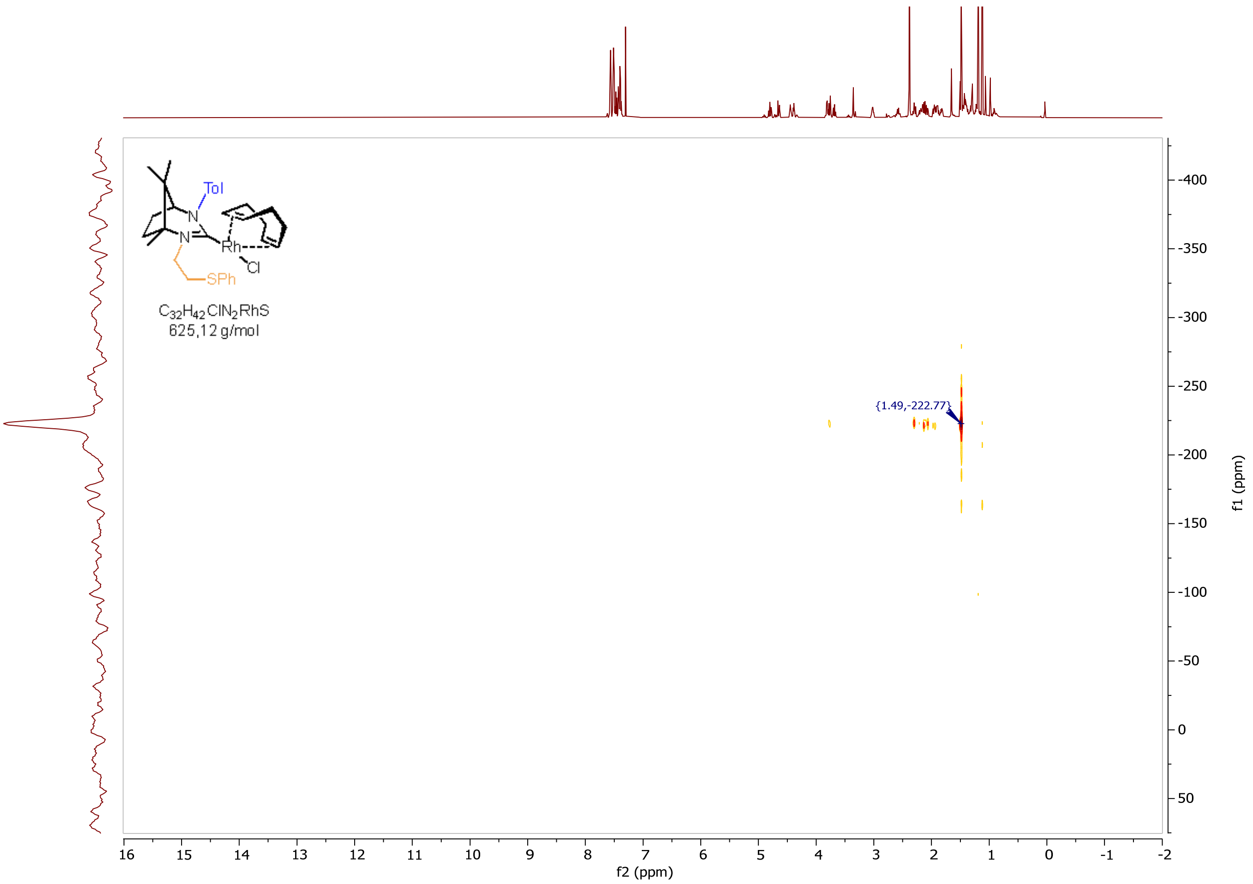

Tol
N
N
Rh
Cl
SPh
C32H42ClN2RhS
625,12 g/mol
{1.49,-222.77}
f1 (ppm)
f2 (ppm)

^{1}H NMR (600 MHz, $CDCl_3$), ^{13}C NMR (151 MHz, $CDCl_3$) and ^{15}N HSQC NMR (61 MHz, $CDCl_3$) Analysis of Compound **Rh5e**

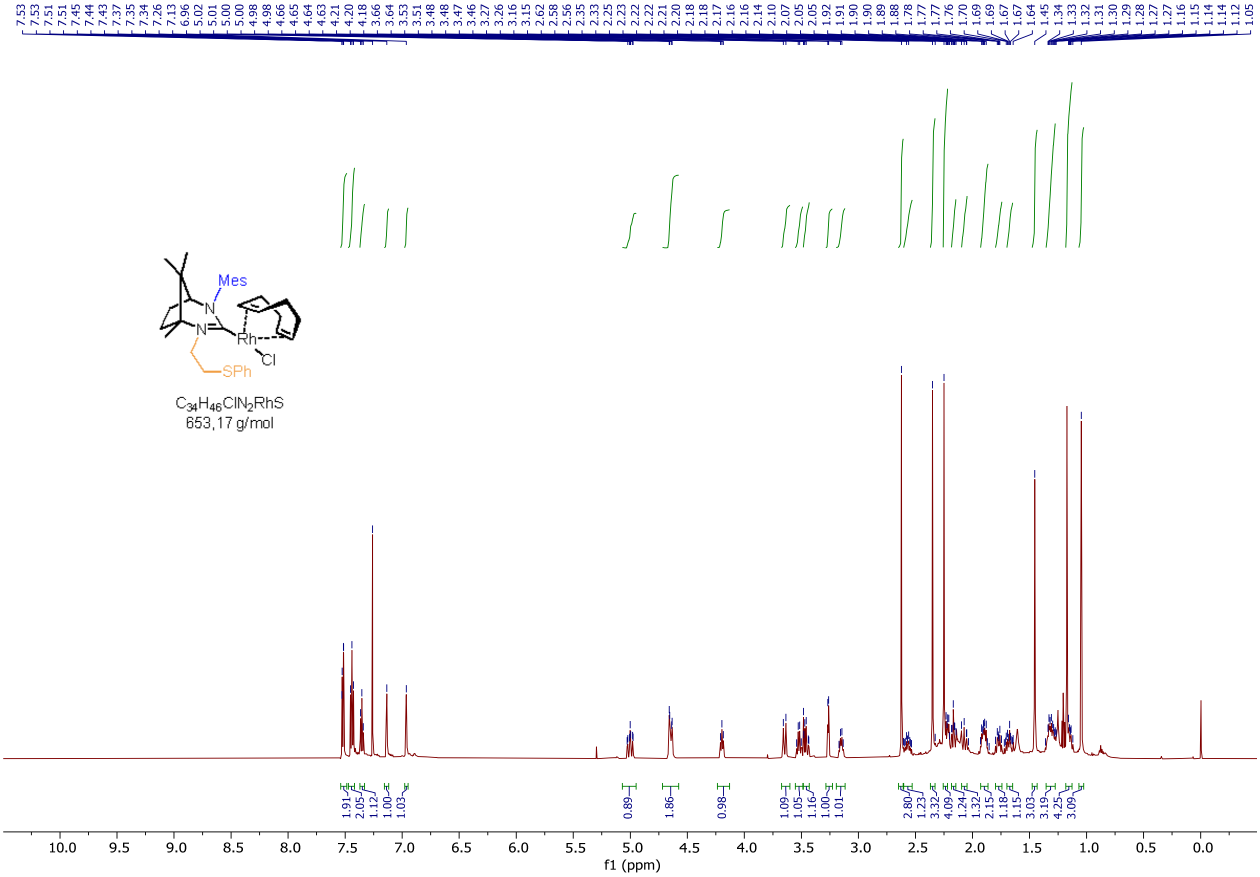

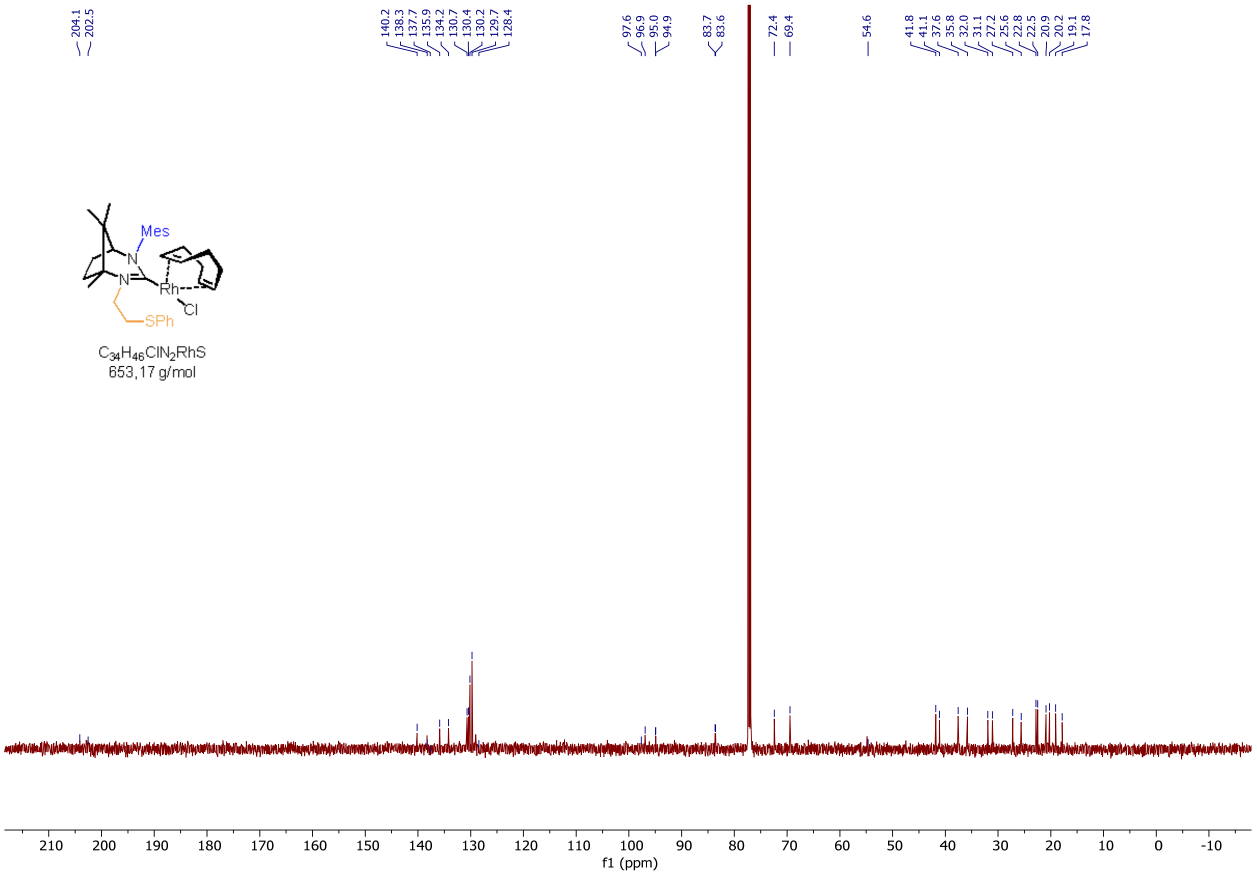

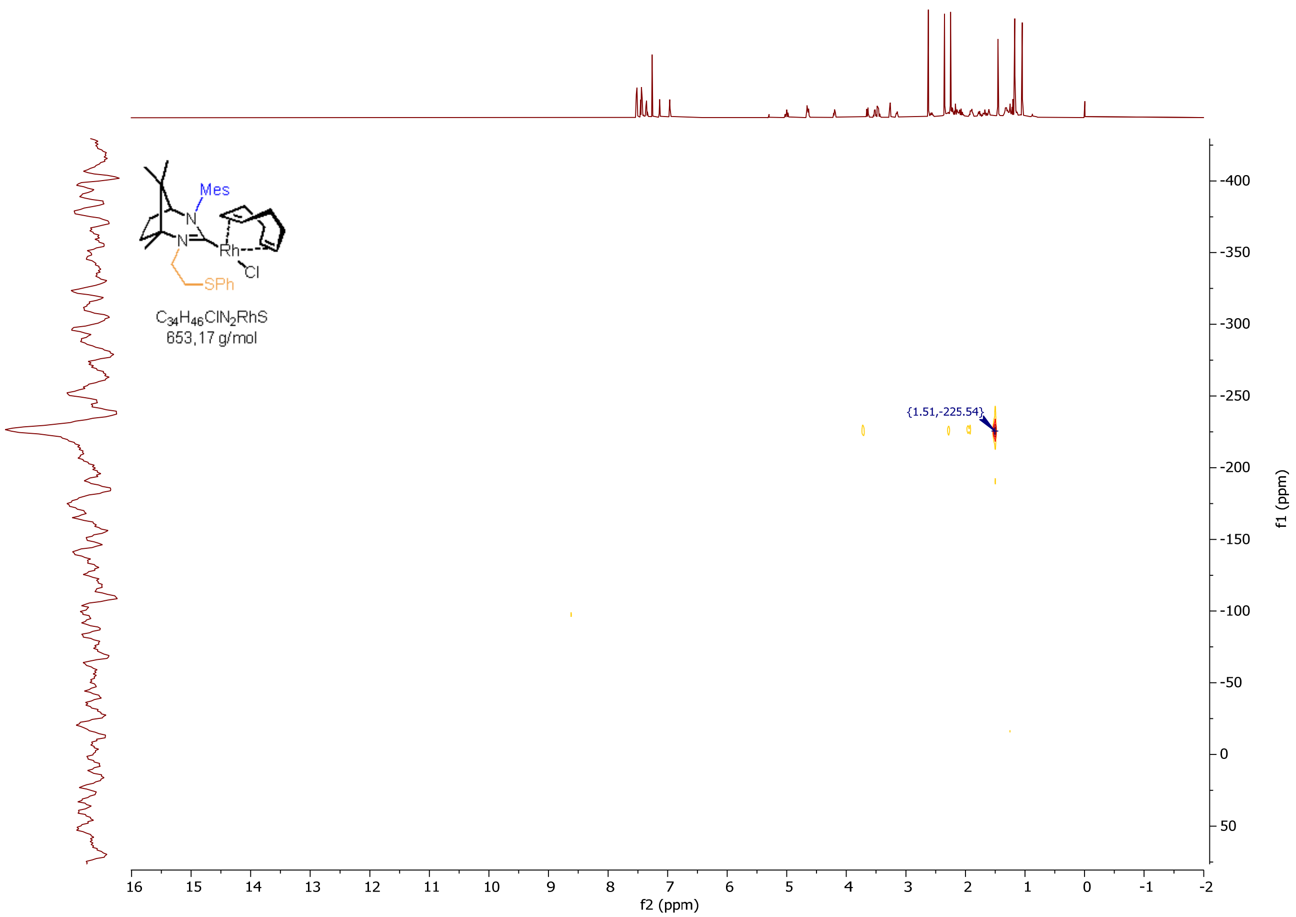

Mes
N
N
Rh
Cl
SPh
C34H46ClN2RhS
653,17 g/mol
{1.51,-225.54}
f2 (ppm)
f1 (ppm)

^{1}H NMR (600 MHz, $CDCl_3$), ^{13}C NMR (151 MHz, $CDCl_3$) and ^{15}N HSQC NMR (61 MHz, $CDCl_3$) Analysis of Compound **Rh5f**

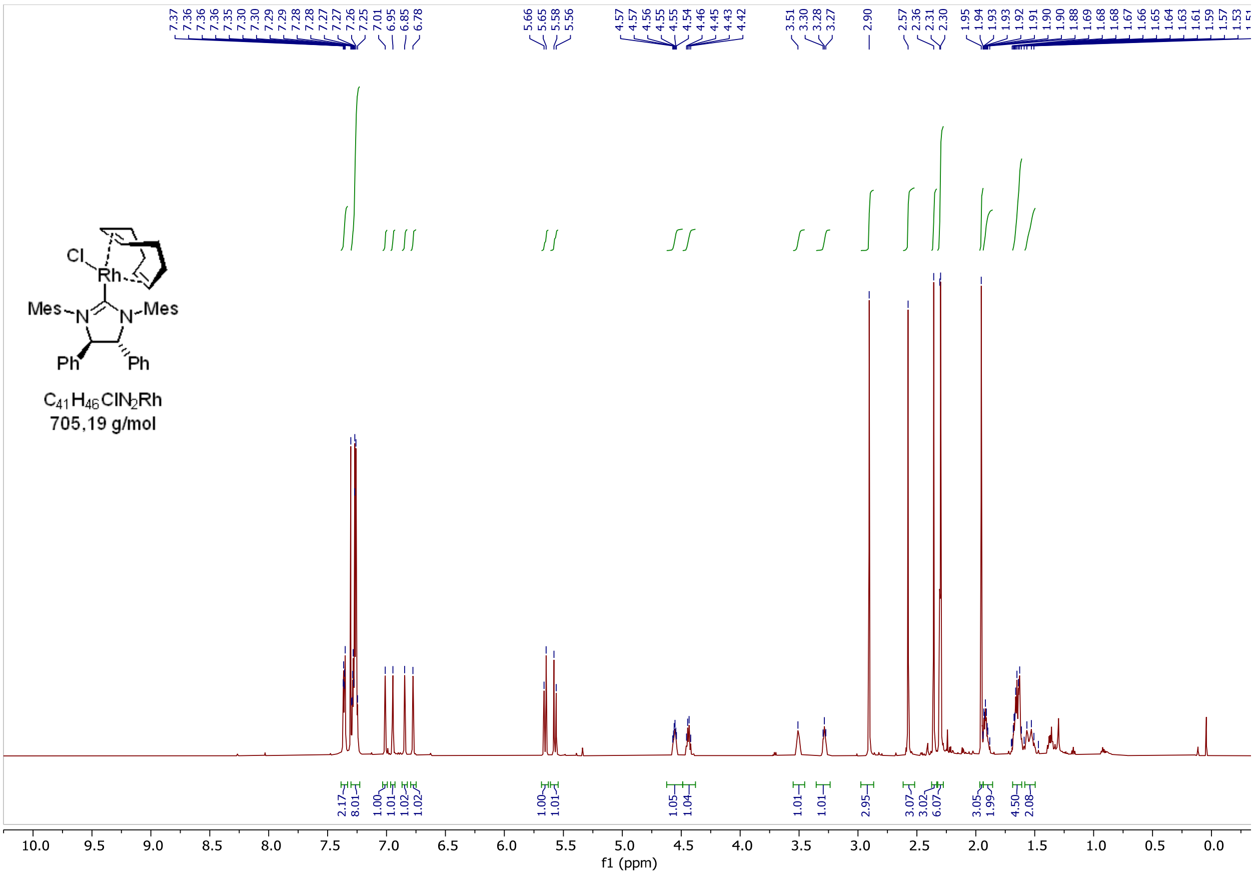

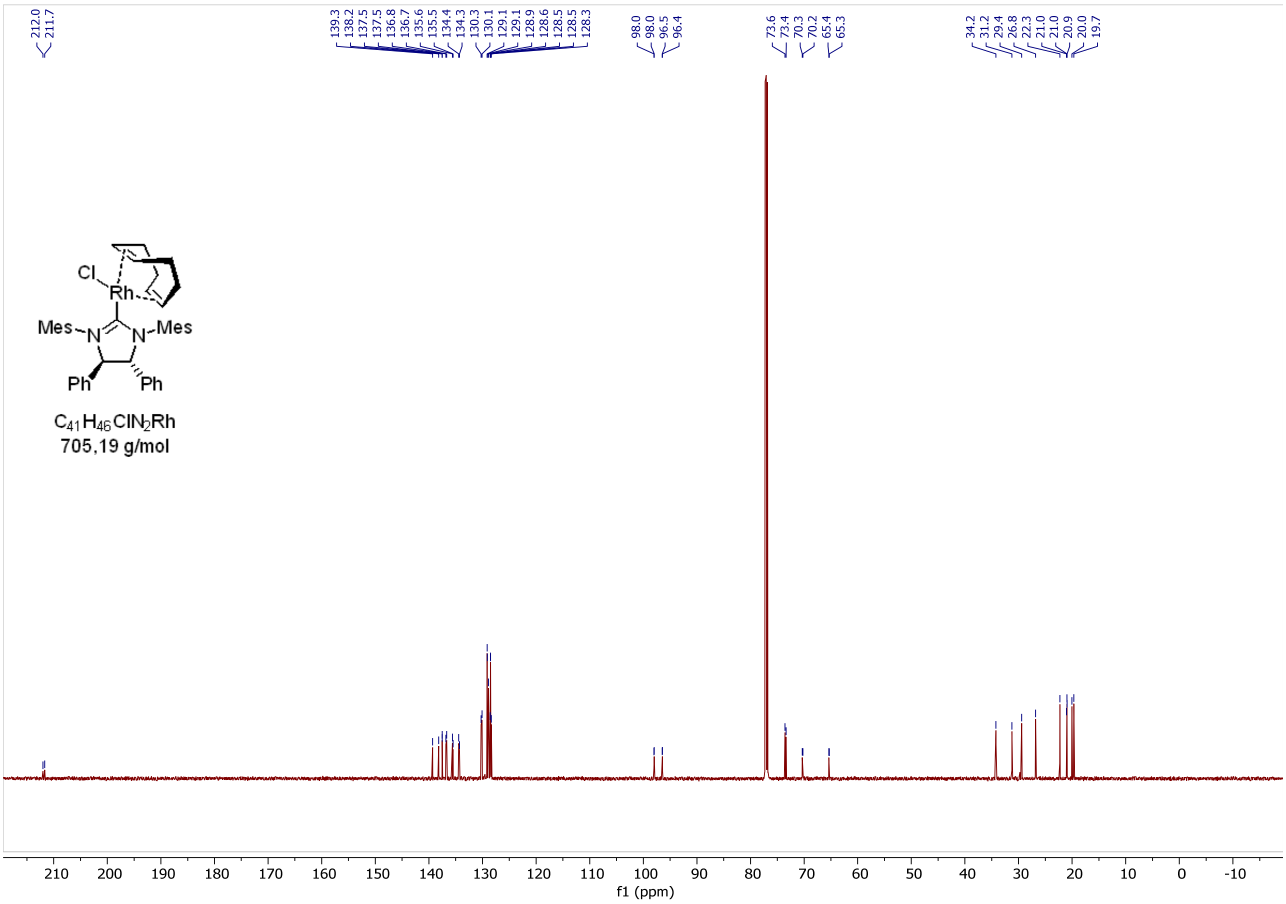

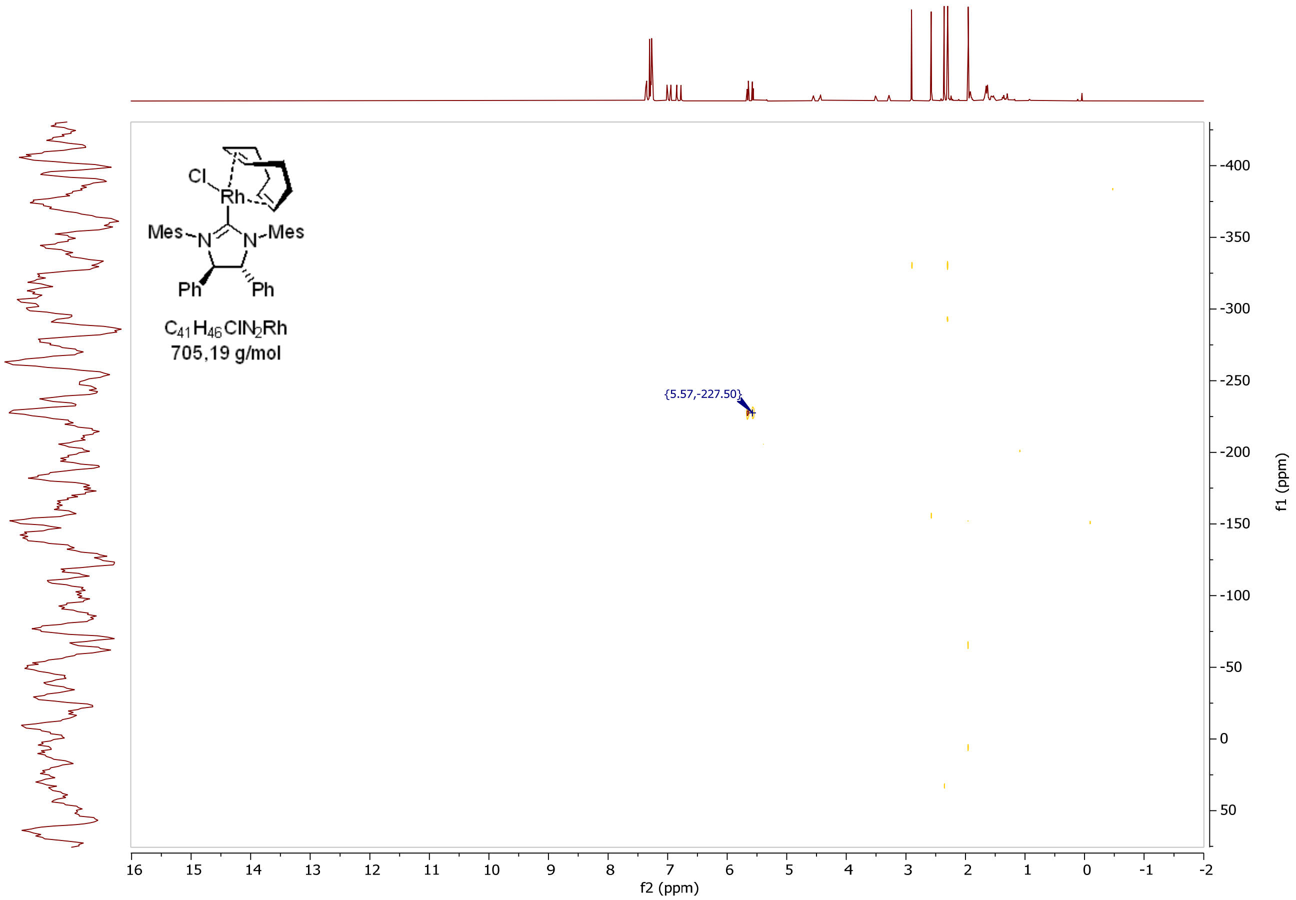
Cl
Rh
Mes
N
N
Mes
Ph
Ph
$C_{41}H_{46}ClN_2Rh$
705,19 g/mol
{5.57,-227.50}
f2 (ppm)
f1 (ppm)

^{1}H NMR (600 MHz, $CDCl_3$), ^{13}C NMR (151 MHz, $CDCl_3$) and ^{15}N HSQC NMR (61 MHz, $CDCl_3$) Analysis of Compound **Rh5g**

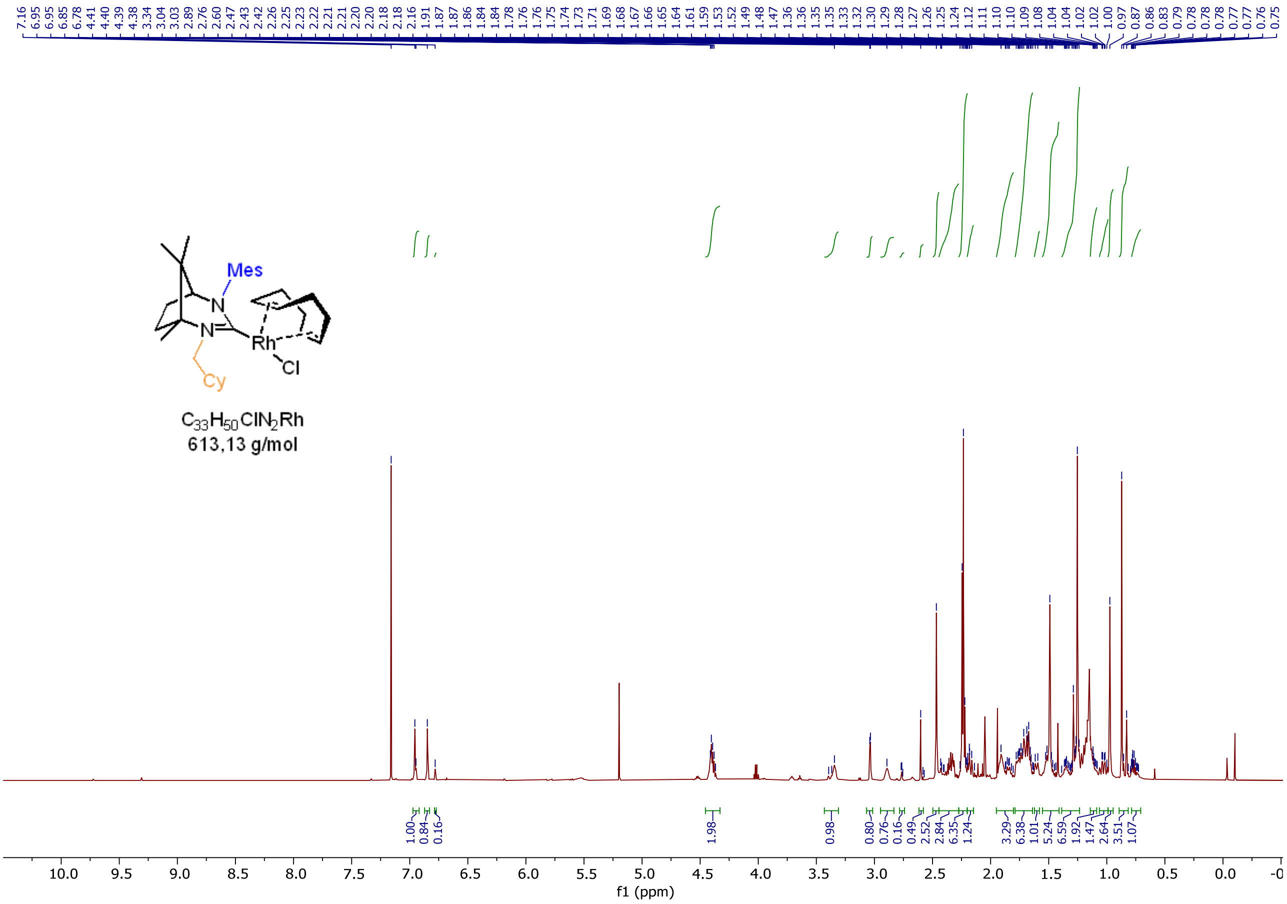

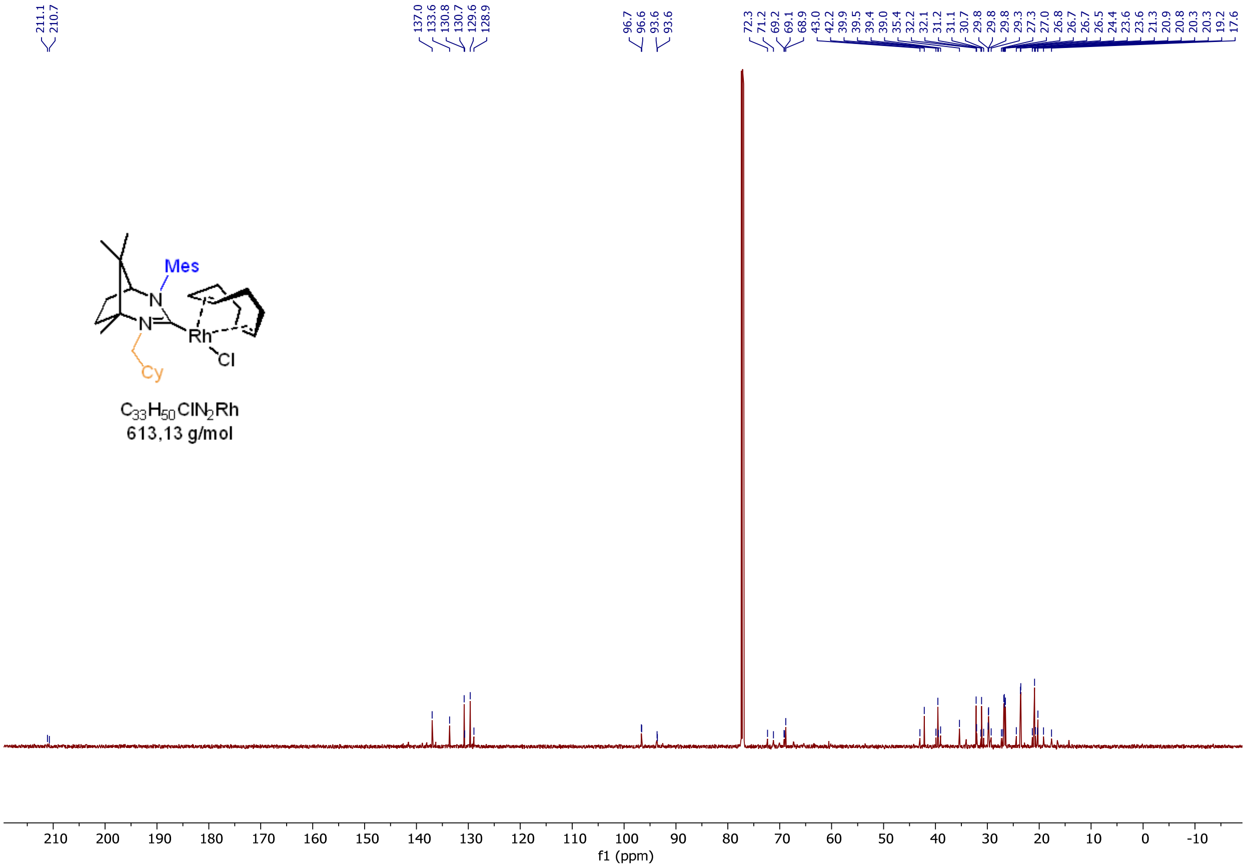

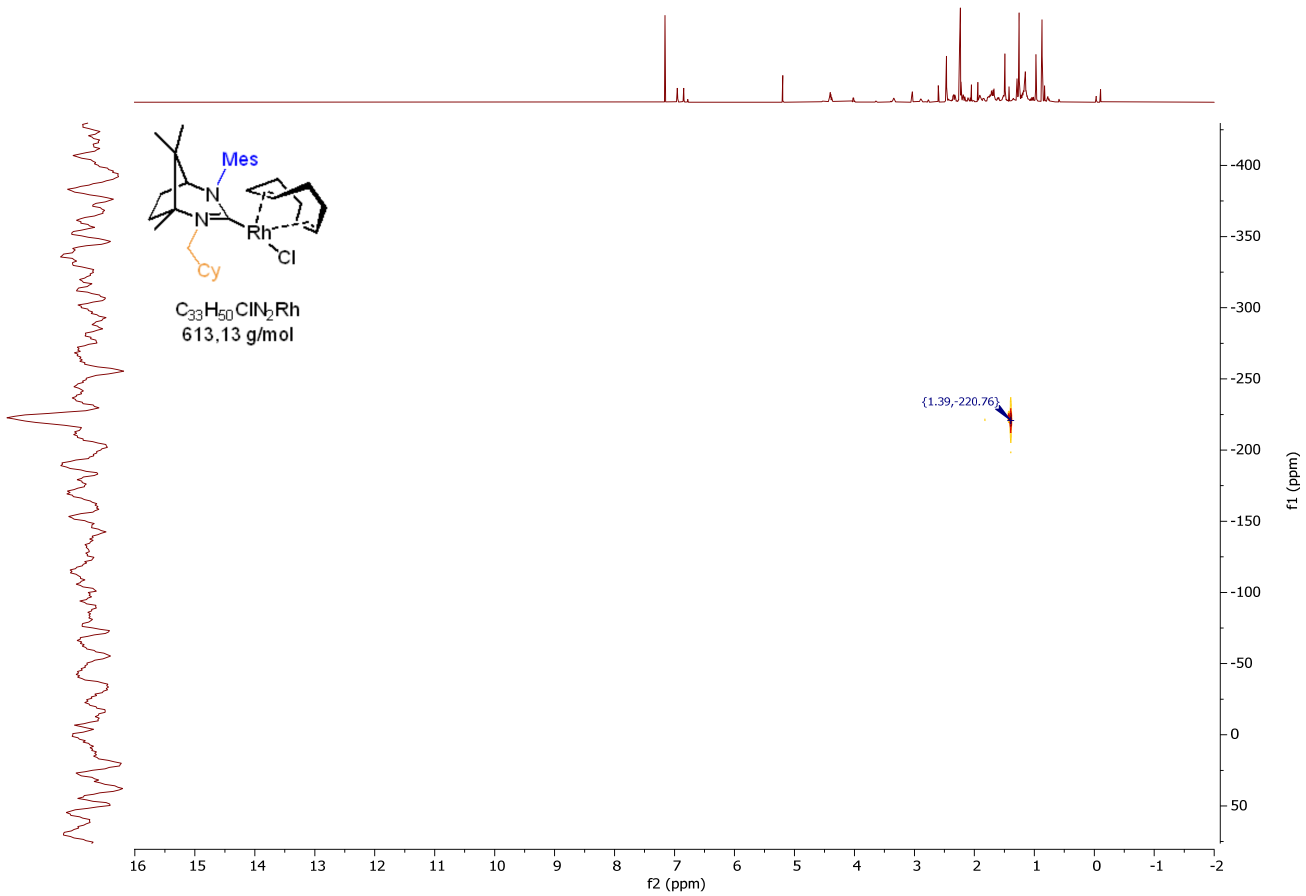
Mes
N
N
Rh
Cl
Cy
$C_{33}H_{50}ClN_2Rh$
613,13 g/mol
{1.39,-220.76}
f1 (ppm)
f2 (ppm)

^{1}H NMR (600 MHz, $CDCl_3$), ^{13}C NMR (151 MHz, $CDCl_3$) and ^{15}N HSQC NMR (61 MHz, $CDCl_3$) Analysis of Compound **Rh(SIMes)**

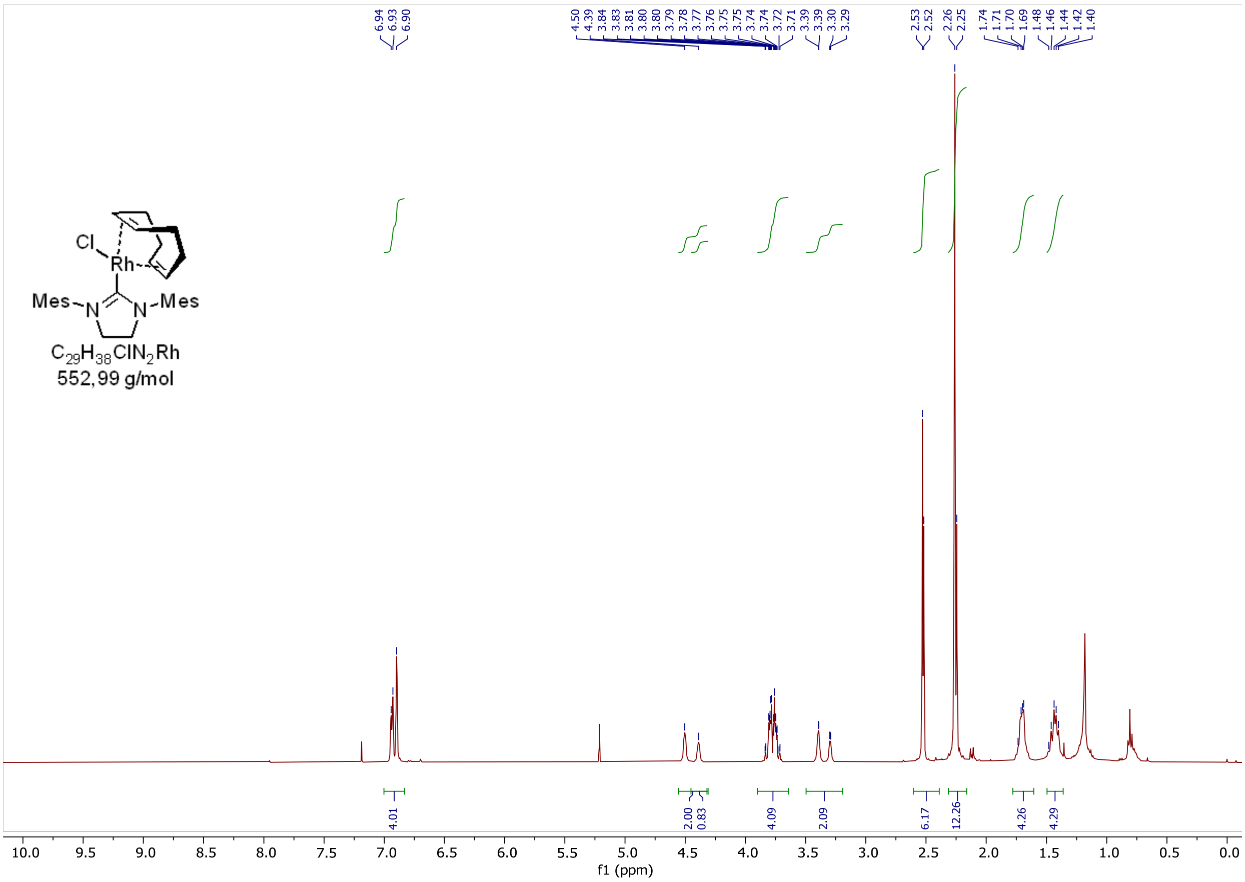

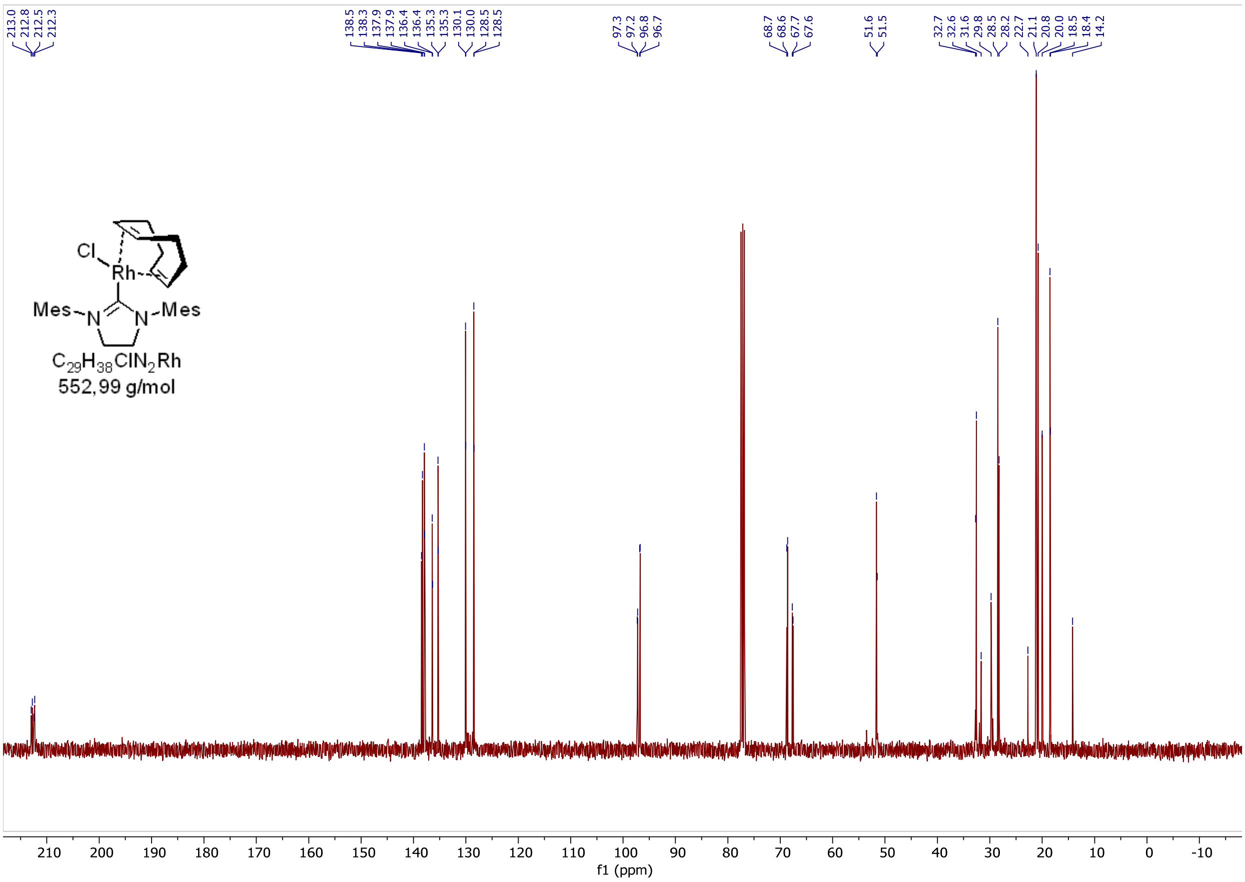

^1H NMR (400 MHz, $CDCl_3$) and ^{13}C NMR (101 MHz, $CDCl_3$) Analysis of Compound **6*N*-Boc**

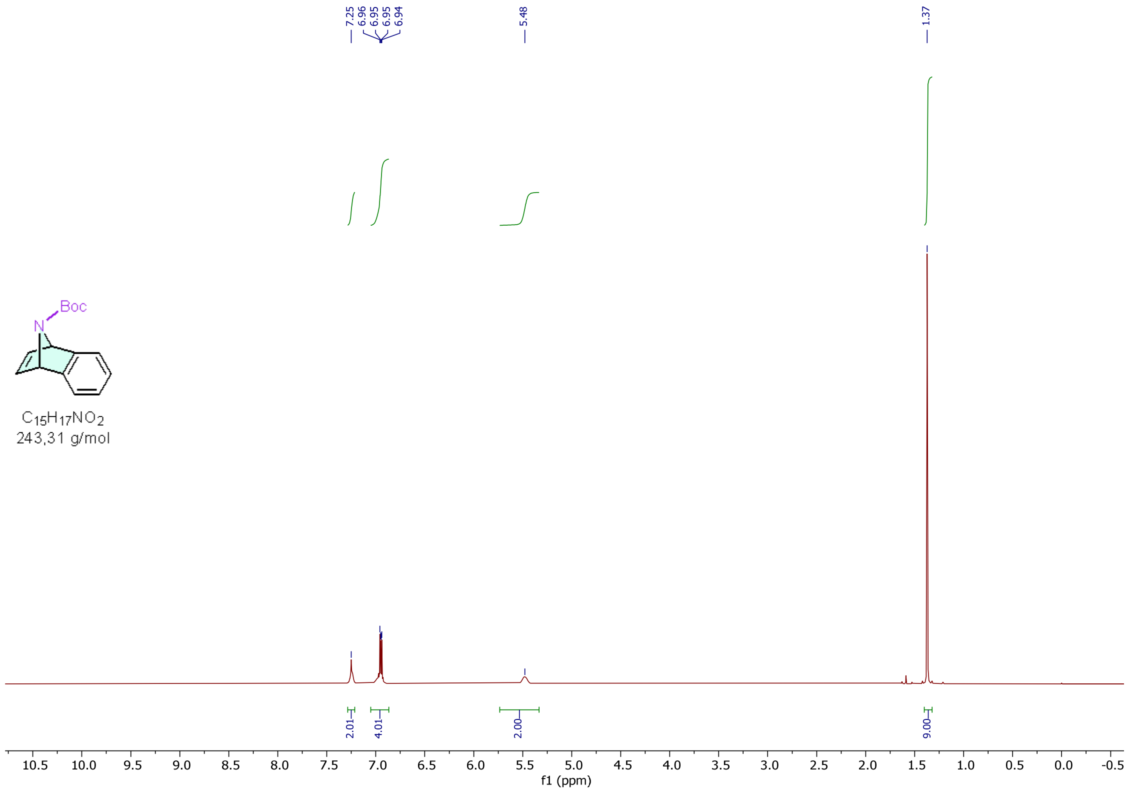

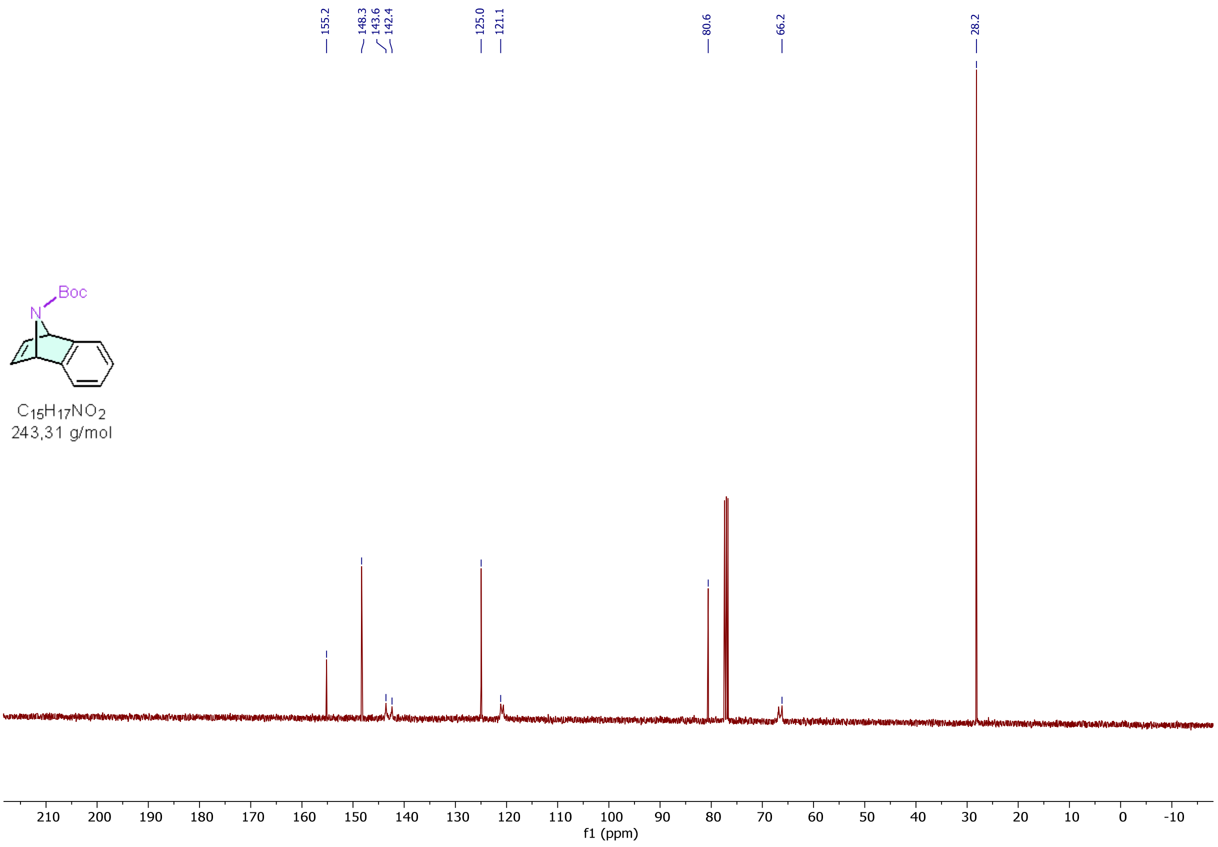

^{1}H NMR (400 MHz, $CDCl_3$) and ^{13}C NMR (101 MHz, $CDCl_3$) Analysis of Compound **6*N*-Ts**

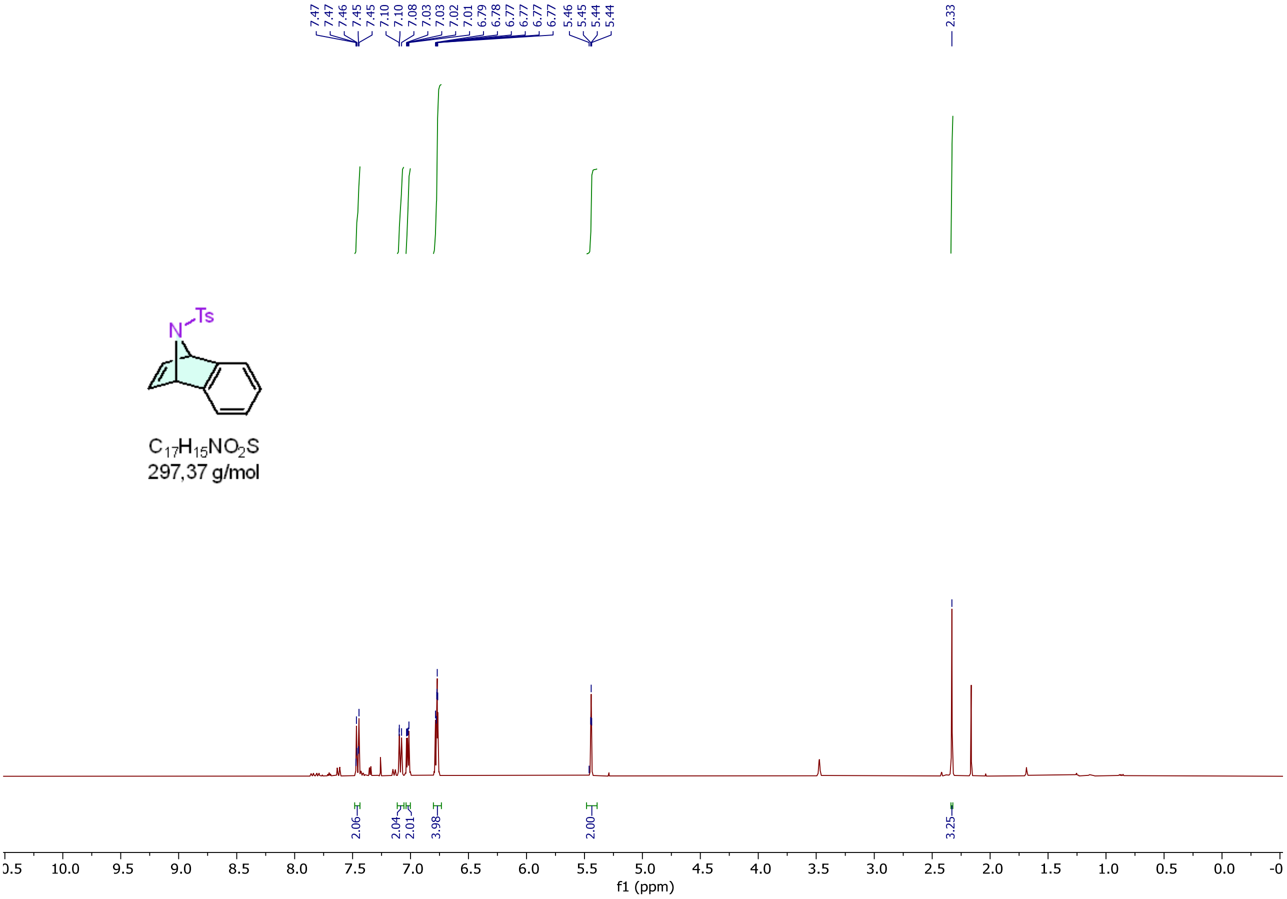

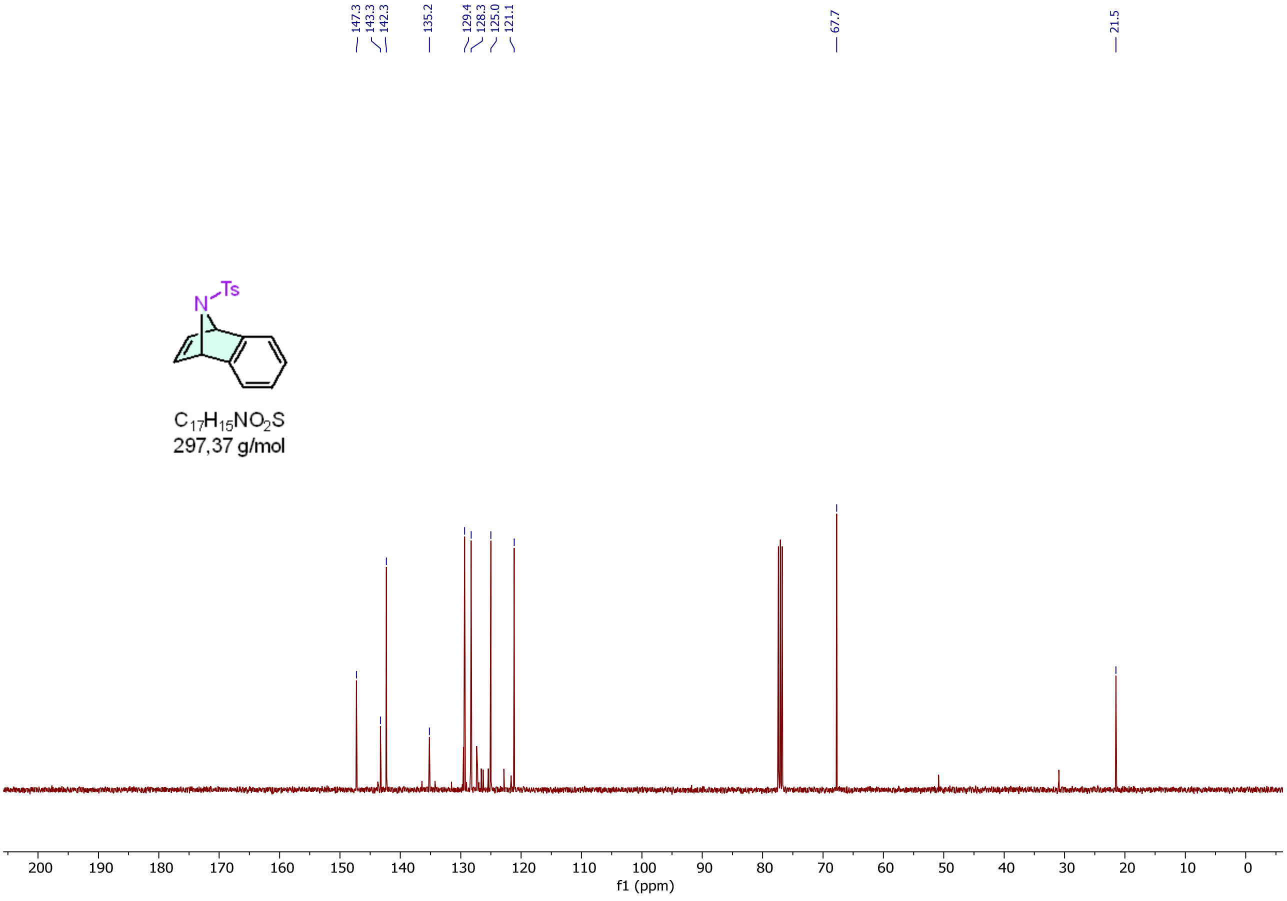

^{1}H NMR (400 MHz, $CDCl_3$) and ^{13}C NMR (101 MHz, $CDCl_3$) Analysis of Compound **6*N*-Ns**

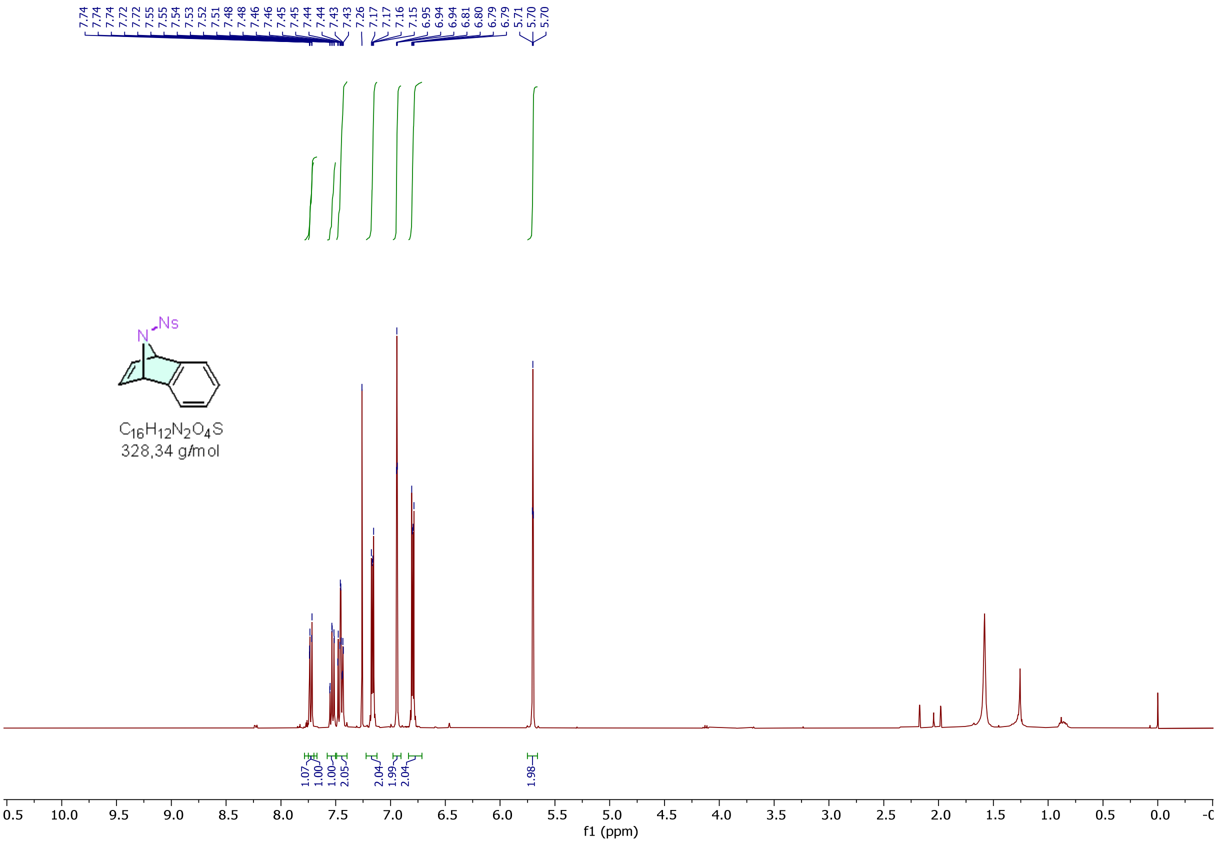

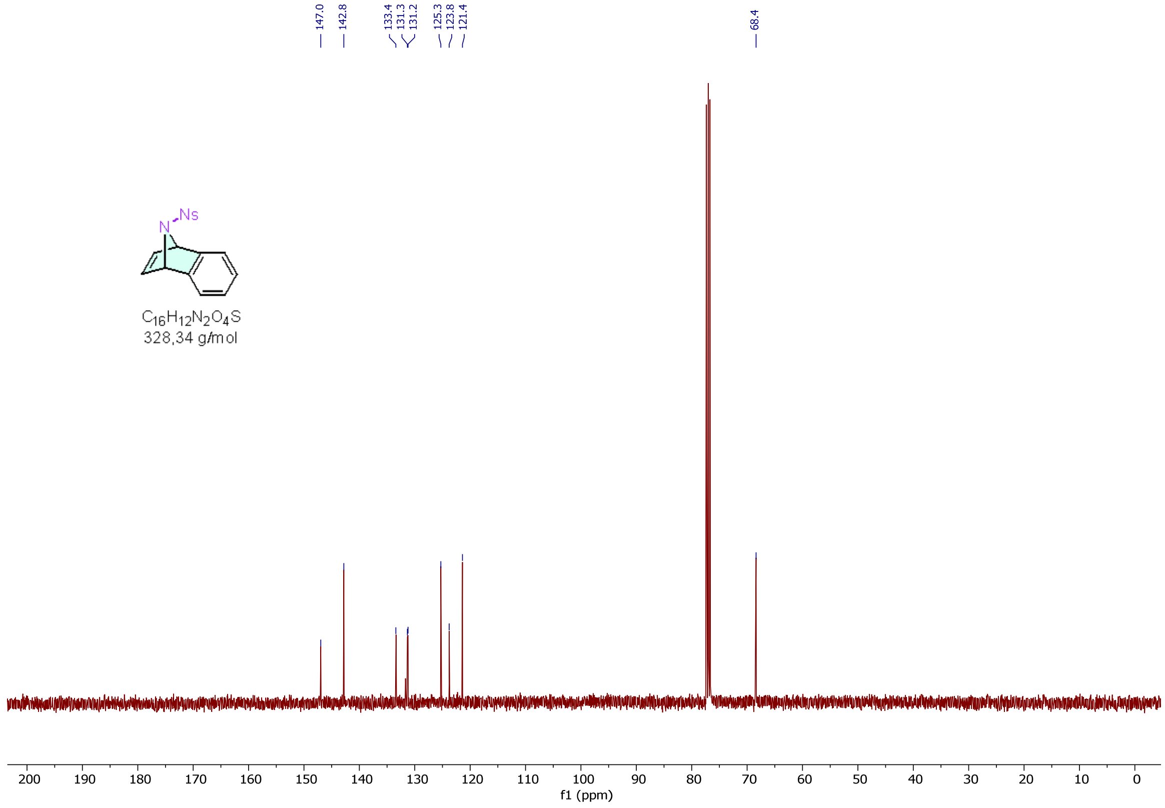

^{1}H NMR (400 MHz, $CDCl_3$) and ^{13}C NMR (101 MHz, $CDCl_3$) Analysis of Compound **6*N*-*p*Ns**

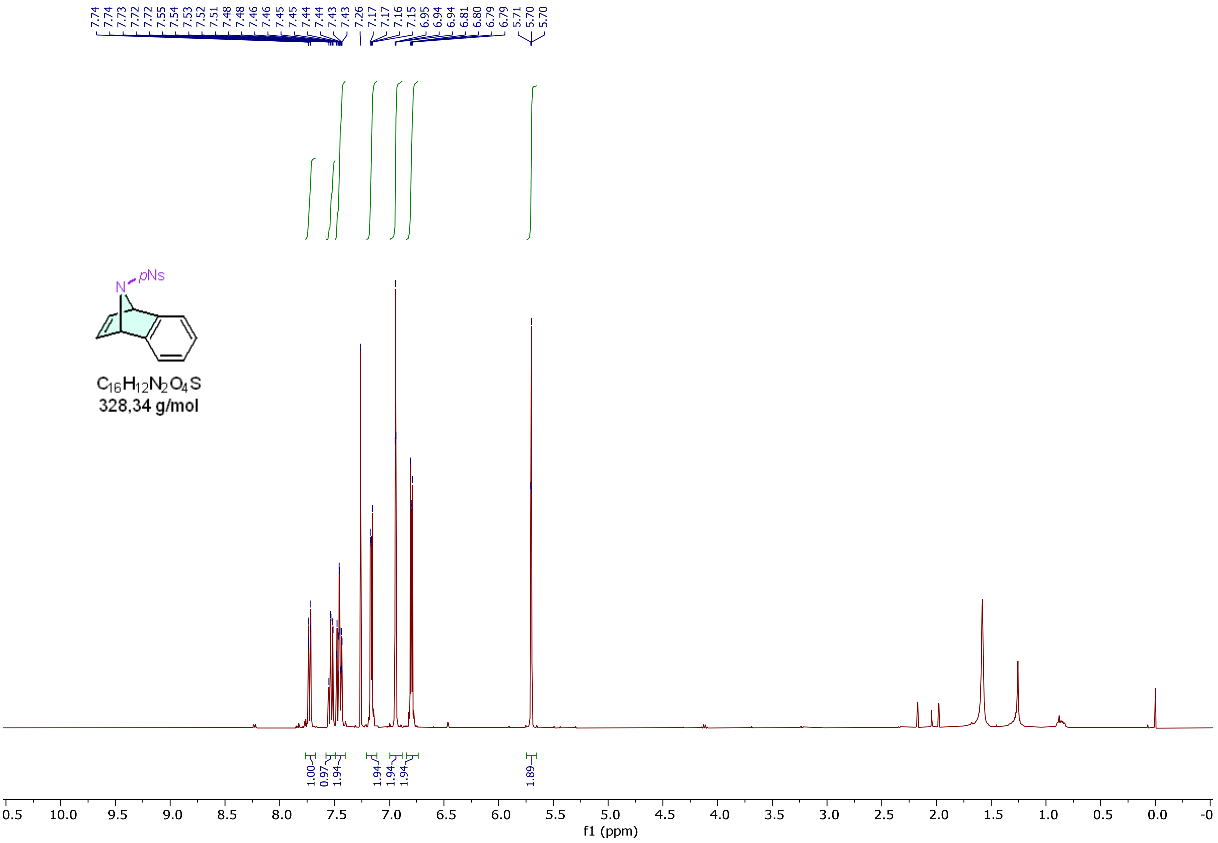

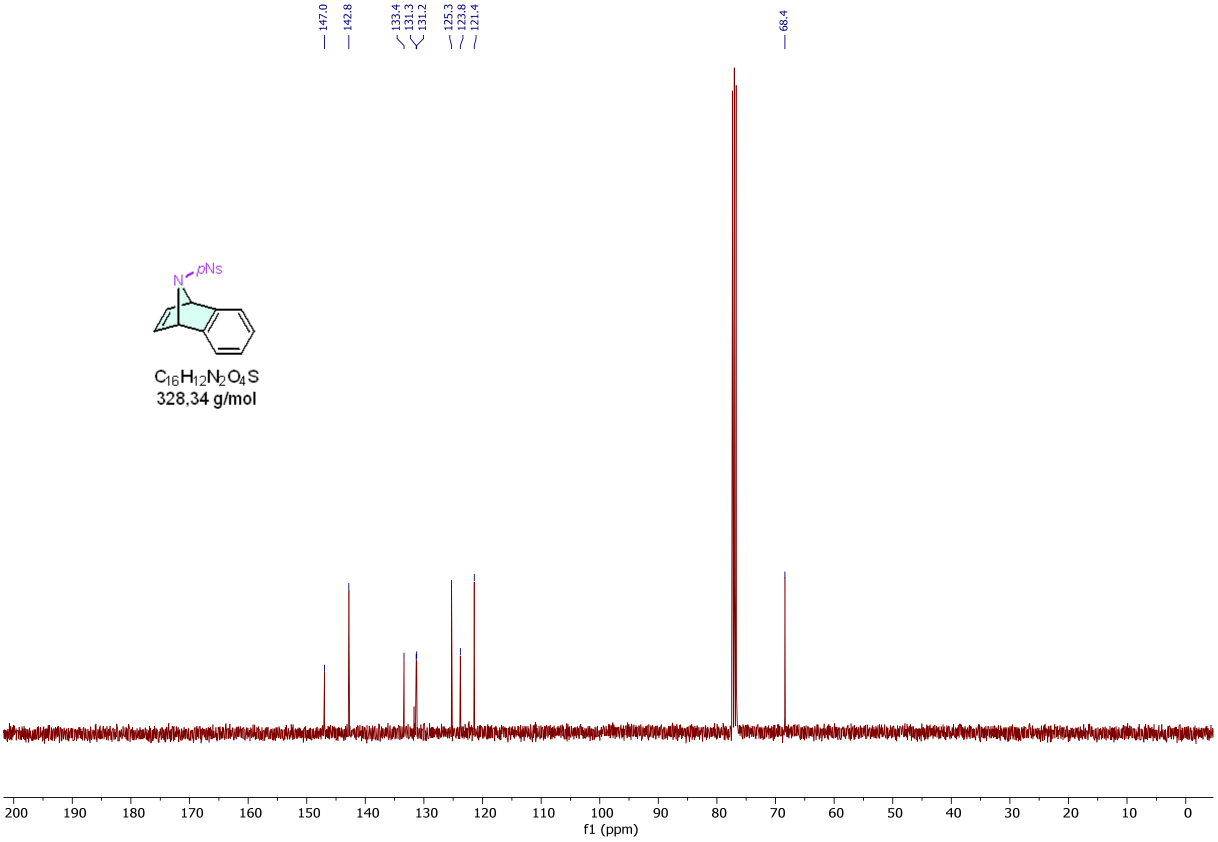

^{1}H NMR (400 MHz, $CDCl_3$) and ^{13}C NMR (101 MHz, $CDCl_3$) Analysis of Compound **6*N*-BsBr**

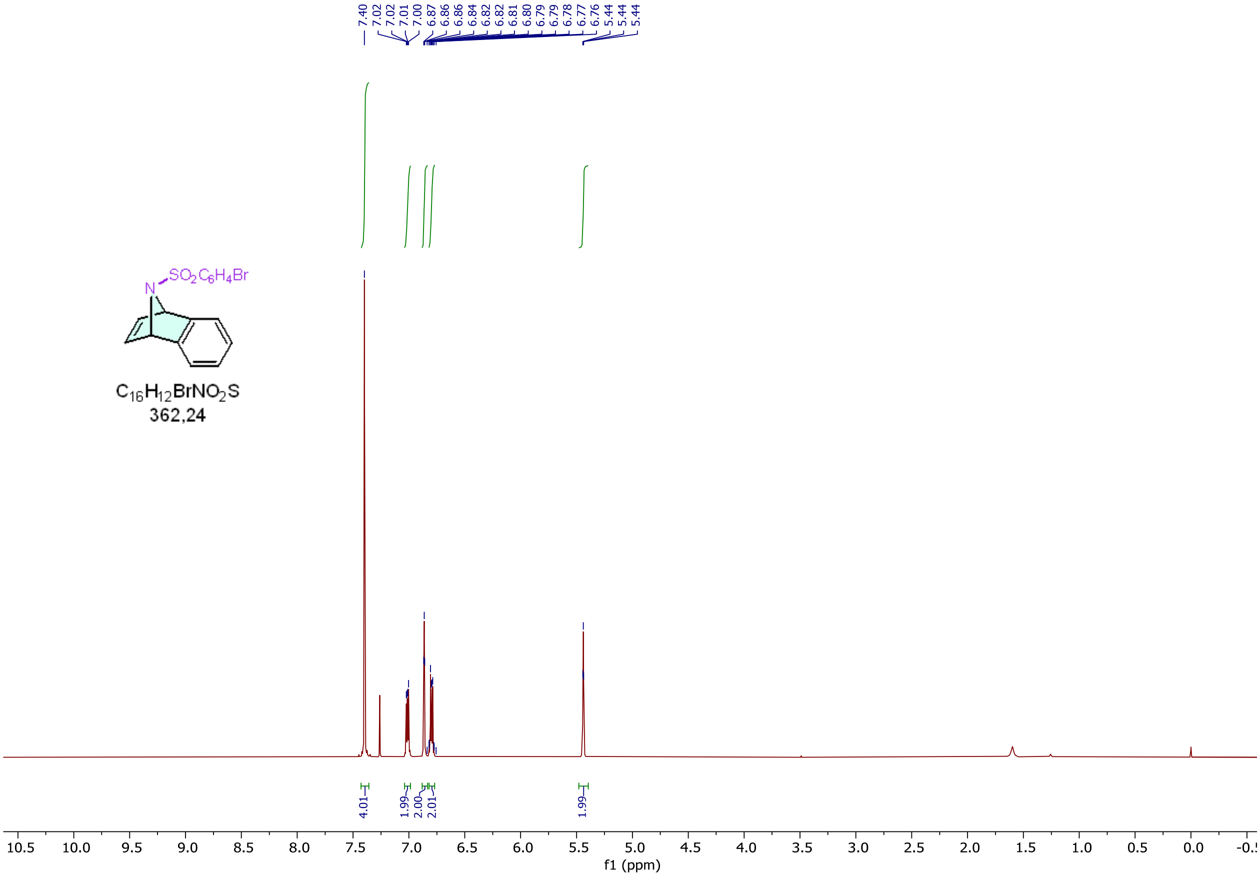

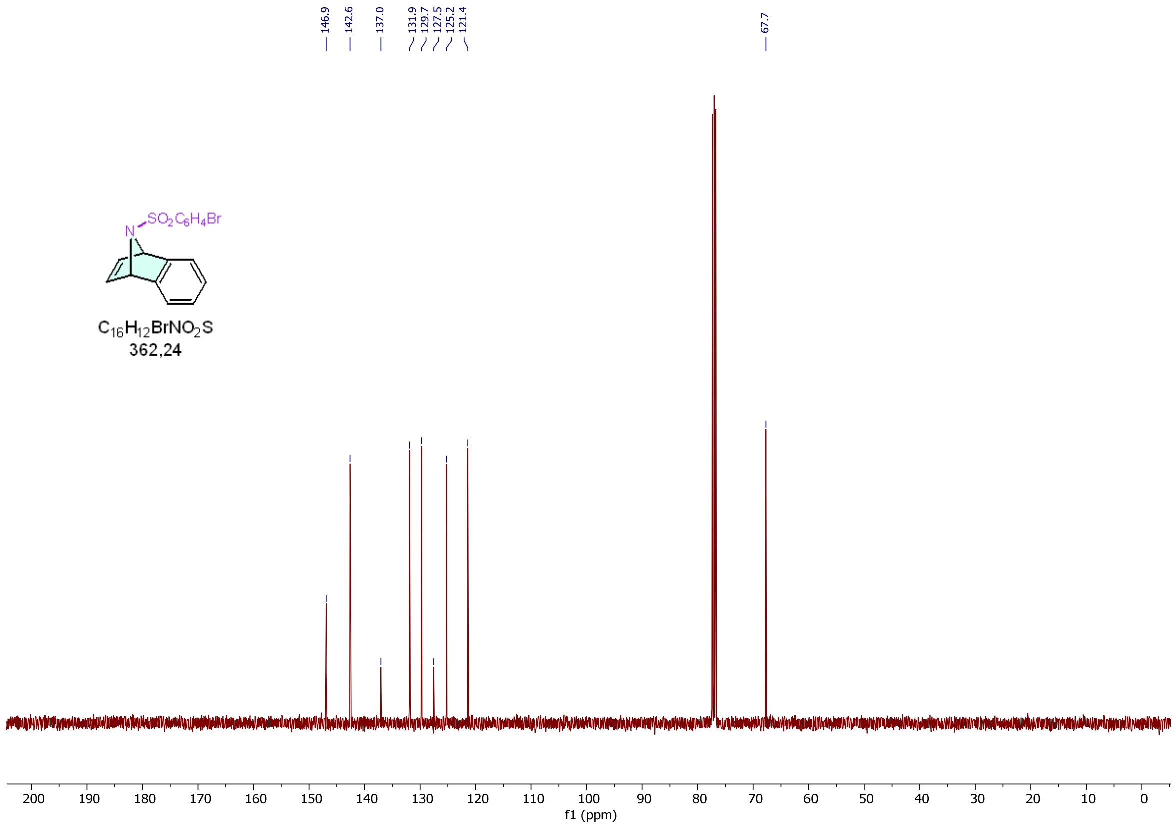

^{1}H NMR (400 MHz, $CDCl_3$) and ^{13}C NMR (101 MHz, $CDCl_3$) Analysis of Compound **6*N*-BsCl**

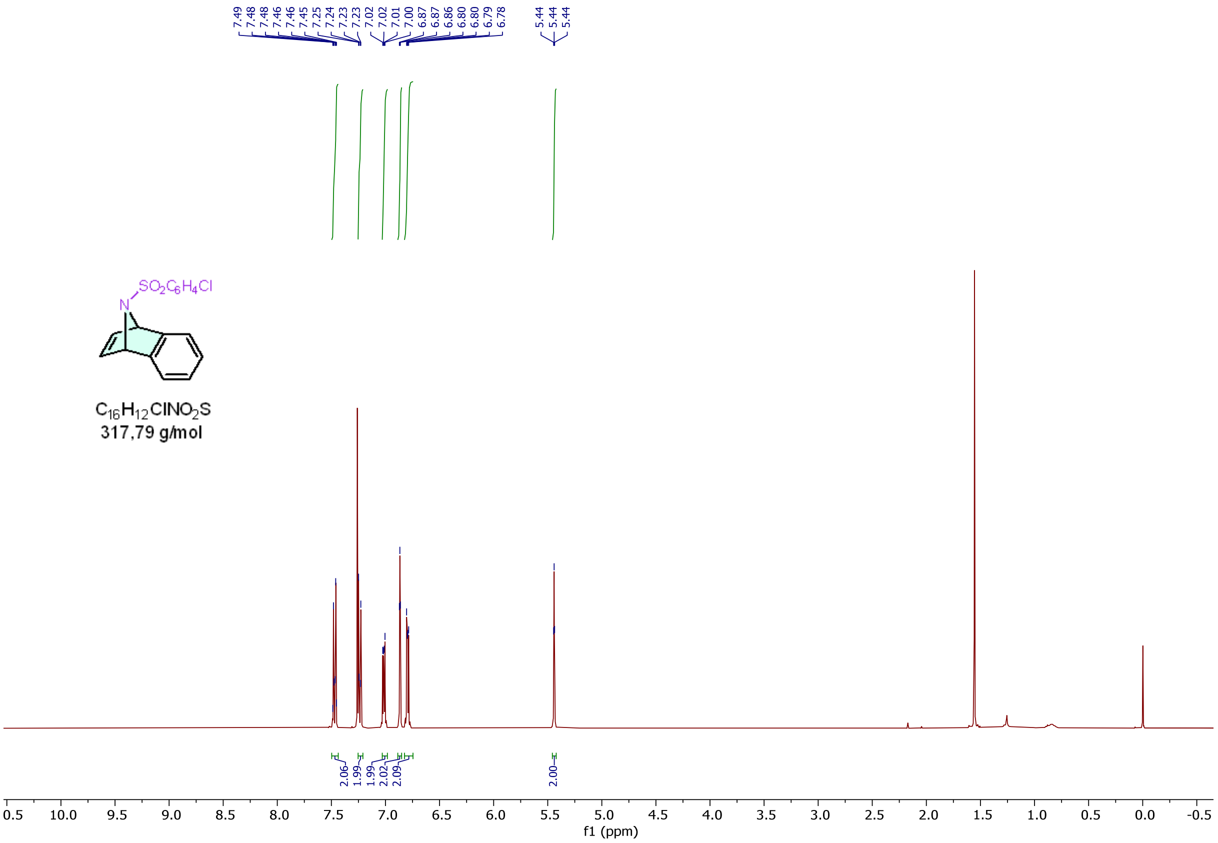

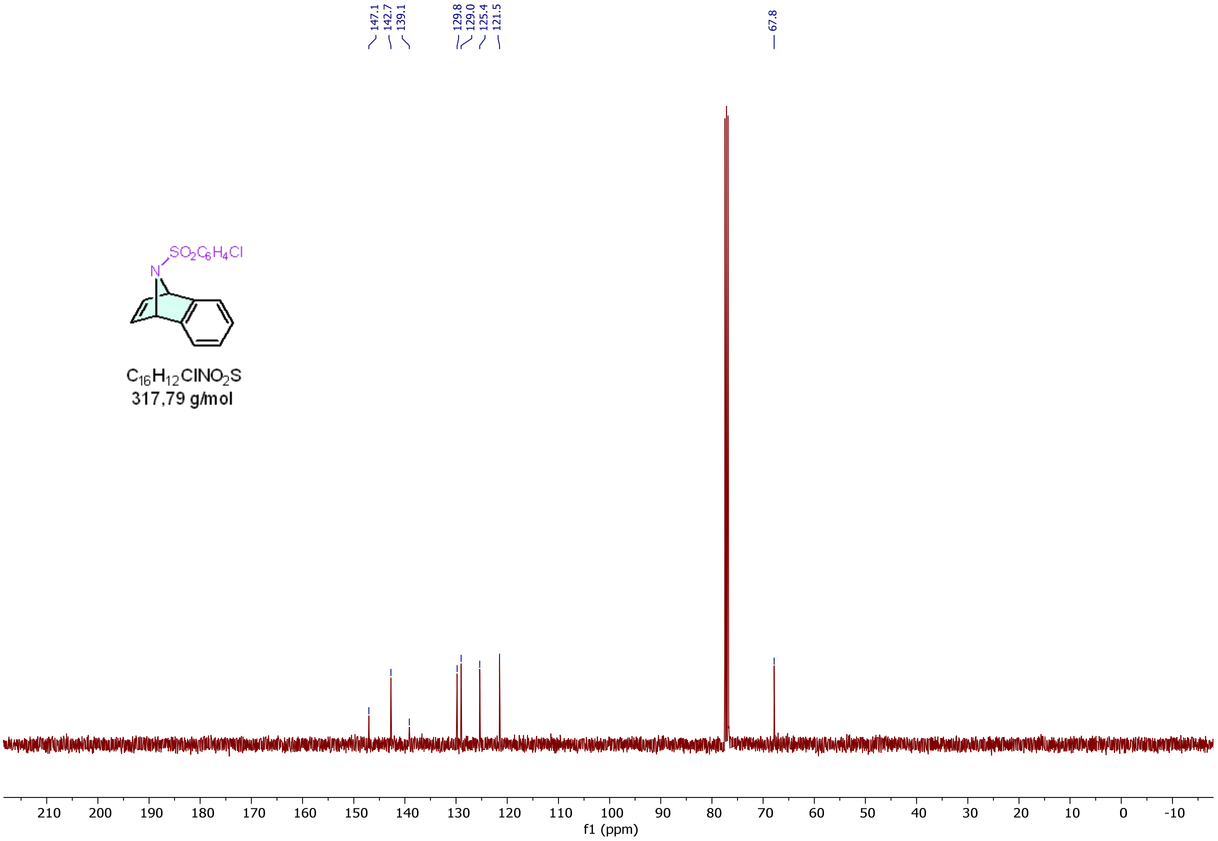

^1H NMR (400 MHz, $CDCl_3$), ^{13}C NMR (101 MHz, $CDCl_3$) and ^{19}F NMR (377 MHz, $CDCl_3$) Analysis of Compound **6*N*-BsF**

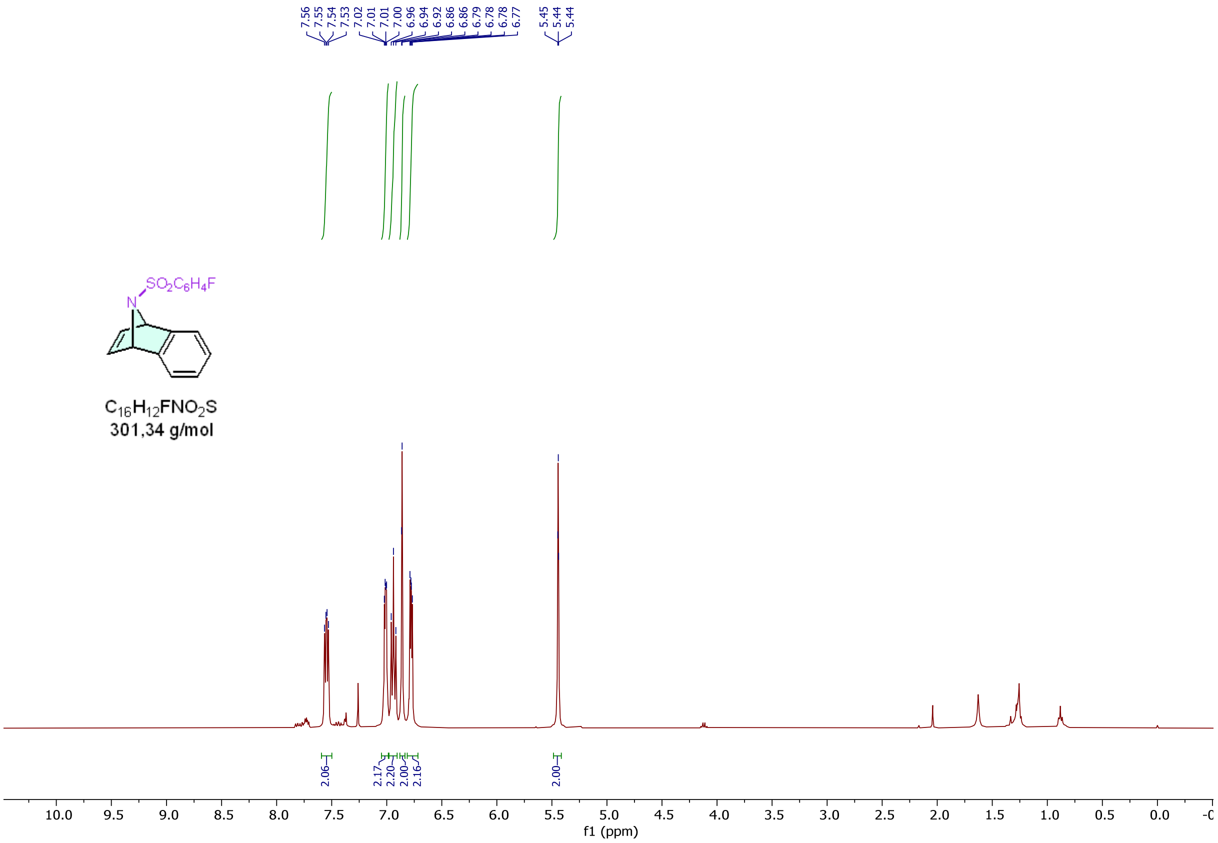

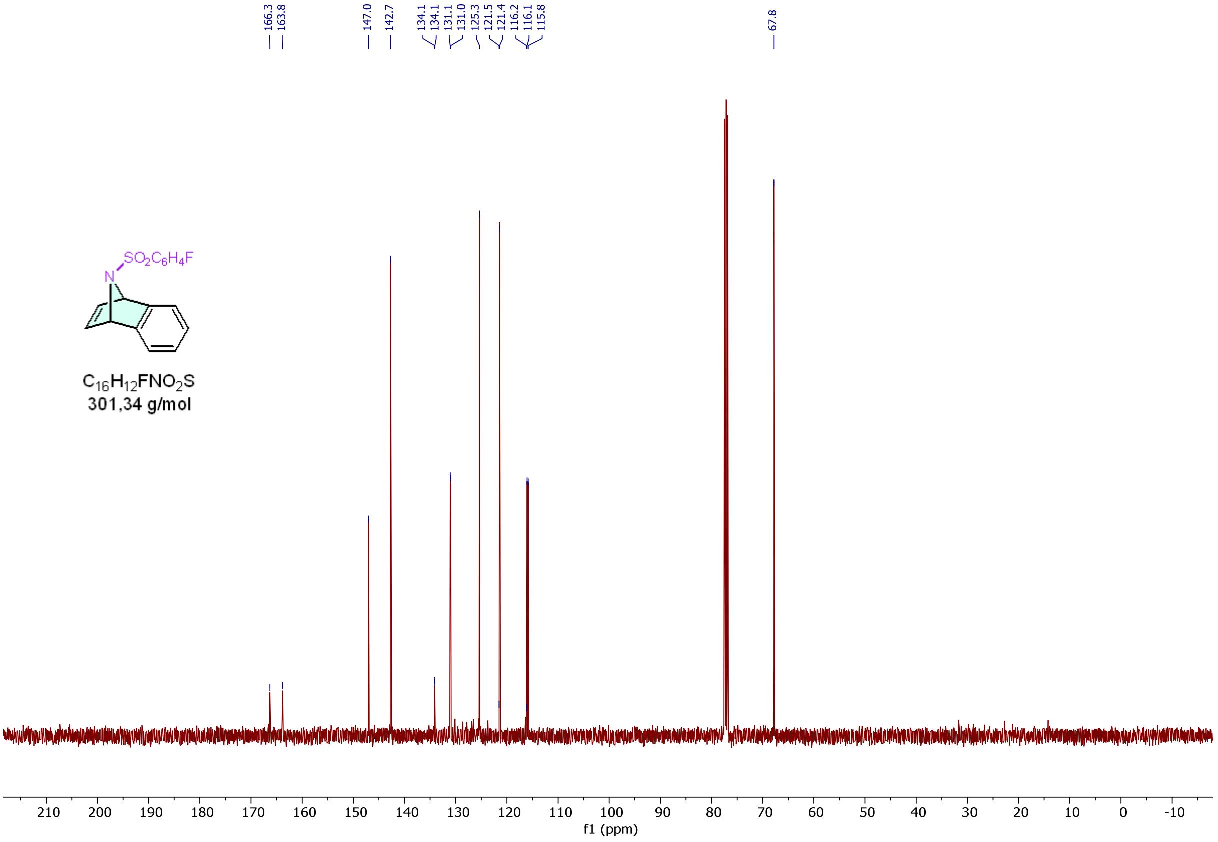

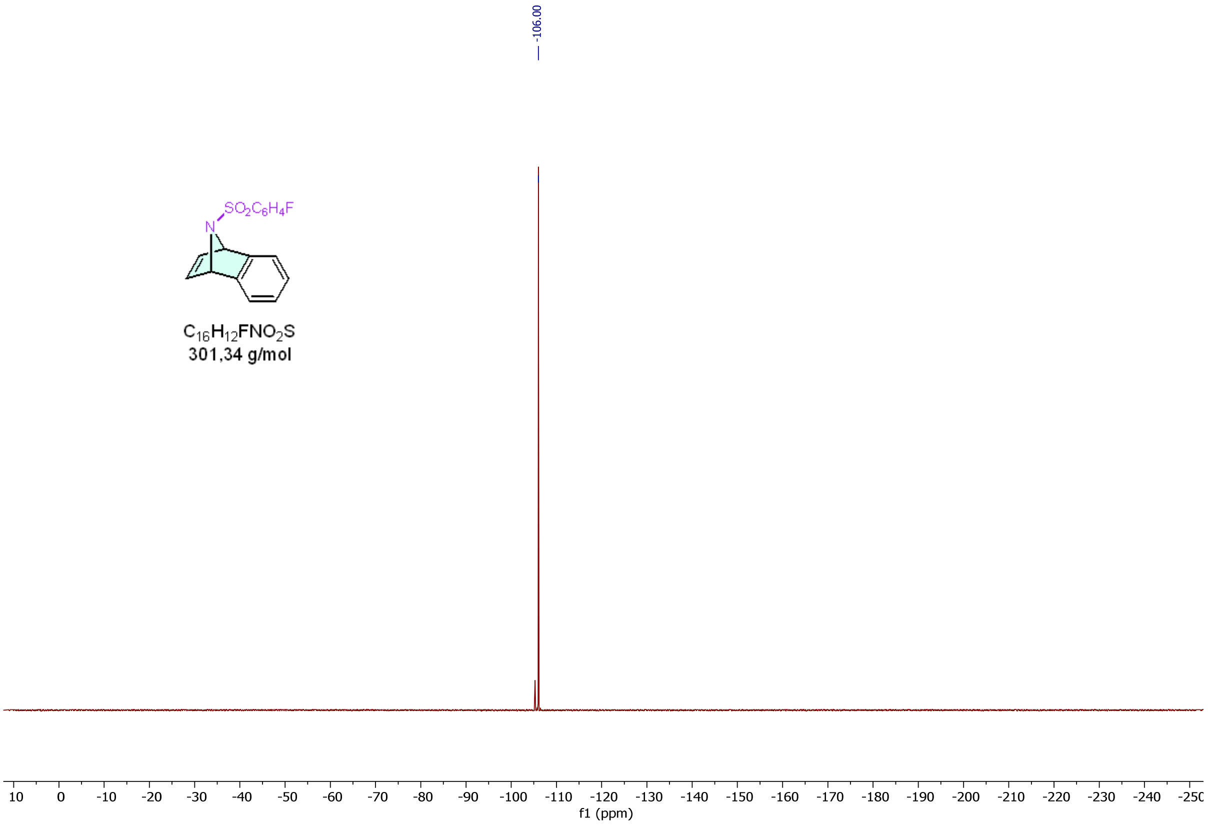
-106.00
$SO_2C_6H_4F$
N
$C_{16}H_{12}FNO_2S$
301,34 g/mol
10 0 -10 -20 -30 -40 -50 -60 -70 -80 -90 -100 -110 -120 -130 -140 -150 -160 -170 -180 -190 -200 -210 -220 -230 -240 -250
f1 (ppm)

^{1}H NMR (400 MHz, $CDCl_3$) and ^{13}C NMR (101 MHz, $CDCl_3$) NMR Analysis of Compound **6b**

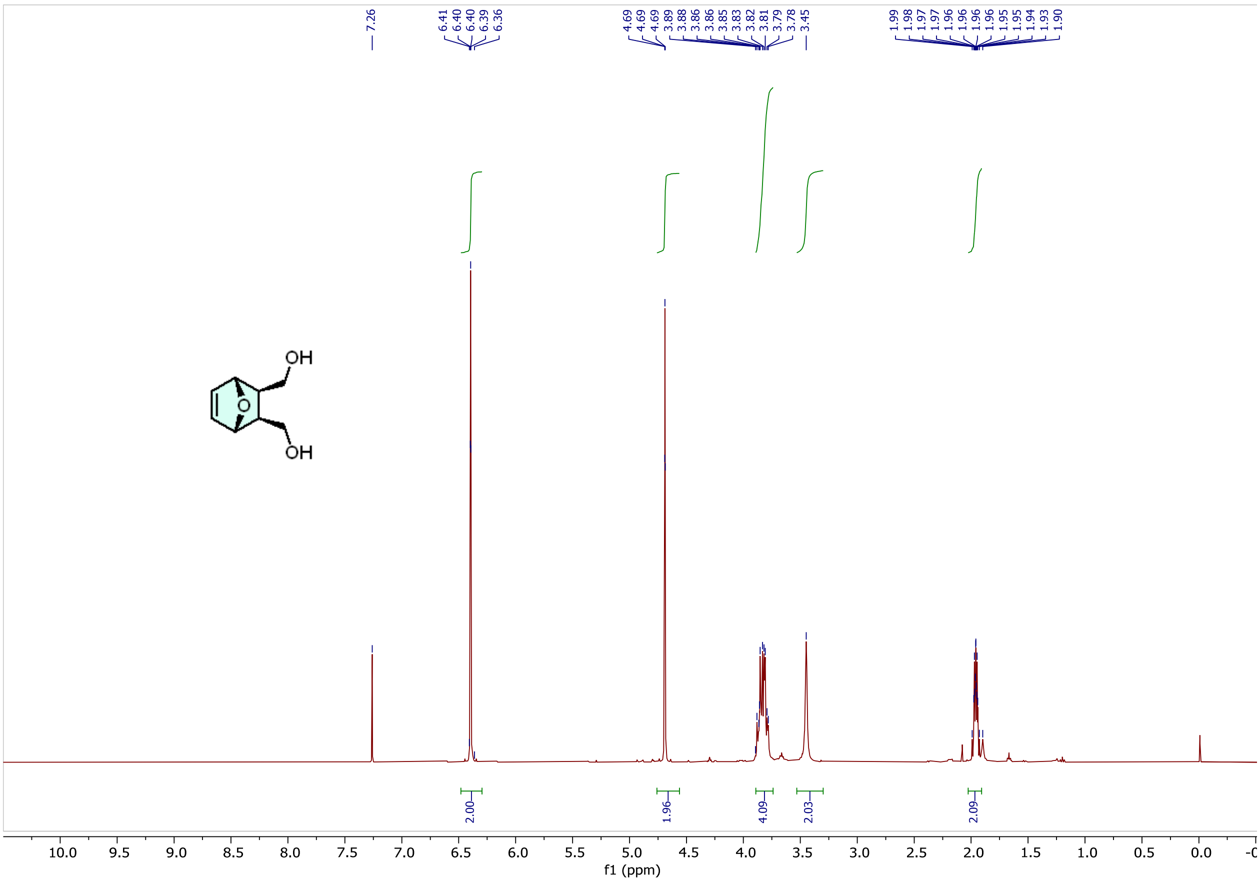

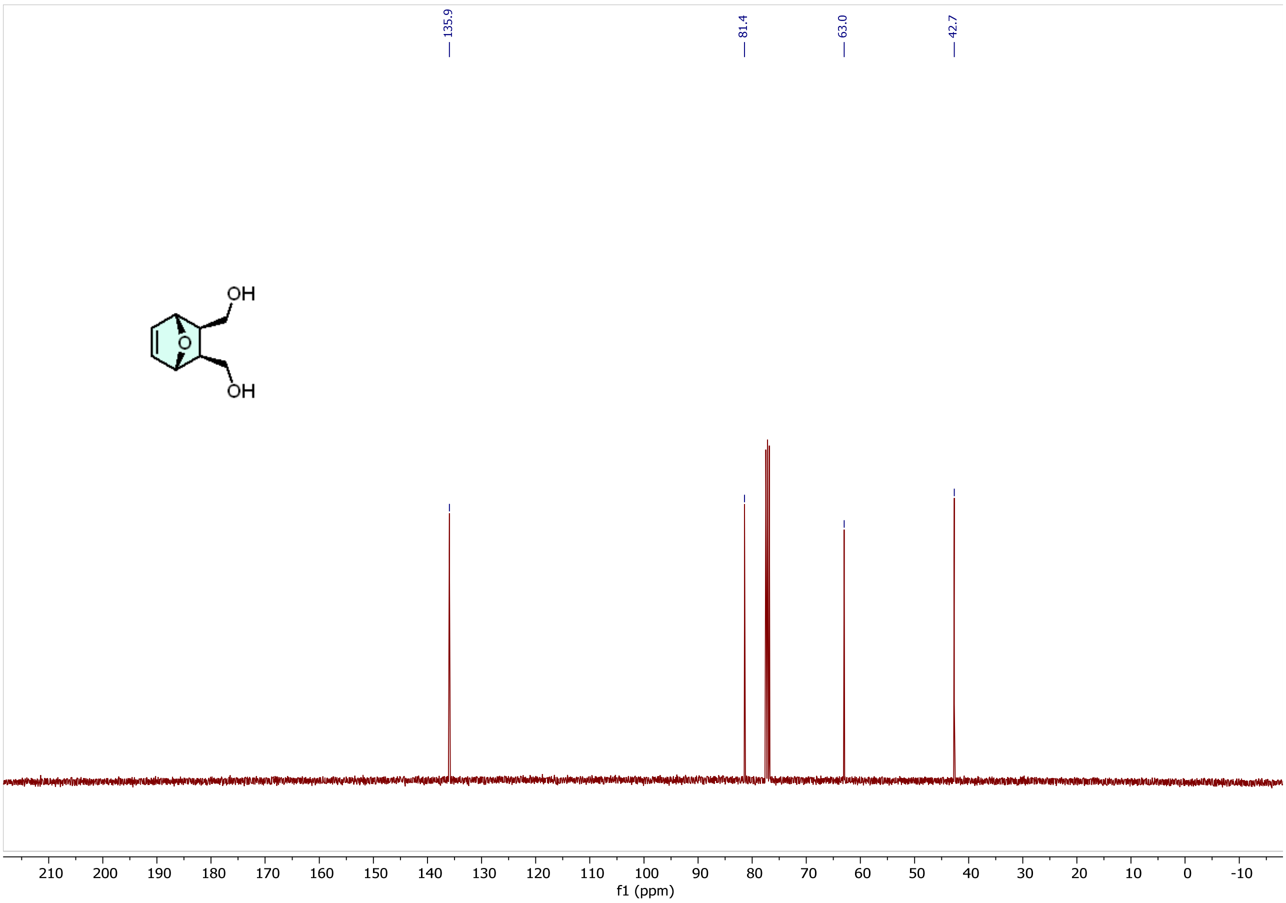

^{1}H NMR (400 MHz, $CDCl_3$) and ^{13}C NMR (101 MHz, $CDCl_3$) Analysis of Compound **6c**

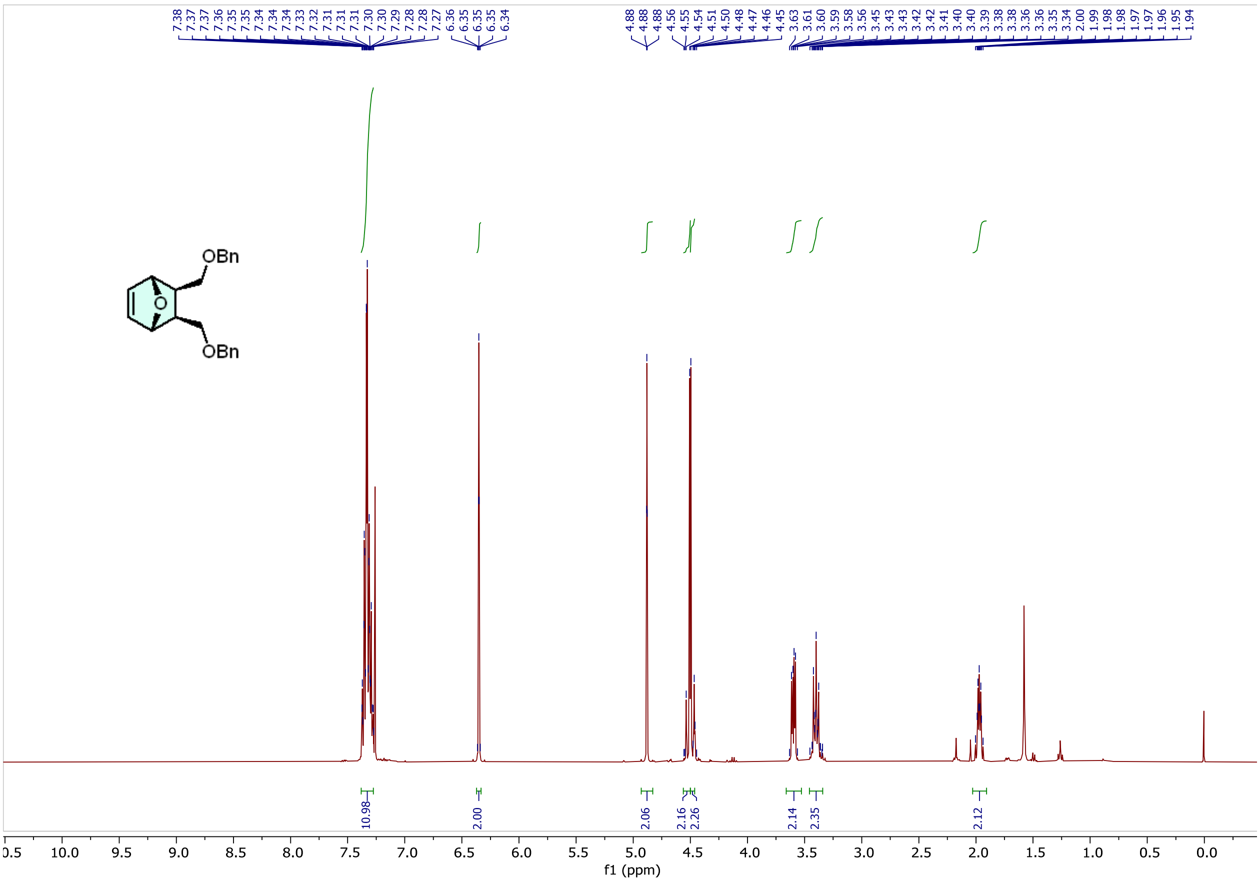

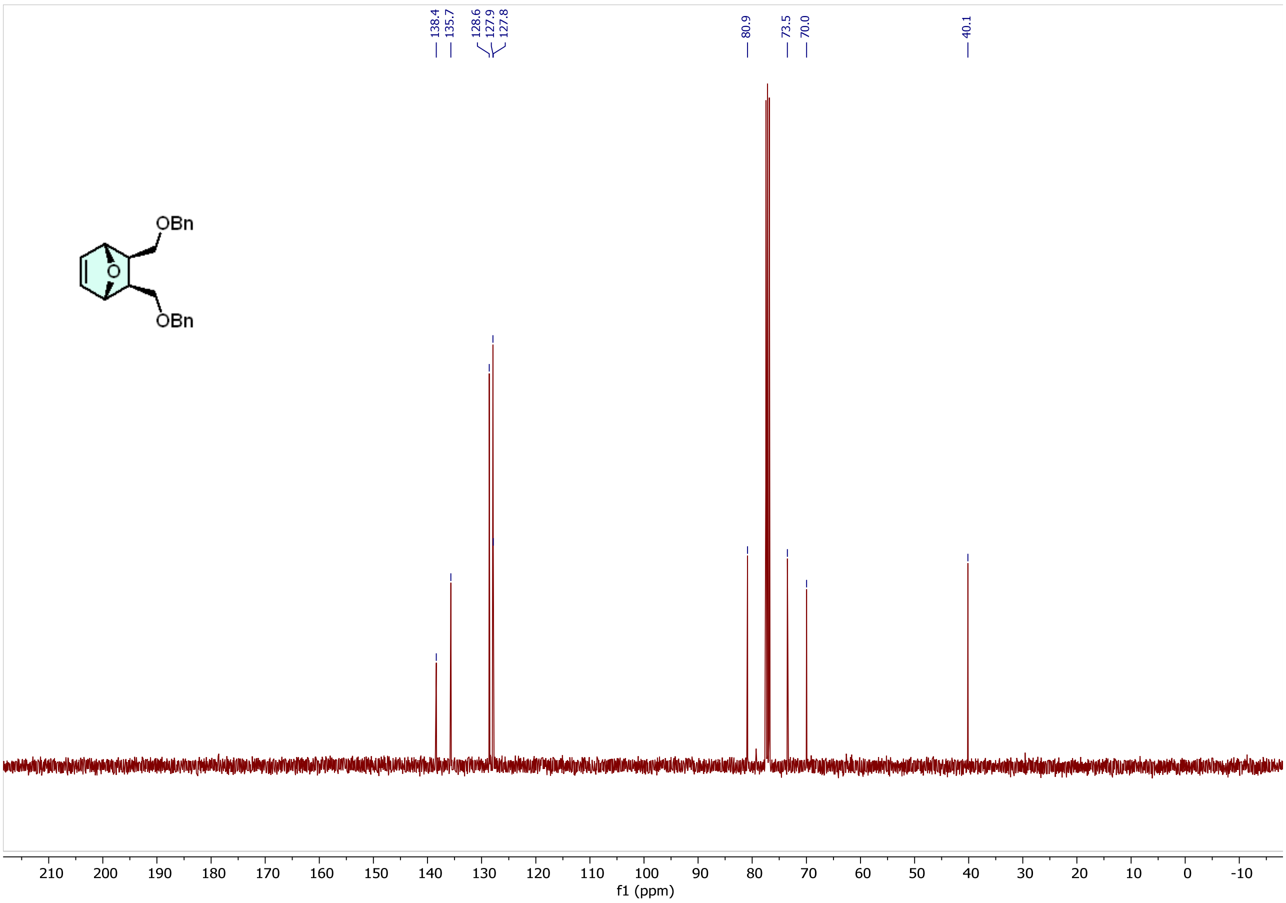

^{1}H NMR (400 MHz, $CDCl_3$) and ^{13}C NMR (101 MHz, $CDCl_3$)Analysis of Compound **7aa**

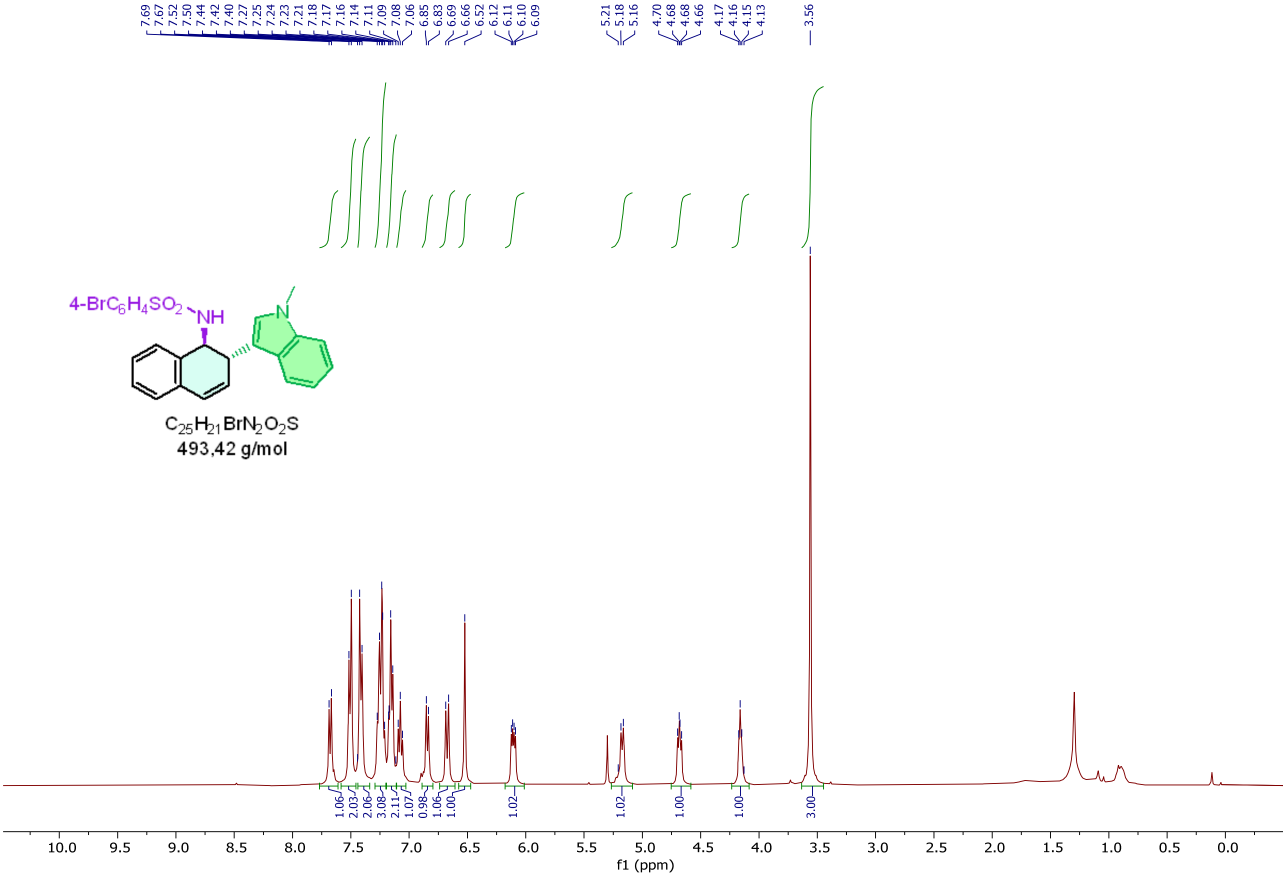

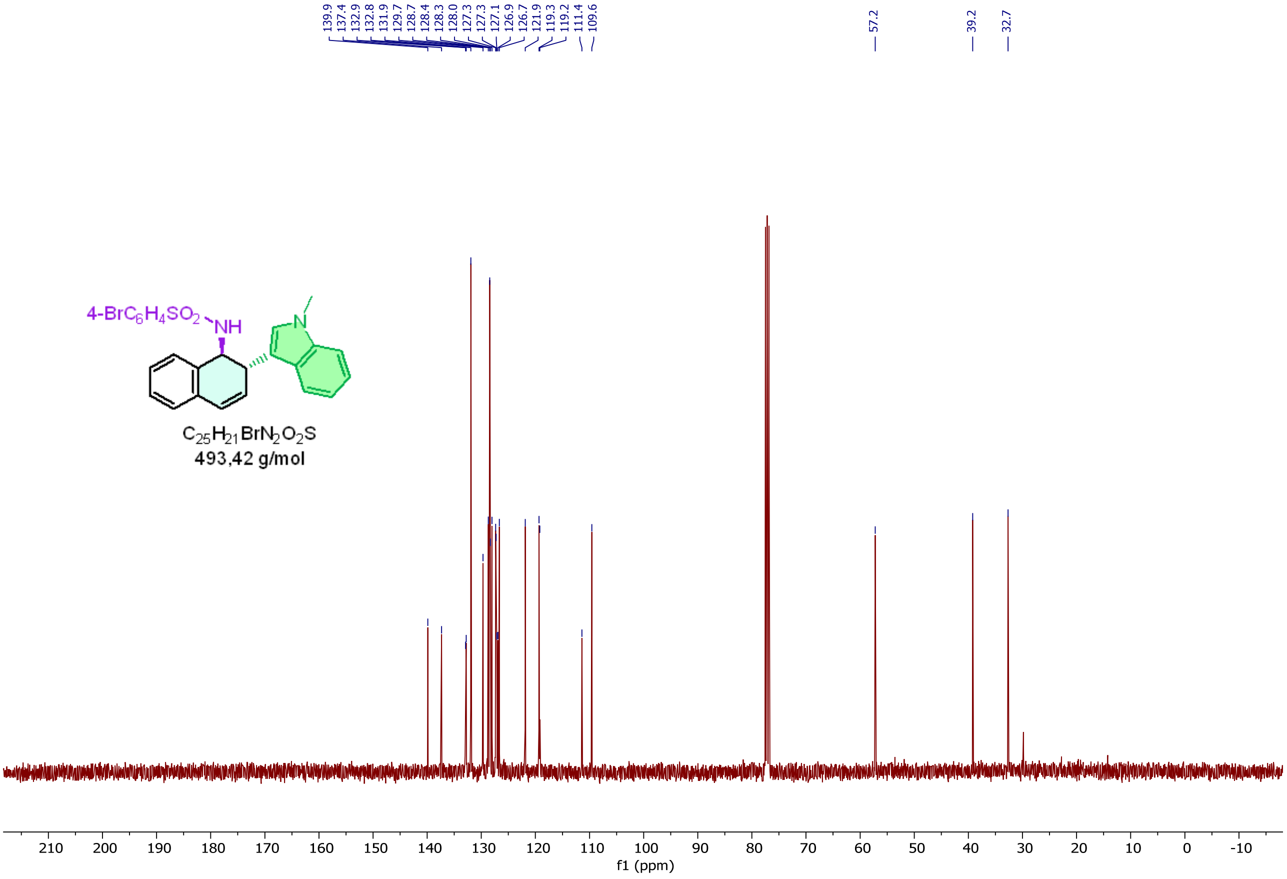

^{1}H NMR (400 MHz, $CDCl_3$), ^{13}C NMR (101 MHz, $CDCl_3$) and ^{19}F NMR (377 MHz, $CDCl_3$) Analysis of Compound **7ab**

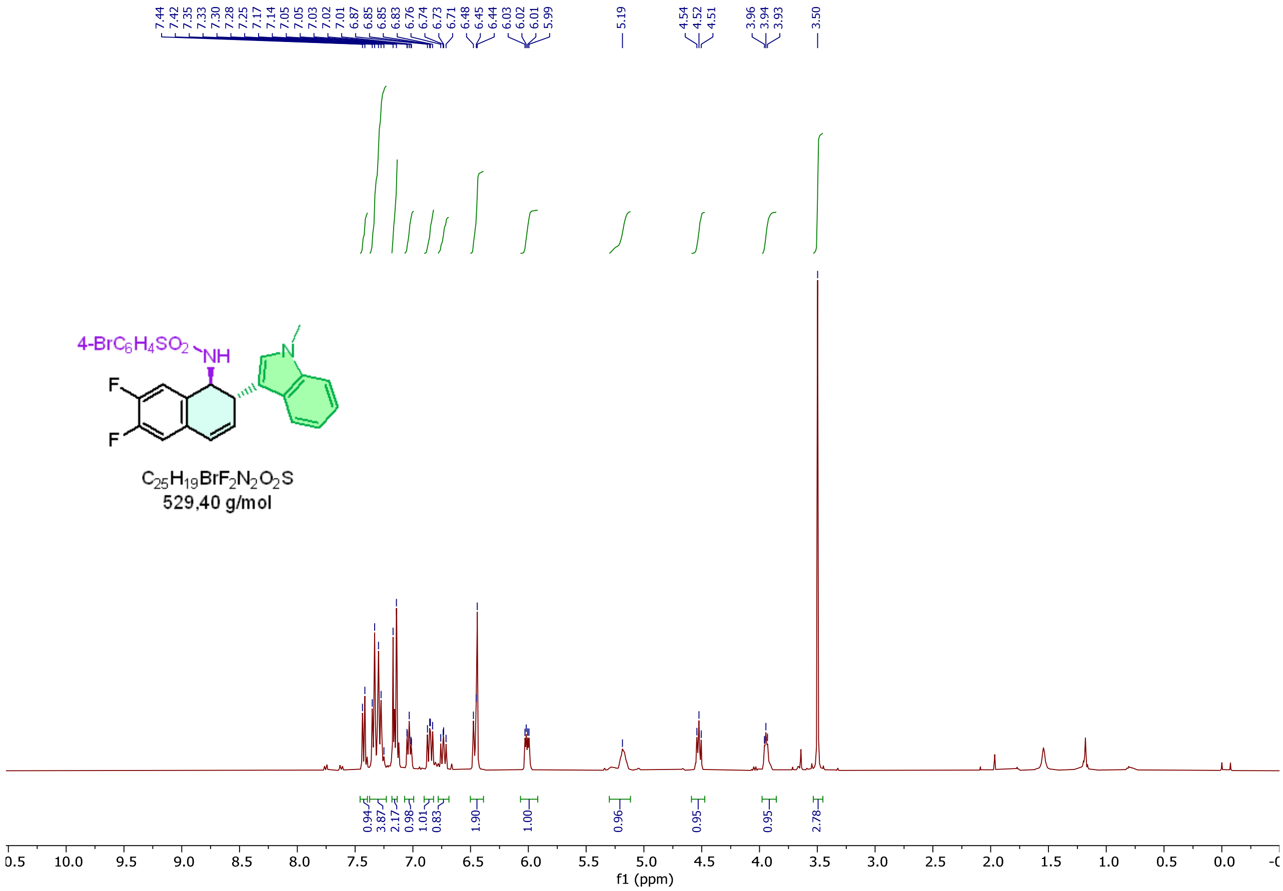

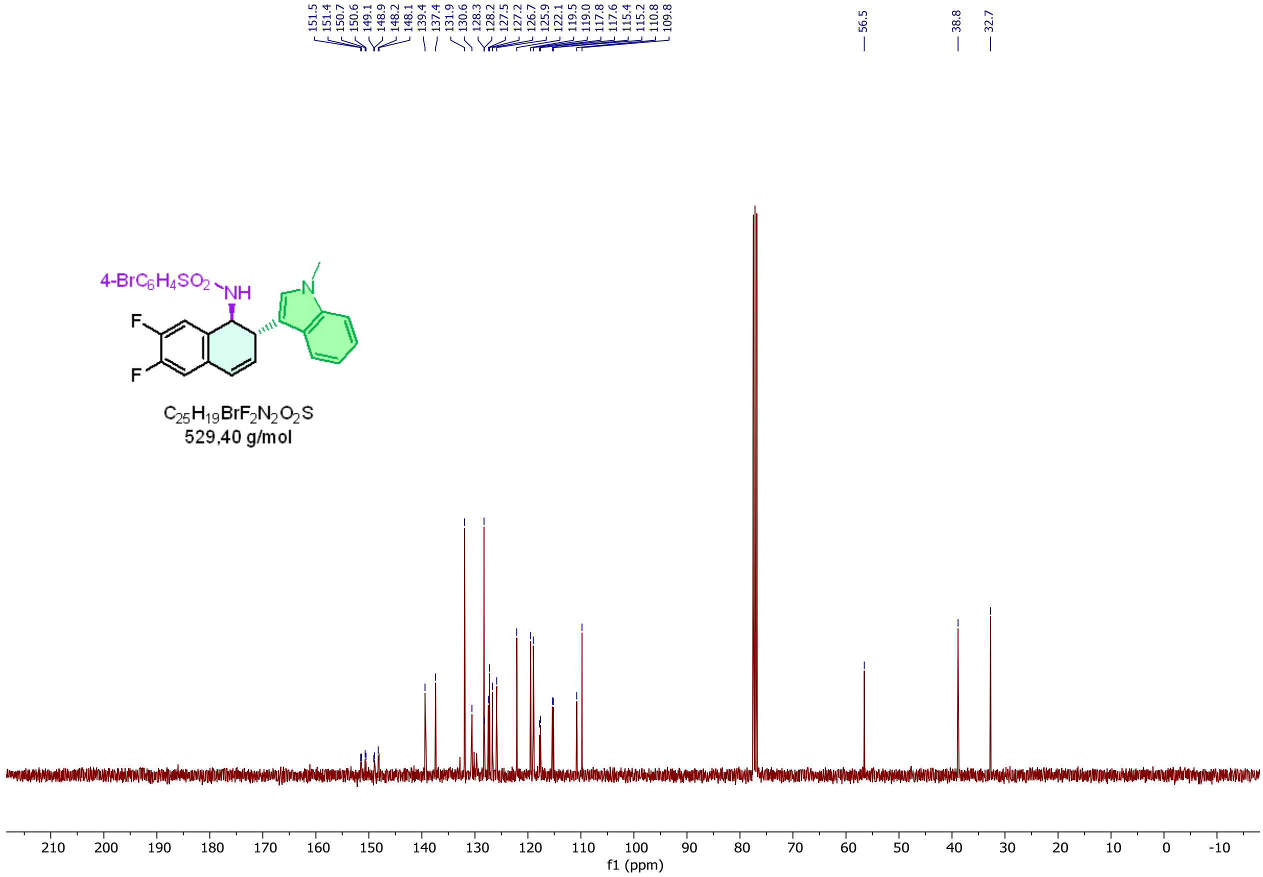

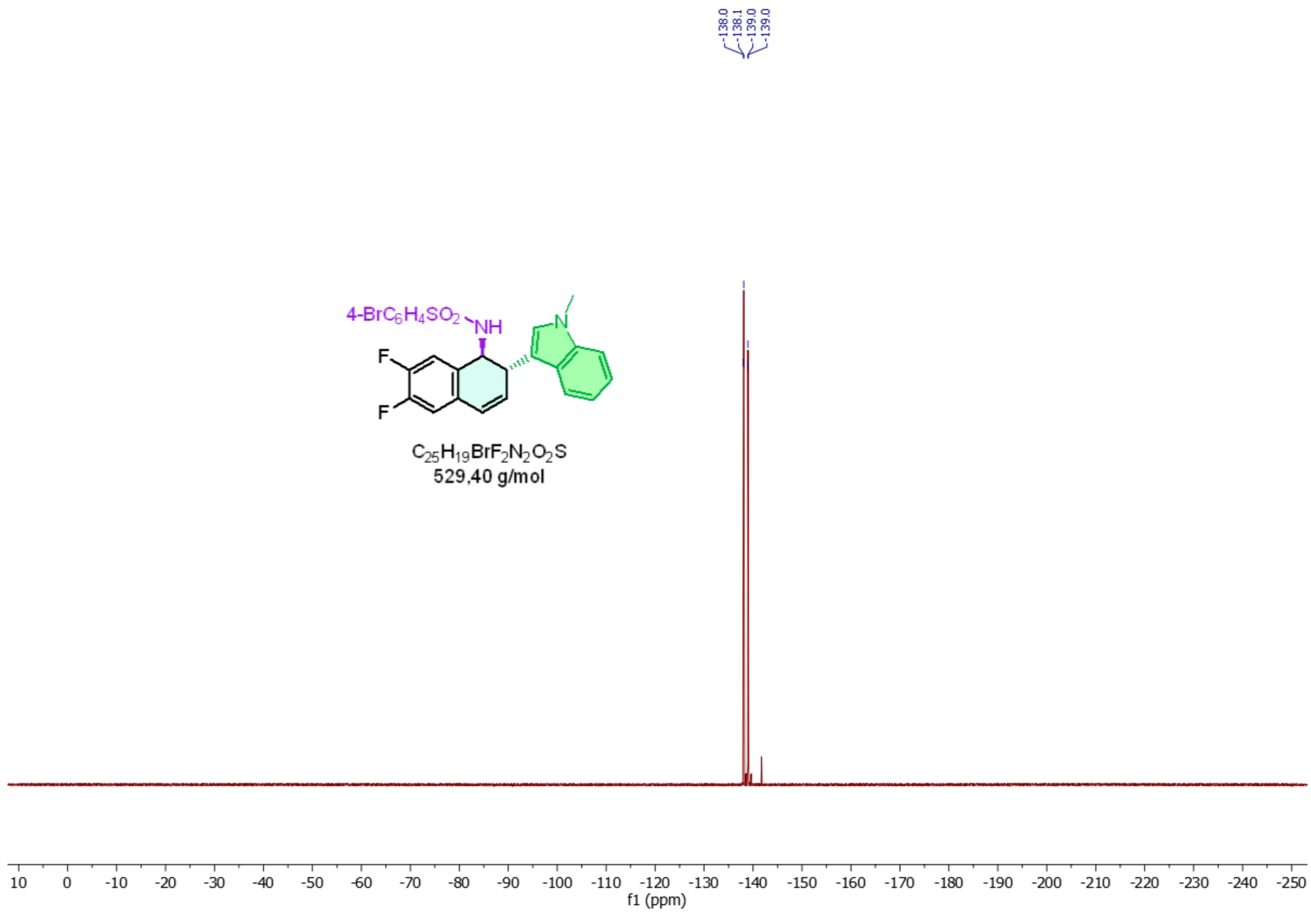
-138.0
-138.1
-139.0
-139.0
4-BrC6H4SO2
NH
N
F
F
C25H19BrF2N2O2S
529,40 g/mol
10
0
-10
-20
-30
-40
-50
-60
-70
-80
-90
-100
-110
-120
-130
-140
-150
-160
-170
-180
-190
-200
-210
-220
-230
-240
-250
f1 (ppm)

^{1}H NMR (400 MHz, $CDCl_3$) and ^{13}C NMR (101 MHz, $CDCl_3$) Analysis of Compound **7b**

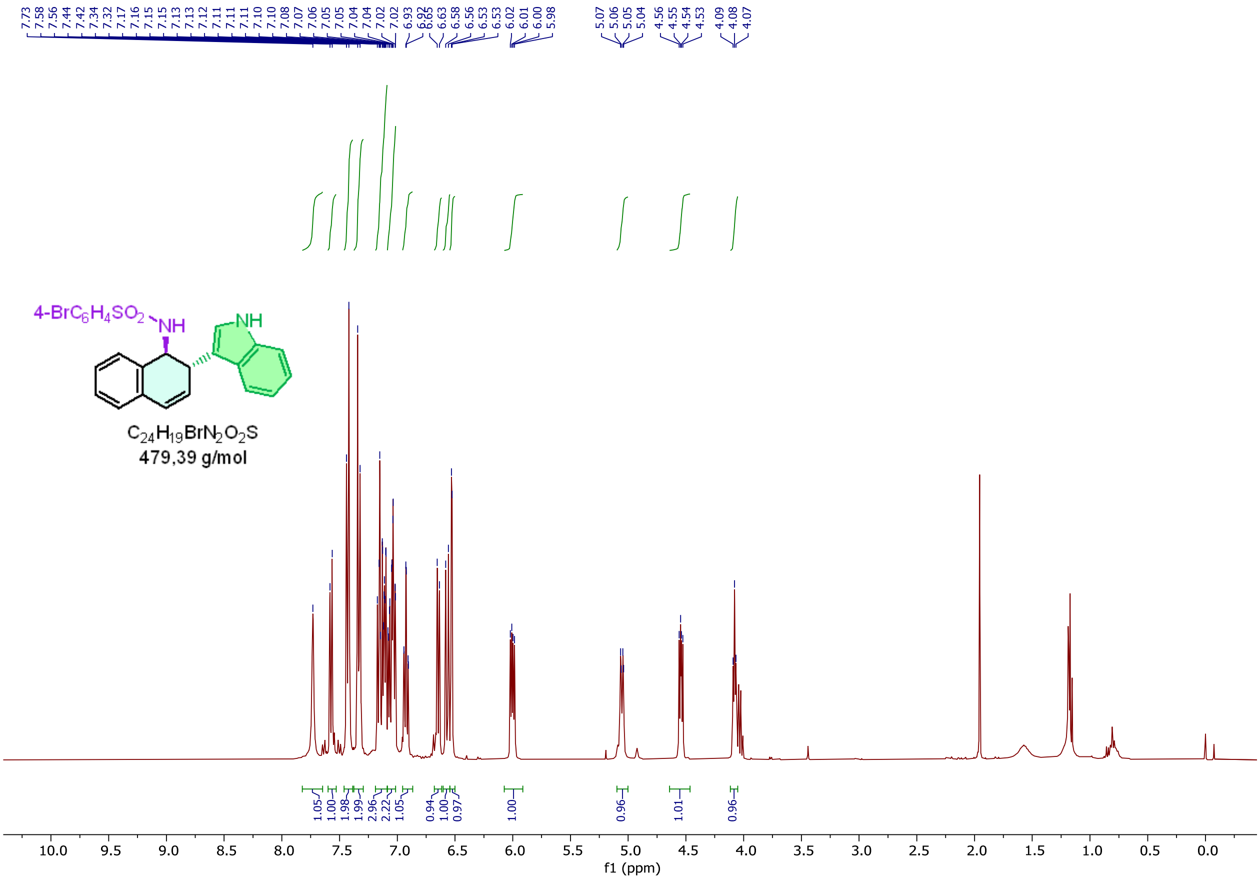

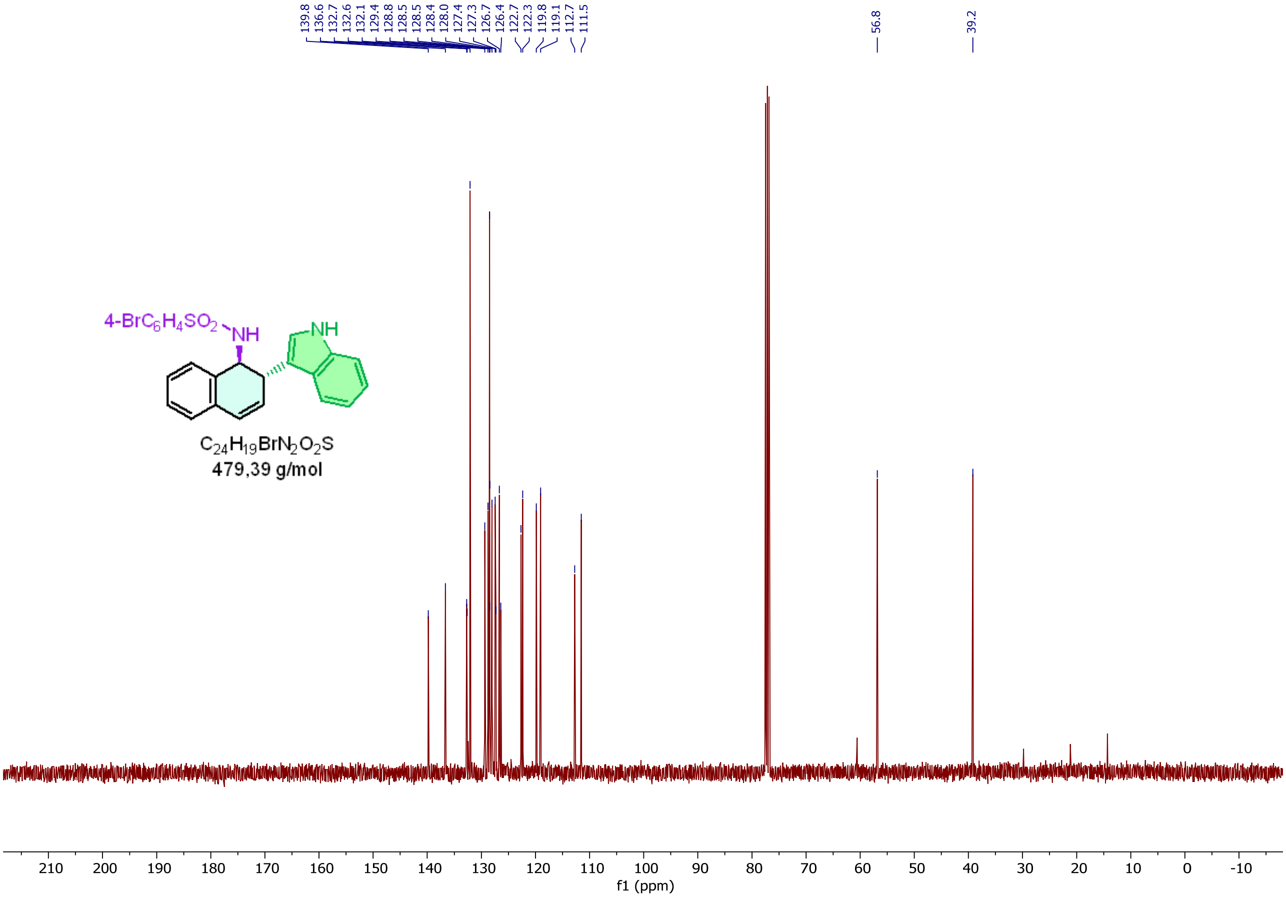

^{1}H NMR (400 MHz, acetone-d_6) and ^{13}C NMR (101 MHz, acetone-d_6) Analysis of Compound **7c**

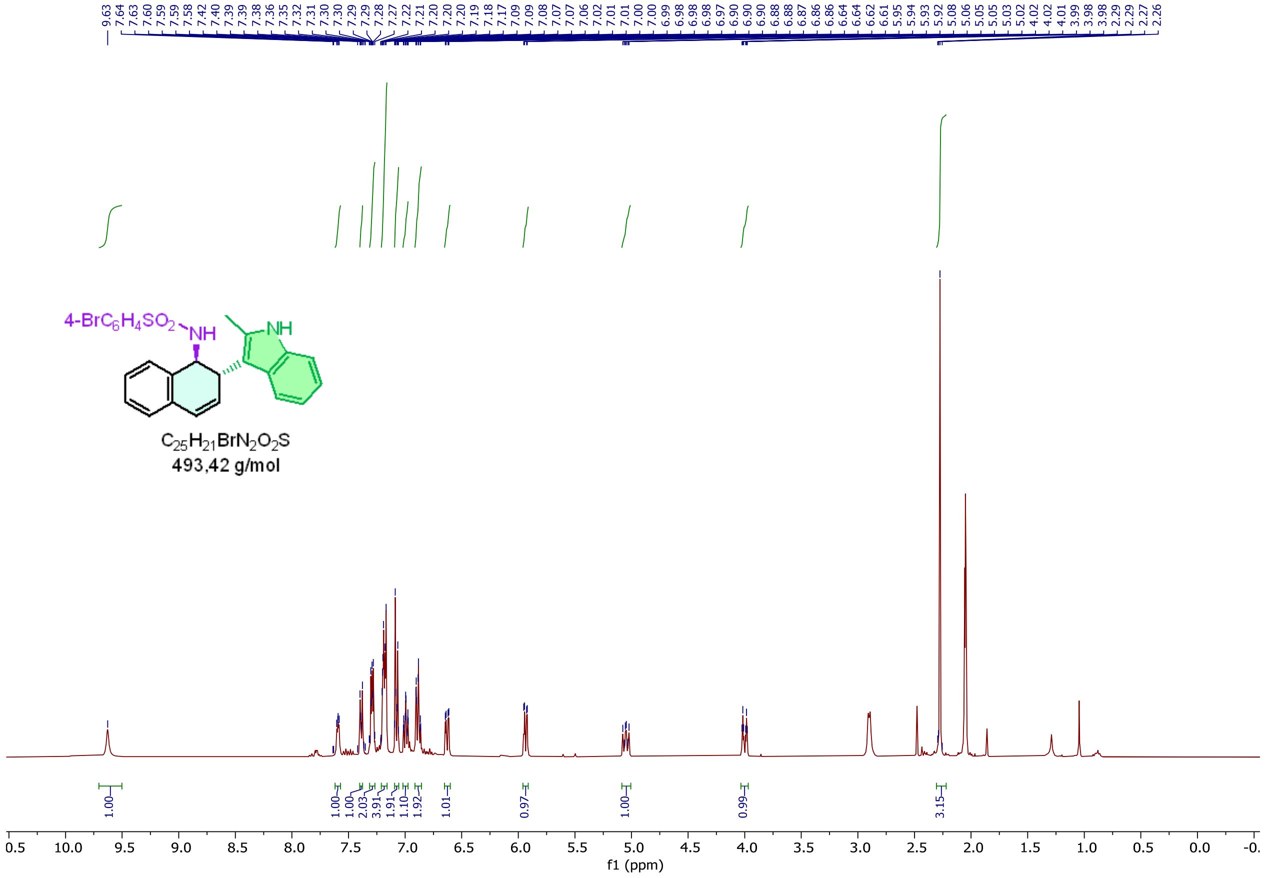

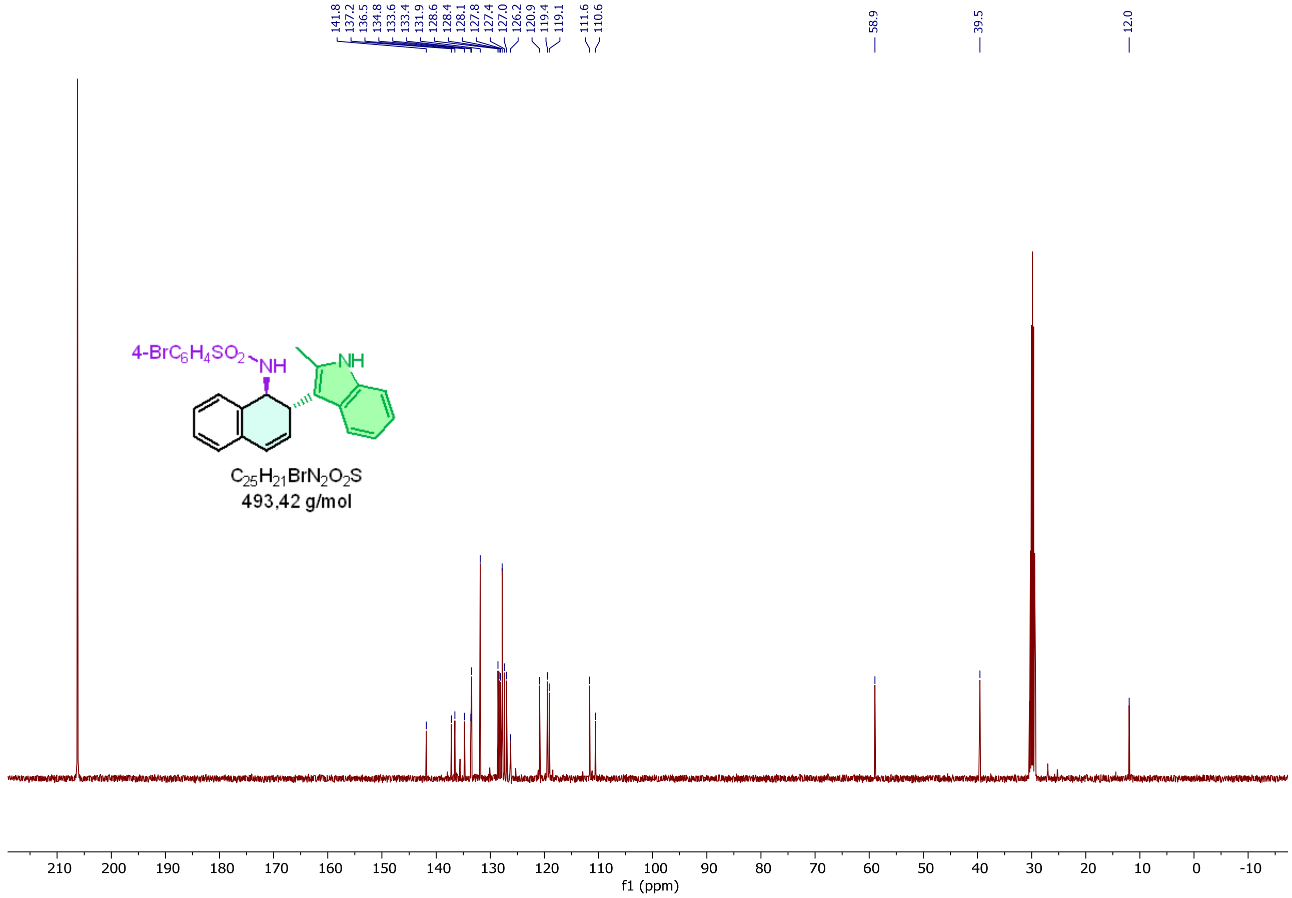

^{1}H NMR (400 MHz, $CDCl_3$) and ^{13}C NMR (101 MHz, $CDCl_3$) Analysis of Compound **7d**

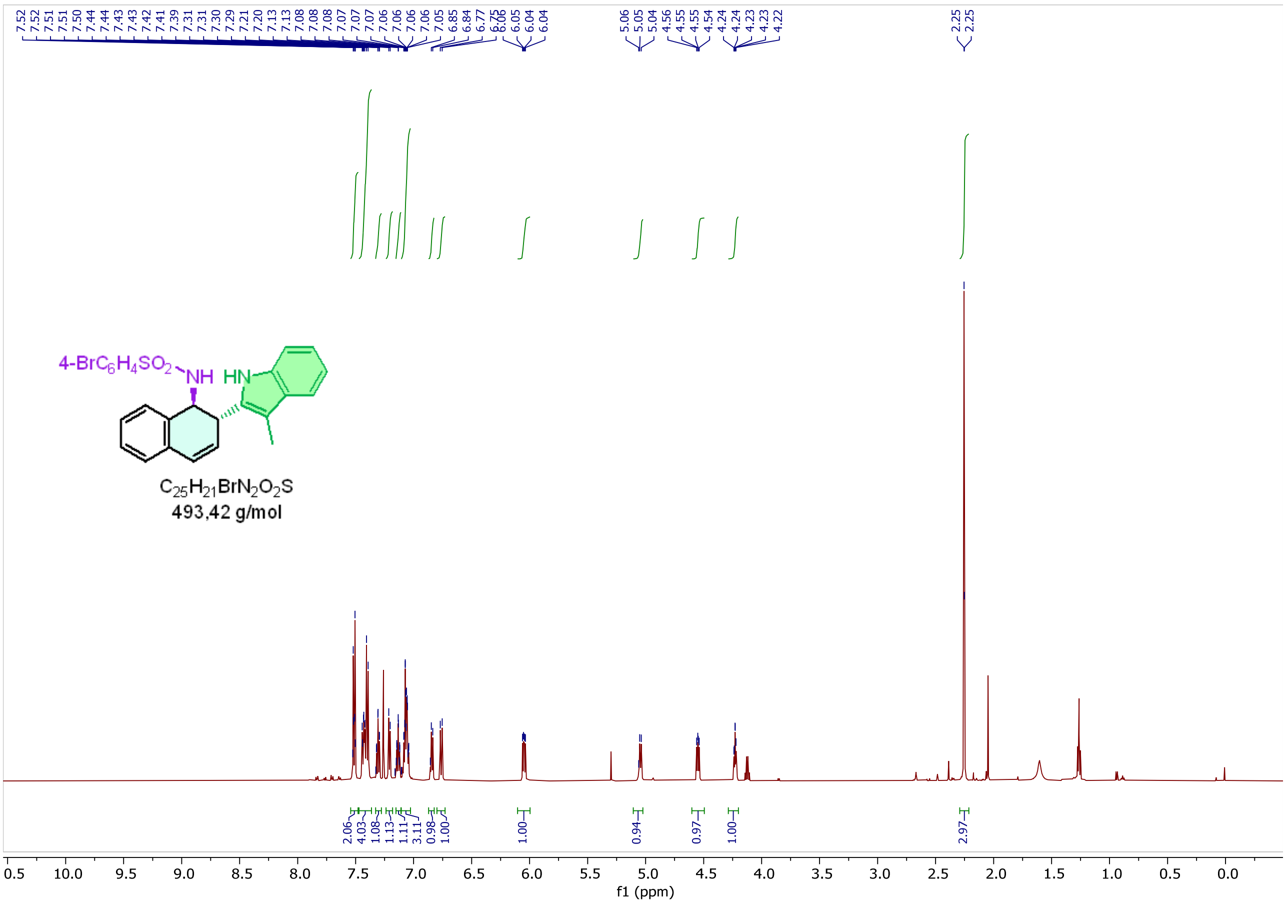

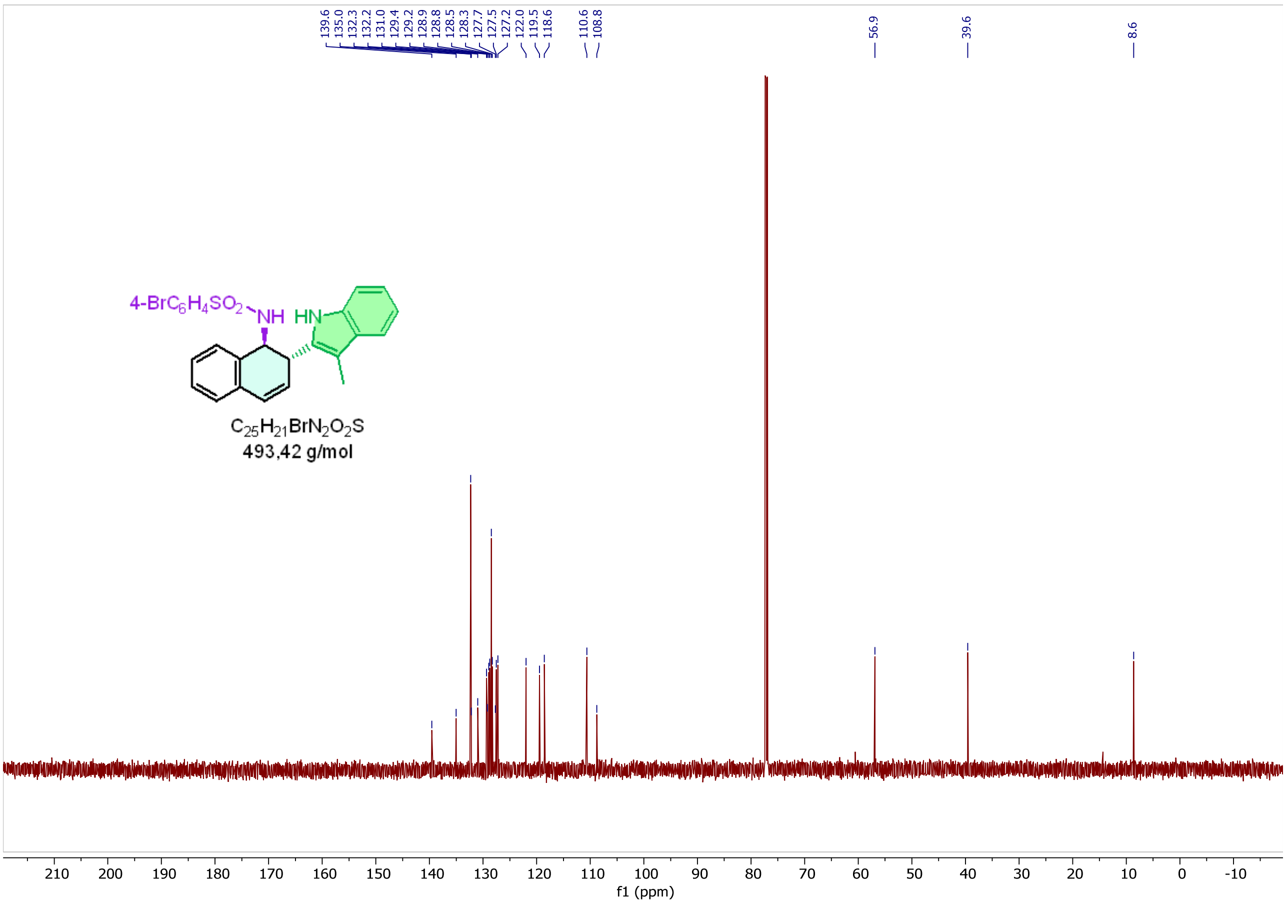

^{1}H NMR (400 MHz, $CDCl_3$) and ^{13}C NMR (101 MHz, $CDCl_3$) Analysis of Compound **7ea**

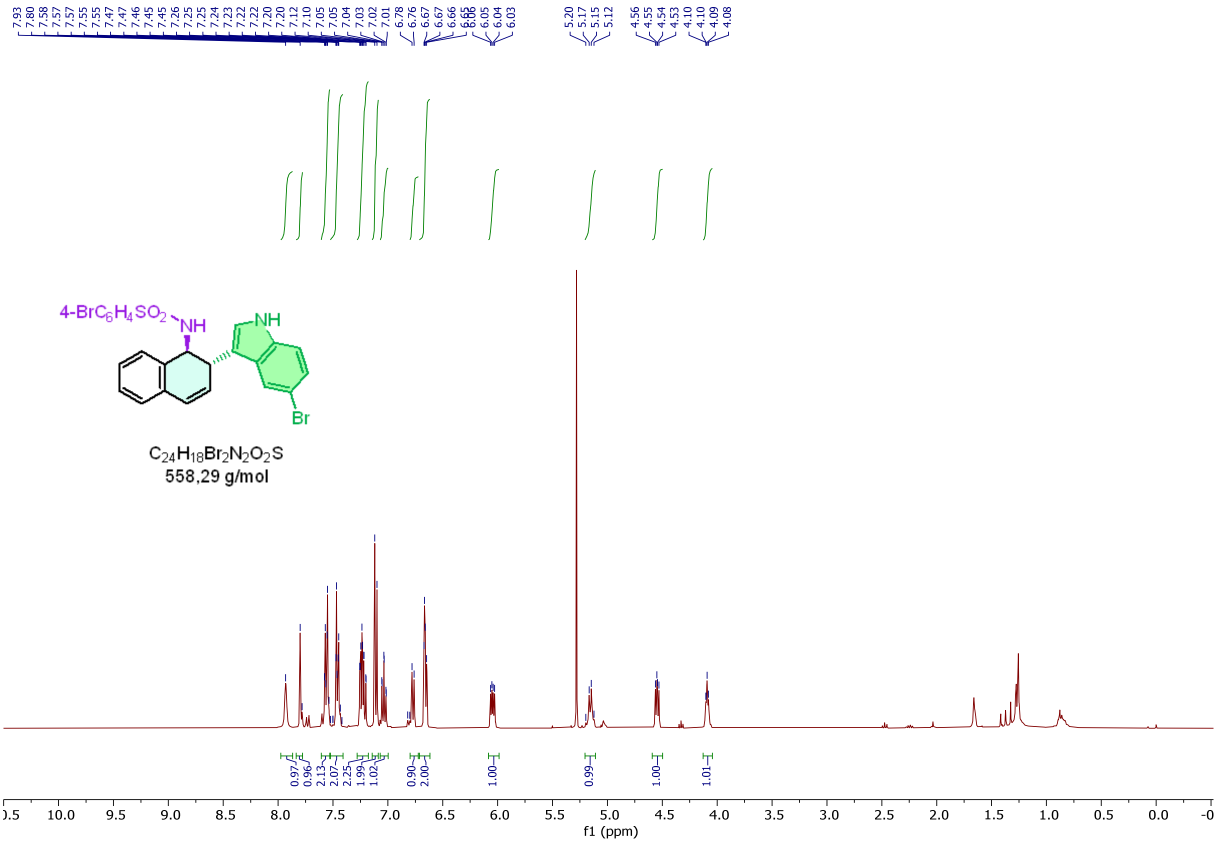

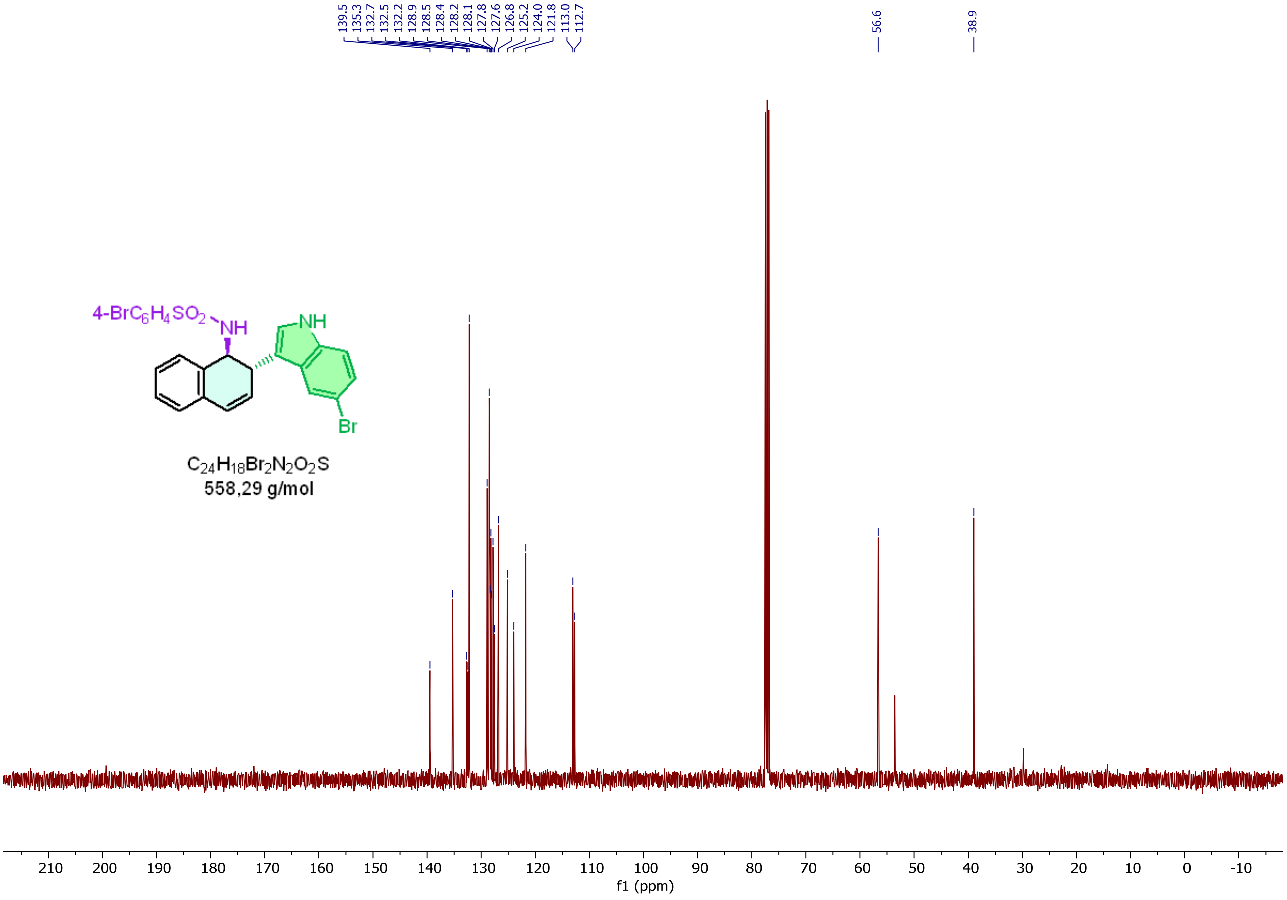

^{1}H NMR (400 MHz, $CDCl_3$), ^{13}C NMR (101 MHz, $CDCl_3$) and ^{19}F NMR (377 MHz, $CDCl_3$) Analysis of Compound **7eb**

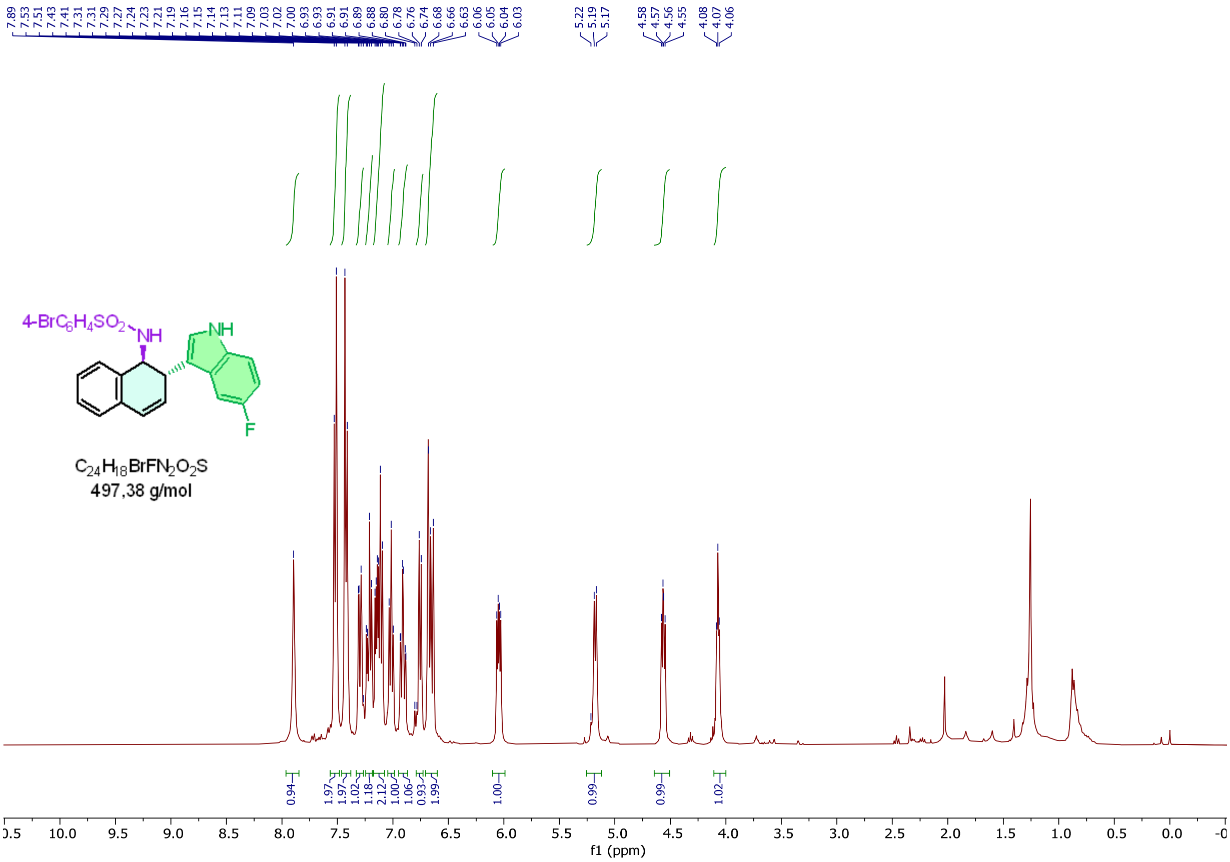

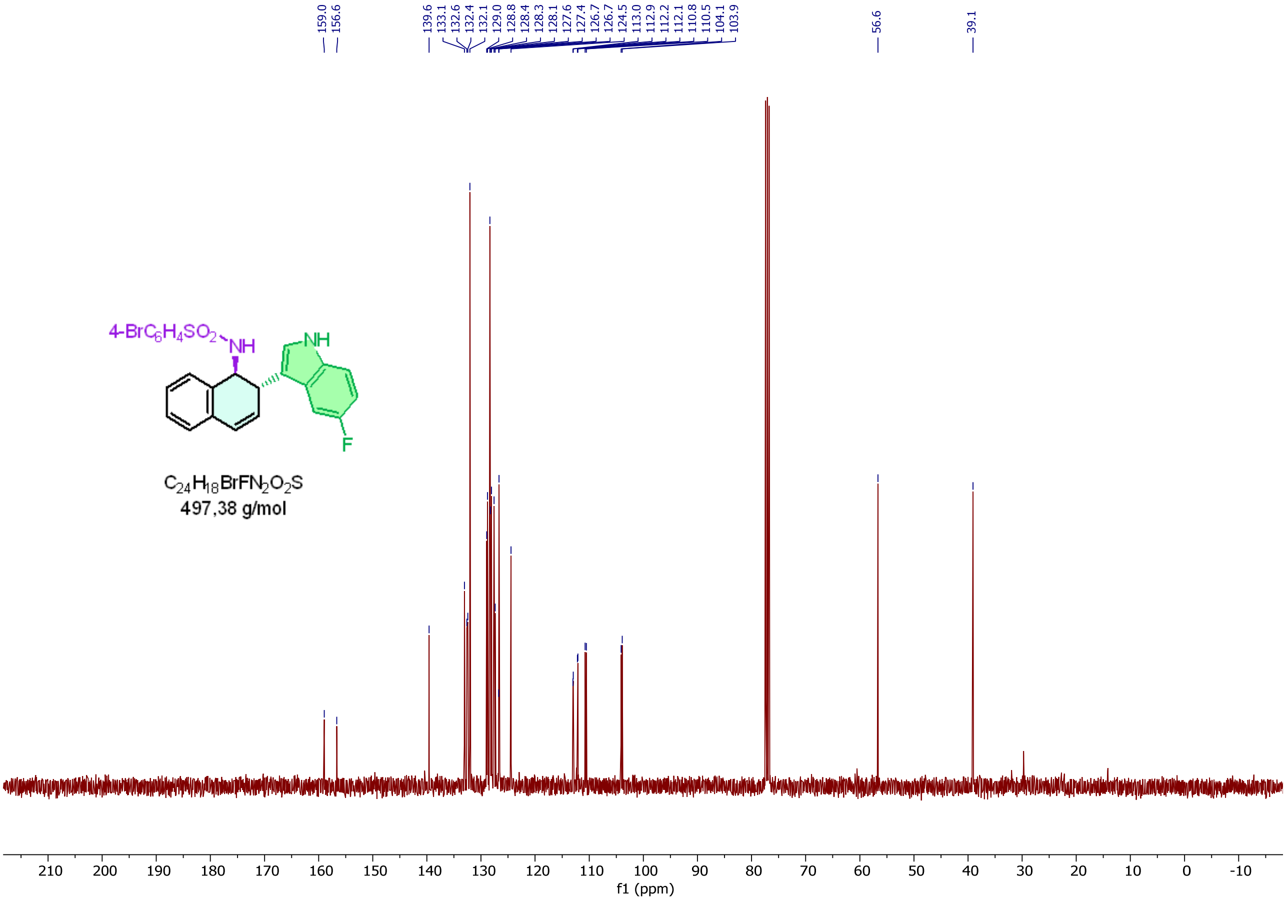

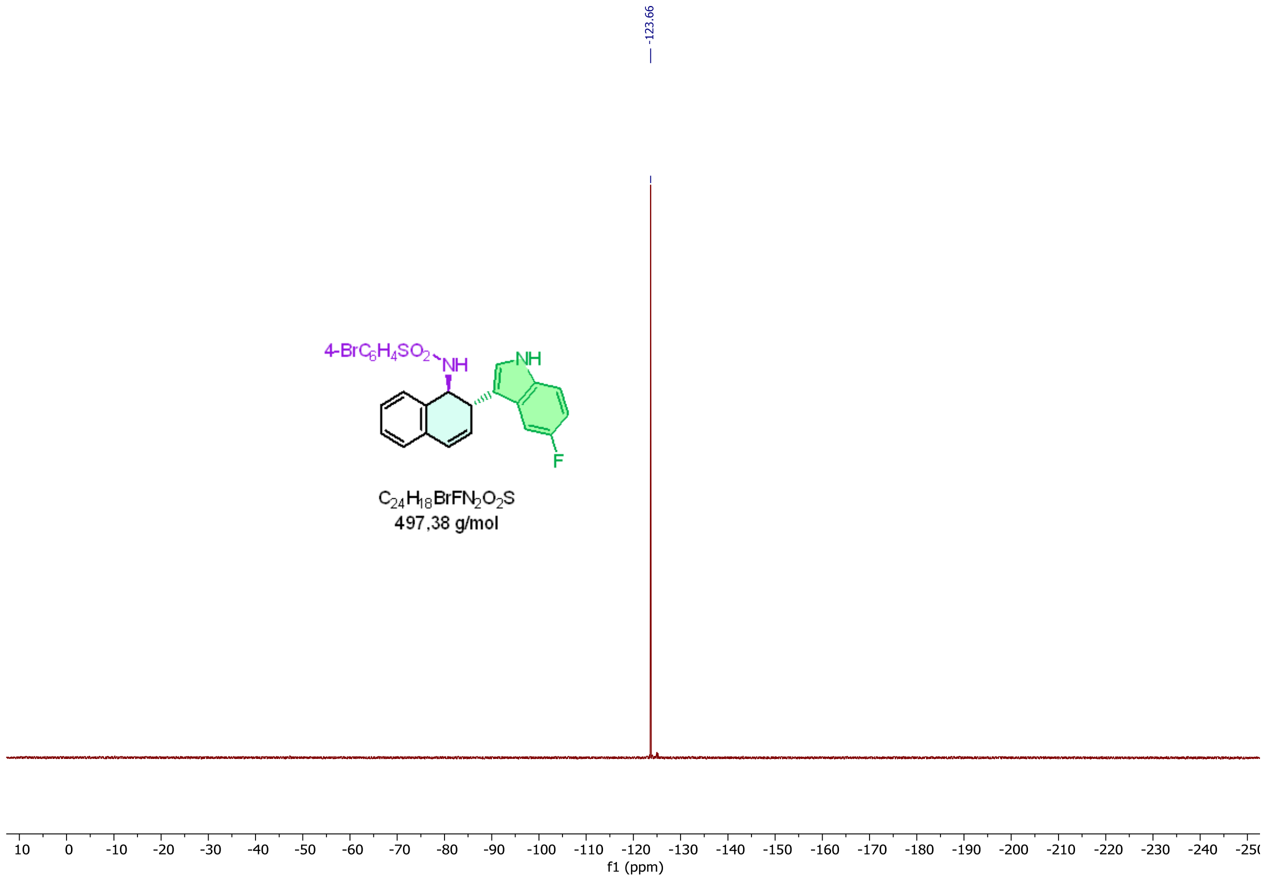
-123.66
4-BrC6H4SO2
NH
NH
F
C24H18BrFN2O2S
497,38 g/mol
10 0 -10 -20 -30 -40 -50 -60 -70 -80 -90 -100 -110 -120 -130 -140 -150 -160 -170 -180 -190 -200 -210 -220 -230 -240
f1 (ppm)

^{1}H NMR (400 MHz, acetone-d$_6$) and ^{13}C NMR (101 MHz, acetone-d$_6$) Analysis of Compound **7f**

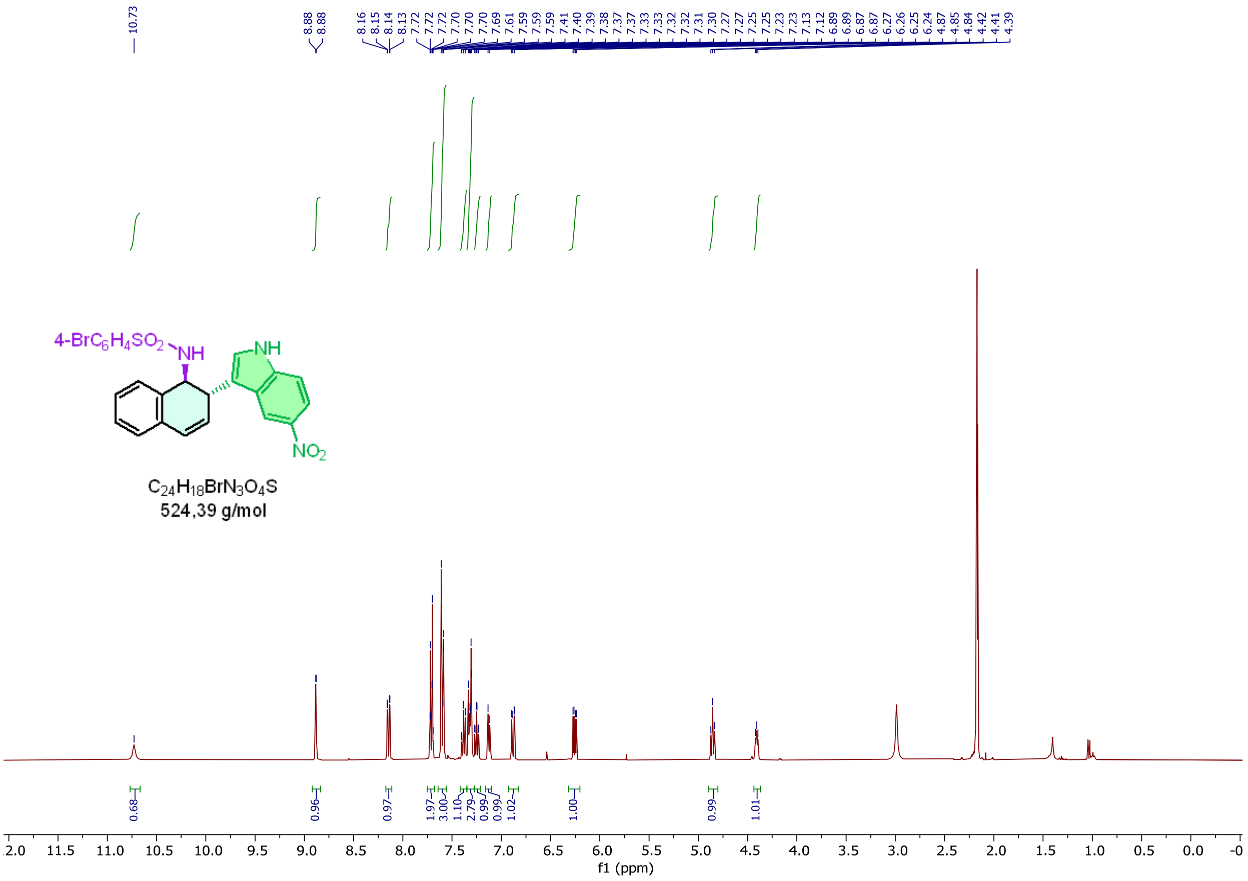

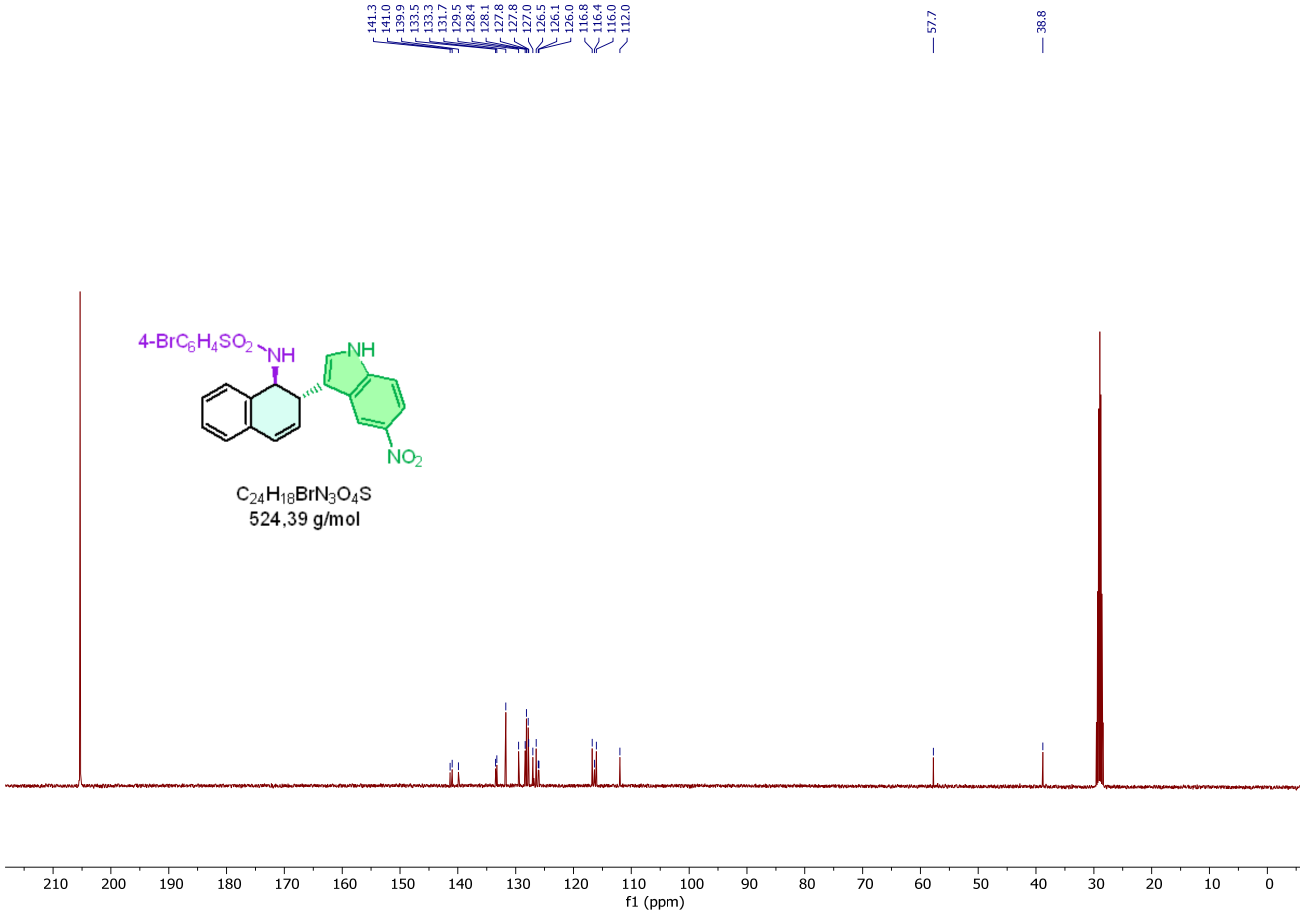

^{1}H NMR (400 MHz, $CDCl_3$) and ^{13}C NMR (101 MHz, $CDCl_3$) Analysis of Compound **7g**

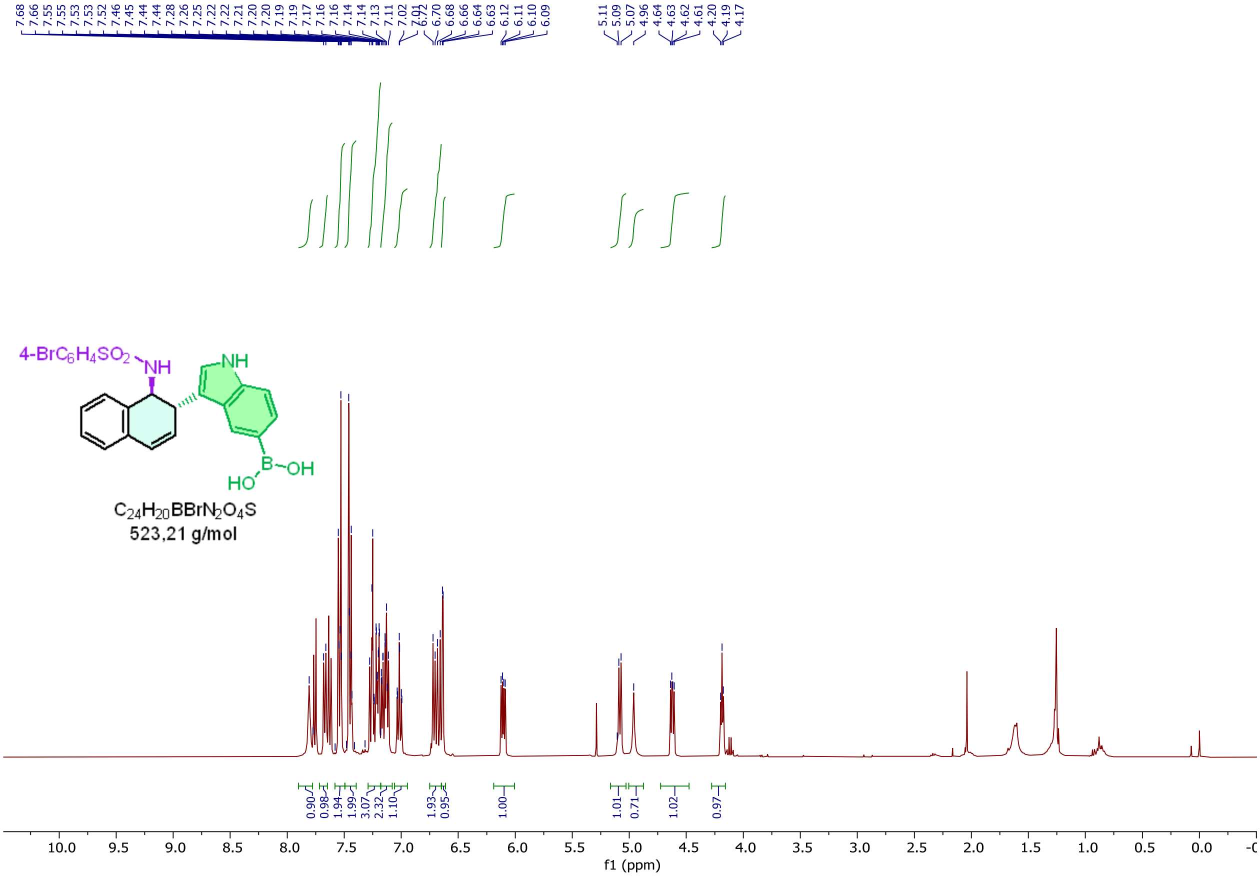

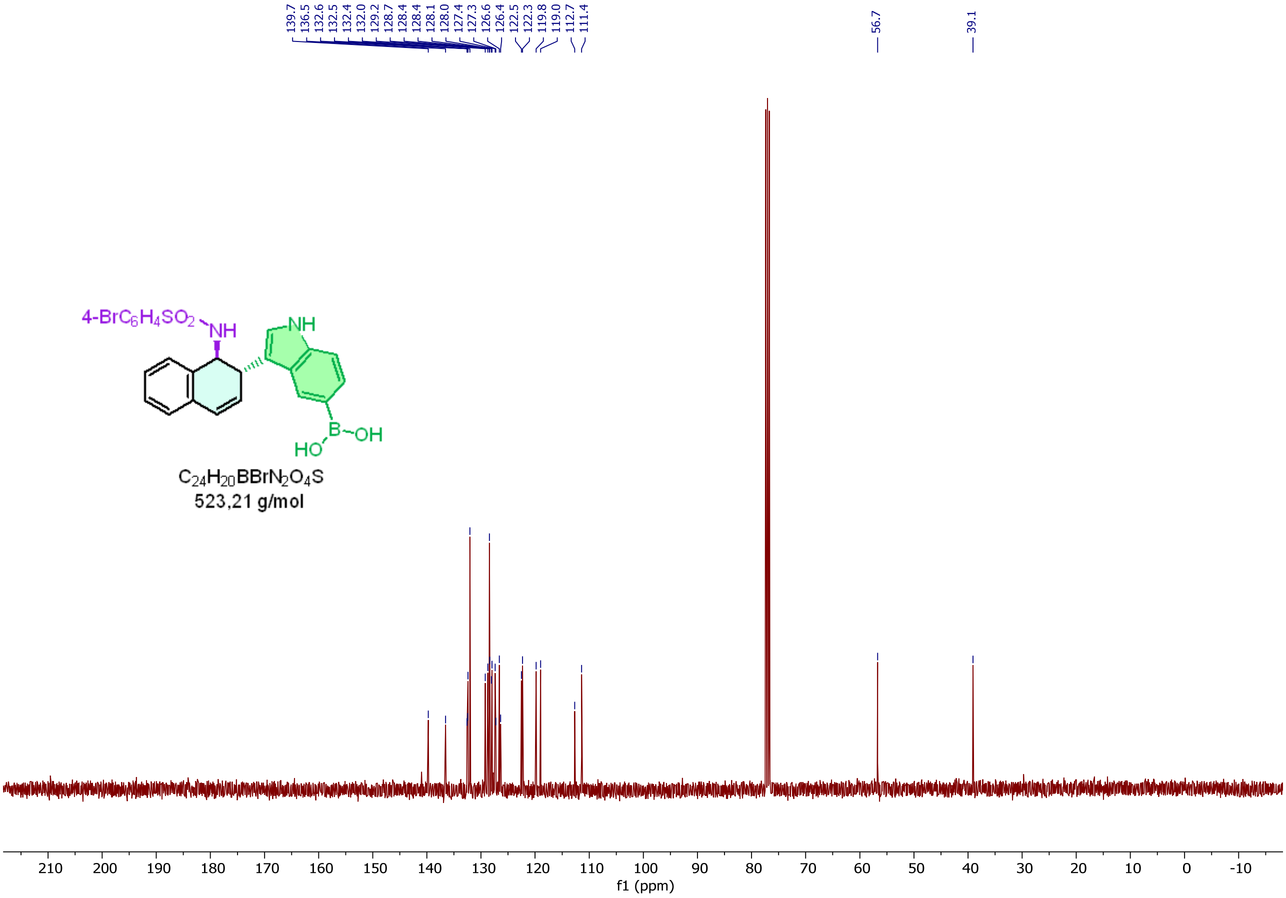

^{1}H NMR (400 MHz, $CDCl_3$) and ^{13}C NMR (101 MHz, $CDCl_3$) Analysis of Compound **7h**

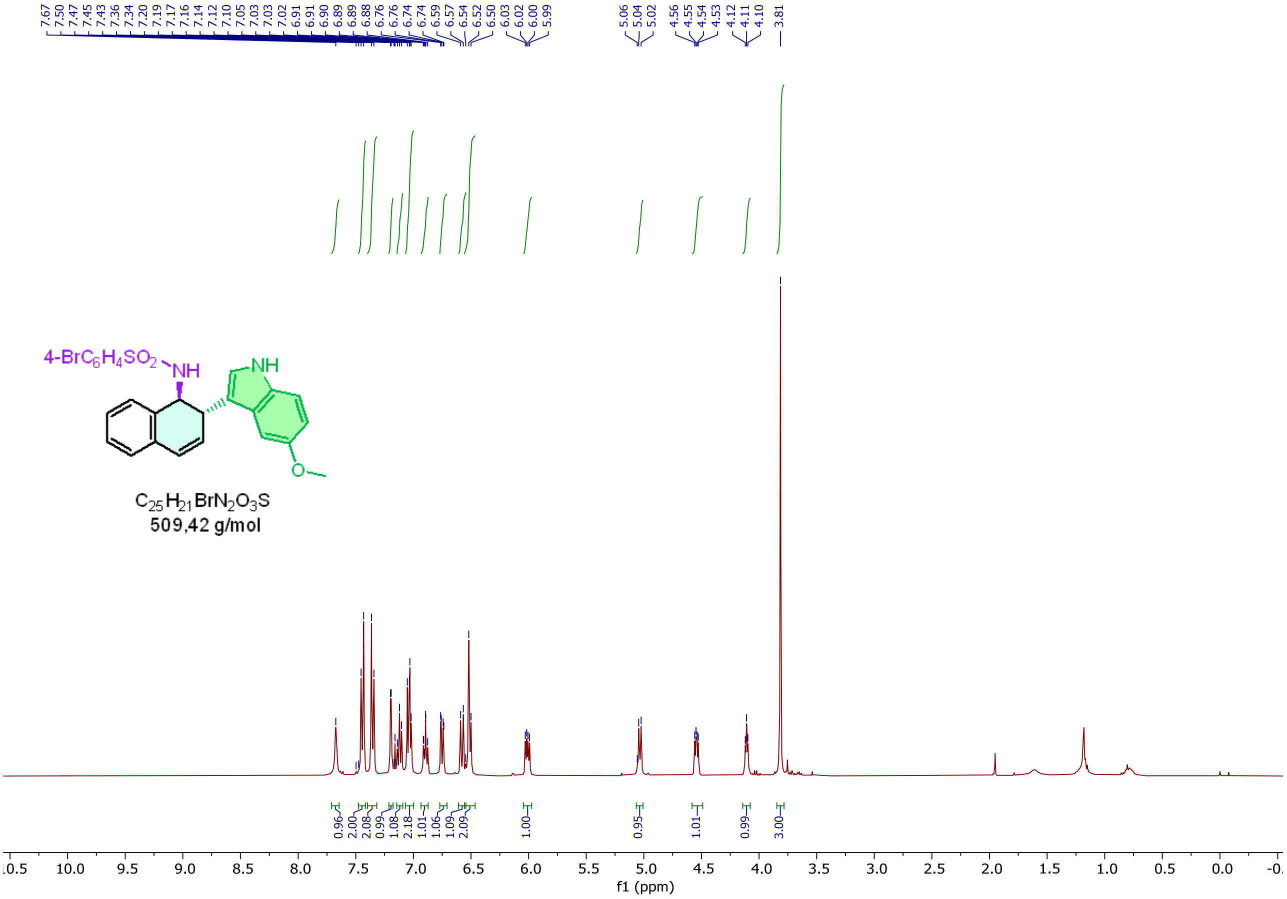

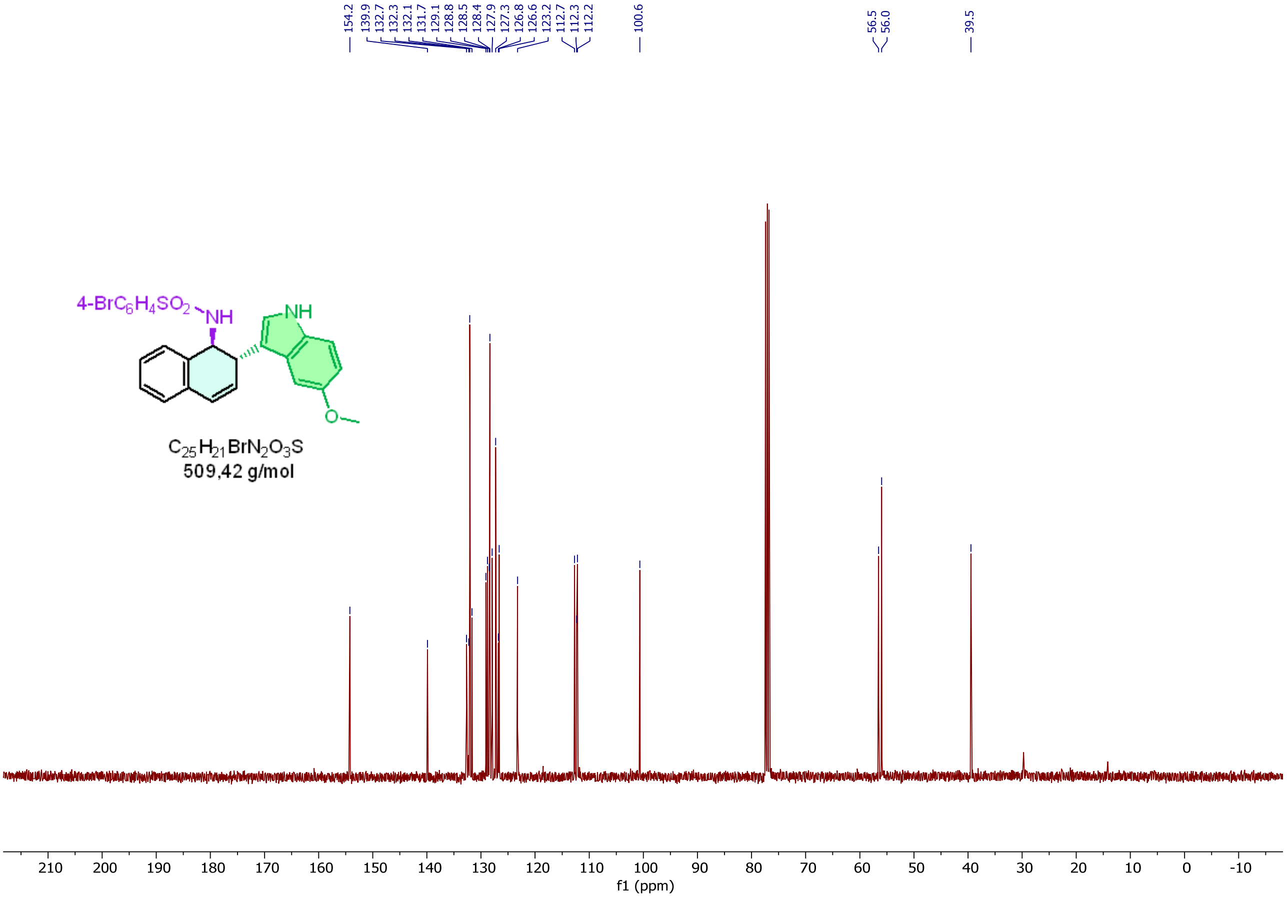

^{1}H NMR (400 MHz, $CDCl_3$) and ^{13}C NMR (101 MHz, $CDCl_3$) Analysis of Compound **7i**

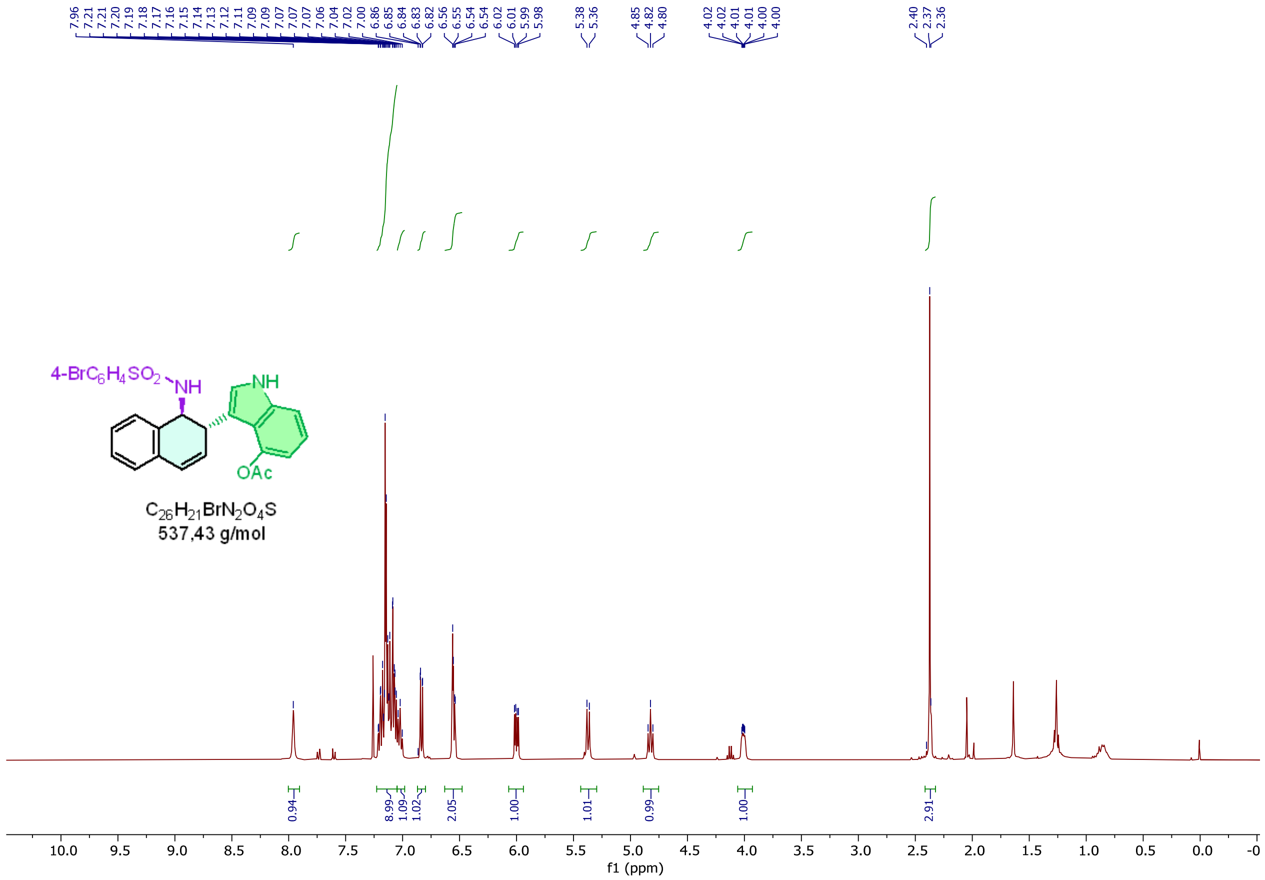

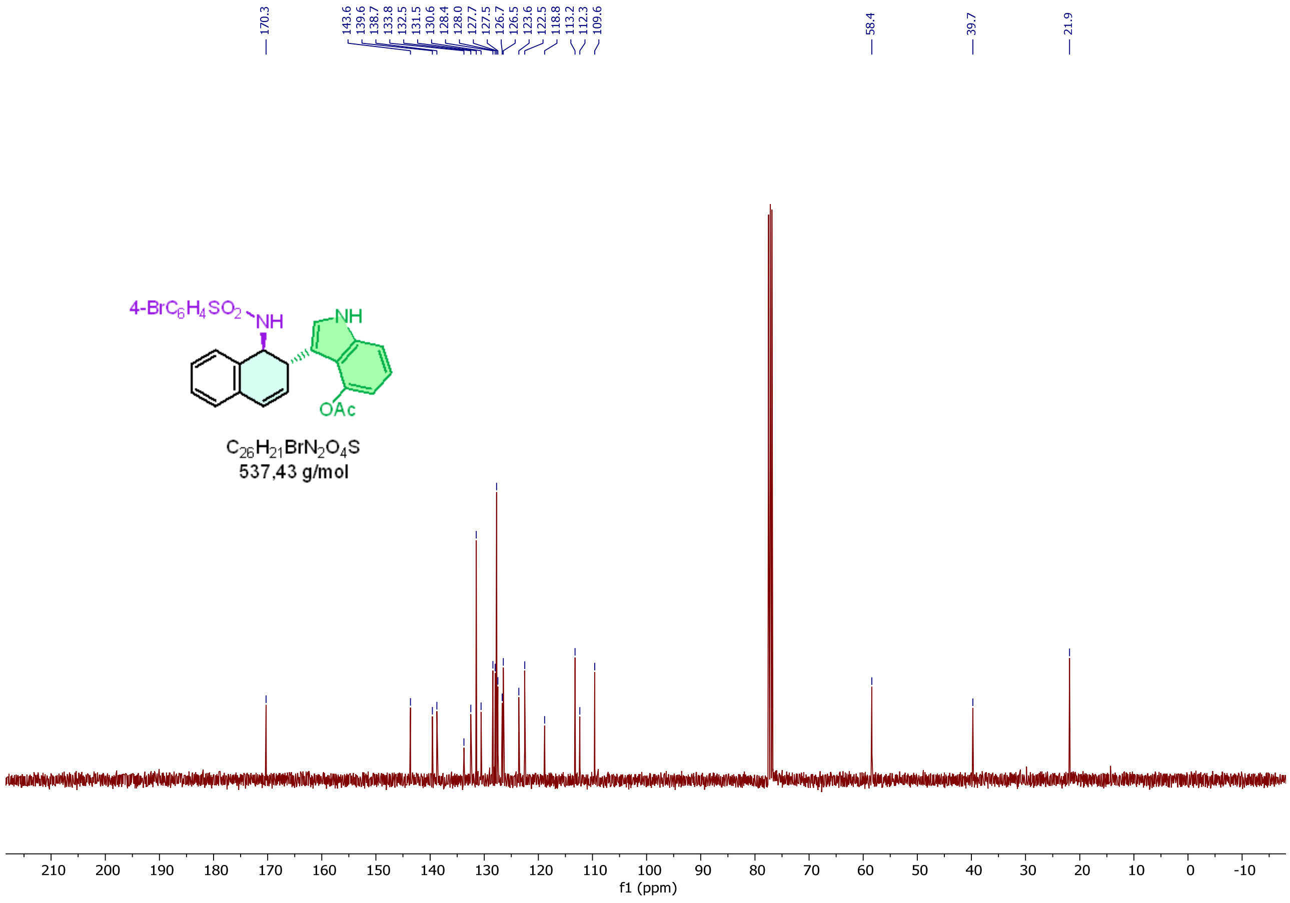

^{1}H NMR (400 MHz, $CDCl_3$) and ^{13}C NMR (101 MHz, $CDCl_3$) Analysis of Compound **7j**

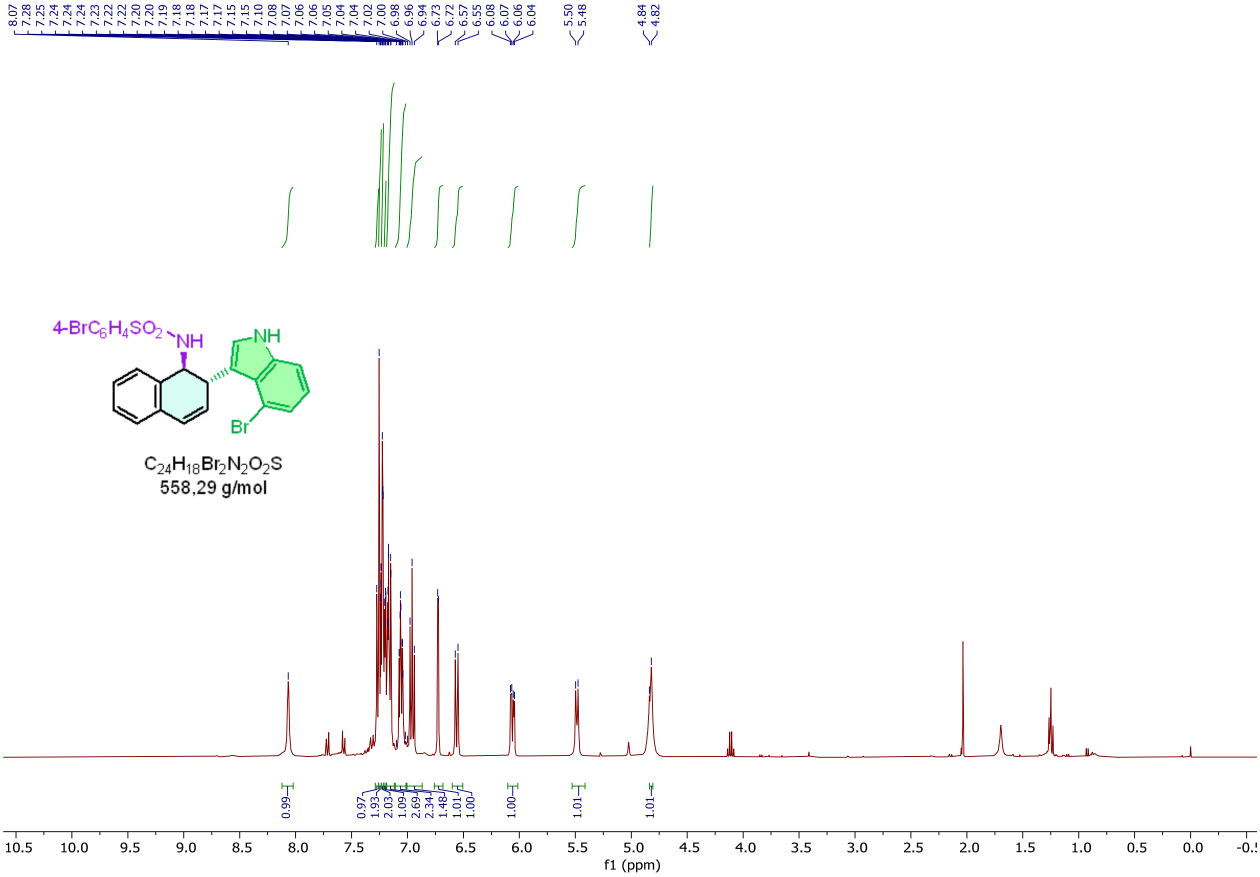

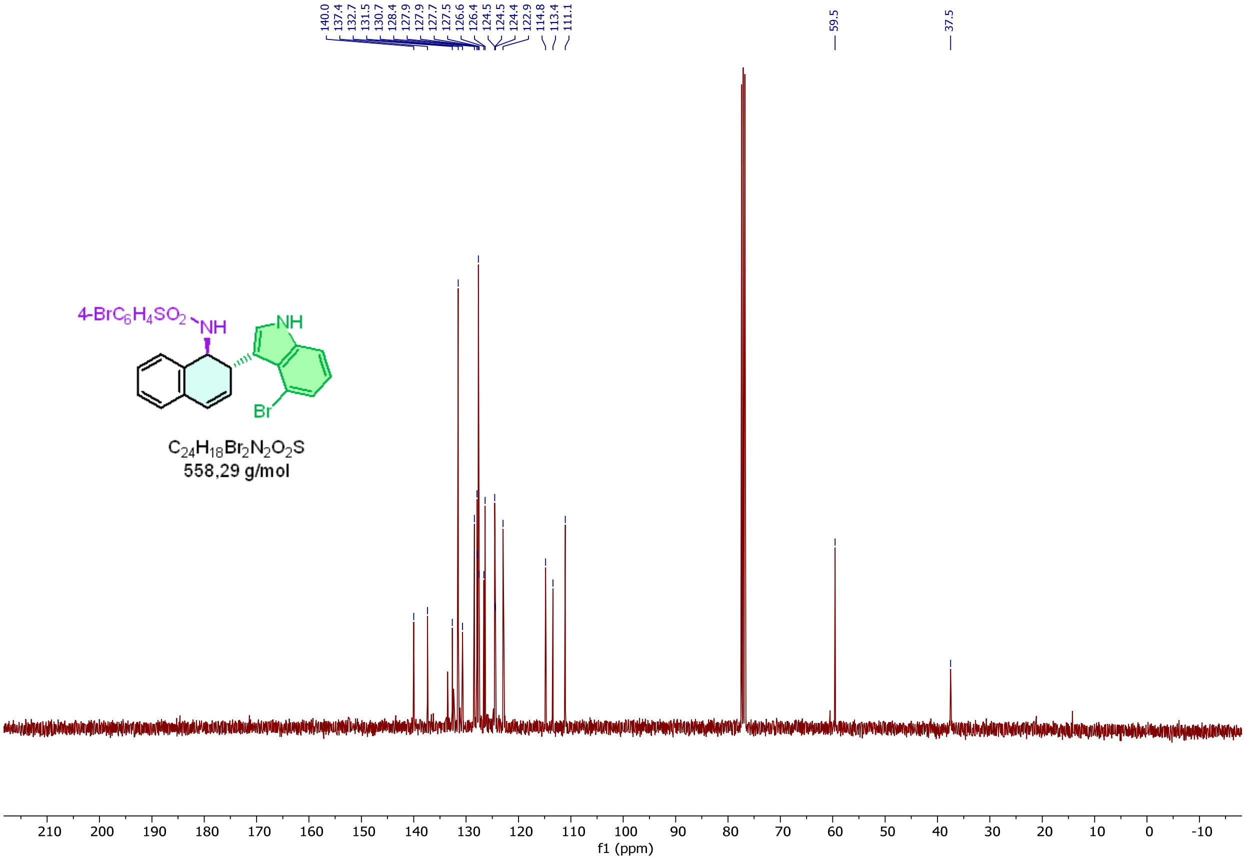

^{1}H NMR (400 MHz, $CDCl_3$) and ^{13}C NMR (101 MHz, $CDCl_3$) Analysis of Compound **7k**

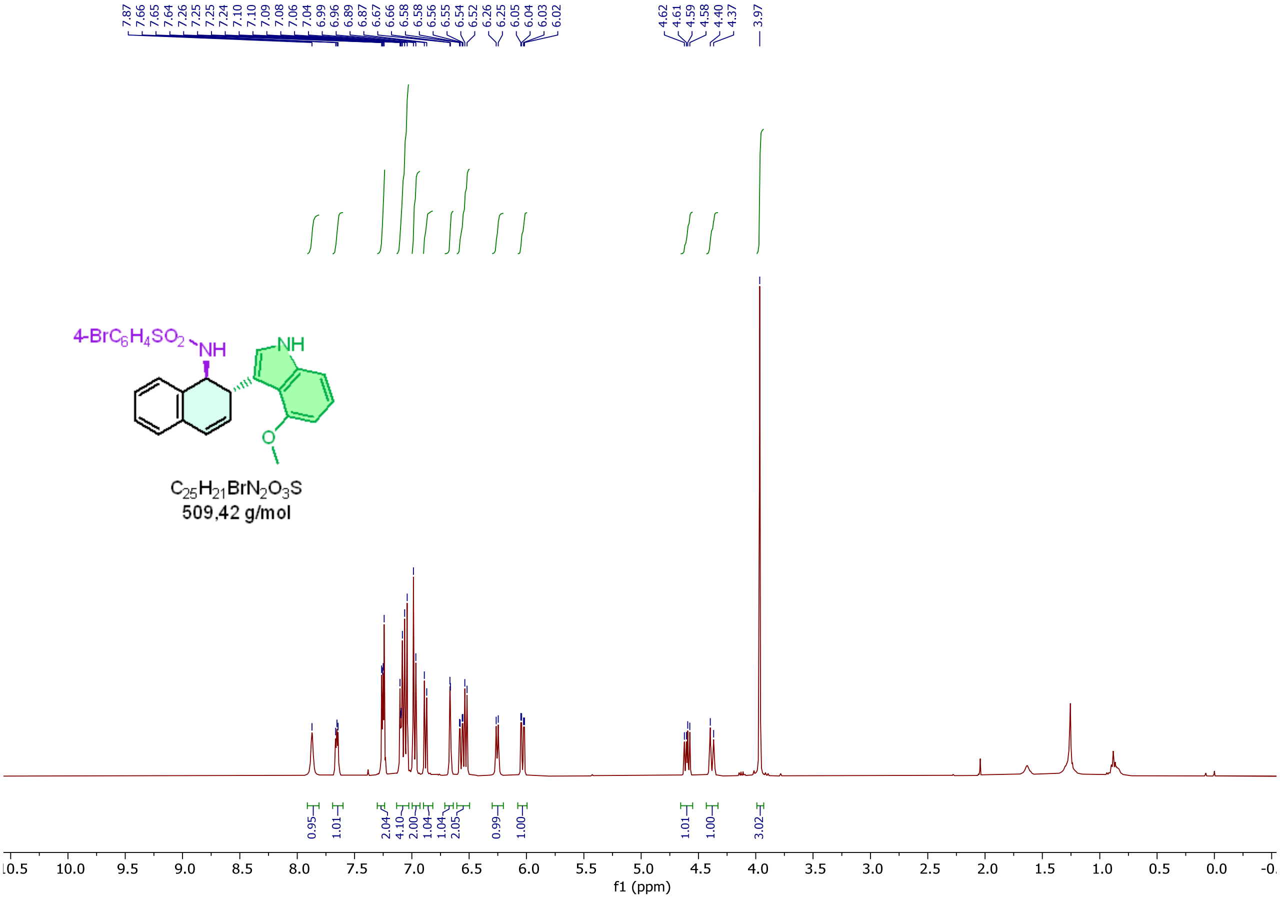

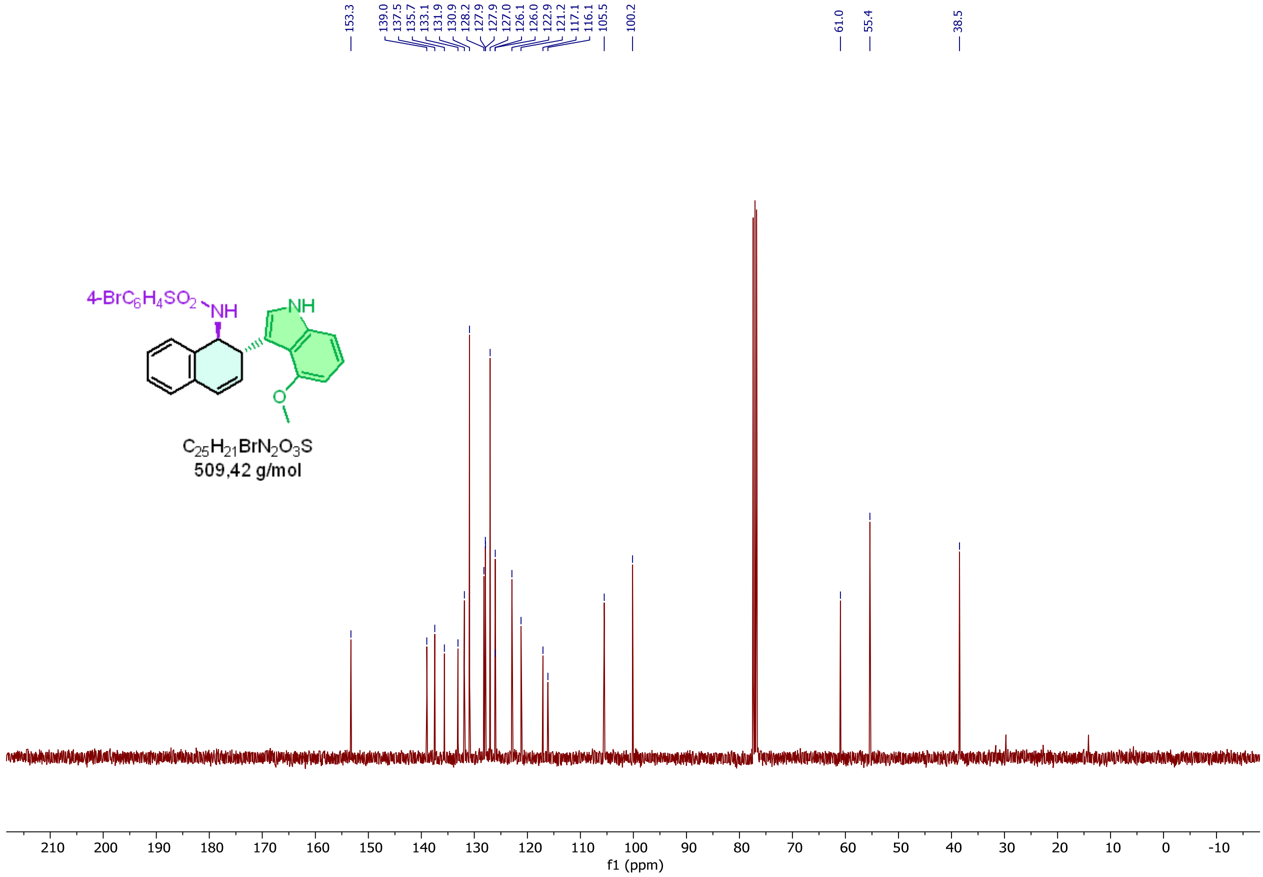

^1H NMR (400 MHz, $CDCl_3$) and ^{13}C NMR (101 MHz, $CDCl_3$) Analysis of Compound **7l**

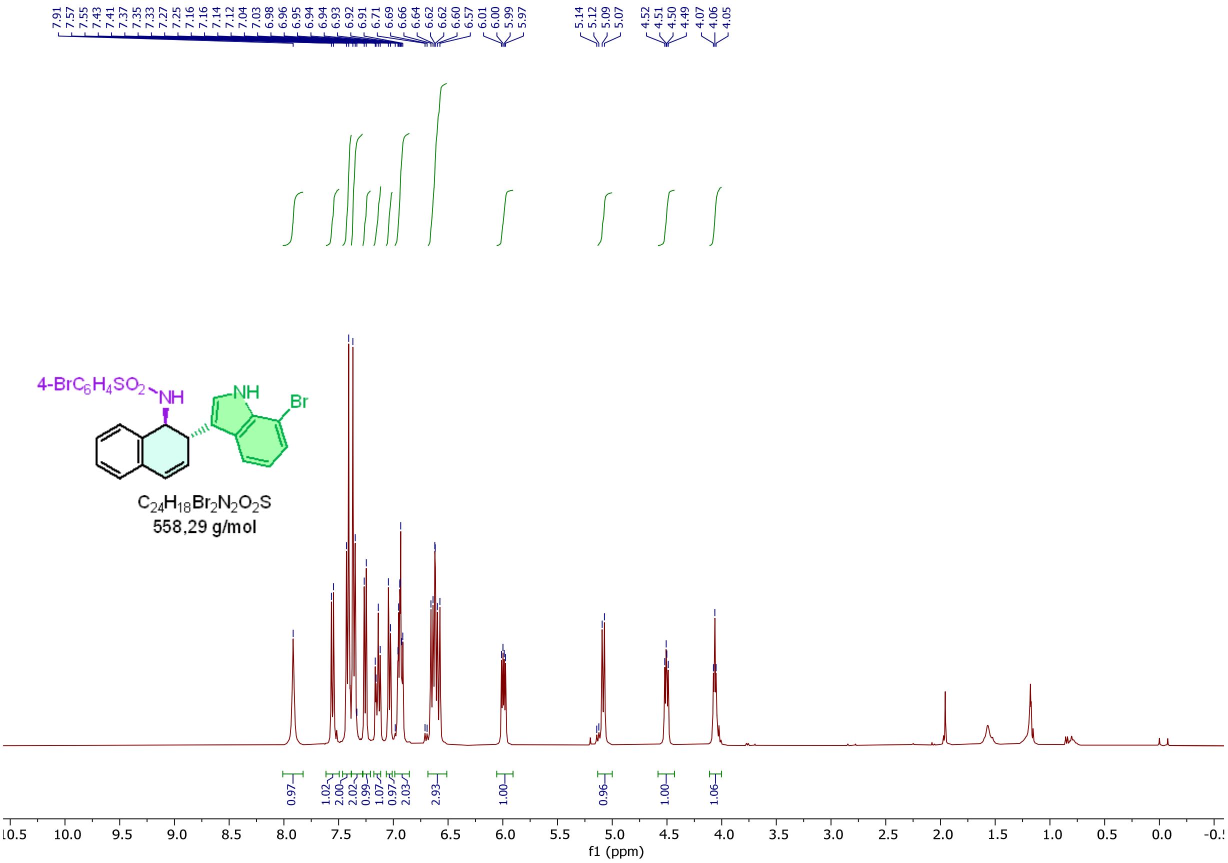

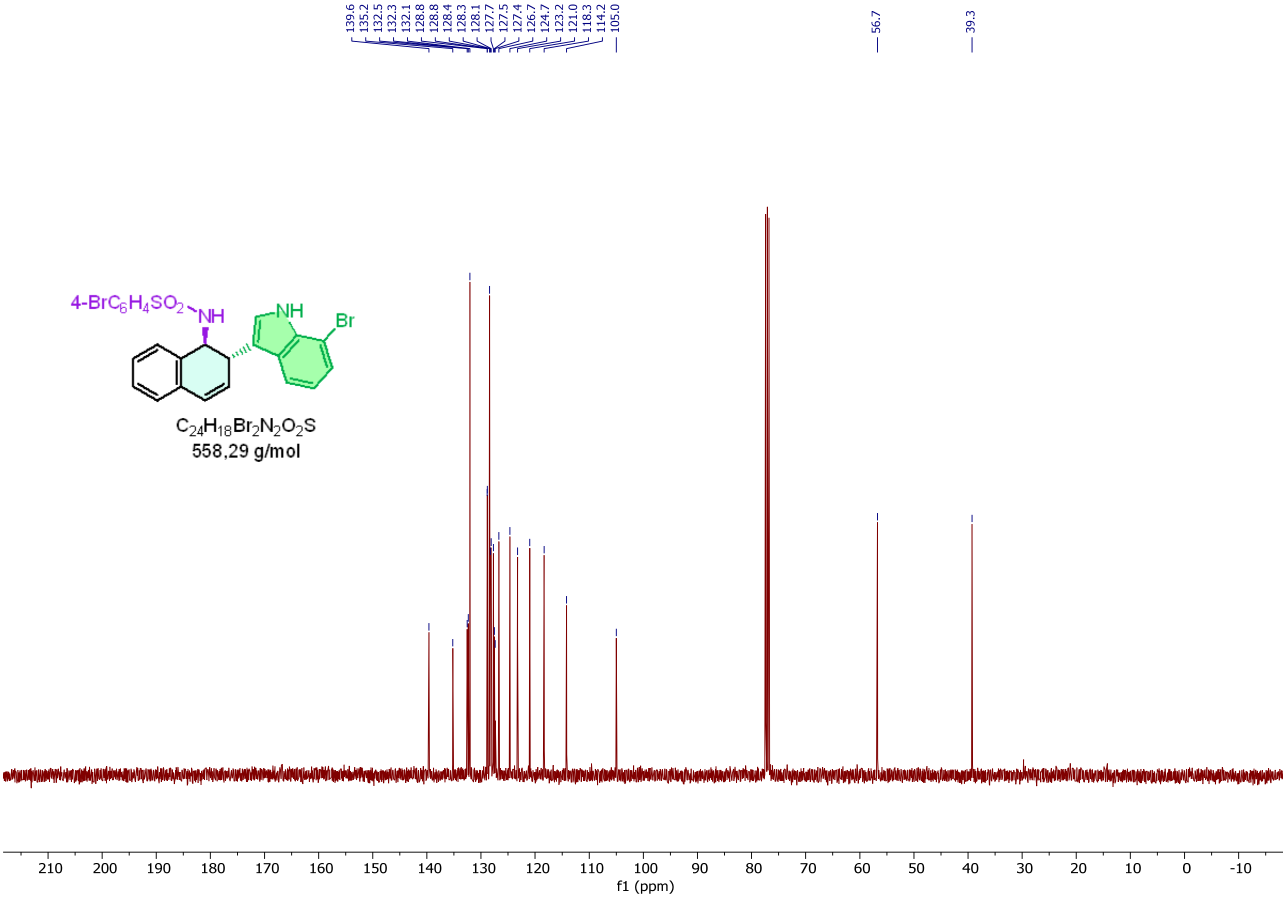

^{1}H NMR (400 MHz, $CDCl_3$) and ^{13}C NMR (101 MHz, $CDCl_3$) Analysis of Compound **7m**

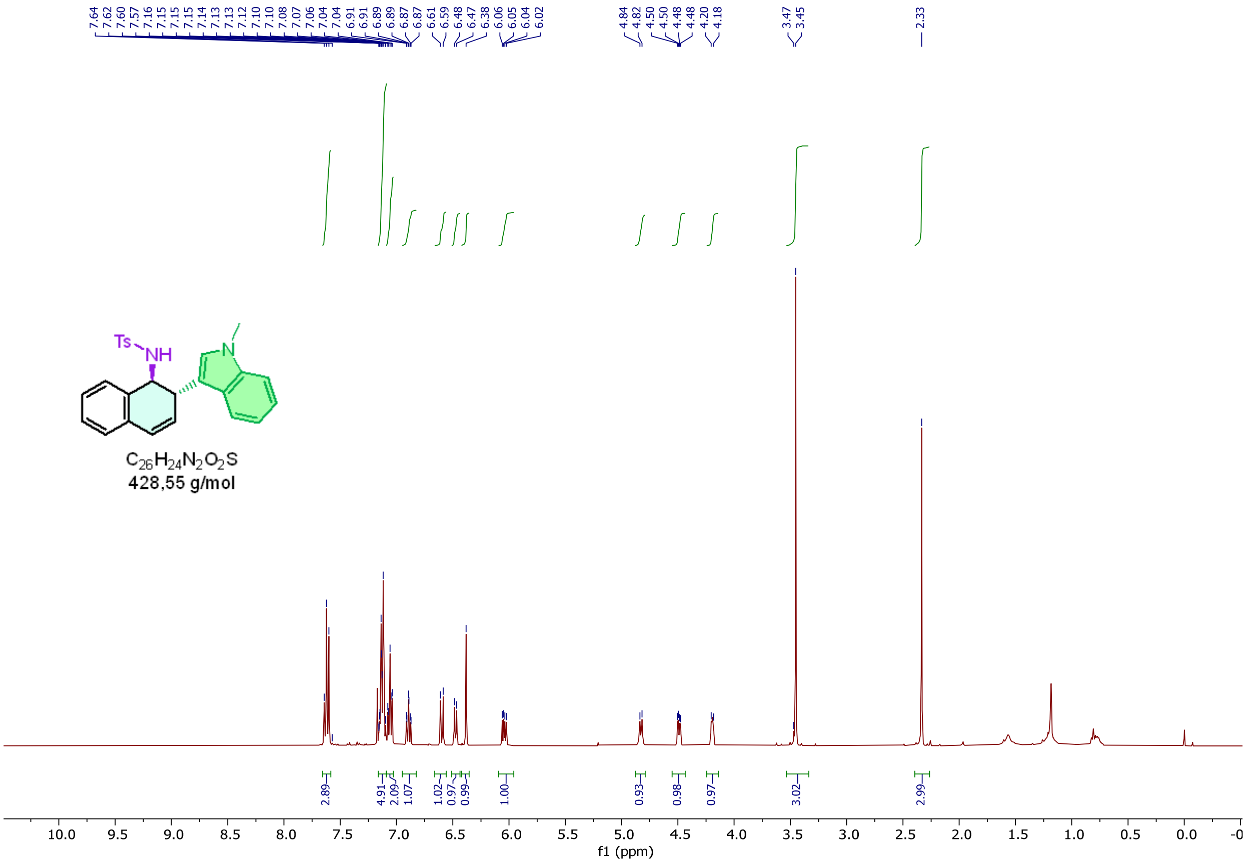

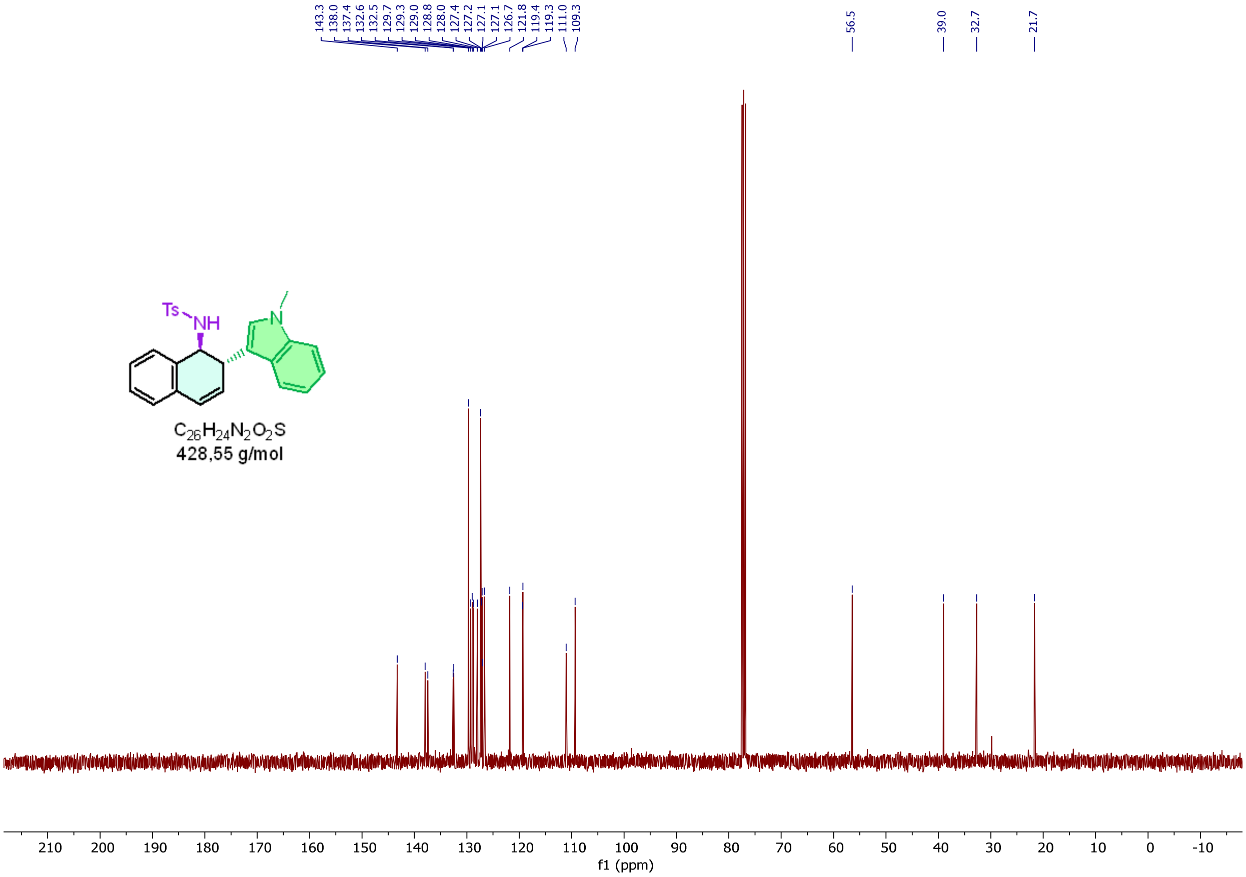

^{1}H NMR (400 MHz, CDCl$_3$), ^{13}C NMR (101 MHz, CDCl$_3$) ^{19}F NMR (377 MHz, CDCl$_3$) Analysis of Compound **7n**

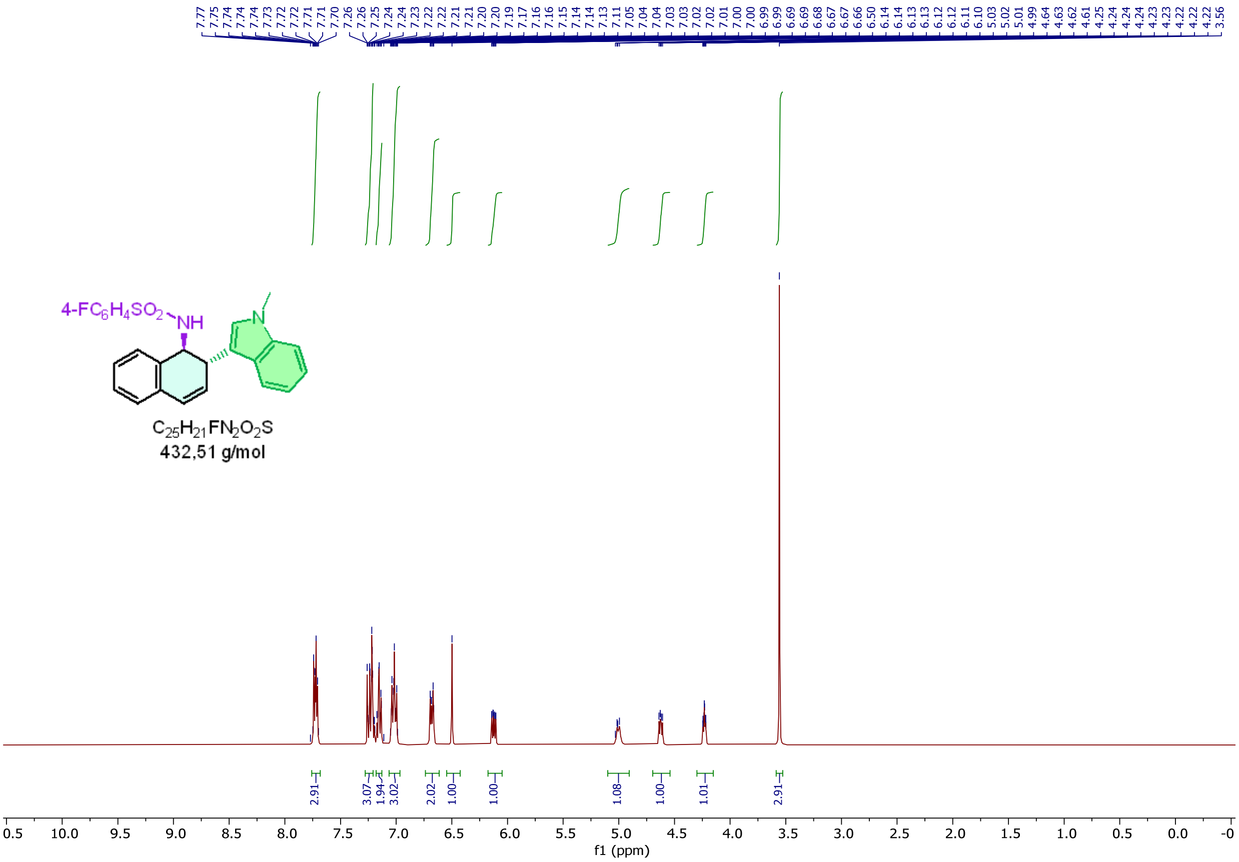

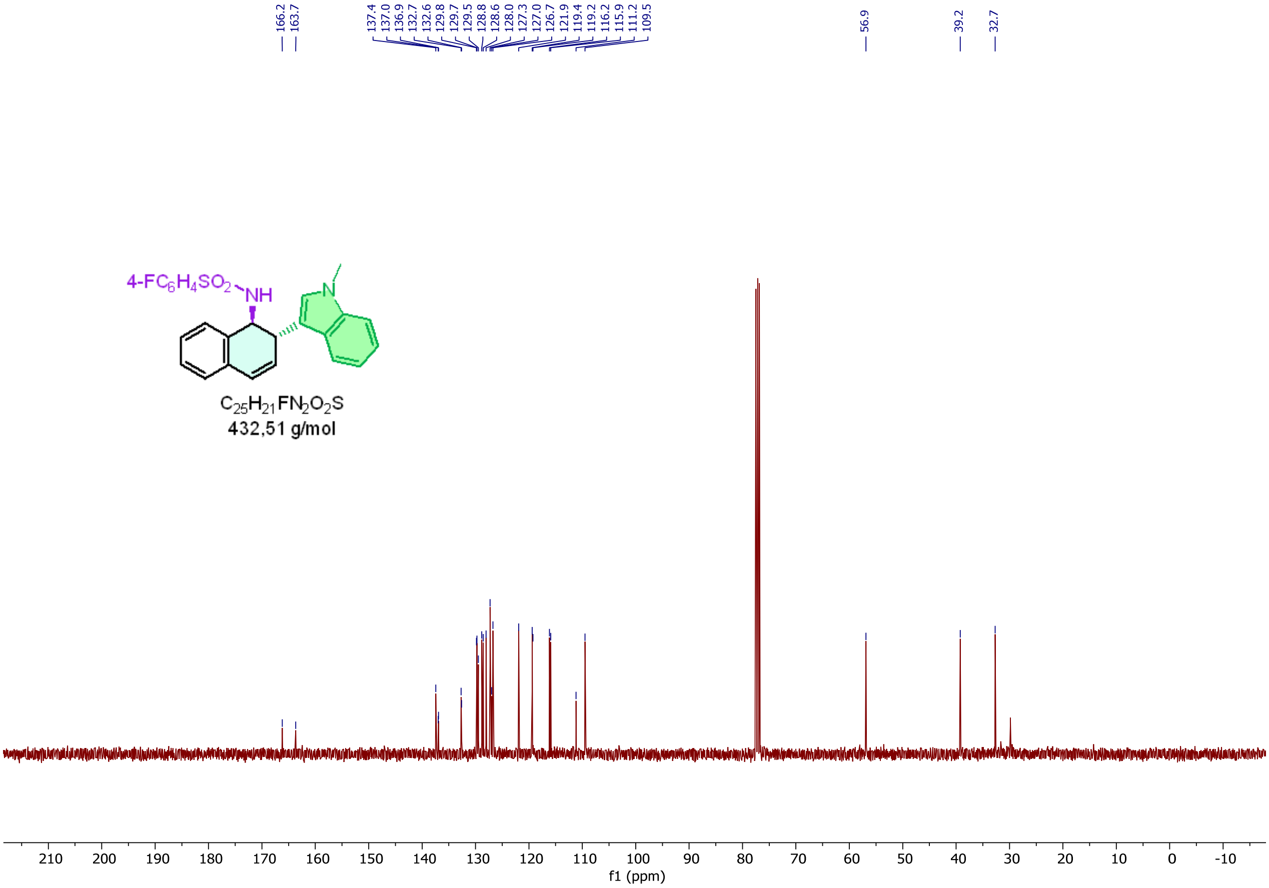

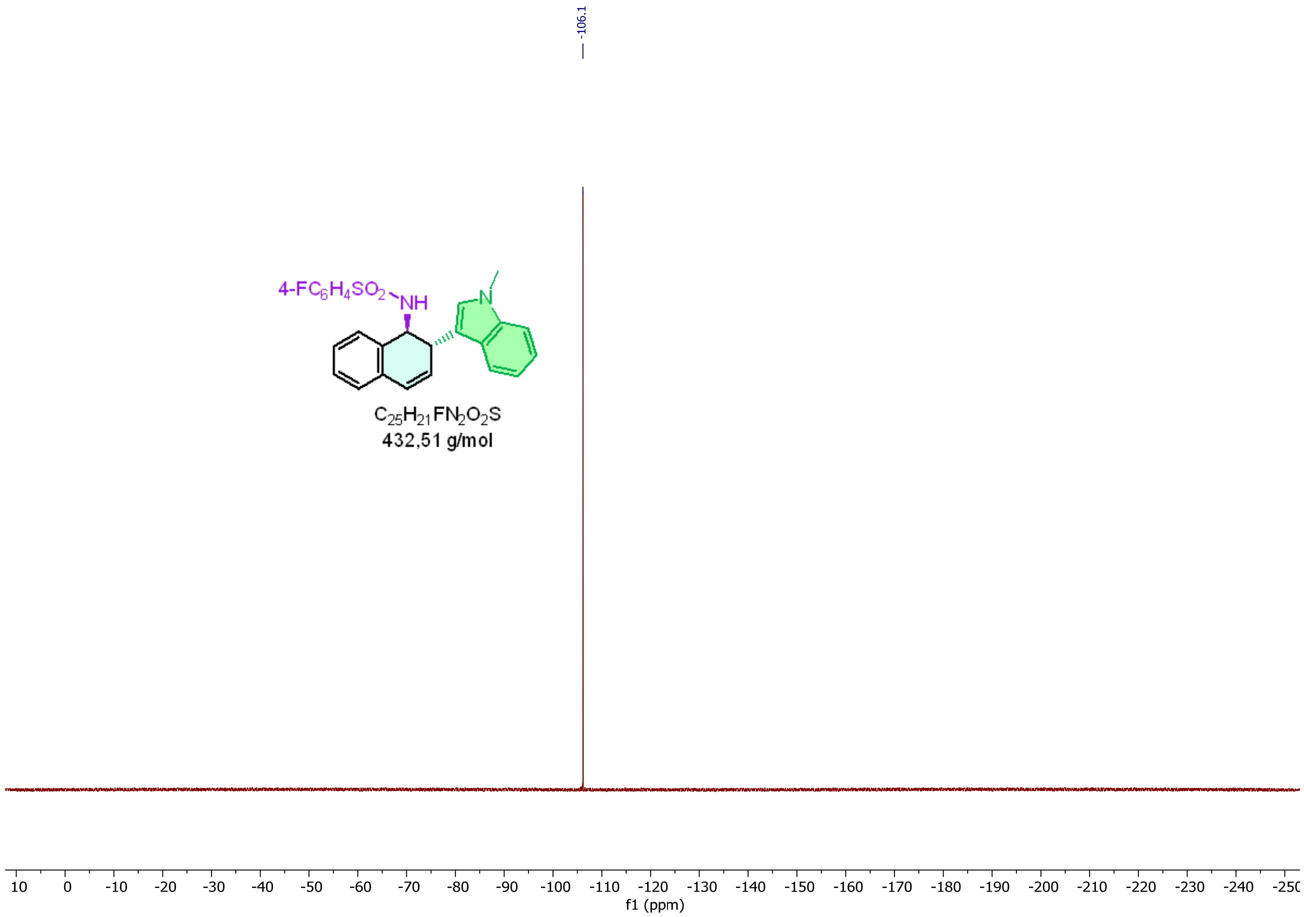
-106.1
4-FC6H4SO2
NH
N
C25H21FN2O2S
432.51 g/mol
10 0 -10 -20 -30 -40 -50 -60 -70 -80 -90 -100 -110 -120 -130 -140 -150 -160 -170 -180 -190 -200 -210 -220 -230 -240 -250
f1 (ppm)

^{1}H NMR (400 MHz, $CDCl_3$) and ^{13}C NMR (101 MHz, $CDCl_3$) Analysis of Compound **7o**

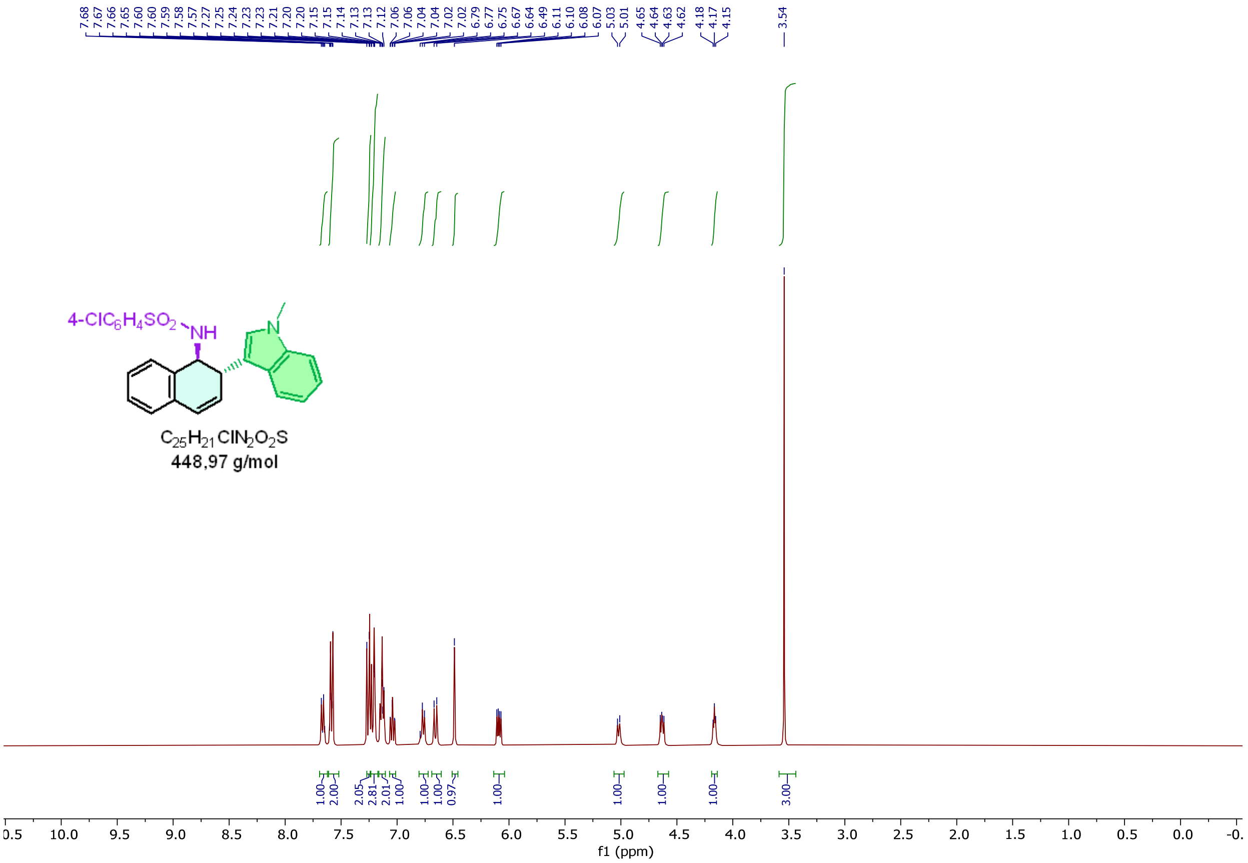

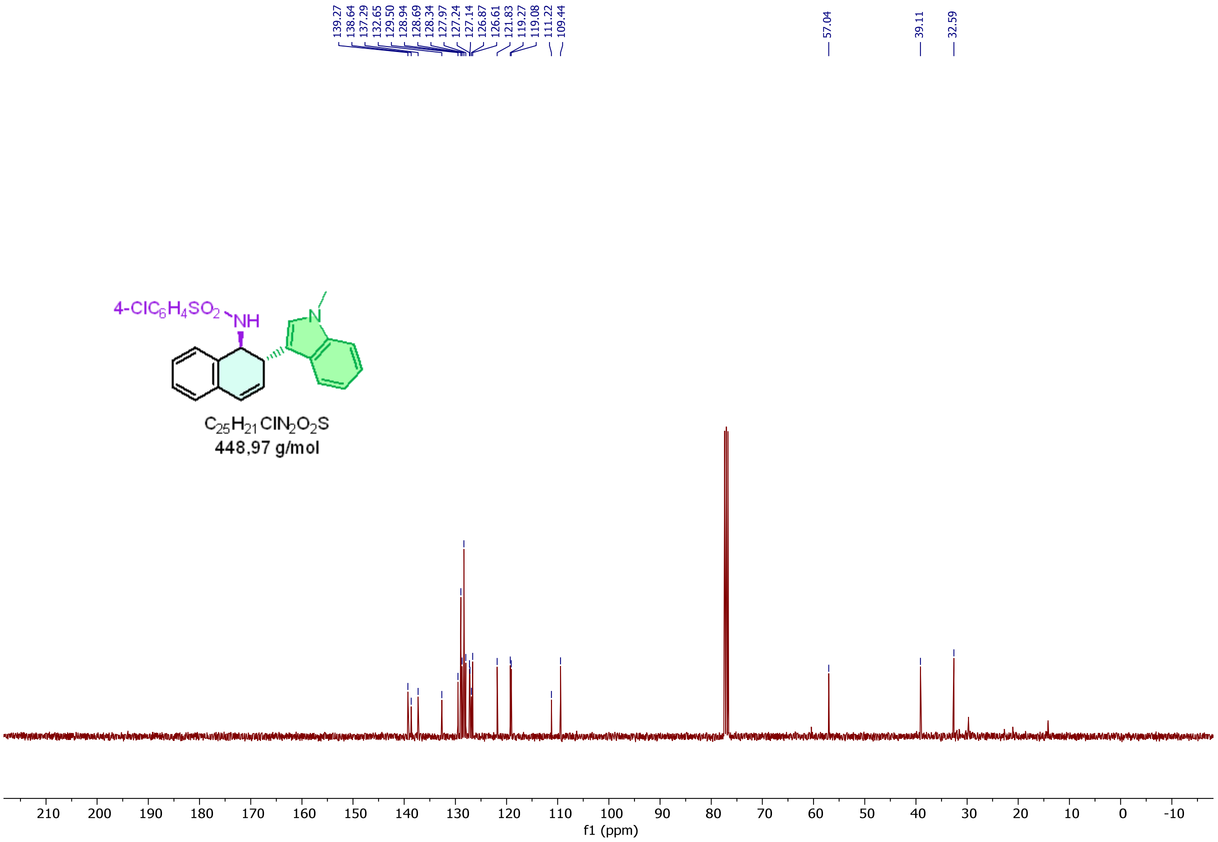

^{1}H NMR (400 MHz, $CDCl_3$) and ^{13}C NMR (101 MHz, $CDCl_3$) Analysis of Compound **7p**

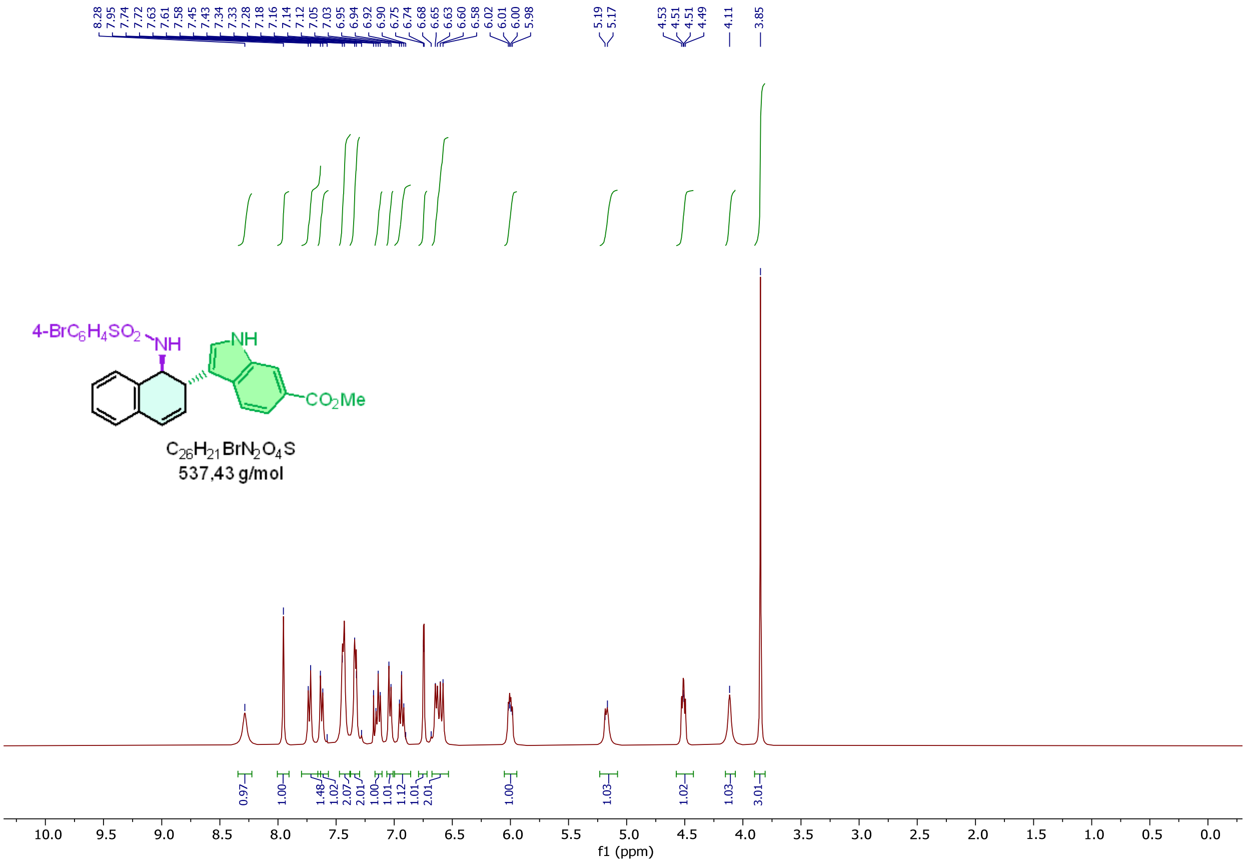

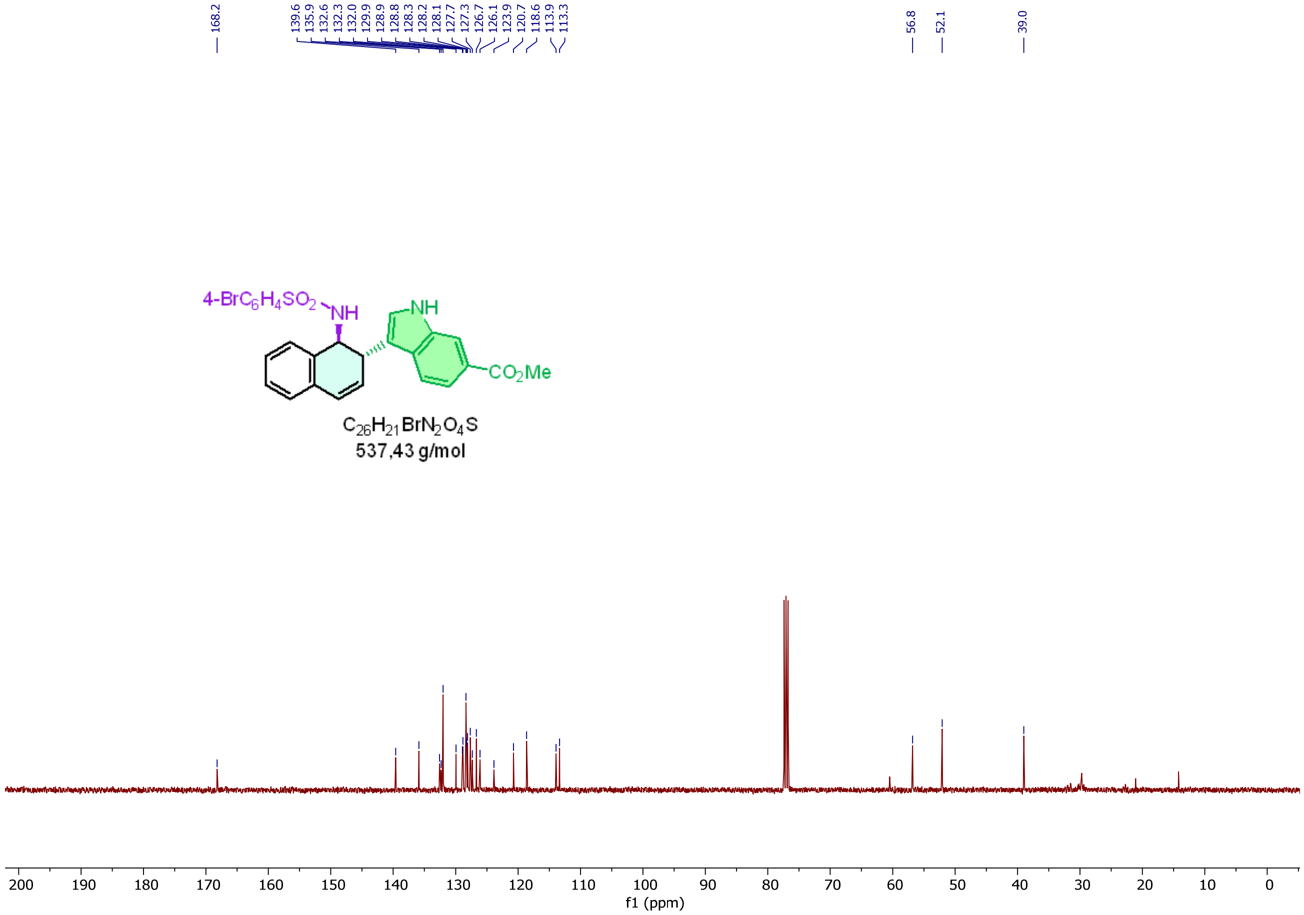

^1H NMR (400 MHz, $CDCl_3$) and ^{13}C NMR (101 MHz, $CDCl_3$) Analysis of Compound **7q**

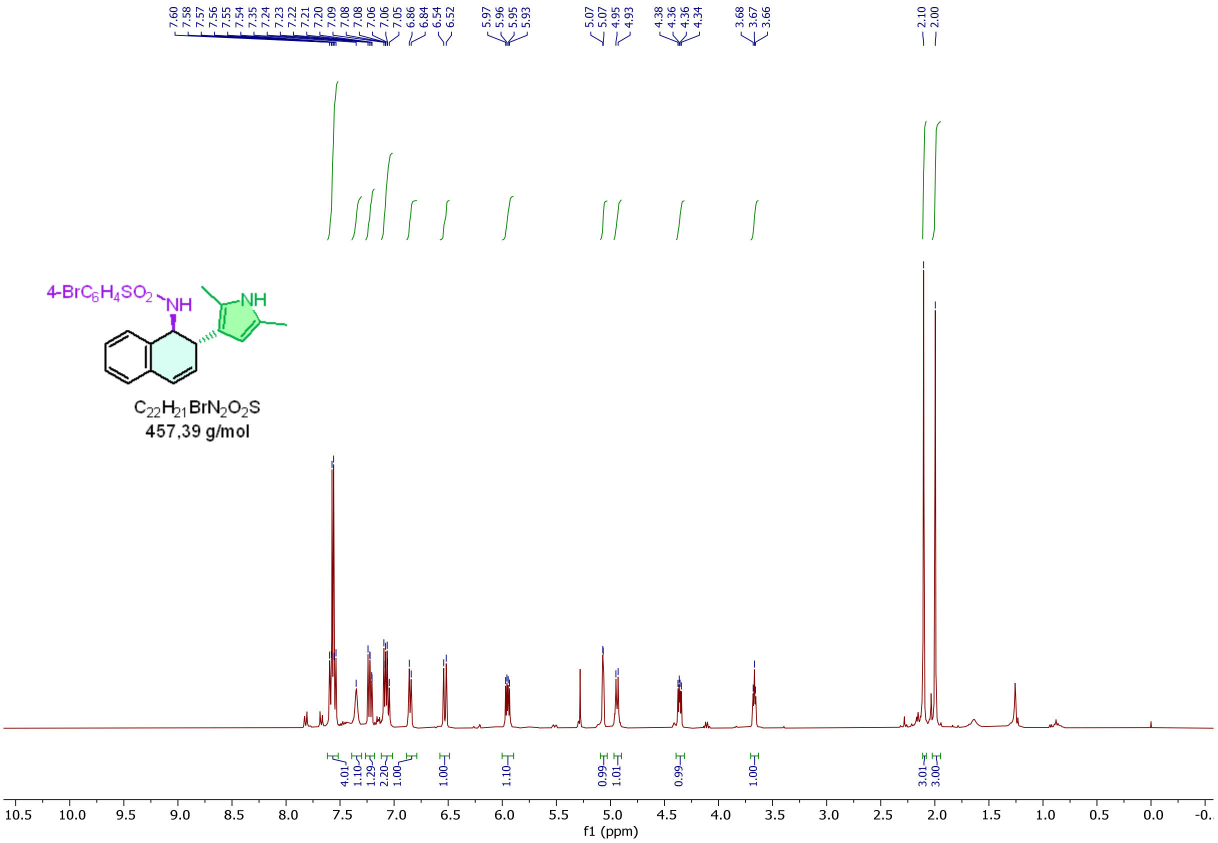

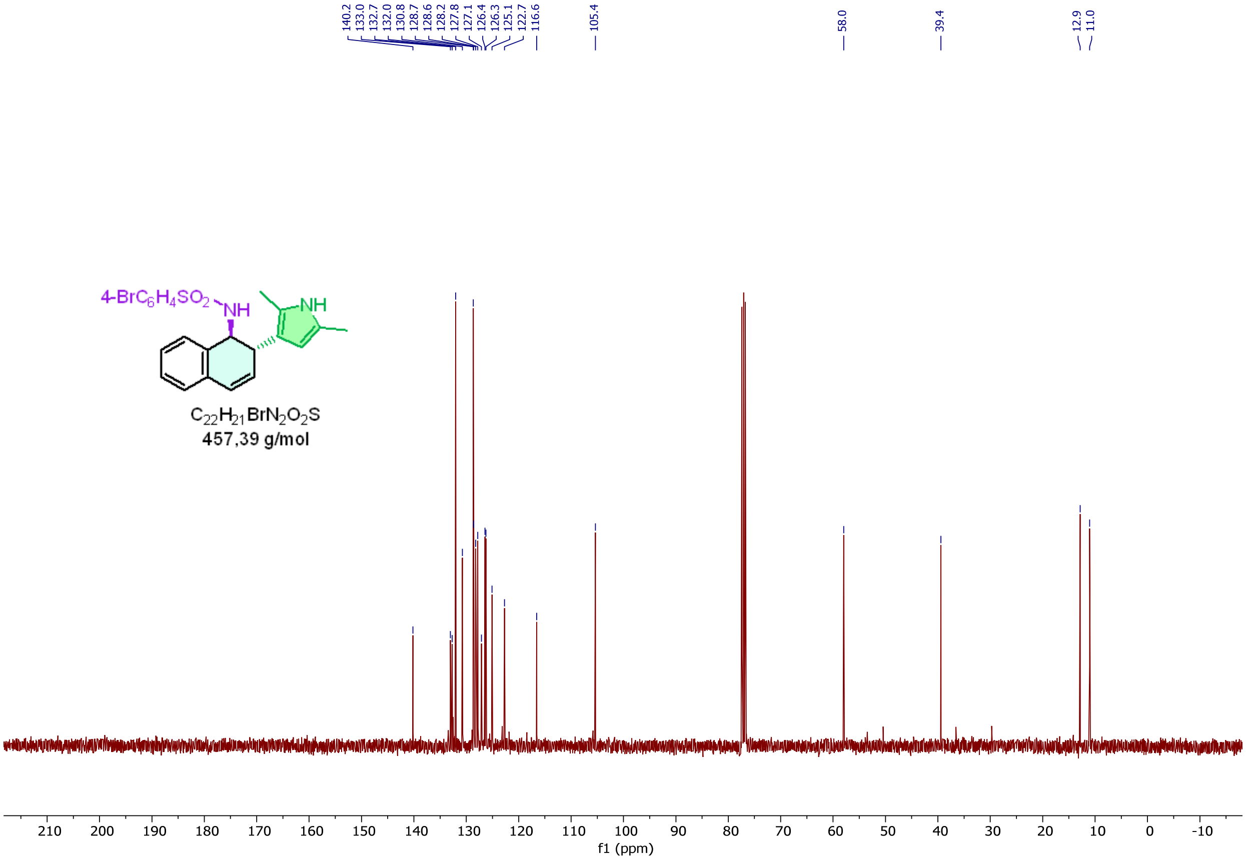

^{1}H NMR (400 MHz, $CDCl_3$) and ^{13}C NMR (101 MHz, $CDCl_3$) Analysis of Compound **7r**

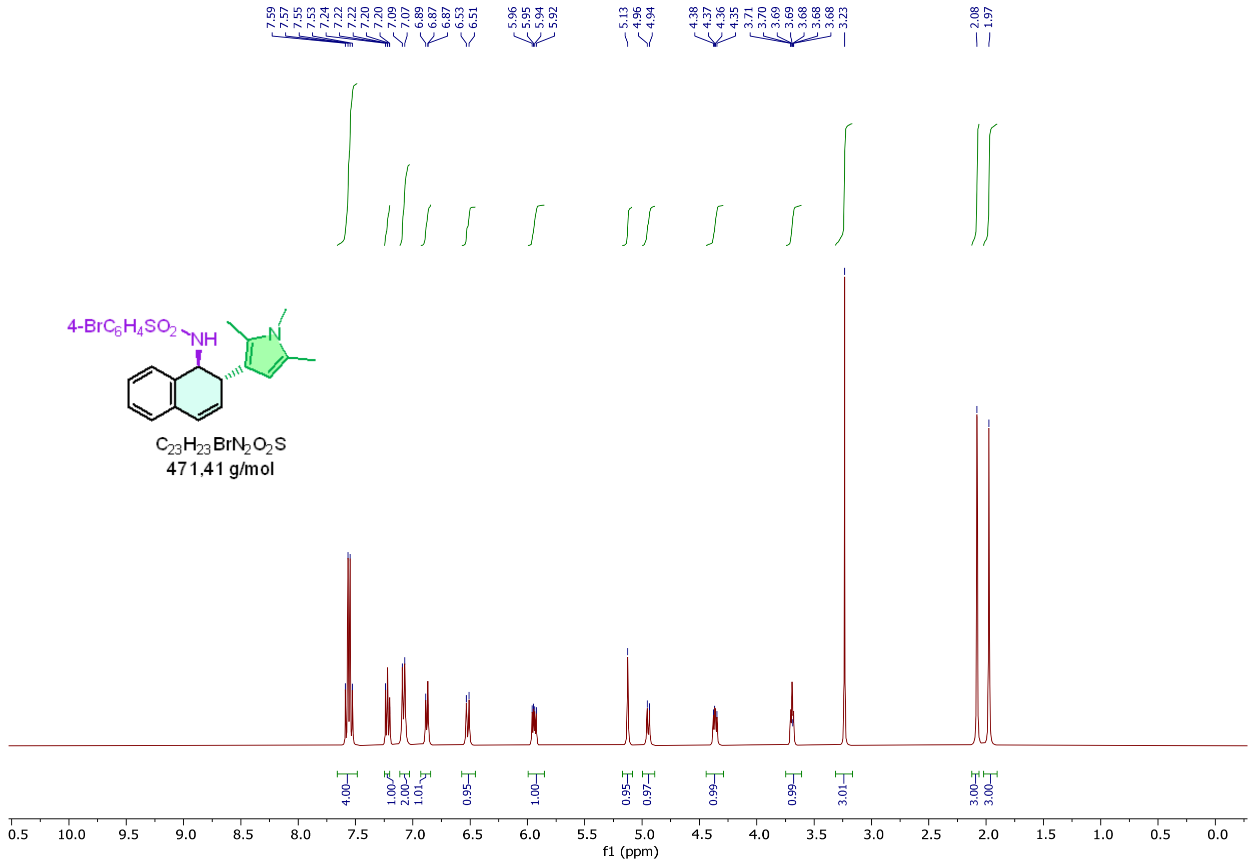

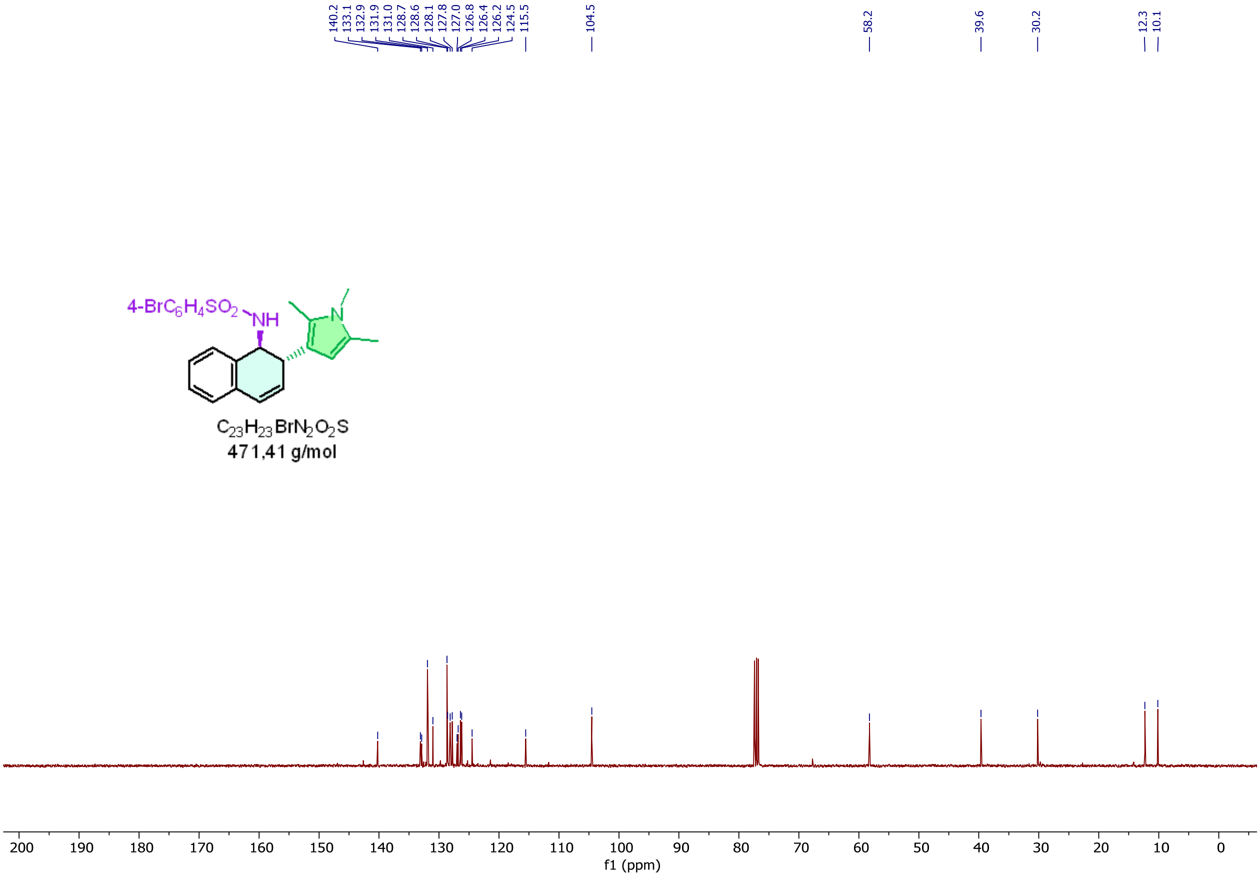

^{1}H NMR (400 MHz, $CDCl_3$) and ^{13}C NMR (101 MHz, $CDCl_3$) Analysis of Compound **7s**

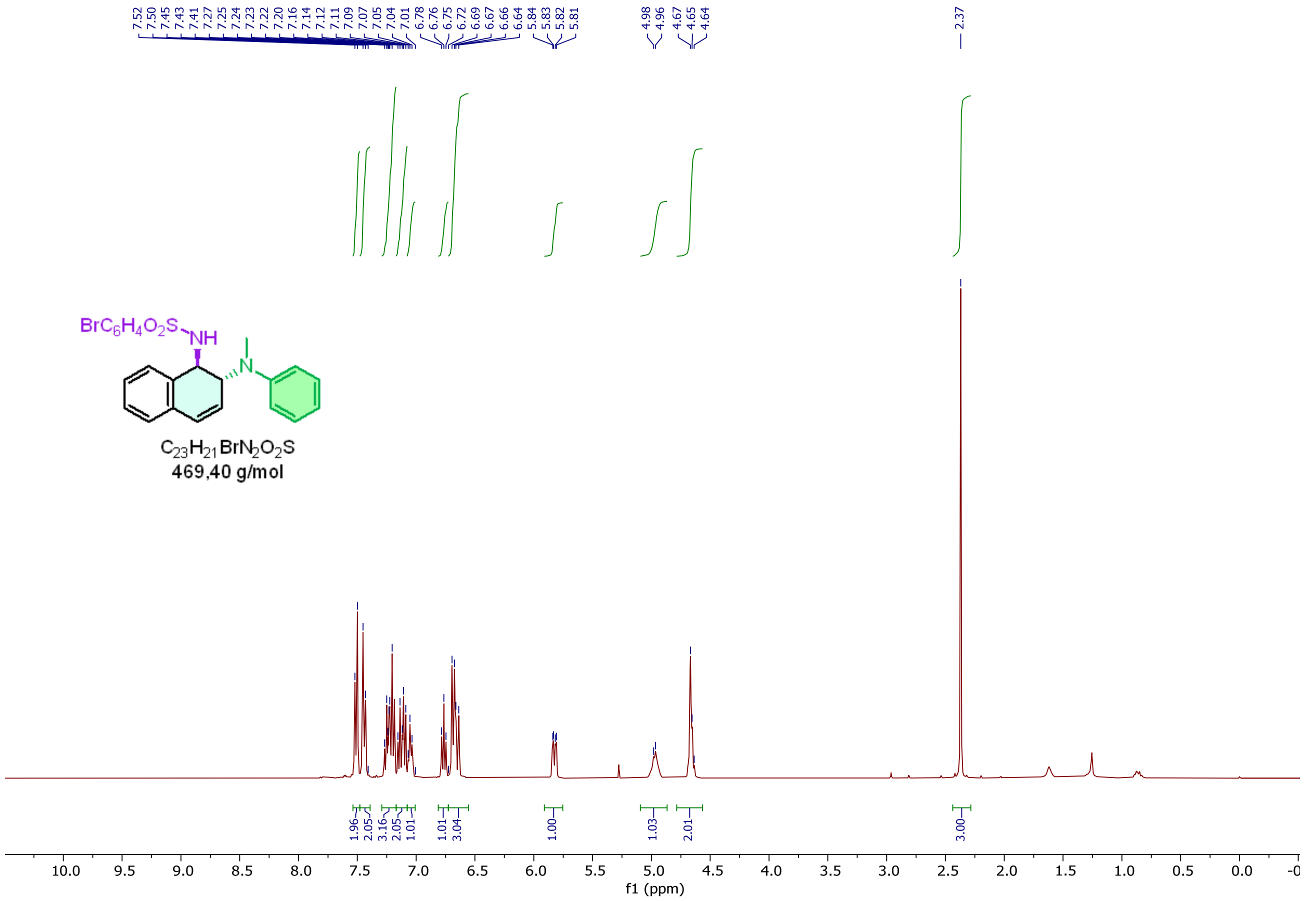

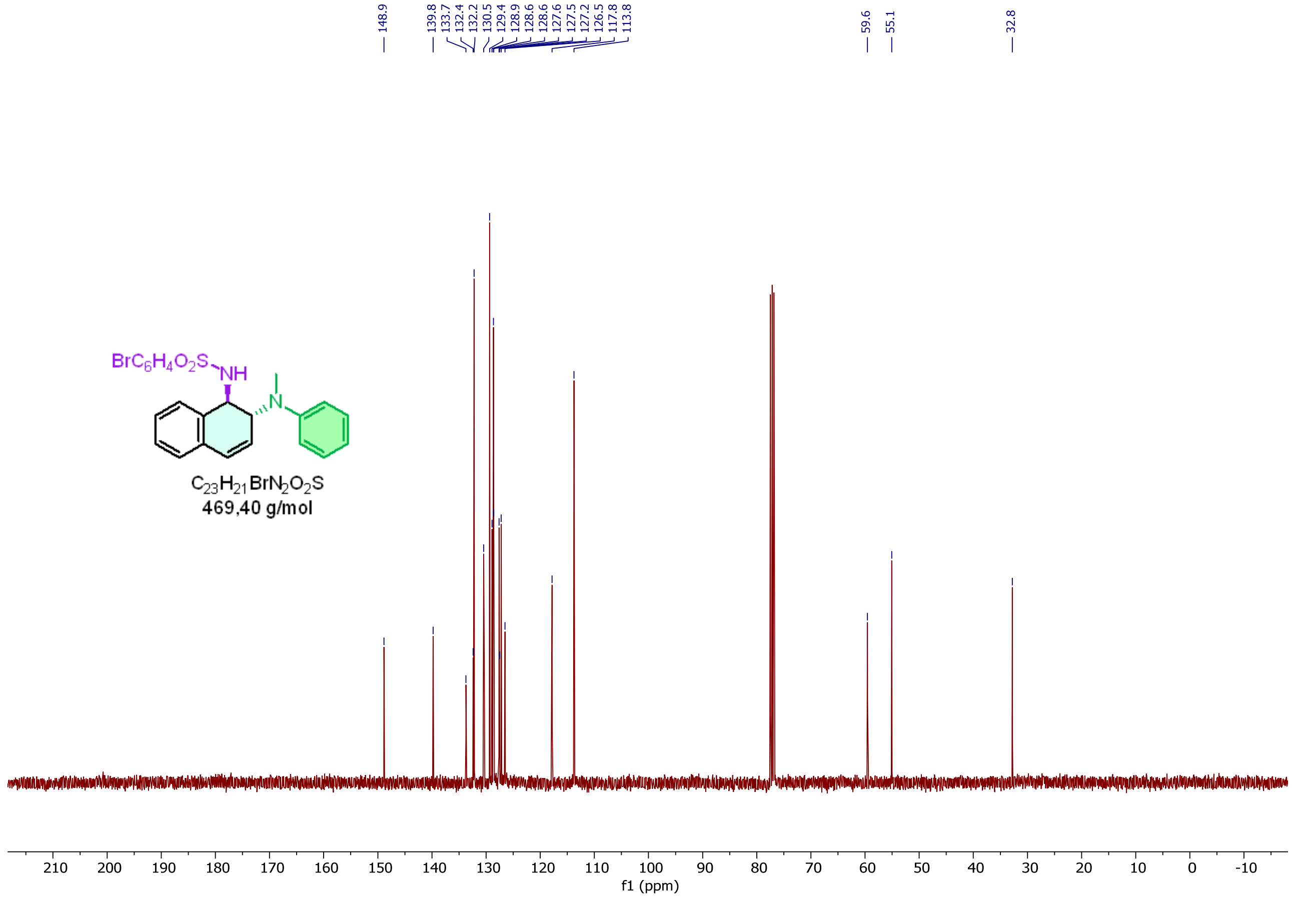

^{1}H NMR (400 MHz, $CDCl_3$) and ^{13}C NMR (101 MHz, $CDCl_3$) Analysis of Compound **7t**

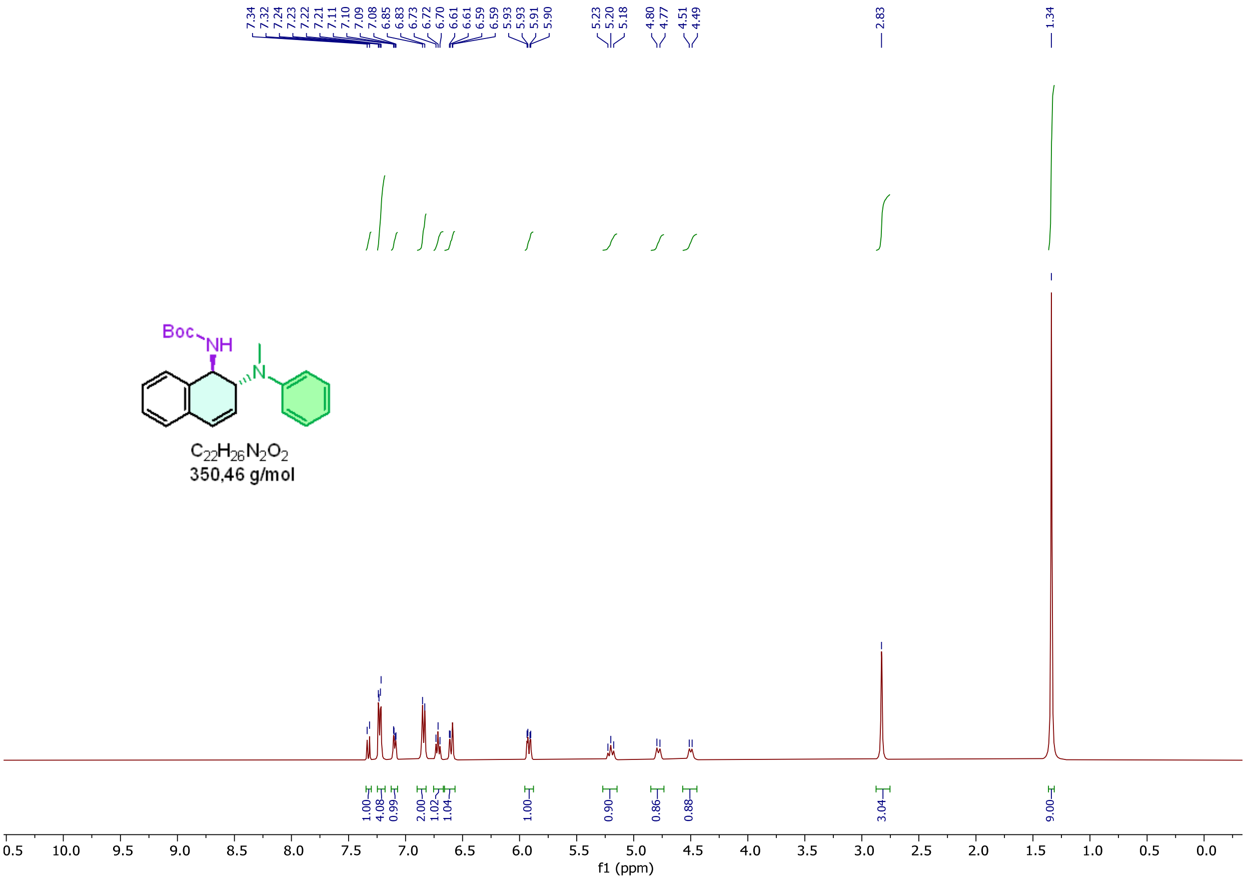

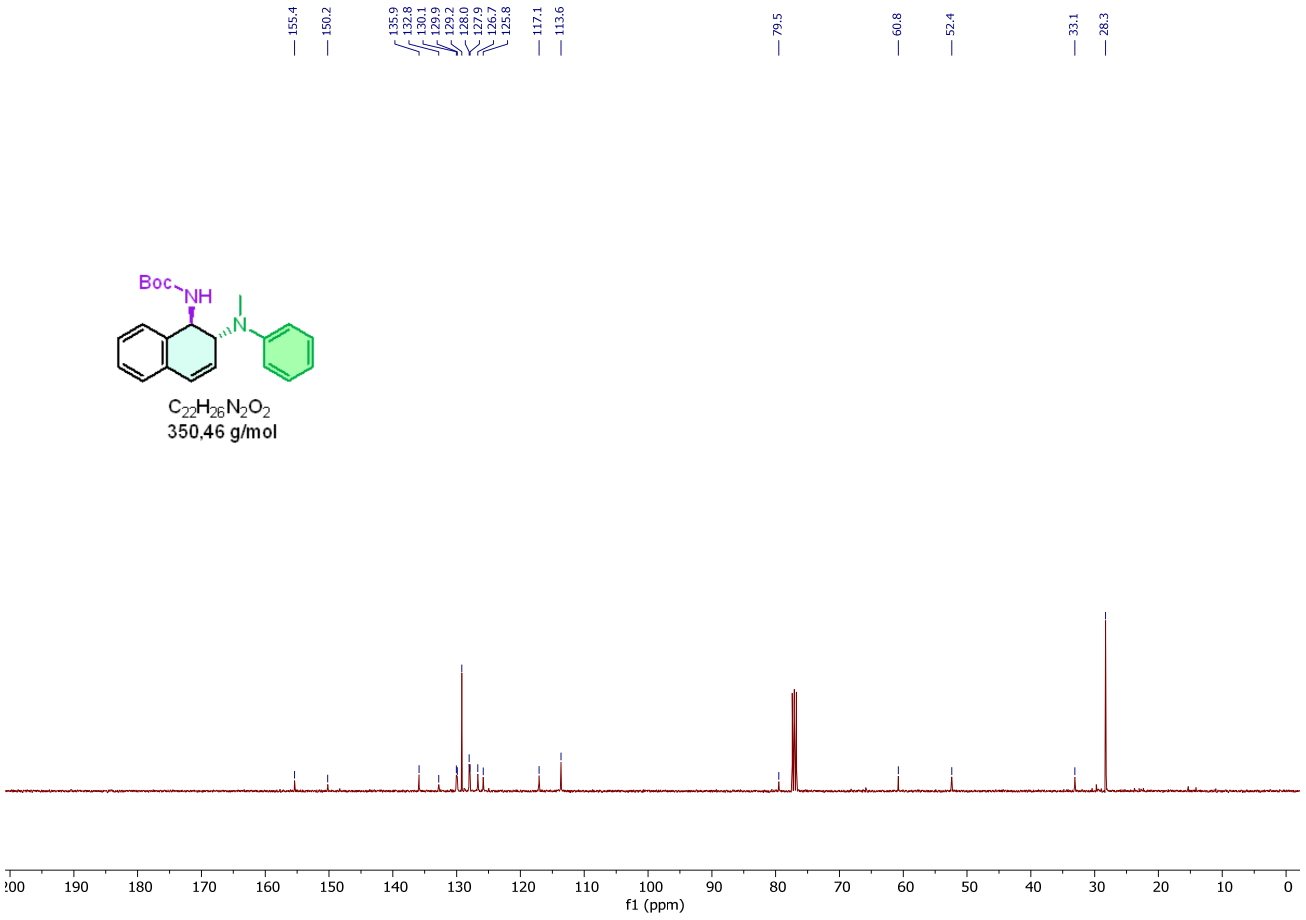

¹H NMR (400 MHz, $CDCl_3$) and ¹³C NMR (101 MHz, $CDCl_3$) Analysis of Compound **7u**

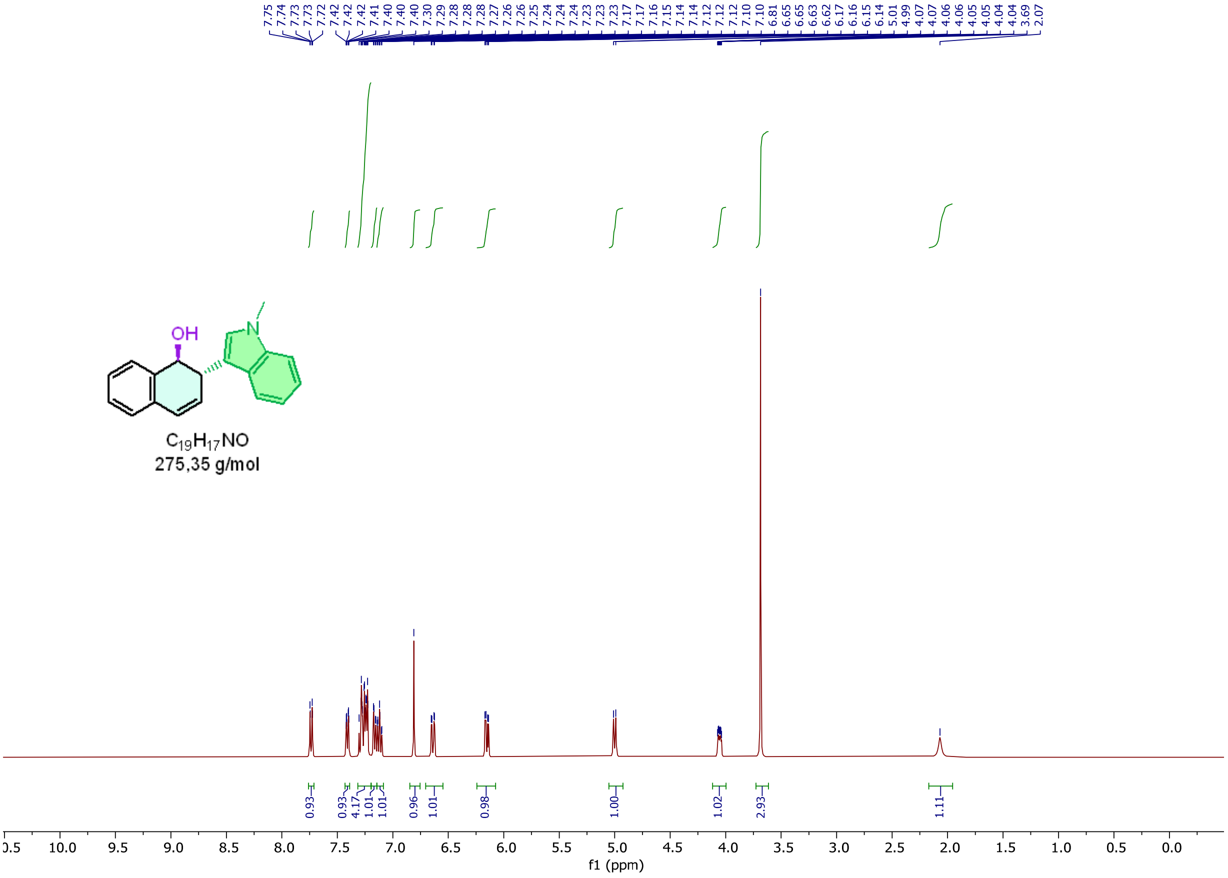

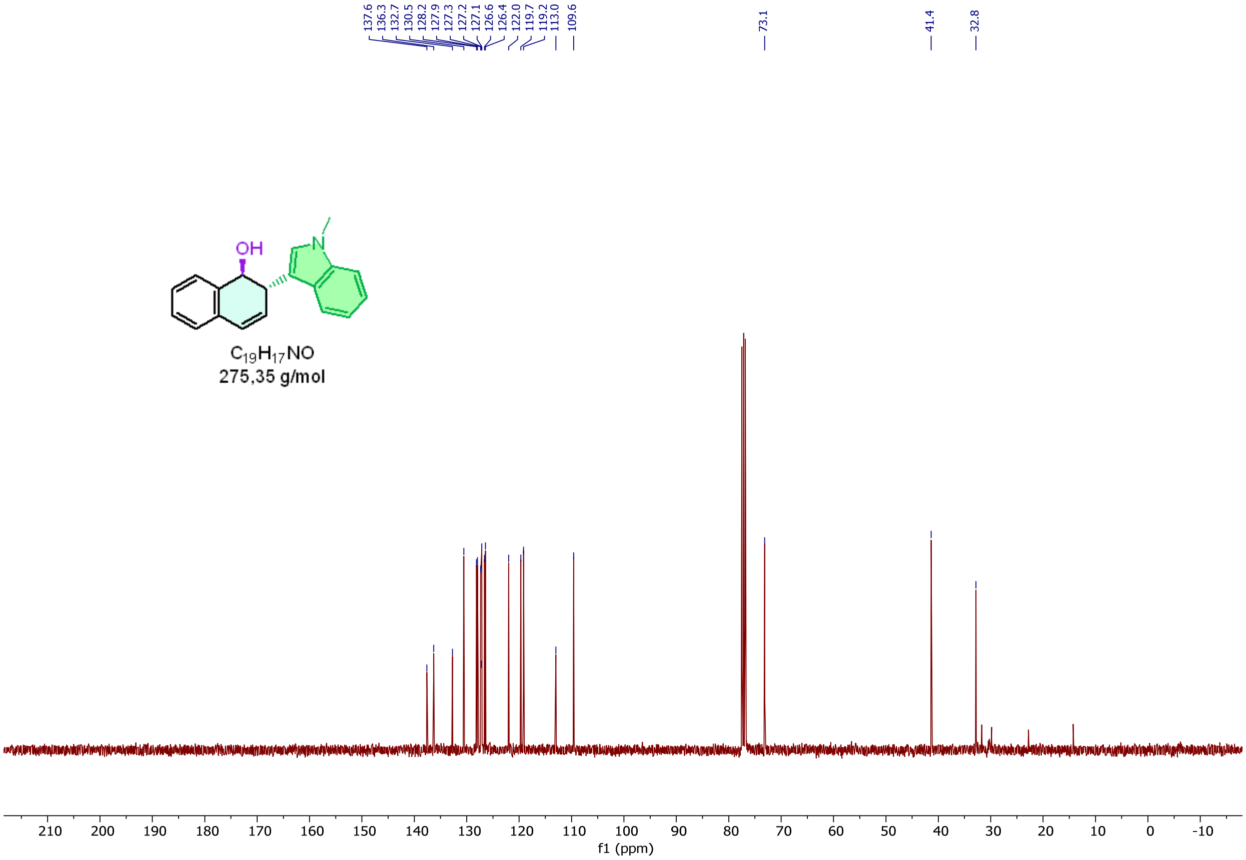

^{1}H NMR (400 MHz, $CDCl_3$) and ^{13}C NMR (101 MHz, $CDCl_3$) Analysis of Compound **7v**

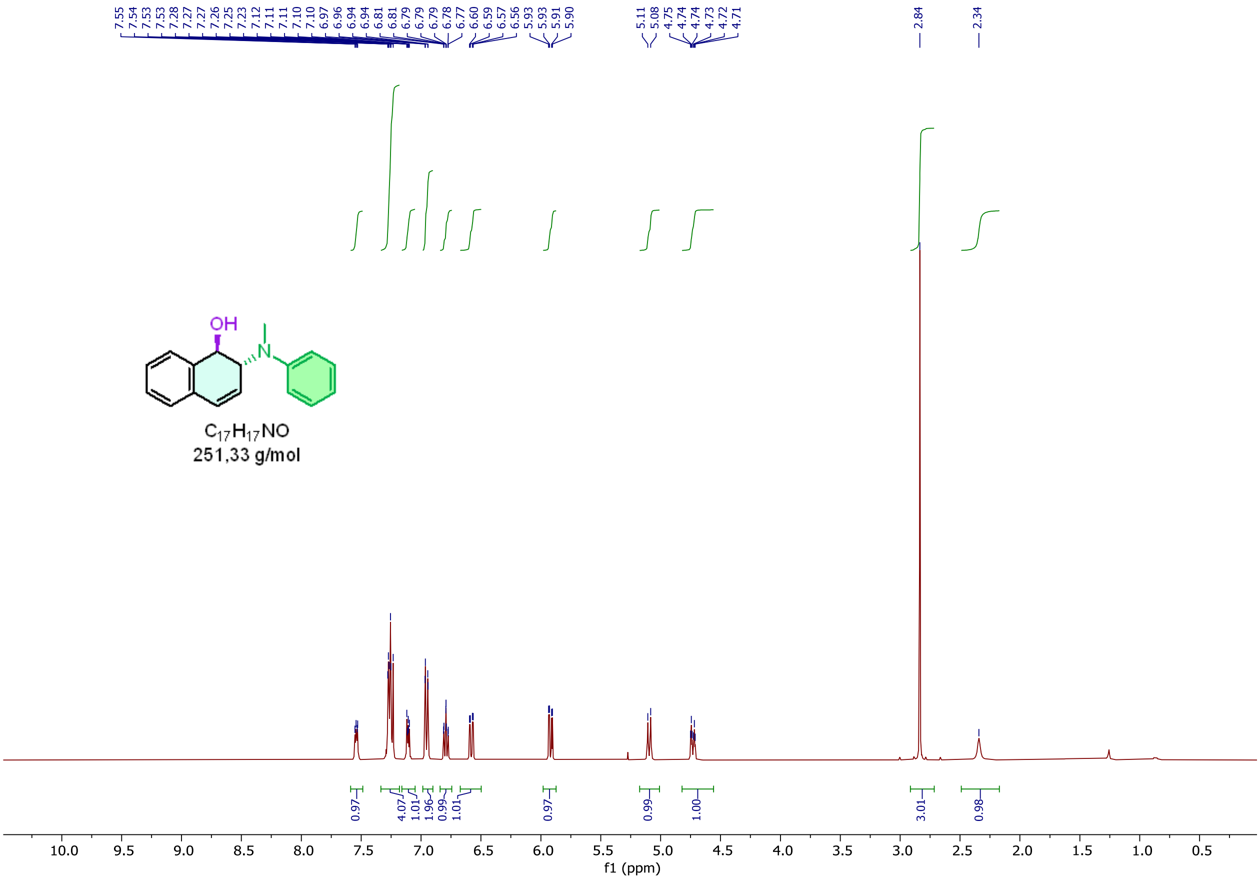

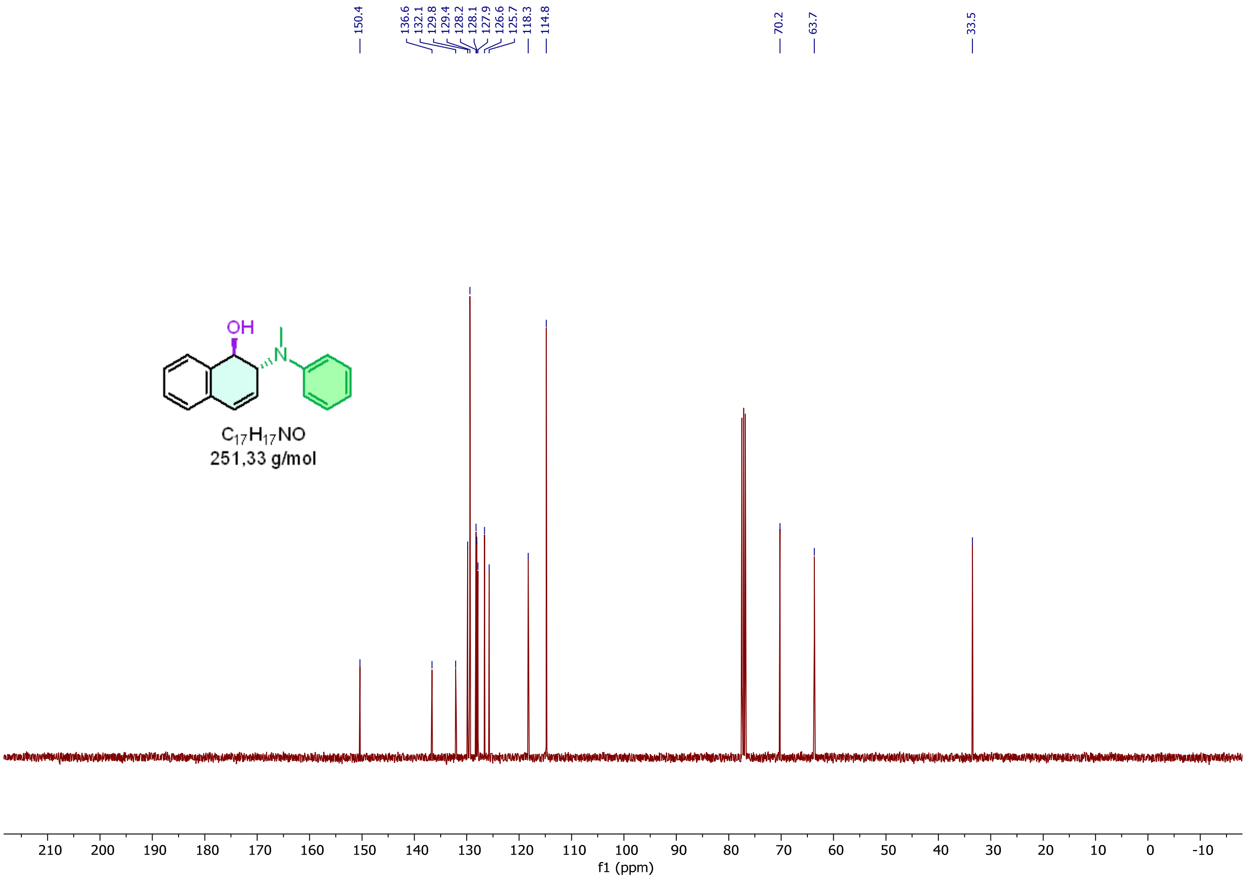

^1H NMR (400 MHz, $CDCl_3$) and ^{13}C NMR (101 MHz, $CDCl_3$) Analysis of Compound **7w**

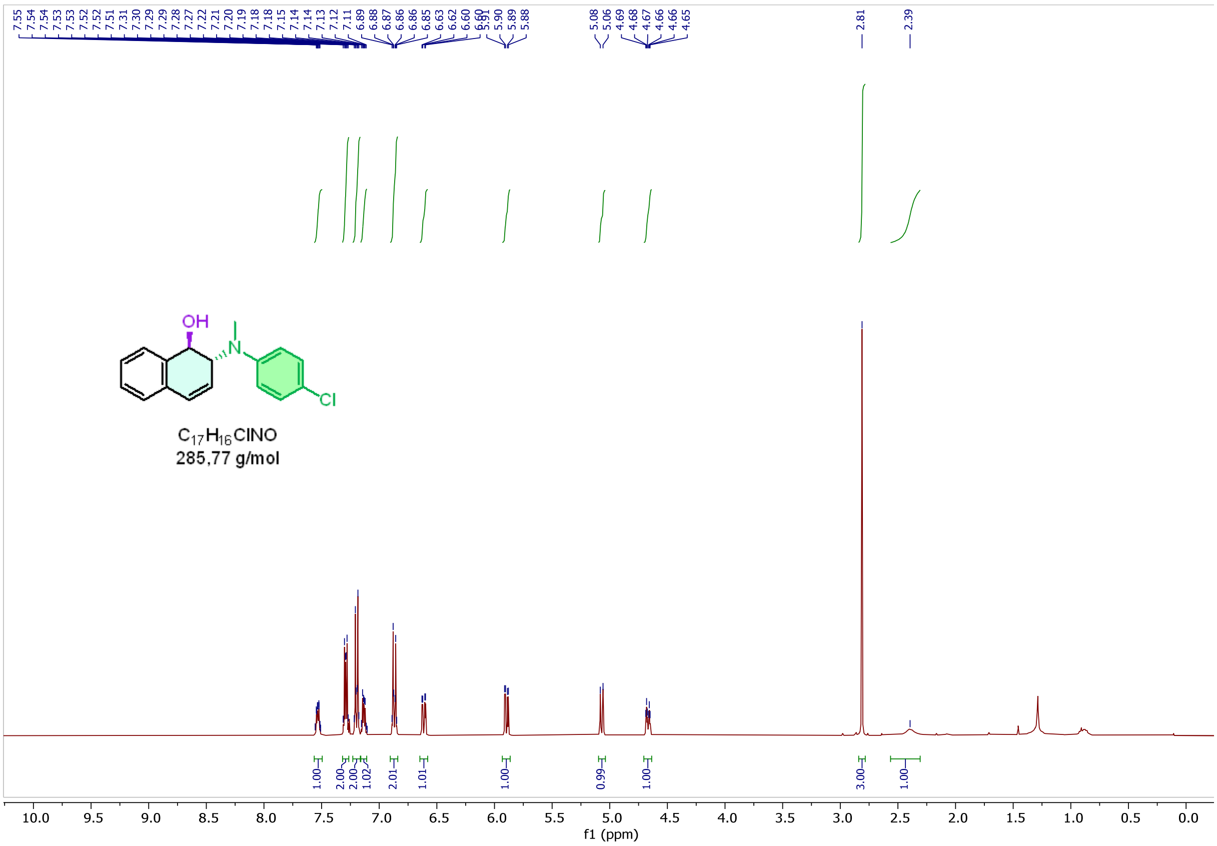

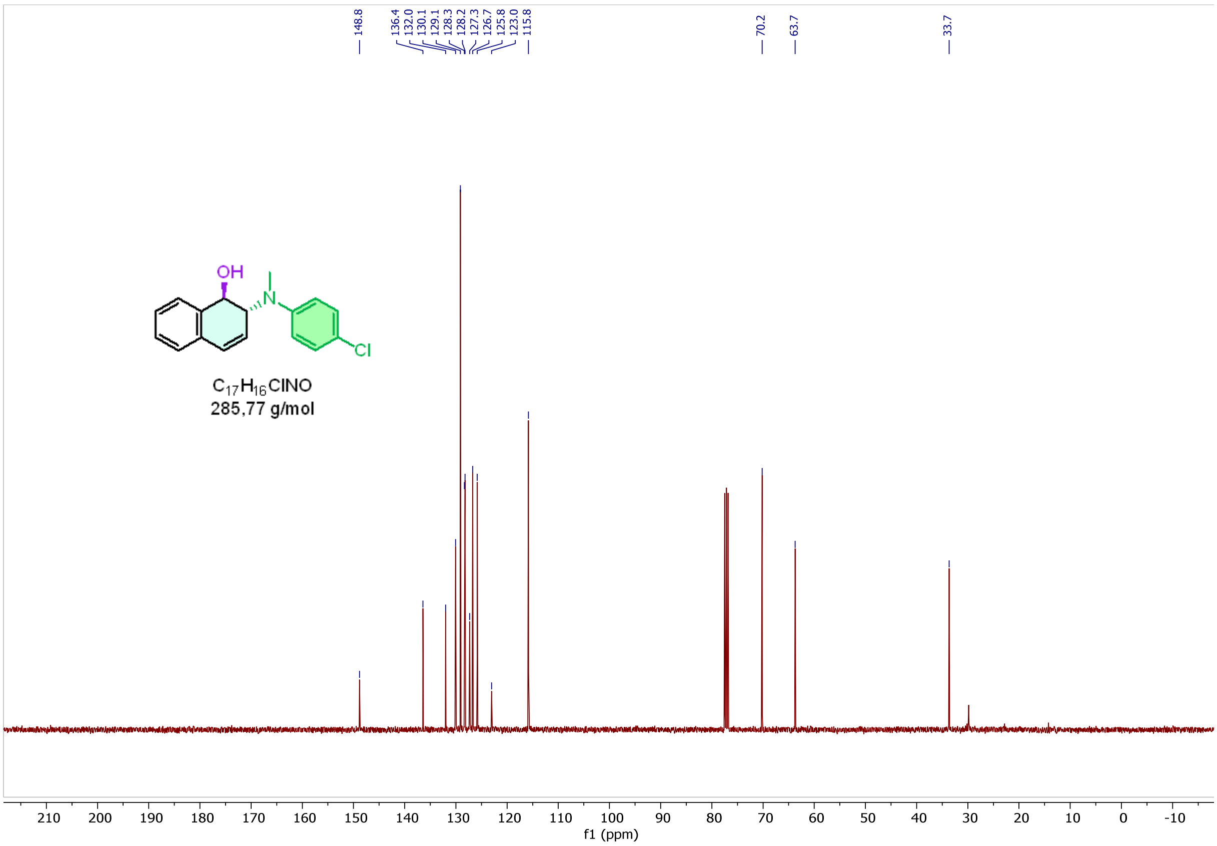

^{1}H NMR (400 MHz, $CDCl_3$) and ^{13}C NMR (101 MHz, $CDCl_3$) Analysis of Compound **7x**

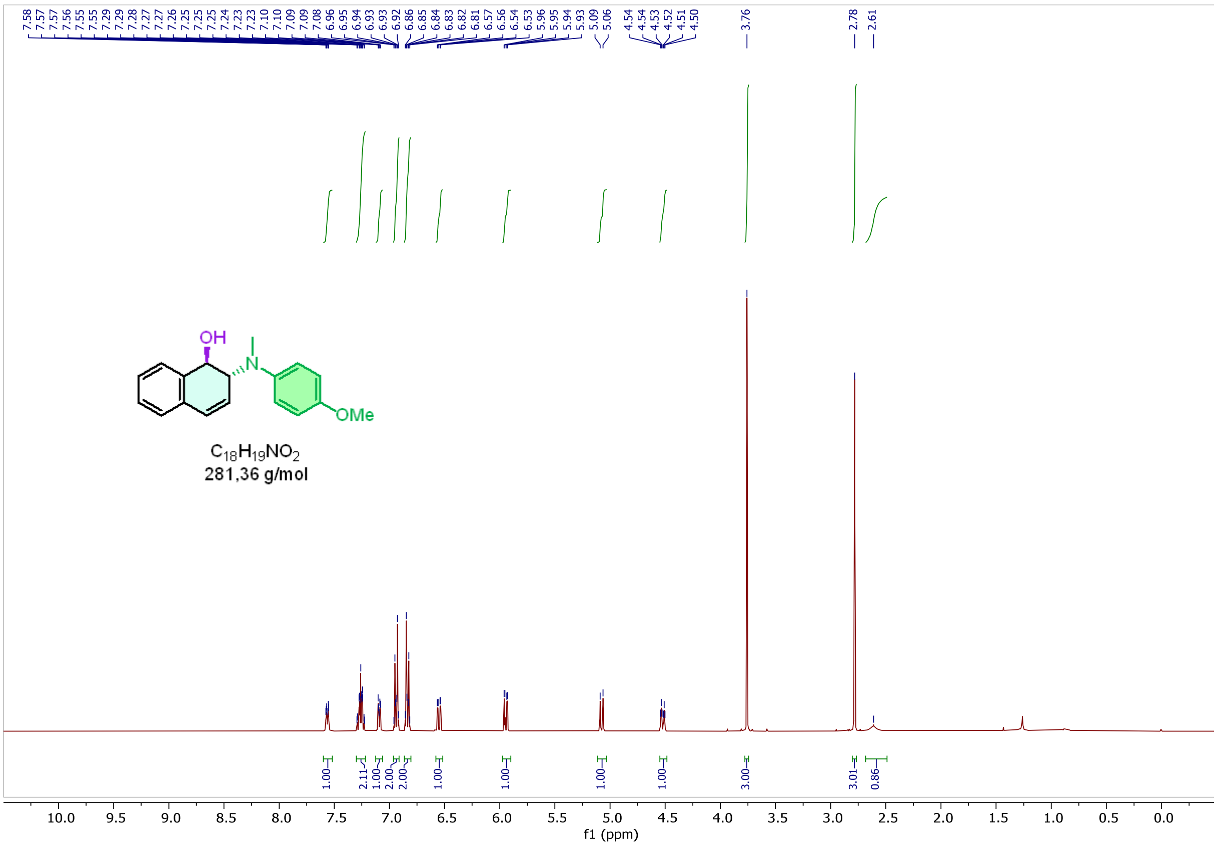

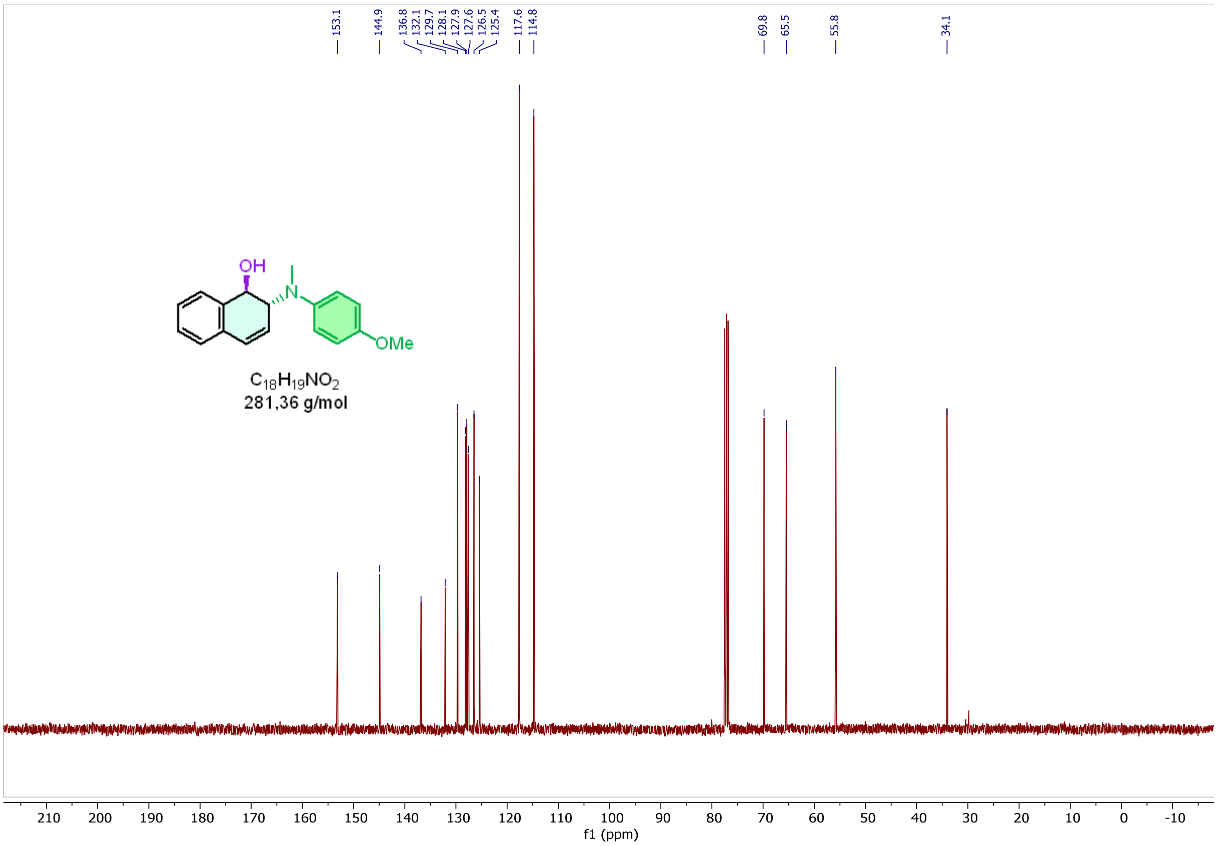

^{1}H NMR (400 MHz, $CDCl_3$) and ^{13}C NMR (101 MHz, $CDCl_3$) Analysis of Compound **7y**

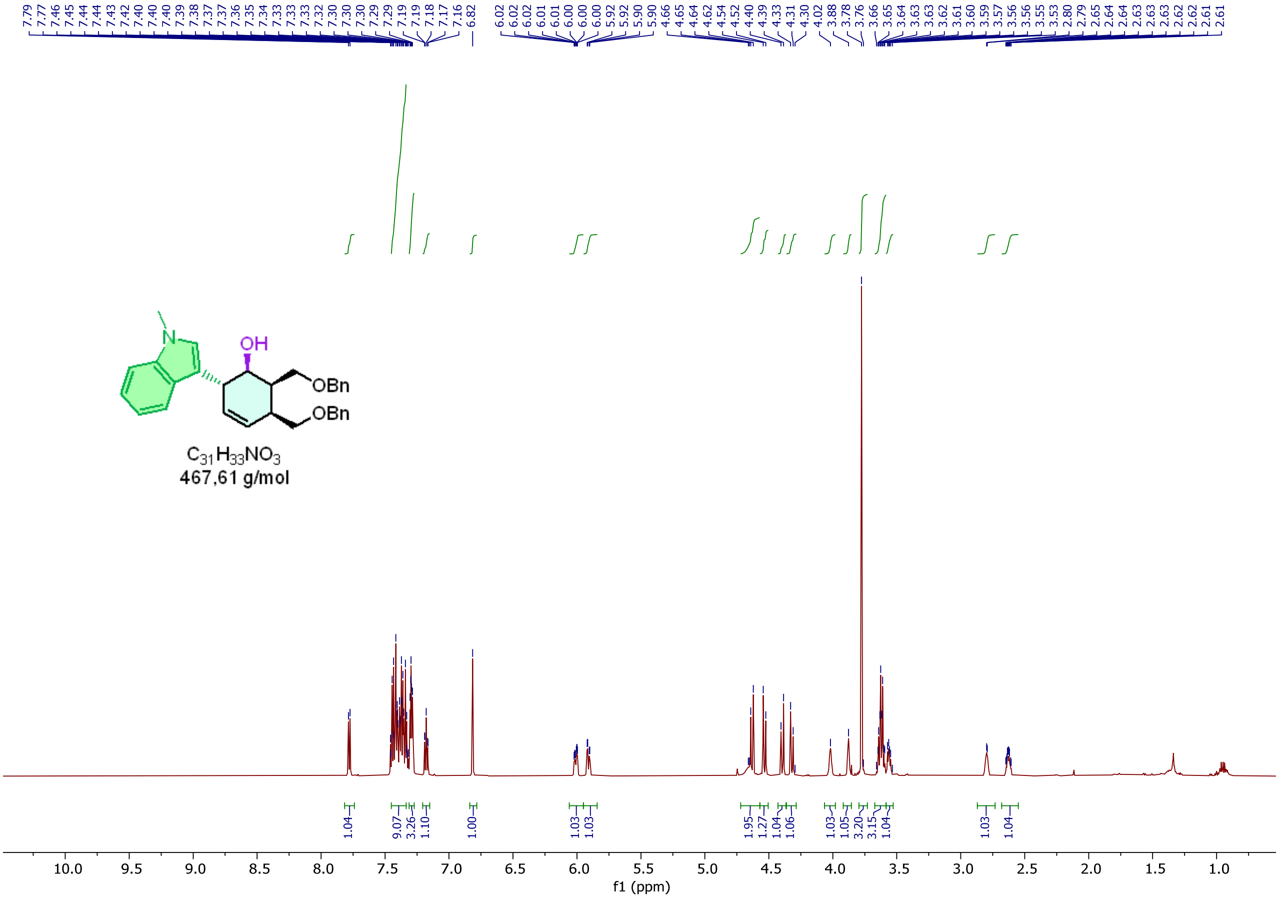

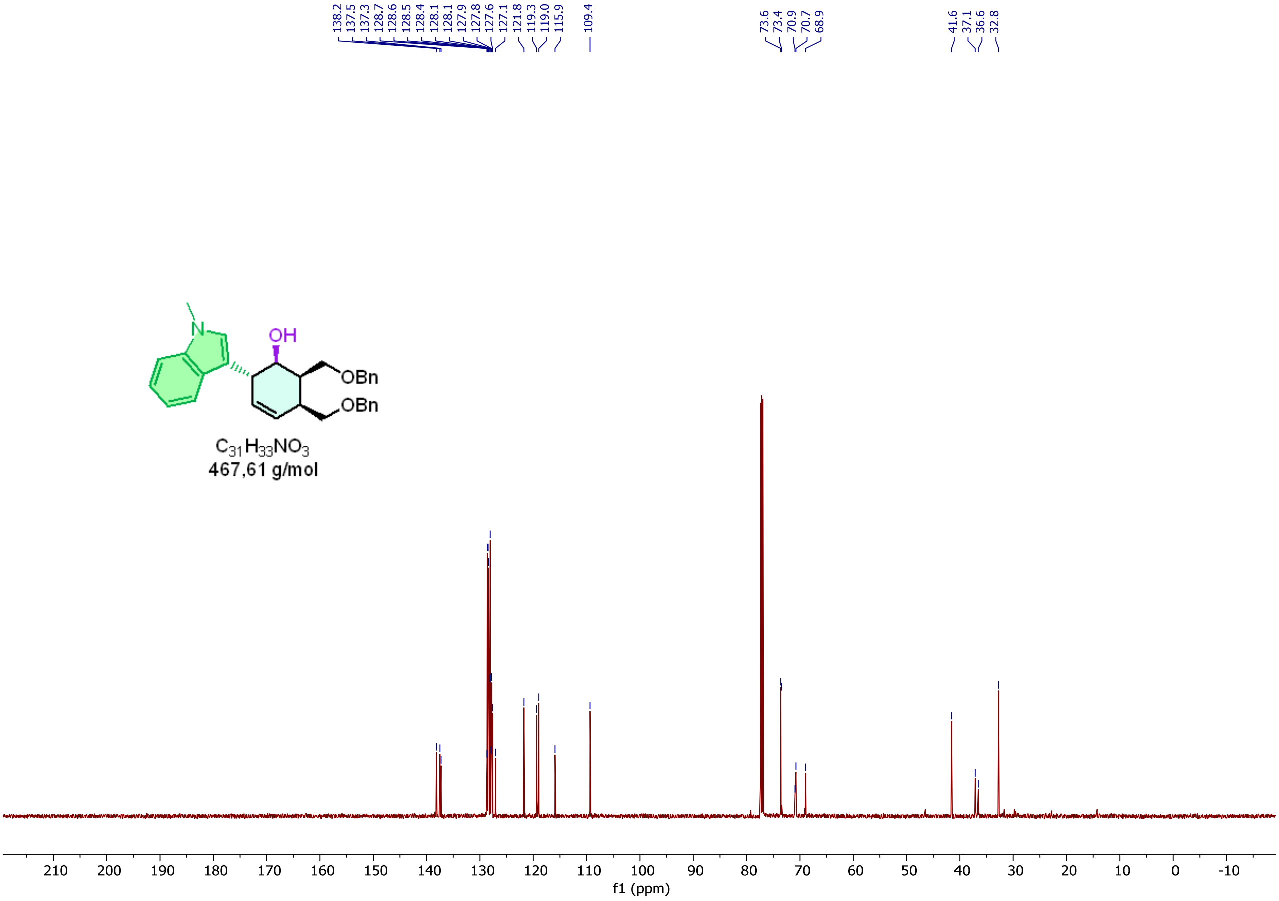

^{1}H NMR (400 MHz, $CDCl_3$) and ^{13}C NMR (101 MHz, $CDCl_3$) Analysis of Compound **8a**

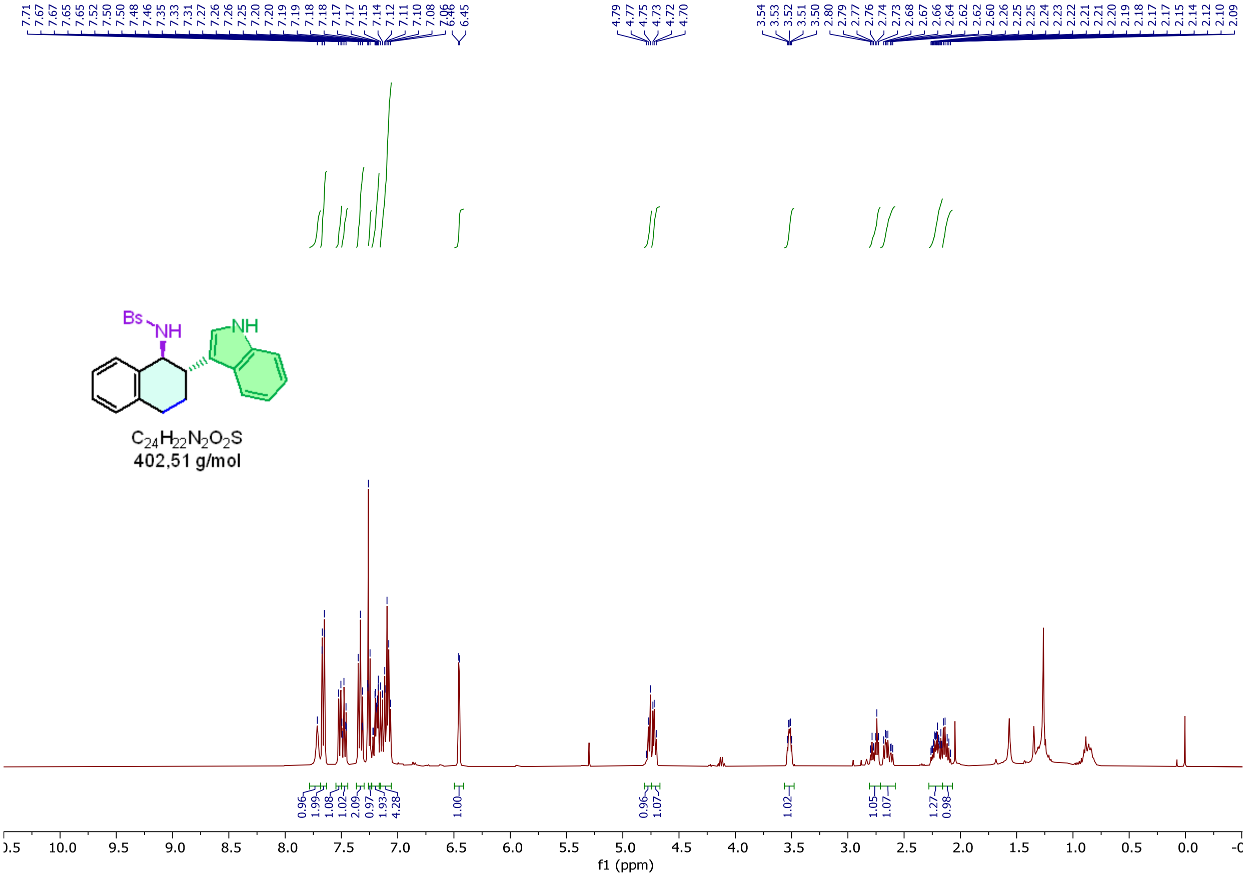

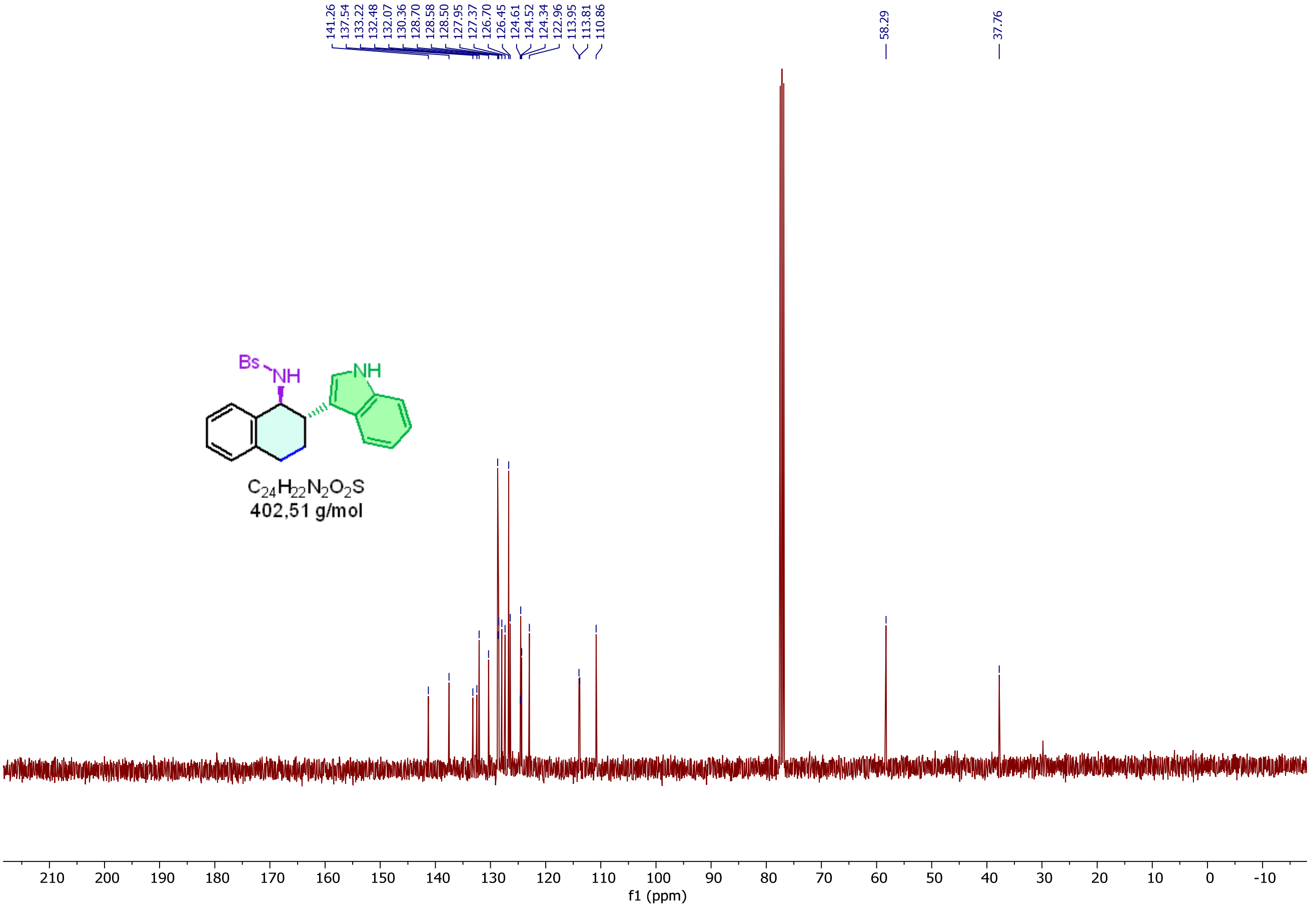

^{1}H NMR (400 MHz, $CDCl_3$) and ^{13}C NMR (101 MHz, $CDCl_3$) Analysis of Compound **8b**

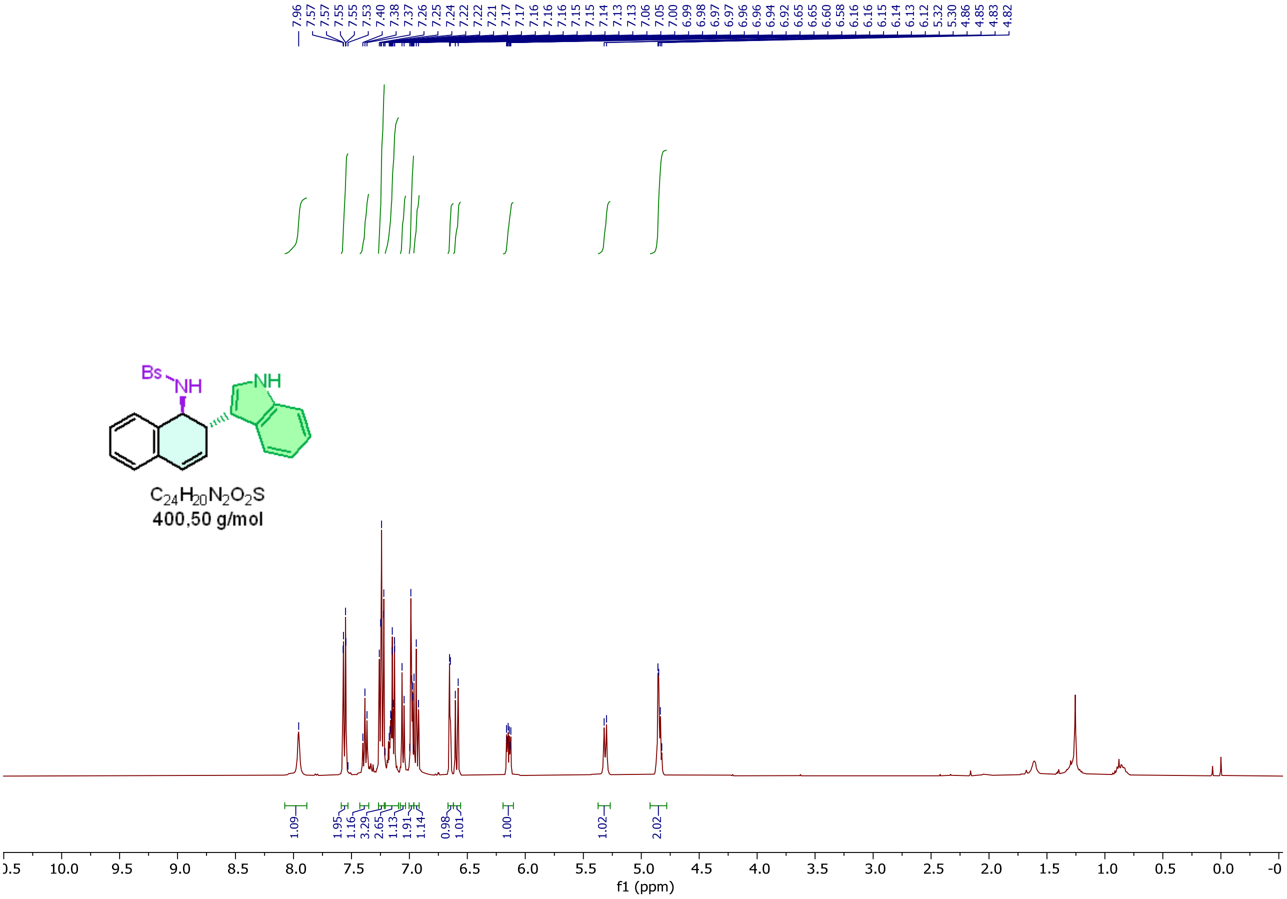

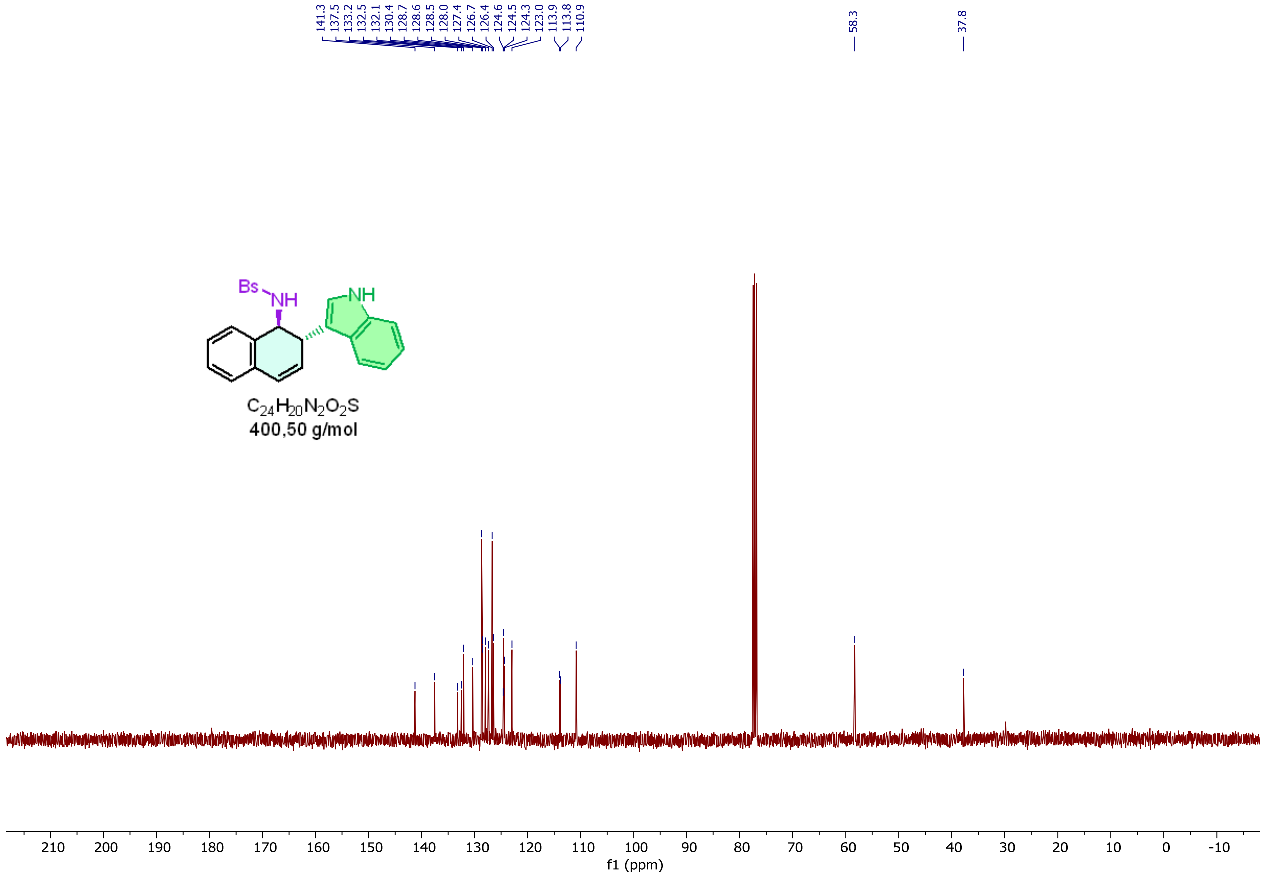

^{1}H NMR (400 MHz, ACN-d$_3$) and ^{13}C NMR (101 MHz, ACN-d$_3$) Analysis of Compound **8c**

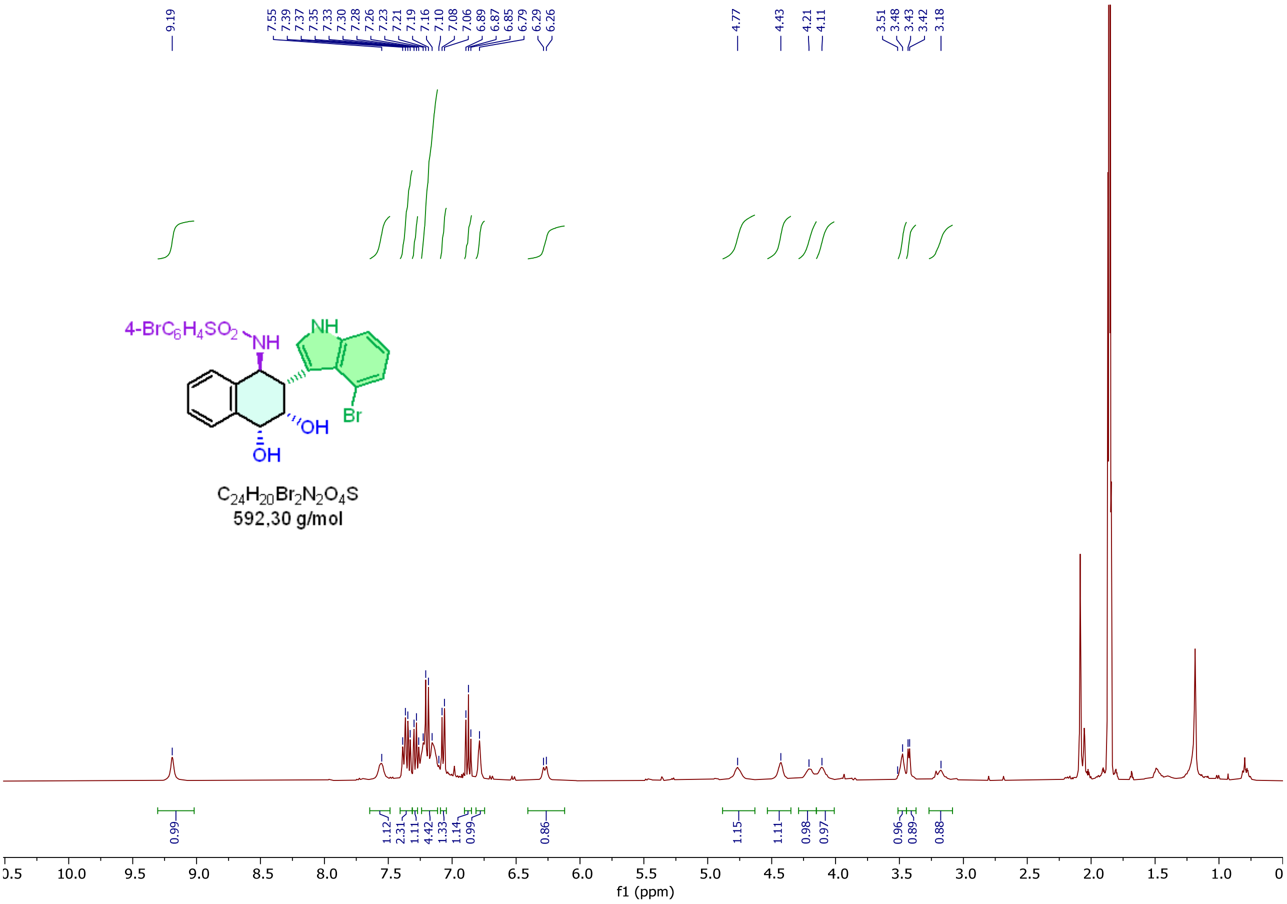

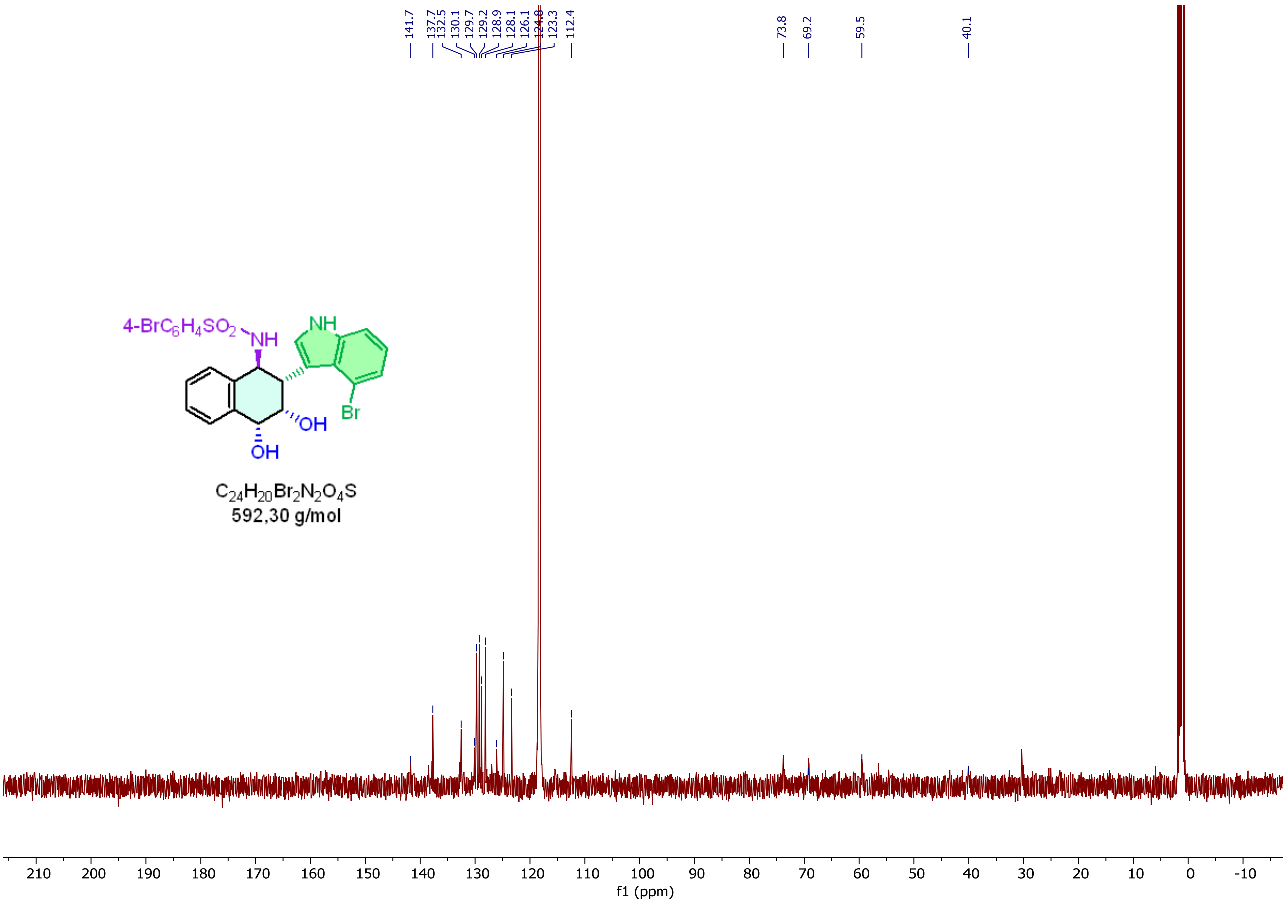

^{1}H NMR (400 MHz, $CDCl_3$) and ^{13}C NMR (101 MHz, $CDCl_3$) Analysis of Compound **8d**

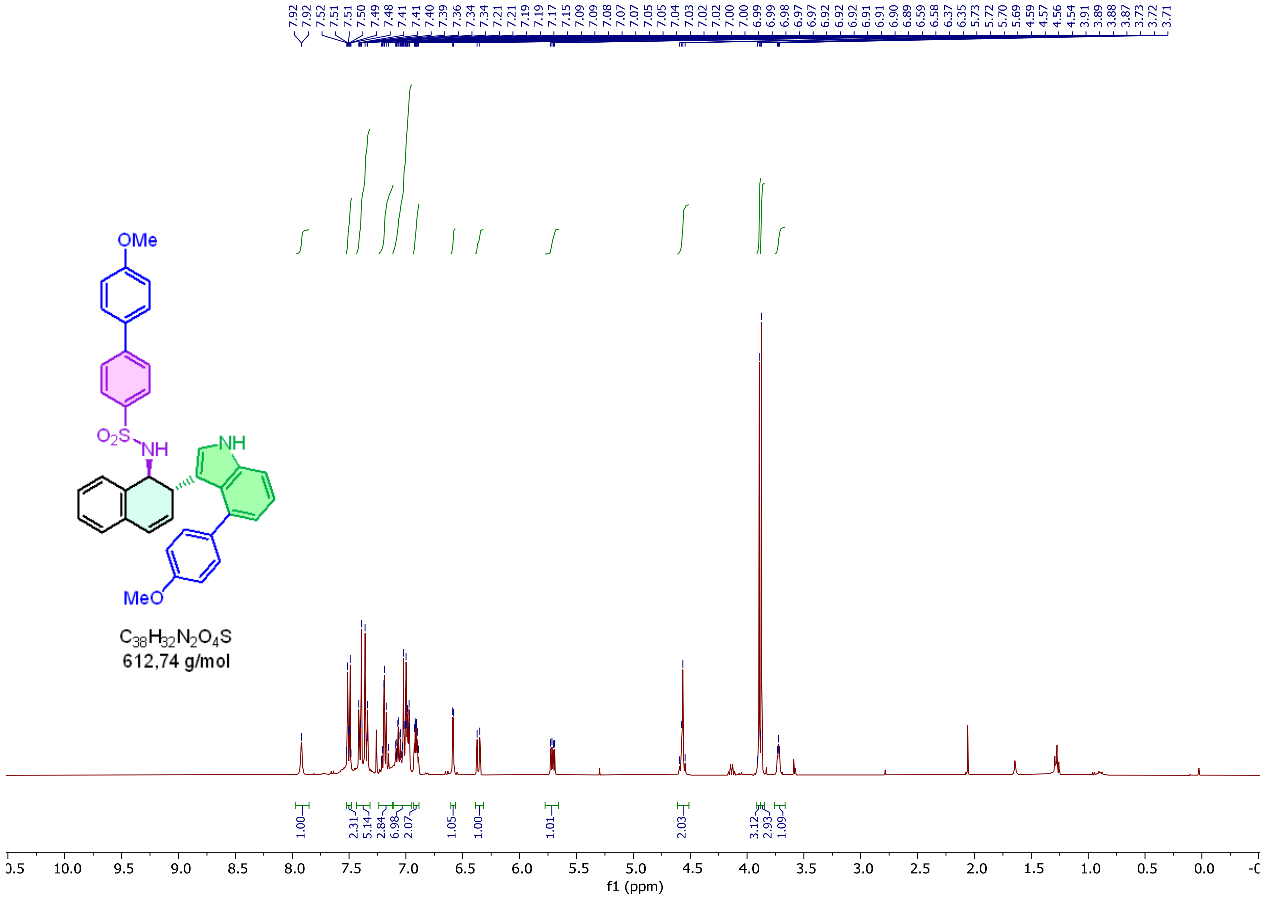

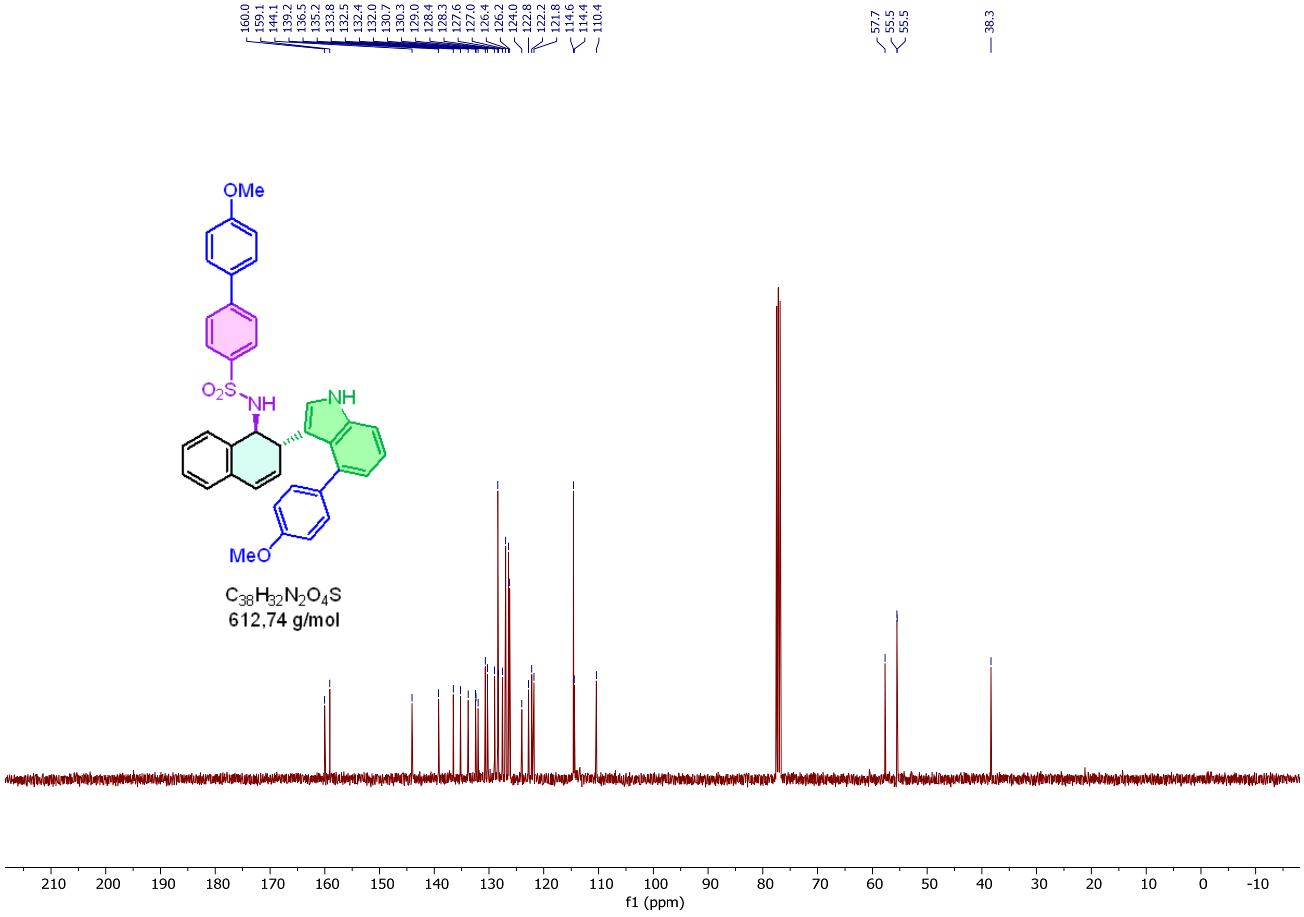

Supplement: Supplementary file 1 [file ol5c02110_si_001.pdf]
